# Discovery of isoquinoline sulfonamides as allosteric gyrase inhibitors with activity against fluoroquinolone-resistant bacteria

**DOI:** 10.1038/s41557-024-01516-x

**Published:** 2024-06-19

**Authors:** Alexander T. Bakker, Ioli Kotsogianni, Mariana Avalos, Jeroen M. Punt, Bing Liu, Diana Piermarini, Berend Gagestein, Cornelis J. Slingerland, Le Zhang, Joost J. Willemse, Leela B. Ghimire, Richard J. H. B. N. van den Berg, Antonius P. A. Janssen, Tom H. M. Ottenhoff, Constant A. A. van Boeckel, Gilles P. van Wezel, Dmitry Ghilarov, Nathaniel I. Martin, Mario van der Stelt

**Affiliations:** 1 Department of Molecular Physiology, Leiden Institute of Chemistry, Leiden University, Leiden, the Netherlands; 2 Biological Chemistry Group, Institute of Biology, Leiden University, Leiden, the Netherlands; 3 Department of Molecular Biotechnology, Institute of Biology, Leiden University, Leiden, the Netherlands; 4 Department of Molecular Microbiology, John Innes Centre, Norwich, UK; 5 Department of Infectious Diseases, Leiden University Medical Center, Leiden, the Netherlands

**Keywords:** Target identification, Antibiotics, Cryoelectron microscopy

## Abstract

Bacteria have evolved resistance to nearly all known antibacterials, emphasizing the need to identify antibiotics that operate via novel mechanisms. Here we report a class of allosteric inhibitors of DNA gyrase with antibacterial activity against fluoroquinolone-resistant clinical isolates of *Escherichia coli*. Screening of a small-molecule library revealed an initial isoquinoline sulfonamide hit, which was optimized via medicinal chemistry efforts to afford the more potent antibacterial LEI-800. Target identification studies, including whole-genome sequencing of in vitro selected mutants with resistance to isoquinoline sulfonamides, unanimously pointed to the DNA gyrase complex, an essential bacterial topoisomerase and an established antibacterial target. Using single-particle cryogenic electron microscopy, we determined the structure of the gyrase–LEI-800–DNA complex. The compound occupies an allosteric, hydrophobic pocket in the GyrA subunit and has a mode of action that is distinct from the clinically used fluoroquinolones or any other gyrase inhibitor reported to date. LEI-800 provides a chemotype suitable for development to counter the increasingly widespread bacterial resistance to fluoroquinolones.

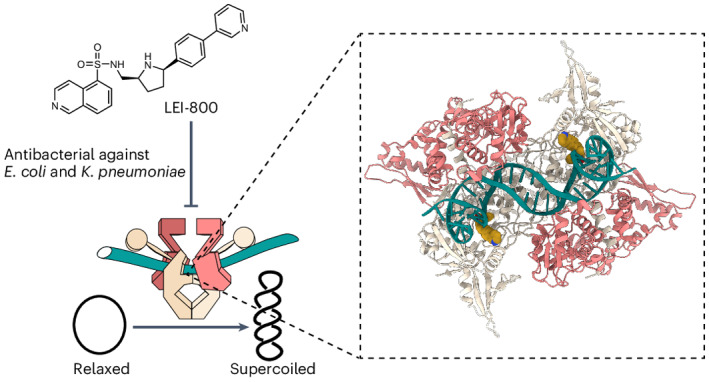

## Abstract (web summary)

Global antibiotic scarcity looms owing to bacterial resistance. Now the discovery of a class of allosteric inhibitors targeting DNA gyrase—essential for bacteria—yields a compound LEI-800 that exhibits activity against fluoroquinolone-resistant *E. coli*. The compound’s unique mode of action, revealed through cryo-EM, makes it a promising candidate for countering bacterial resistance.

## Main

In 2019, an estimated 1.27 million deaths worldwide were directly attributable to antibiotic resistance, 20% of which were linked to drug-resistant *Escherichia coli*^1^. Multi-drug-resistant (MDR) Gram-negative pathogens, such as *E.* *coli* and *Klebsiella pneumoniae* strains displaying resistance to third-generation cephalosporins, carbapenems and fluoroquinolones, are now widespread^2^ and pose a daunting challenge to healthcare systems. At the same time, resistance to antibiotics of last resort^3^ has also been detected in several countries. The emergence of mobilized colistin resistance-1 (*mcr-1*) in 2015 (ref. ^4^) and tigecycline resistance (*tetX3–tetX5*) genes in 2019 (ref. ^5^) threatens to render Gram-negative MDR infections untreatable. Clearly, new antibiotics are urgently needed to keep pace with drug-resistant Gram-negative bacteria.

Target-based screening is an effective approach to identify novel small molecules as hits in drug discovery programs, but so far has been less successful in identifying novel antibiotics^6,7^. Rational modification of existing drugs^8,9^ and phenotypic screening have instead been the more commonly used strategies strategies^10,11^. Historically, phenotypic screens with libraries of natural product extracts have proven to be a fruitful strategy^12,13^. Today, however, the risk of rediscovery and challenges associated with the structure elucidation and chemical synthesis of complex natural products has diminished antibiotic discovery from such sources. As an alternative, phenotypic screening using libraries of small synthetic compounds ensures both synthetic accessibility and structural novelty.

In this Article, we report the discovery of a novel class of isoquinoline sulfonamides with potent antibiotic activity against clinically relevant Gram-negative bacteria. A phenotypic screen of a focused library of synthetic small molecules previously developed in-house, led to hit **1**. A subsequent medicinal chemistry program to map the structure–activity relationships (SAR) of **1** led to the rational design of LEI-800, a compound with enhanced antimicrobial activity against *E.* *coli* and *K.* *pneumoniae*. Bacterial cytological profiling (BCP) and whole-genome sequencing (WGS) of resistant strains, followed by re-introduction of mutations into wild-type (WT) background and biochemical studies led to the unambiguous identification of DNA gyrase as the target for LEI-800. DNA gyrase is an essential bacterial topoisomerase that is targeted by fluoroquinolones for which widespread resistance has been observed in the clinic. Importantly, LEI-800 did not show any cross-resistance with the clinically used fluoroquinolone ciprofloxacin (CIP) or vice versa. Structural biology studies using single-particle cryogenic electron microscopy (cryo-EM) on the gyrase–DNA–LEI-800 complex revealed an unprecedented binding site for LEI-800 distinct from fluoroquinolones or any other known gyrase inhibitor. Further biochemical experiments established that LEI-800 inhibits the enzyme by allosterically inhibiting DNA cleavage. Our discovery of the isoquinoline sulfonamides as allosteric DNA gyrase inhibitors paves the way for the development of a novel class of antibiotics targeting Gram-negative bacteria, including those that have become resistant to fluoroquinolones.

## Results

### In-house antibacterial screen unveils isoquinoline sulfonamide

**Fig. 1:**
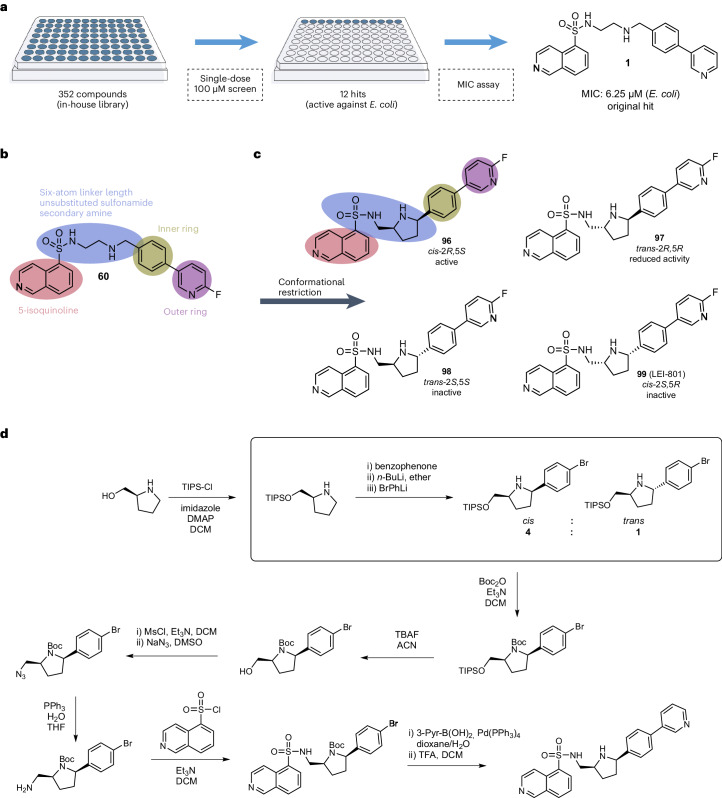
Initial hit finding and synthetic optimization strategy. **a**, The initial library screen of 352 compounds at a single 100 µM dose. The 12 hits found were further investigated in MIC assays, resulting in the most potent hit **1**. **b**, SAR overview of **1**. The 5-isoquinoline (coral), linker (blue), inner ring (gold) and outer ring (purple) were systematically modified using structure **1** as reference. The essential groups are noted in corresponding colours. **c**, Conformational restriction using a pyrrolidine linker retains all the core molecular features, while reducing loss of entropy upon binding. The *trans*-2*R*,5*S* isomer is the only diastereomer that is more potent than the parent compound. The *trans*-2*R*,5*R* isomer still inhibits growth but at higher concentrations, while the *cis*-2*S*,5*R* and the *trans*-2*S*,5*S* isomers both do not show activity. **d**, Synthetic route to make conformationally restricted isoquinoline sulfonamide LEI-800. The synthesis consists of 12 individual steps, of which steps 2–4 introduce a second stereocentre. The synthetic procedures are found in full in Supporting Information. DMAP, dimethylaminopyridine; THF, tetrahydrofuran; ACN, acetonitrile; DCM, dichloromethane.

To identify novel antibiotics with activity against Gram-negative bacteria, we screened a diverse library of 352 small molecules previously assembled for other projects in our laboratory. These compounds were tested for activity against *E.* *coli* at an initial concentration of 100 µM (Fig. 1a and Supplementary Data 1). This screen identified 12 compounds that inhibited bacterial growth, with isoquinoline sulfonamide **1** being the most potent with a minimum inhibitory concentration (MIC) of 6.25 µM (2.6 µg ml^−1^). Further testing on other bacterial species (Supplementary Table 7) revealed that antimicrobial activity was also exhibited against *K.* *pneumoniae* (MIC of 12.5 µM).

### Structure–activity relationship study reveals activity cliffs

Compound **1** was previously synthesized in a SAR study^14^ involving kinase inhibitors^15^. To explore the antibacterial properties of this scaffold, we rescreened 45 analogues (**2**–**46**), previously synthesized as part of that program, against *E.* *coli* (Supplementary Table 1 and Supplementary Data 2). Interestingly, we observed steep activity cliffs, as no other compound showed antimicrobial activity (MIC >50 µM). A clear difference between compound **1** and the other compounds tested was the six-atom linker between the isoquinoline and the inner phenyl ring present in **1** (Fig. 1b). With this in mind, we systematically modified the isoquinoline and the inner and outer aromatic rings, while keeping the linker constant (Supplementary Data 3). First, we investigated the importance of the isoquinoline by synthesizing compounds **47**–**55** (Supplementary Table 2) using an amine building block, which was reacted with either in situ-generated sulfinates^16^ or commercially available sulfonyl chlorides (for synthesis schemes, see Supplementary Information). Compound **47** (in which the nitrogen of the isoquinoline was substituted for a carbon atom) showed a fourfold reduced activity (Supplementary Table 2), whereas compound **48** (phenyl) lost all activity. This suggests that reduced electron density of the isoquinoline ring might be important for activity and/or that the nitrogen accepts a hydrogen bond. Compounds **49**–**55** contained structural motifs that were previously shown to form more hydrogen bonds with backbone amides of proteins than an isoquinoline moiety, thereby enhancing the potency or specificity of the interaction. In this case, compounds **49**–**55** were inactive, which was taken to suggest that the isoquinoline might reside in a hydrophobic pocket or that these groups may reduce cell permeability. Next, the electronic and hydrophobic properties of the outer aryl ring were systematically studied by applying the Topliss scheme^17^. To this end, compounds **56**–**78** were synthesized through Suzuki reactions of the phenyl bromide building block with a range of boronic acids (for synthesis schemes, see Supplementary Information and Supplementary Table 3). The nitrogen in pyridinyl (**1**) was shown not to be important because compound **56** with a phenyl ring or a thiophene as a phenyl bioisoster (**57**), but not furan (**58**), were equally potent. Small-electron withdrawing substituents (for example, a fluoro (**59** and **60**) or chloro (**61**) group), but not more polar or donating substituents (**62**–**65**), were tolerated. Of note, compound **59** with a para-fluoro group showed a twofold increase in potency with a MIC of 3.1 µM on *E.* *coli* and was the most potent compound identified in this series, while all compounds with ortho or meta substituents (**66**–**75**) demonstrated a reduced potency (Supplementary Table 3). Introduction of a second ring on the phenyl group (compounds **76**–**78**) resulted in a complete loss of potency, which may indicate the presence of a restricted binding pocket or lack of cell penetration. Introducing a nitrogen in the inner phenyl ring (compounds **79**–**82**) to reduce lipophilicity or an ortho substituent (compounds **83**–**90**) to create a rotational barrier did not improve potency (Supplementary Table 4). Finally, we investigated the importance of the sulfonamide and secondary amine in the six-atom linker (Supplementary Table 5). Replacement of the sulfonamide with an amide (**91**) or substituting the nitrogen with a methyl group (**92**) led to a loss of activity, suggesting that the sulfonamide is important for directing the isoquinoline into a specific orientation. Replacing the secondary amine with an amide (**93**) or ether (**94**), as well as substituting it with a methyl group (**95**), abolished all activity, thereby suggesting the secondary amine may form an important hydrogen bond or is essential for cellular uptake. Although, the isoquinolines are in general more potent in *E.* *coli* than in *K.* *pneumoniae*, the observed trends in the SAR were similar (Supplementary Tables 2–5). The most active compounds identified (**56**, **57**, **59**, **60**, **61**, **63** and **89**) were tested for cytotoxicity using human kidney (HEK293T) and liver (HepG2) cell lines (Supplementary Table 6 and Supplementary Fig. 1). Compound **60** was found to show the lowest cytotoxicity and was therefore selected as the lead for further development.

### Conformational restriction results in discovery of LEI-800

The initial SAR study revealed a steep SAR that could potentially be explained by a defined binding pocket on a specific target and/or essential molecular features required for cellular uptake. It has previously been observed that cell penetration of antibiotics in Gram-negative bacteria can be enhanced by conformational restriction^18^, so we hypothesized that reducing the number of rotatable bonds in the linker in the isoquinoline sulfonamide series might increase antibiotic activity. To this end, we designed four conformationally restricted diastereomeric derivatives (**96**–**99**) based on compound **60** in which we replaced the secondary amine linker with a 2,5-disubstituted pyrrolidine motif (Fig. 1c). In doing so we employed the enantiomerically pure O-triisopropylsilyl (TIPS) protected prolinol building blocks, after which the second chiral centre was introduced through alpha-functionalization of the pyrrolidine with lithiated dibromobenzene, forming a new carbon–carbon bond (Fig. 1d and Supplementary Information for synthesis). Following the procedures of Seidel et al.^19,20^, both the *cis* and *trans* isomers were obtained and separated by column chromatography. After Boc protection of the pyrrolidine, the TIPS-protected hydroxyl was converted to the primary amine in four steps: tetra-*n*-butylammonium fluoride (TBAF) deprotection generated the free alcohol that was in turn converted to the mesylate followed by azide displacement and reduction under Staudinger conditions. The isoquinoline moiety and phenyl substituents were then introduced under similar conditions as in the original scaffold, followed by acidic deprotection of the Boc group.

**Fig. 2:**
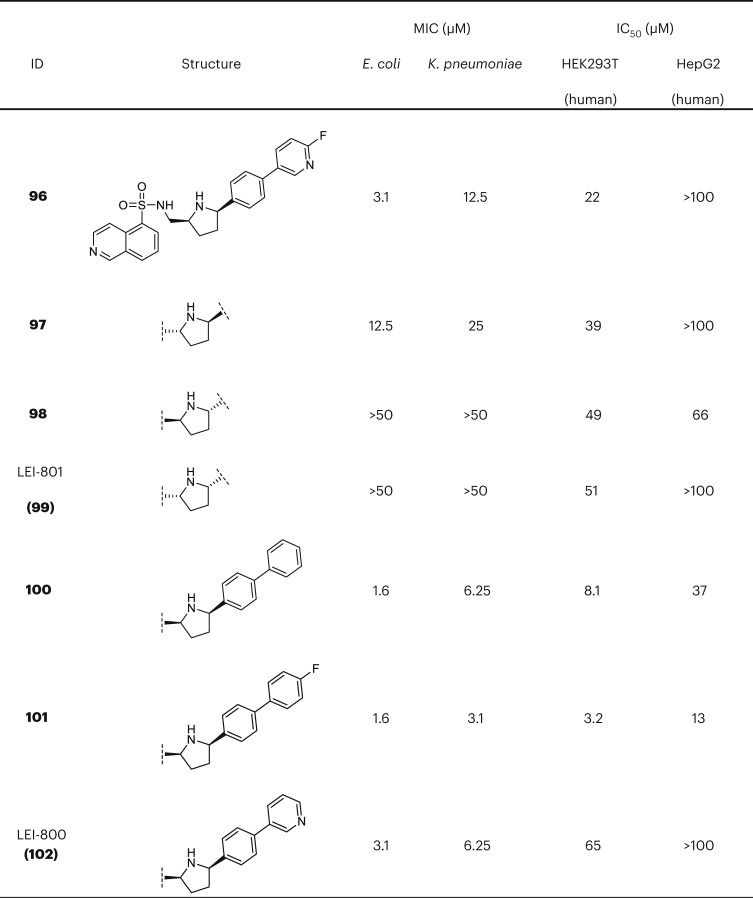
Selected derivatives of hit 1 with their antibacterial potency and human cell cytotoxicity. *E. coli: Escherichia coli* W3110; *K. pneumoniae: Klebsiella pneumoniae* ATCC 29665. For complete set of structures see Supplementary Tables 1–6. Cytotoxicity data in Supplementary Fig. 1.

With the diastereomers **96**–**99** in hand, we tested their activity against *E.* *coli* and *K.* *pneumoniae*. Notably, we found that diastereomer **96** bearing the *cis*-2*R*,5*S* configuration was much more active than the others and also showed a twofold increase in potency compared with compound **60** (Fig. 2). The other diastereomers were either fourfold less active (*trans*-2*R*,5*R* (**97**)) or completely inactive (*trans*-2*S*,5*S* (**98**) and *cis*-2*S*,5*R* (**99**)). Building from this finding, three outer ring derivatives of **96**, based on the earlier SAR, were synthesized (**100**–**102**) and tested in the MIC and cytotoxicity assays (Fig. 2). Compounds **100** and **101**, phenyl and *p*-fluorophenyl analogues, respectively, were found to be most potent with MICs of 1.6 µM, but both also displayed substantial cytotoxicity. In contrast, compound **102** containing a pyridinyl substituent was found to exhibit a MIC of 3.1 µM, a more favourable cytotoxicity profile (half-maximum inhibitory concentration (IC_50_) HEK293T of 65 µM and IC_50_ HepG2 >100 µM), and no haemolytic activity (Fig. 2 and Supplementary Fig. 2).

Also of note, compound **102** was found to be bactericidal against *E.* *coli*, when evaluated at higher concentrations (Supplementary Fig. 3). In line with the activity profile of the parent compounds, **102** showed selectivity towards the Gram-negative strains *E.* *coli* and *K.* *pneumoniae* when tested against a panel of pathogenic bacteria (Supplementary Table 7). Furthermore, this activity was maintained when **102** was tested on several clinical isolates of *E.* *coli* (Supplementary Table 8), including MDR, *mcr-1* positive and extended-spectrum beta lactamase-producing strains. In view of this excellent profile, we selected compound **102** for further profiling and termed it LEI-800, whereas closely related inactive compound **99** (LEI-801) was chosen as a negative control compound.

### LEI-800 induces distinct morphological profile in bacteria

**Fig. 3:**
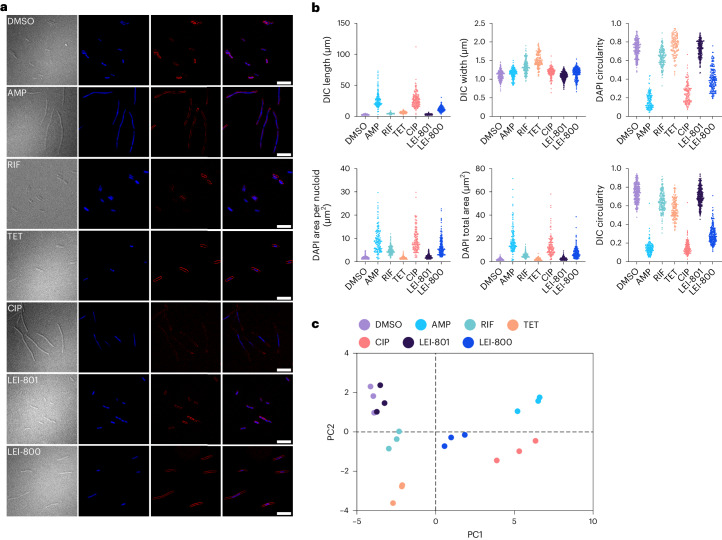
BCP. **a**, Microscopy images of *E.* *coli* BW25113 from the BCP experiment. Conditions are, from top to bottom: DMSO (1.25%), AMP (12.5 µM), RIF (12.5 µM), TET (6.25 µM), CIP (0.0625 µM), LEI-801 (6.25 µM) and LEI-800 (6.25 µM). From left to right, the channels indicate DIC, DAPI, FM4-64 and DAPI/FM4-64 combined. The bacteria treated with LEI-801 displayed a phenotype that is consistent with the DMSO control, while incubation with LEI-800 led to a phenotype resembling the CIP and TET morphological profile. Scale bars, 10 μm. **b**, Individual graphs of six exemplary features extracted from BCP microscopy after image analysis. Each dot represents the measured value for an individual bacterium. **c**, PCA plot of all the morphological features extracted from three independent BCP experiments.

To gain insights into the mode of action (MoA) of LEI-800, we employed an imaging-based approach termed BCP, developed by Pogliano and co-workers^21^. This method exploits the different morphological changes bacteria exhibit in response to antibiotics with differing MoAs. BCP allows for the quantification of these different phenotypes and, by constructing a reference map of antibiotics, the phenotypic similarity of cells treated with LEI-800 was compared with cells treated with a number of clinically used reference antibiotics. To this end, the morphological effects of LEI-800 on *E.* *coli* were compared with a known cell wall synthesis inhibitor (ampicillin (AMP)), an RNA synthesis inhibitor (rifampicin (RIF)), a protein synthesis inhibitor (tetracycline (TET)) and a DNA synthesis inhibitor (CIP) along with negative control (dimethylsulfoxide (DMSO) treatment) as well as a structurally similar inactive control (LEI-801). Clear morphological changes were observed in response to all active compounds. Notably, LEI-800 induced elongation of the bacteria and condensation of the DNA, which resembled to some extent the effects observed in bacteria treated with CIP (Fig. 3a). To obtain a more quantitative image analysis, 24 features that describe cell dimensions and fluorescence intensities of individual bacteria, were extracted (Supplementary Data 4).

Principle component analysis (PCA) of these 24 features revealed that the inactive control LEI-801 clustered with DMSO, while the reference antibiotics clustered separately from one another (Fig. 3b,c). LEI-800 formed a cluster by itself, which was interesting considering its apparent similarity to CIP. This led to the suggestion that LEI-800 may have a unique MoA related to the inhibition of DNA synthesis.

### Genomic studies reveal DNA gyrase as target of LEI-800

**Fig. 4:**
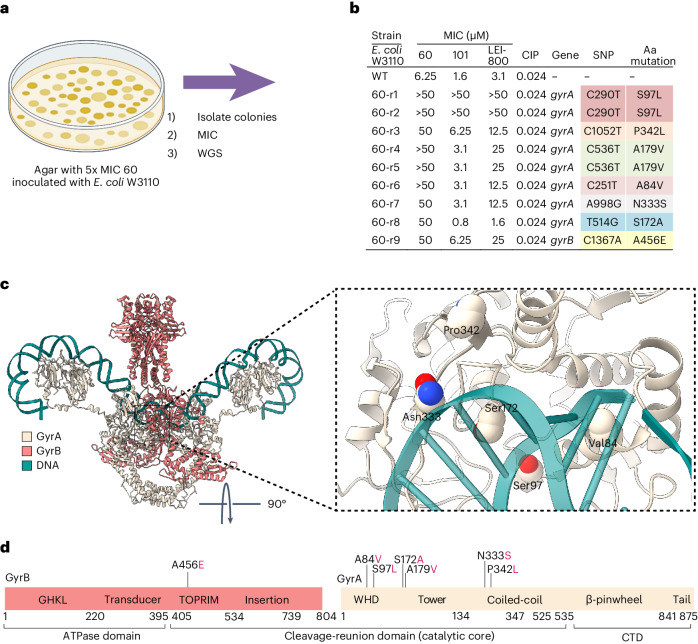
Genome studies reveal DNA gyrase as a target. **a**, Agar containing **60** (5× MIC) was inoculated with 10^7^ c.f.u. of *E.* *coli*. After 1 day, nine viable colonies were isolated and used for MIC testing and WGS. **b**, Susceptibility assays confirm spontaneous resistance to **60** and WGS identified mutations located in the DNA gyrase, predominantly in subunit A. Similar mutations are similarly coloured. Cross-resistance is observed for conformationally restricted compounds **101** and LEI-800. **c**, Localization of the mutations in the core of the DNA gyrase heterotetramer (PDB: 6RKW) outline a possible binding site of isoquinoline sulfonamides. **d**, All mutations are found in the cleavage-reunion domain of DNA gyrase. Domain organization of DNA gyrase, GyrB (coral) and GyrA (beige) subunits.

To identify the specific target of LEI-800, we generated drug-resistant mutants and used comparative genomics to identify single-nucleotide polymorphisms (SNPs) correlated to the resistance phenotype. To this end, *E.* *coli* was grown on agar containing compound **60** (5× MIC) resulting in the identification of nine viable colonies (Fig. 4a,b). All colonies had at least an eightfold increase in MIC for compound **60** and also exhibited resistance to LEI-800 and related compound **101**, but no cross-resistance to other common antibiotics (Supplementary Table 10). Of interest, one of the colonies showing resistance to **60** exhibited an enhanced sensitivity towards LEI-800 and **101**.

WGS of the strains showing resistance to compound **60** revealed that each had mutations in a gene encoding bacterial DNA gyrase (Supplementary Data 5), a heterotetrameric topoisomerase complex comprising two GyrA and two GyrB subunits. Eight of the colonies were found to contain mutations in GyrA, primarily in the N-terminal winged helix (A84V and S97L) or the tower domains (S172A, A179V, N33S and P324L) (Fig. 4c), and one in GyrB (A456E). In addition, a mutation in the gene for nicotinamide adenine dinucleotide phosphate-dependent isocitrate dehydrogenase was found in seven of the nine samples. However, both the base quality score and depth of the reads of this mutation was low (Supplementary Data 5). Furthermore, this mutation (D398E) is assumed to be non-disruptive, both due to the acidic nature of the amino acids and its location, and is therefore unlikely to have major consequences for the protein’s function in carbohydrate metabolism^22,23^. To validate that the mutations in *gyrA* were indeed responsible for the resistant phenotype towards **60**, a clustered regularly interspaced short palindromic repeats–associated protein 9 (CRISPR–Cas9) system was used to introduce the *gyrA* S97L mutation found in mutants 60-r1 and 60-r2 (Fig. 4b) in an *E.* *coli* WT background. In doing so, we confirmed that bacteria containing the S97L mutation were resistant against compounds **60**, **101** and LEI-800 (Supplementary Table 11). Mapping these mutation sites on a recently published structural model of DNA gyrase (Protein Data Bank (PDB): 6RKW)^24^ revealed their spatial proximity and suggested a potential binding pocket (Fig. 4d).

### LEI-800 is a DNA gyrase inhibitor

**Fig. 5:**
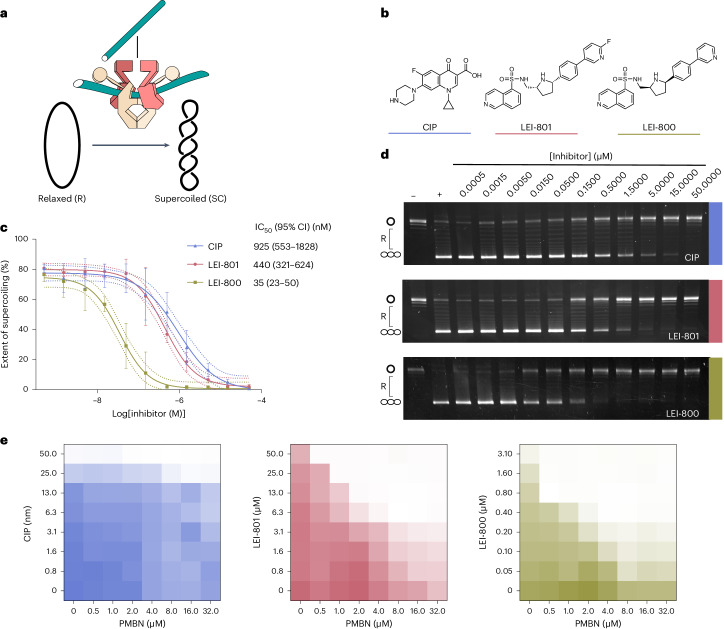
LEI-800 is an exceptionally strong supercoiling inhibitor. **a**, A schematic representation of negative supercoiling. In a supercoiling assay, relaxed (R) DNA is transformed into supercoiled (SC) DNA by DNA gyrase. **b**, Three compounds tested for supercoiling inhibition. CIP (blue) is used as positive control, LEI-801 (coral) is used as antimicrobially inactive compound and LEI-800 (gold) is used as lead compound. **c**, Dose-response curves of DNA gyrase supercoiling inhibition, based on *n* = 3 gels. The mean is shown with error bars and dotted lines representing the standard deviation at each concentration and the 95% confidence interval (CI), respectively. **d**, Representative agarose gels for *E.* *coli* DNA gyrase supercoiling inhibition by the three compounds. **e**, Checkerboard broth microdilution assays show pharmacological disruption of LPS by PMBN does not have an effect on the antimicrobial activity of CIP, yet potentiates both LEI-801 and LEI-800. Source data

DNA gyrase is responsible for introducing negative supercoils in DNA (Fig. 5a), a process that is required for DNA synthesis and proliferation of bacteria. DNA gyrase is a target of the fluoroquinolone antibiotics including CIP. To test whether LEI-800 directly interacts with DNA gyrase, we assessed its ability to inhibit the ATP-dependent supercoiling activity of recombinant *E.* *coli* DNA gyrase in a gel-based assay. The activity of LEI-800 was compared with the inactive control LEI-801 (Fig. 5b,c). CIP was taken along as a positive control and produced an IC_50_ value of 925 nM, in line with previously reported values^25^. Remarkably, LEI-800 inhibited DNA gyrase activity with an IC_50_ of 35 nM, thereby making it >25-fold more potent than CIP. Mirroring the phenotypic screening results, control compound LEI-801 was 13-fold less potent than LEI-800, thereby confirming that the *cis-2R,5R* is the distomer. A reverse ATP-independent supercoiling relaxation reaction was also found to be inhibited by LEI-800 (Supplementary Fig. 5).

### Cellular penetration limits activity of LEI-800

Our biochemical data showed that LEI-800 is a nanomolar range inhibitor of DNA gyrase, but its antimicrobial activity is in the low micromolar range (Extended Data Table 1). We hypothesized that this discrepancy could be due to active efflux by TolC, a major bacterial transporter, or poor penetration of the Gram-negative outer membrane (OM)^26^. To distinguish between these possibilities, we first tested LEI-800 and LEI-801 against a Δ*tolC* efflux pump mutant. No increase in potency was observed, suggesting that the isoquinoline sulfonamides are not substrates of the TolC efflux pump. Next, we tested LEI-800 and LEI-801 on *E.* *coli* strains containing loss-of-function mutations to OM lipopolysaccharide (LPS) assembly genes (Δ*rfaC*, Δ*rfaY*, Δ*rfaI*, Δ*rfaP* and Δ*rfaD*) against which LEI-800 was found to be strongly potentiated (Supplementary Table 12). Of note, the activity of LEI-801 was also potentiated in the Δ*rfaC*, Δ*rfaP* and Δ*rfaD* strains. The activity of LEI-800 and LEI-801 against *E.* *coli* was also enhanced in the presence of polymyxin B nonapeptide (PMBN) (Extended Data Table 1), a well-characterized^27^ OM disrupting agent^28^. The synergistic effect of PMBN and the LEI compounds was further explored with a checkerboard assay. Clear synergy was seen for LEI-800 and LEI-801, while none was observed for CIP (Fig. 5e and Supplementary Table 8). It is noteworthy that the distomeric LEI-801 is a more potent inhibitor of DNA gyrase than CIP in vitro but shows no antibacterial activity against WT *E.* *coli* while LEI-800 does (Extended Data Table 1). This observation, and the findings that LEI-801 is more active against LPS deficient strains and is strongly potentiated by PMBN (Supplementary Table 17), indicate that the compound is less capable of crossing the OM than the more active LEI-800.

### LEI-800 binds a hydrophobic pocket within the GyrA subunit

**Fig. 6:**
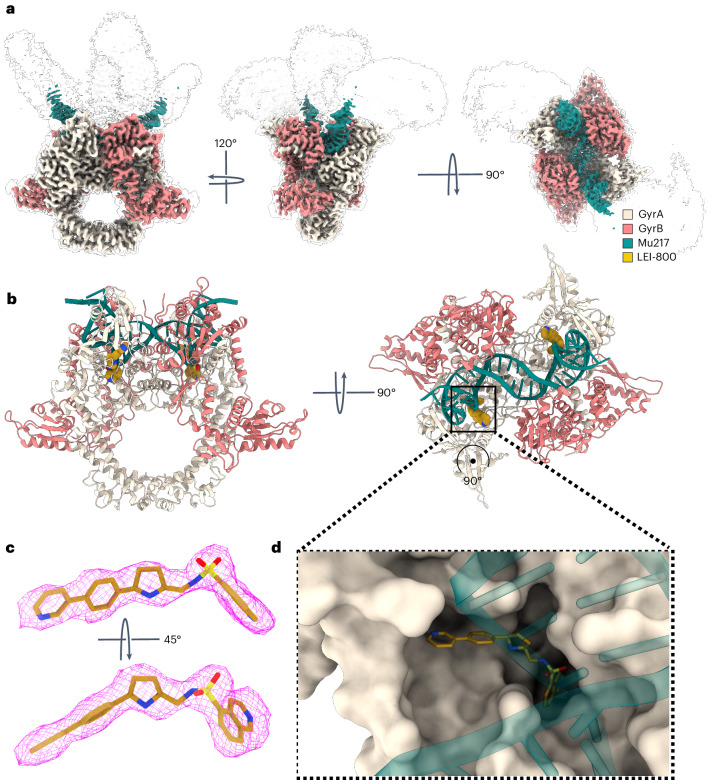
Cryo-EM reveals a unique binding pocket of LEI-800 within DNA gyrase. **a**, An overview of the Gyr–Mu217–LEI-800 cryo-EM map depicted as an overlay of two different contour level maps. Low-resolution contour (white, 5σ) illustrates the position of GyrA CTDs and GyrB ATPase domains. The high-resolution part (12σ) is coloured according to the scheme: coral, GyrB; beige, GyrA; teal, DNA; golden, LEI-800. **b**, An overview of the model of the Gyr–Mu217–LEI-800 complex. GyrA (8–524) and GyrB (402–800) are depicted as beige and coral cartoon representations. The modelled 26-bp central part of Mu217 DNA is shown in teal. Two molecules of LEI-800 observed in a single gyrase heteroteramer are shown as golden van der Waals spheres. A uniform colour scheme is used throughout the manuscript. **c**, A Coulomb potential density map for LEI-800 (contoured at 12σ). **d**, A close-up of the horseshoe-like LEI-800 binding pocket on the DNA-binding surface of GyrA. GyrA is shown as molecular surface, DNA as cartoon representation and LEI-800 as van der Waals spheres.

**Fig. 7:**
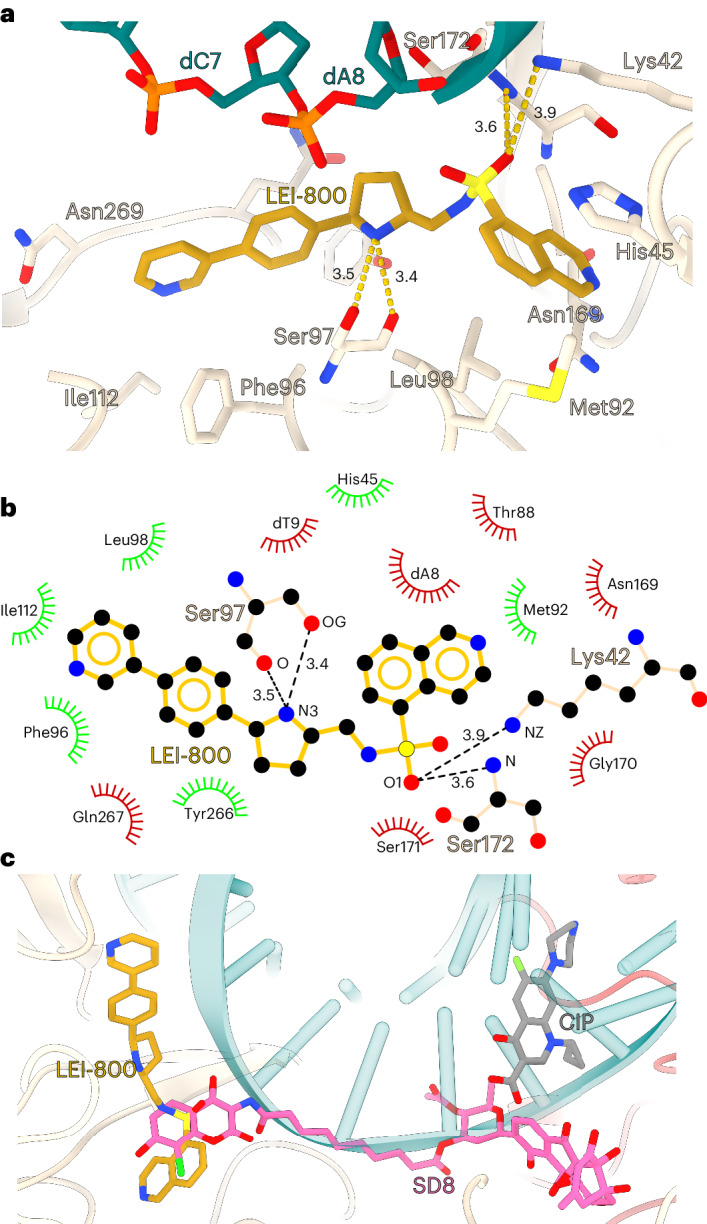
Molecular interactions of LEI-800 explain the mutation data and SAR. **a**, Molecular interactions between GyrA and LEI-800. LEI-800 is shown as stick representation. The main GyrA residues important for LEI-800 binding are labelled. Key hydrogen bonds (between side-chain and main-chain oxygens of Ser97 and N3 nitrogen of central pyrrolidine ring of LEI-800; main chain nitrogen of Ser172, side-chain nitrogen of Lys42 and sulfonic acid of LEI-800 are shown in gold and distances in Angstrom are indicated. **b**, A comparison of LEI-800 (current study), CIP (PDB: 2XCT) and SD8 (PDB: 4CKL) binding sites on the GyrA DNA-binding surface. LEI-800 is shown as golden, SD8 as pink and CIP as grey stick representations. A quinoline ring of LEI-800 shares the binding pocket with the aminocoumarin moiety of SD8, but unlike SD8, LEI-800 does not interfere with DNA binding. **c**, A 2D diagram of the LEI-800 binding site generated by LigPlot^44^. Key hydrogen bonds to Ser97 and Ser172 are shown in black and distances in Å are indicated. Spiked red arcs show non-bonded interactions and green arcs hydrophobic interactions with residues within 3.9 Å distance.

To validate the presumed binding pocket of LEI-800 on the GyrA subunit, we used cryo-EM to determine the high-resolution structure of *E.* *coli* gyrase holocomplex (A_2_B_2_) bound to the substrate 217-bp double-stranded DNA derived from the bacteriophage Mu strong gyrase site (Mu217), nucleotide analogue 5′-adenylyl β,γ-imidodiphosphate (ADPNP) and LEI-800. We visualized the entire enzyme–DNA complex in the wrapped state with a global map resolution of 2.9 Å (Fig. 6a) and built a complete atomic model for the cleavage-reunion domain of the enzyme (residues 8–524 of GyrA and 405–804 of GyrB) at a resolution of 3.1 Å (Fig. 6a). While the overall conformation of the complex is comparable to the published *E.* *coli* gyrase cryo-EM structure in complex with gepotidacin^24^ (PDB: 6RKW), there is a striking difference in the position of the C-terminal domains (CTDs) (Supplementary Fig. 6g). Guided by the mutation analysis (Fig. 4b) and helped by high local resolution around the binding site (2.7 Å, Supplementary Fig. 6f), we readily identified LEI-800 as a region of distinct density, clearly separated from both DNA and protein (Fig. 6c). One molecule of LEI-800 binds to each GyrA subunit, occupying a horseshoe-like hydrophobic pocket on the DNA-binding surface (Fig. 6b,d) just underneath the DNA but far away (>20 Å) from the gyrase catalytic residues. The curved shape of LEI-800 corresponds well to the shape of this pocket, while the opposite would be the case for a distomer LEI-801. The quinoline ring slots between H45 and L98 of GyrA while the pyridine ring is positioned above I112 and F96 (Fig. 7a). Remarkably, four hydrogen bonds anchor LEI-800 in place with the side chain and carbonyl oxygen of GyrA S97 (to the N3 of the central pyrrolidine ring) and the main chain nitrogen of GyrA S172 and side chain of K42 (to the sulfonic acid moiety) (Fig. 7a,b). These findings illuminate how the main mutation (S97L) identified in the LEI-800-resistant mutants prevents binding and also explain the SAR observation that both the nitrogen-containing linker and sulfonamide are of crucial importance. To directly validate these results in vitro, we produced the S97L GyrA variant and, as a control, the S172A variant, and tested the activity of both in a gel-based supercoiling assay. The S97L variant was highly resistant to both LEI-800 and LEI-801 (Supplementary Fig. 8), while LEI-800 and LEI-801 were still active on the S172A (IC_50_ values of 86 and 360 nM, respectively) (Supplementary Fig. 9). These biochemical results support the binding mode observed with the cryo-EM study and are in line with the genetic data.

Intrigued by the apparent selective activity of LEI-800 for *E.* *coli* over other bacteria, we inspected the predicted compound binding pocket in the GyrA proteins from *Acinetobacter baumannii* and *Pseudomonas aeruginosa* as present in the AlphaFold database. Importantly, we found no clear differences in the binding pocket of these bacterial species (Supplementary Fig. 10), therefore we tested LEI-800 against purified gyrase from both organisms. This revealed that LEI-800 effectively inhibited both enzymes (IC_50_ of 103 and 96 nM, respectively) (Supplementary Fig. 11). These findings indicate that the isoquinoline sulfonamides inhibit gyrase from various Gram-negative bacteria, but that their lower activity against these pathogens in MIC assays may result from limited permeability and/or compound efflux.

### LEI-800 blocks gyrase-dependent DNA cleavage

The LEI-800 pocket is not, to the best of our knowledge, exploited by any other natural or synthetic gyrase inhibitors. This presents an exciting opportunity for the development of a novel class of DNA gyrase inhibitors with the potential to overcome fluoroquinolone resistance. The only inhibitor that partially overlaps with the LEI-800 binding site is simocyclinone D8 (SD8), a natural product that prevents DNA binding to GyrA (Fig. 7c). However, in contrast to SD8, LEI-800 binding does not affect interaction with double-stranded DNA. Also, while CIP binding promotes double-strand cleavage by the enzyme, upon LEI-800 binding the DNA was found to remain fully intact. During gyrase-mediated DNA cleavage, a metal ion is proposed to move between the two metal-binding sites, ‘A’ and ‘B’, corresponding to the pre-cleavage and post-cleavage states, respectively^29^. Inspection of metal binding pocket in Gyr–Mu217–LEI-800 revealed a clear density consistent with a single metal ion (assumed to be Mg^2+^ based on the buffer used) bound in the ‘A’ configuration (Supplementary Fig. 12a). These findings indicate that LEI-800 probably locks the gyrase–DNA complex in a pre-cleavage state. In line with this hypothesis, time-course cleavage assays (Supplementary Fig. 12c,e) showed clear inhibition of CIP-mediated DNA cleavage by LEI-800. To further demonstrate that LEI-800 inhibits native DNA cleavage by the gyrase, we investigated the effect of replacing Mg^2+^ in the assay buffer with Ca^2+^, which is known to increase the levels of intrinsic cleavage by the enzyme^30^. Again, DNA cleavage was strongly inhibited in the presence of LEI-800 (Supplementary Fig. 12d,e). Taken together, these findings reveal LEI-800 to be the first reported gyrase inhibitor that inhibits DNA cleavage, rather than promoting it, and thereby blocks both supercoiling and relaxation activities of the enzyme.

### LEI-800 is active against fluoroquinolone-resistant clinical isolates

With structural evidence supporting a unique binding and activity mode for LEI-800, we hypothesized that it would be active against fluoroquinolone-resistant strains. We first queried cross-resistance between LEI-800 and CIP by testing strains with point mutations that confer resistance against one of the drugs. CIP was still fully active against all isoquinoline resistant strains generated (Fig. 4b). At the same time, introduction of single mutations in GyrA known to confer fluoroquinolone resistance (specifically S83L^31,32^ and D87N^31^) resulted in at least 60-fold increase in the MIC of CIP, but did not affect the susceptibility to LEI-800 (Supplementary Table 11). In addition, LEI-800 was also found to be active against nine highly fluoroquinolone-resistant clinical isolates (Extended Data Table 2). As with the other strains tested, the addition of PMBN resulted in strong potentiation of the antibacterial activity of LEI-800 against CIP resistant isolates.

Finally, we assessed the in vitro antibacterial efficacy of the combination of LEI-800 and CIP. While no synergy was observed between the antibiotics in a susceptibility assay (Supplementary Fig. 4), no resistant *E.* *coli* colonies were isolated when a combination of the LEI-800 and CIP (each at 5× MIC) was used, corresponding to a frequency of resistance (FoR) of <1 × 10^−8^. In contrast, resistance was observed when the bacteria were tested against each compound alone, with a FoR for CIP of 1 × 10^−6^ and LEI-800 5 × 10^−7^ (Supplementary Table 13). Collectively, these results suggest that a fluoroquinolone combined with LEI-800 may limit the emergence of resistance in *E.* *coli*.

## Discussion

For decades, the bacterial DNA gyrase has proven to be an excellent target for antibiotics with a number of clinically used anti-infectives operating by inhibiting its activity^33^. Given that DNA gyrase lacks a direct human homologue, has multiple target sites and is essential to bacterial DNA replication and transcription, inhibitors have the potential to selectively target bacterial cells versus host cells. Notably, the negative supercoiling activity of DNA gyrase is a multi-step process that can be interrupted at multiple stages through pharmacological intervention^34^. Key examples are the classical fluoroquinolone antibiotics^35^ and the more recent novel bacterial topoisomerase inhibitors^36^ and albicidin^37^ that intercalate in DNA and stabilize the complex after DNA cleavage, the aminocoumarins^38^ that compete with ATP, and SD8^39^ that prevents DNA binding with the gyrase complex. Despite successful clinical application, resistance to fluoroquinolones is increasingly abundant^40,41^ and the mammalian toxicity of fluoroquinolones^42^, novel bacterial topoisomerase inhibitors^43^ and aminocoumarins present serious clinical concerns. Therefore, there is a clear need and opportunity for the development of improved next-generation DNA gyrase inhibitors.

Here, we disclose an entirely novel class of DNA gyrase inhibitors termed the isoquinoline sulfonamides. Initially found through phenotypic screening of an existing compound set, extensive SAR studies led to the identification of key isoquinoline sulfonamide pharmacophores required for in vitro activity against *E.* *coli* and *K.* *pneumoniae*, after which conformational restriction of the initial scaffold led to the lead compound LEI-800. LEI-800 exhibits potent, Gram-negative-specific antibacterial activity with little mammalian cell toxicity. Cell morphology and mutation selection clearly pointed to DNA gyrase as the target for LEI-800, after which cryo-EM studies revealed that the compound binds to a previously unexploited site on the GyrA subunit. The specific antibacterial activity of LEI-800 against *E.* *coli* and *K.* *pneumoniae* but not towards Gram-positive bacterial pathogens may be explained by the lower homology of the *E.* *coli* DNA gyrase with other bacteria (Supplementary Table 14), particularly with the Leu98 → Tyr alteration in the Gram-positive GyrA subunit (Supplementary Fig. 10)

When directly compared with the clinically used fluoroquinolone CIP, LEI-800 was found to exhibit a number of key differences: (1) morphological studies with BCP indicated that *E.* *coli* cells respond differently to CIP treatment than to LEI-800, (2) limited cross-resistance was found between CIP and LEI-800 in strains harbouring resistance-inducing point mutations, further highlighted by the susceptibility of CIP resistant clinical isolates to LEI-800 and (3) mechanistic insights derived from cryo-EM studies reveal no overlap in the binding site of LEI-800 in the DNA gyrase compared with that of CIP or any other gyrase inhibitor.

It is worth noting that our structure, to the best of our knowledge, represents the first full-length *E.* *coli* gyrase structure in complex with an intact DNA molecule. This structural insight carries implications for our understanding of the proposed DNA cleavage mechanism. Notably, the observed metal ion position aligns with the so-called A site position, previously documented in multiple crystal structures where the catalytic tyrosine was replaced by phenylalanine^29^. Our structure also contrasts another recently published cryo-EM structure of the *E.* *coli* gyrase complexed with albicidin, where cleaved DNA and a metal bound in the ‘B site’ was observed^37^. Thus, our native solution-based structure supports the previously postulated cleavage mechanism^29^.

On the basis of our structural findings, we propose that LEI-800 exerts its function by impeding the cleavage activity of the enzyme. To validate this hypothesis, we conducted DNA cleavage assays that unequivocally demonstrate that LEI-800 inhibits cleavage induced by both CIP and Ca^2+^ ions. We suggest that by specifically interacting with S97, LEI-800 locks the catalytic loop (harbouring the Y122–R121 pair) in place and thereby impairs the attack of Y122 on the scissile phosphate bond thus inhibiting cleavage–religation cycle of the enzyme. Taken together, this evidence underscores LEI-800 as the pioneering representative of an entirely novel class of topoisomerase inhibitors.

While CIP was found to generally exhibit lower MIC values than LEI-800 against susceptible strains, it is interesting to note that LEI-800 is a much more potent inhibitor of DNA gyrase supercoiling activity, an effect most probably ascribed to the Gram-negative OM. Indeed, synergy assays with PMBN indicate that once past the OM, the antibacterial activity of LEI-800 is substantially enhanced. This points to the potential for developing analogues of LEI-800 with improved OM permeability. While activity of LEI-800 is specific to Gram-negative bacteria, the structural insights obtained provide a blueprint for the design of next-generation gyrase inhibitors. Notably, a combination of CIP and LEI-800 was found to limit the emergence of resistance, which may hold promise for the future clinical application of such compounds. In summary, LEI-800 belongs to a novel class of antibacterial agents with potent activity against Gram-negative bacteria, including clinical isolates, and binds to a unique pocket of DNA gyrase that is not exploited by other inhibitors of this key antibiotic target.

## Methods

### Reagents and materials

Buffers and salts were of American Chemical Society reagent grade or higher and were purchased commercially, from Carl Roth GmbH and Sigma-Aldrich, biological materials and growth media were purchased from Sigma-Aldrich, Scharlab S.L. and Fischer Scientific. Antibiotics trimethoprim (Sigma-Aldrich), ceftazidime (ceftazidime pentahydrate, Thermo Scientific) and kanamycin (kanamycin monosulfate, MP biomedicals) were dissolved in DMSO stored in −20 °C, apart from CIP, which was used from a 3 M aqueous solution containing 0.01% AcOH. All test compounds were used from 10 mM DMSO stock solutions made from the freeze dried powder and stored at −20 °C.

### Bacterial strains

*K.* *pneumoniae* American Type Culture Collection (ATCC) 29665 (National Collection of Type Cultures (NCTC) 11228), *E.* *coli* ATCC 25922, *P.* *aeruginosa* ATCC 27853, *A.* *baumannii* ATCC BAA747 and *Staphylococcus aureus* USA300 (ATCC BAA1717) belong to the ATCC. *E.* *coli* NCTC 13463 and 13846 belong to the NTCT (UK Health Security Agency). *E.* *coli* JW5503, JW3600, JW3602, JW3605, JW3594 and JW3596 belong to the Keio Collection^45^ of single-gene knockouts. *E.* *coli* strains 552059.1 and 552060.1 were isolated from urine^46^ and acquired from the clinical Medical Microbiology department at the University Medical Center Utrecht. *E.* *coli* mcr-1 (EQAS 2016 412016126, mcr-1 positive, recovered during international antimicrobial resistance programmes), W3110 and BW25113, belong to the laboratory collection of N.I.M. Fluoroquinolone-resistant *E.* *coli* strains 965, 991, 1022, 1075, 1104, 1146, 1175, 1192, 1201 and 1233 were isolated from blood cultures, positive CIP resistance during July–August 2021 and were acquired from the Department of Medical Microbiology and Infection Prevention, Amsterdam University Medical Centre. The following reagents were obtained through Biodefense and Emerging Infections Resources, National Institute of Allergy and Infectious Diseases, National Institutes of Health: *E.* *coli*, strain MVAST0072, NR-51488.

### Library screen

From glycerol stocks, *E.* *coli* W3110 was cultured on blood agar plates (PB5039A, Thermo Scientific) by overnight (18 ± 2 h) aerobic incubation at 37 °C. A single colony was transferred to tryptic soy broth (TSB, 02-200-500, Scharlab). The cultures were grown to exponential phase (OD_600_ of 0.5) at 37 °C. The bacterial suspensions were diluted 200-fold in cation-adjusted Mueller–Hinton broth (CAMHB) and 99 µl was added to a library of test compounds (1 µl 10 mM DMSO stock, per well in technical duplicates) in polypropylene 96-well microtitre plates to reach a volume of 100 µl and a final concentration of 100 µM for each test compound and a maximum of 1% DMSO. The plates were sealed with breathable membranes and incubated at 37 °C for 18 h with constant shaking (600 r.p.m.). Screening hits were selected from the wells where no visible bacterial growth was observed, as compared with the inoculum controls containing 1% DMSO. The follow-up screen of H-89 derivatives was performed under similar conditions with a final concentration of 50 µM.

### Antibacterial activity screen

From glycerol stocks, *S.* *aureus* USA300, as the Gram-positive representative strain, and *E.* *coli* W3110, as the Gram-negative representative strain, were cultured on blood agar plates by overnight (18 ± 2 h) aerobic incubation at 37 °C. A single colony was transferred to TSB or lysogeny broth (LB). Cultures were grown to exponential phase (OD_600_ of 0.5) aerobically at 37 °C. The bacterial suspensions were diluted 200-fold in cation-adjusted CAMHB and 99 µl was added in a library of test compounds (1 µl DMSO stock solution per well in technical duplicates) in polypropylene 96-well microtitre plates to reach a volume of 100 µl and a final concentration of 100 µM for each test compound and a maximum of 1% DMSO. The plates were sealed with breathable membranes and incubated at 37 °C overnight with constant shaking (600 r.p.m.). Screening hits were selected from the wells where no visible bacterial growth was observed as compared with the inoculum controls containing 1% DMSO.

### MIC

MIC was determined by broth microdilution. Single-colony cultures were grown to exponential phase (OD_600_ of 0.5) aerobically at 37 °C. In case of strains from the Keio collection^45^, 50 µg ml^−1^ kanamycin was supplemented to the media to ascertain a homogeneous population. The bacterial suspensions were diluted 100-fold in CAMHB and 50 µl was added to a twofold serial dilution series of test compounds (50 µl per well) in polypropylene 96-well microtitre plates to reach a volume of 100 µl. The plates were sealed with breathable membranes and incubated overnight at 37 °C with constant shaking (600 r.p.m.). The MIC was determined as the lowest concentration at which no visible bacterial growth was observed as compared with the inoculum controls from the median of a minimum of triplicates.

### Cytotoxicity assay (MTT)

HepG2 (ATCC HB-8065) and HEK293T (ATCC CRL-3216) cell lines were cultured at 37 °C and 7% CO_2_ in Dulbecco’s modified Eagle medium (Sigma-Aldrich, D6546) with GlutaMax, penicillin (100 µg ml^−1^), streptomycin (100 µg ml^−1^) and 10% fetal calf serum. Cells were passaged twice a week by first detaching using 0.05% trypsin in phosphate-buffered saline (PBS) and then diluting to appropriate confluence. Compound cytotoxicity was evaluated using the standard (3-(4,5-dimethylthiazol-2-yl)-2,5-diphenyltetrazolium bromide (MTT) assay protocol (MTT assay protocol for cell viability and proliferation, Merck) with slight changes. HepG2 and HEK293T cells were seeded at a density of 1.5 × 10^4^ cells per well in a clear 96-well tissue culture treated plate in a final volume of 100 µl in Dulbecco’s modified Eagle medium, supplemented with fetal bovine serum (1%), GlutaMax and penicillin/streptomycin. Cells were incubated for 24 h at 37 °C, 7% CO_2_ to allow cells to attach to the plates. In addition to a single vehicle control, compounds (diluted from DMSO stock) were added into each well at eight concentrations ranging from 100 µM to 0.046 µM in threefold dilutions (final DMSO concentration 0.5%). Incubation was done for 24 h at 37 °C, 7% CO_2_. After the incubation, MTT was added to each well at a final concentration of 0.40 mg ml^−1^. The plates were then incubated for 2 h at 37 °C, 7% CO_2_. Medium was carefully removed via suction, and purple formazan crystals were resuspended in 100 µl DMSO. Absorbance was read at 570 nm using a Clariostar plate reader with MARS (V4.20). The data were then analysed with GraphPad Prism software. IC_50_ values were calculated using non-linear fitted curve with variable slope settings, with values adjusted for background (plotted absorbance (ABS)_sample_ = (ABS_sample_ – ABS_background_) / (ABS_vehicle_ – ABS_background_)). Technical triplicates for each condition were used, along with biological duplicates.

### Time-kill assay

From glycerol stocks, bacterial strains were cultured on blood agar plates by overnight incubation at 37 °C. Subsequently, a single colony was cultured in TSB overnight at 37 °C. The culture was diluted 100-fold in fresh CAMHB and grown until early exponential phase (OD_600_ of 0.25) followed by 100-fold dilution in media. The culture was split in separate culture tubes containing 2 ml. Test compounds were added to the cultures at concentrations of 3.1 µM and 6.2 µM (corresponding to 4× and 8× MIC, respectively) and incubated at 37 °C for a total of 24 h. At indicated timepoints (*t*: 0, 1/2, 1, 2, 4, 8 and 24 h) 100 µl of each culture was centrifuged for 5 min (9,800*g*). The supernatant was discarded and pellets were washed once with filter-sterilized PBS, then resuspended in an equal volume of fresh buffer and samples were tenfold serially diluted until a 10^5^ dilution. Then, 10 µl of the appropriate dilutions were inoculated on LB agar plates in technical duplicates, subsequently allowed to evaporate and incubated at 37 °C for 18 h. The colonies were counted and used to calculate the colony forming unit (c.f.u.) per ml remaining in the original culture by taking the dilution factors into account. The experiment was performed in biological duplicates.

### Haemolytic activity

Whole defibrinated sheep blood (10631715, Fisher Scientific) was centrifuged (400*g*) for 15 min at 4 °C. The supernatant was discarded and the remaining blood cell suspension was mixed with PBS and centrifuged (400*g*) for 15 min at 4 °C. Washing cycles were repeated at least three times, until the supernatant was clear after centrifugation. The packed blood cells were diluted 25-fold in PBS with 0.002% polysorbate 80. Test compounds were serially diluted twofold in U-bottom polypropylene 96-well microtitre plates in PBS with 0.002% polysorbate 80 (75 µl). An equal volume (75 µl) of the blood cell suspension was added to all wells. Final concentrations of antibiotics ranged from 1.6 µM to 50 µM in triplicate. The well plates were incubated for 20 h at 37 °C with continuous shaking (500 r.p.m.). After incubation, plates were centrifuged (800*g*) for 5 min, and 25 µl of supernatant was transferred to a clear UV-star flat-bottom polystyrene 96-well plate containing 100 µl ultrapure water per well. Absorption was measured at 415 nm with a Spark multimode microplate reader (Tecan). Data were corrected for the background response of 0.5% DMSO in the presence of cells with no antibiotic and normalized using the absorbance of 0.1% Triton X-100 with blood cells, as 100% haemolytic activity.

### BCP

Overnight *E.* *coli* BW25113 cultures were diluted 1:100 in fresh LB medium in a 100 ml Erlenmeyer flask and incubated at 120 r.p.m. and 37 °C. When OD_600_ >0.2, the cultures were diluted to OD_600_ of 0.1 in fresh LB medium with a final volume of 200 µl in a 2 ml Eppendorf tube. The appropriate antibiotic or control treatment was applied and the samples were incubated at 200 r.p.m. and 30 °C for 2 h. Subsequently, 4,6-diamidino-2-phenylindole (DAPI) (2 µg ml^−1^), FM4-64 (2 µg ml^−1^) and SYTOX Green (0.5 µM) were added to reach the desired concentration and the samples were incubated a further 10 min. The samples were then centrifuged at 3,300*g* for 50 s and the pellets resuspended in half the original volume. From this suspension, 5 µl was spotted onto a 2.0% agarose pad for microscopy.

Microscopy was performed on a Zeiss Axio Observer Z1/7 inverted microscope. For differential interference contrast (DIC), the transmitted light source was an Aquilla TL halogen lamp (3.0 V). For wide-field fluorescence, the Zeiss Colibri.2 light-emitting diode lamps were configured as follows: DAPI (365 nm, 150 ms, 25.42%), FM4-64 (590 nm, 1,000 ms, 100%) and SYTOX Green (470 nm, 150 ms, 25.42%). Illuminated and reflected light were led through the following filters: DAPI (Zeiss 49: excitation of G 365, beam splitter FT 395, emission BP 445/50), FM4-64 (Zeiss 63: excitation BP 572/25 (HE), beam splitter FT 590 (HE), emission BP 629/62 (HE)) and SYTOX Green (Zeiss 38: excitation BP 470/40 (HE), beam splitter FT 495 (HE), emission BP 525/50 (HE)). Images were collected through a Plan-Apochromat 100×/1.40 objective in oil immersion (*n* = 1.518) with a Hamamatsu C9100-02 camera.

Specific information on the programs and parameters used during image analysis are displayed in Supplementary Table 9. For the qualitative assessment of the bacterial morphology, the acquired images were first pre-processed in ImageJ (v1.53m)^47^. Thereafter the brightness and contrast were adjusted to allow the clearest visual inspection of the particular phenotype. For the quantitative BCP, DIC images were first pre-processed and then a random forest classifier was trained with Illastik (v1.3.3)^48^ to generate a segmented image. These binary masks were then combined together with the original DIC image and processed with the ImageJ plugin MicrobeJ (v 5.13l)^49^. MicrobeJ generated cell outlines and performed measurements on cell shape accordingly. The recorded cell outlines produced by MicrobeJ were exported and used as a guide to characterize the DNA shape within each bacterium using a custom-made ImageJ macro (Appendix II). DAPI and SYTOX Green intensity were measured on raw images in a similar manner. Then, by hand, polygons were drawn that excluded bacteria to determine background intensity. After background correction the intensity was standardized to the DMSO control within the same biological replicate. For PCA, the BCP assay was executed for three independently biological replicates. During each experiment, images were acquired until 24 or more bacteria in total had been observed. Following feature extraction for each replicate, principal component analysis was performed using GraphPad Prism (v9.0.0) using multiple variable analysis with standardized data. Principal components (PCs) were selected based on the per cent of total explained variance (minimum 80%).

### Resistant mutant generation

Spontaneous resistant colonies were obtained by plating 100 µl of *E.* *coli* W3110 inoculum grown to an OD_600_ of 0.5 (~10^7^ c.f.u.) onto LB agar plates containing 5× the MIC of compound **60**. The plates were incubated at 37 °C and checked for growth after 24 and 48 h. Single colonies were picked after 48 h of incubation and their MIC was determined as described previously. Nine colonies with an increased MIC were selected for further analysis and stored as glycerol stocks.

### WGS

Nine spontaneous **60**-resistant mutants of *E.* *coli* were selected for genome sequencing. DNA was extracted as described elsewhere^50^. Briefly, *E.* *coli* cells were collected from an overnight culture and resuspended in lysis buffer (Tris-EDTA, sodium dodecyl sulfate (SDS) 10% and proteinase K), incubated for 1 h at 37 °C. Classical extraction with phenol–chloroform was performed and the aqueous layer was precipitated with absolute ethanol. The DNA pellet was washed with 70% ethanol, dried and solubilized in TE to perform RNA digestion with RNase 50 µg ml^−1^ (RNase A, Thermo Fisher). Degraded RNA was removed by phenol–chloroform extraction followed by ethanol precipitation. DNA was resuspended in nuclease-free water. Genome sequencing was performed using Illumina Novaseq 6000 PE150 at Novogene Co. Ltd. Paired-end sequence reads were generated and mapped against the reference genome of *E.* *coli* W3110. The alignment to the reference genome was performed using the Burrow–Wheeler alignment tool (v0.7.8 (ref. ^51^)). SNP–insertion/deletion calling, annotation and statistics were performed using SAMtools^52^ (v0.1.19) and ANNOVAR^53^ (v2015MAR22). The structural variant calling annotation and statistics was performed with BreakDancer v1.4.4 and ANNOVAR^53^ (v2015MAR22). The WT *E.* *coli* W3110 was also sequenced and compared with the reference genome to confirm that the mutations found in the **60**-resistant colonies were unique and related to the antibiotic resistance.

### FoR

*E.* *coli* W3110 from an overnight culture (1.4 × 10^9^ c.f.u. ml^−1^) were washed in PBS and subsequently inoculated onto LB agar plates containing 5× MIC and 10× MIC antibiotic (LEI-800 or CIP) or the combination (5× MIC of LEI-800 + CIP) with 50 µl per plate, onto three plates per condition, at a density of 7 × 10^7^ c.f.u. per plate. The titre was determined by removing 100 µl of culture, serially diluted and plated (50 µl per plate, onto three plates per dilution). The plates were incubated at 37 °C and examined for colonies every 12 h for a total of 48 h. The number of colonies on each plate was counted. Antibiotic-selected colonies were randomly picked (three per plate) and tested for confirmation of resistance. Mutation frequency was calculated by dividing the number of confirmed resistant mutants obtained by the total bacteria plated.

### Construction of *gyrA* recombinant mutants in *E.coli* W3110

Mutants of the DNA gyrase subunit A encoding gene (*gyrA*) were constructed following the protocol for gene editing via the CRISPR–Cas9 system^54^. Strains and plasmids used are listed in Supplementary Table 15, and primers used can be found in Supplementary Table 16. The following mutations were created: S97L in *E.* *coli* W3110. Two common mutations in the *gyrA* gene that give resistance to fluoroquinolone antibiotics were created in *E.* *coli* W3110, namely S83L and D87N. To create the *gyrA* (S97L) mutants, the construct psgRNA-gyrA-M5 was assembled as follows: The 20-nt spacer sequence was introduced by PCR on pTargetF using primers gyrA_P11 and gyrA_P12, the homology-directed repair (HDR) arms were amplified from *E.* *coli* W3110 genomic DNA by PCR using primer pairs gyrA_P05 and gyrA_P013, and gyrA_P08 and gyrA_P14. The three PCR fragments generated were cloned into the SpeI and BglII digested pTargetF via Gibson assembly. To avoid off-target effects of CRISPR, besides desired point mutation for *gyrA* (S97L), four other silent point mutations were also included in the HDR template. Consequently, the amino acid sequence of *gyrA* will remain the same in the generated mutant except for the desired mutation. Similarly, constructs psgRNA-gyrA-S83L and psgRNA-gyrA-D87N were created to introduce mutations S83L and D87N in *gyrA*, respectively. In psgRNA-gyrA-S83L, spacer sequence was introduced using primers gyrA_P12 and gyrA_P17, HDR template was amplified using primer pairs gyrA_P05 and gyrA_P18, and gyrA_P08 and gyrA_P19. In psgRNA-gyrA-D87N, spacer sequence was introduced using primers gyrA_P12 and gyrA_P20, HDR template was amplified using primer pairs gyrA_P05 and gyrA_P21, and gyrA_P08 and gyrA_P22. The verified constructs for *gyrA* engineering were transformed into *E.* *coli* W3110 carrying pCas9, and plated in LB containing kanamycin (50 µg ml^−1^) for the selection of the pCas9 plasmid and spectinomycin (100 µg ml^−1^) for the selection of psgRNA-gyrA-X. The plates were incubated at 30 °C. Plasmids were cured by growing the strains with 0.5 mM isopropylthiogalactoside with no antibiotics to lose first the psgRNA-gyrA plasmids. After losing the spectinomycin resistance, the strain was grown in LB with no antibiotics at 42 °C to lose the pCas plasmid. Colony PCR was performed on plasmid-free colonies using primers gyrA_P9 and gyrA_P10, and PCR products were sequenced to confirm the desired mutation

### Topoisomerase assays

*E.* *coli* gyrase supercoiling inhibition assays for both mutant and WT enzymes were executed by setting up a master mix including 0.5 µg relaxed plasmid (pBR322) in 35 mM Tris–HCl (pH 7.5), 24 mM KCl, 4 mM MgCl_2_, 2 mM dithiothreitol (DTT), 1.8 mM spermidine, 1 mM ATP, 6.5% (wt/vol) glycerol and 0.1 mg ml^−1^ albumin per reaction. Of this stock solution, 27 µl was aliquoted for each reaction in 1.5 ml Eppendorf tubes and supplemented with 0.6 µl of the appropriate compound dilution or corresponding solvent with a final DMSO concentration of 2%. The reactions were started with 3 µl of enzyme (1 U for WT gyrase retrieved from Inspiralis Limited or 25–30 nM gyrase variants purified in house) in dilution buffer (50 mM Tris–HCl (pH 7.5), 100 mM KCl, 2 mM DTT, 1 mM EDTA and 50% (wt/vol) glycerol) for a final volume of 30 µl. Reactions were run at 37 °C for 30 min and stopped with the addition of 30 µl STEB buffer (40% (wt/vol) sucrose, 100 mM Tris–HCl pH 8, 100 mM EDTA, 0.5 mg ml^−1^ bromophenol blue) and 30 µl of a chloroform:isoamyl alcohol solution (24:1 vol/vol). After brief vortexing and centrifugation at 2,300*g* for 1 min, 20 µl of the aqueous phase was loaded onto 1% (wt/vol) agarose gels in TAE (Tris–acetate 0.04 mM and EDTA 0.002 mM) gel. The gels were run at 85 V for 2 h followed by staining (15 min) in 1 µg ml^−1^ ethidium bromide and destaining (5–10 min) in water. DNA was visualized (602/50, UV Trans, auto optimal exposure) with a ChemiDoc MP (Bio-Rad Laboratories Inc., equipped with ImageLab Touch Software v2.0.0.27) and the percentage of supercoiled DNA relative to the total amount material per lane was determined with ImageLab 6.1 software (Bio-Rad Laboratories Inc.) or ImageJ^47^ software. IC_50_ curves were generated in GraphPad Prism (v9.0.0) using the non-linear regression curve fit variable slope with four parameters and least squares regression. *A.* *baumannii* and *P.* *aeruginosa* gyrase supercoiling inhibition assays were set up in the same way as *E.* *coli* gyrase supercoiling assays. The enzyme was retrieved from Inspiralis Ltd. (PAG1001 and ABG1001) and tested in-house for activity with ×5 U enzyme used for each reaction.

*E.* *coli* relaxation inhibition assays were conducted in a similar manner to supercoiling inhibition assays with the following modifications: spermidine and ATP were omitted from the assay buffer, supercoiled plasmid (pBR322) was used as DNA substrate and 200 nM *E.* *coli* gyrase was added to each reaction. After stopping each reaction with STEB, 3 µl 2% SDS was added and tubes were vigorously vortexed before loading the aqueous phase onto the gel.

Time courses of CIP- and Ca^2+^-mediated DNA cleavage were performed based on a previously published protocol^55^. *E.* *coli* gyrase (200 nM) was incubated at 25 °C in 360 µl reactions with assay buffer (35 mM Tris–HCl (pH 7.5), 24 mM KCl, 4 mM MgCl_2_, 2 mM DTT, 1.8 mM spermidine, 1 mM ATP, 6.5% (wt/vol) glycerol and 0.1 mg ml^−1^ albumin) and 500 ng supercoiled pBR322 DNA (Inspiralis Ltd.). For CIP-induced cleavage, 5 µM was added into the master mix. For Ca^2+^-induced cleavage, the assay buffer was supplemented with 5 mM CaCl_2_. In both assays involving induced cleavage, 5 µM LEI-800 was added before either CIP or CaCl_2_. At selected timepoints, 30 µl aliquots were withdrawn and stopped by an addition of 3 µl 2% SDS and 3 µl 100 mM EDTA. After the time course, aliquots were treated with proteinase K (0.5 mg ml^−1^) for 30 min at 37 °C and extracted with 30 µ chloroform:isoamyl alcohol solution (24:1 vol/vol). The aqueous layers from the assays were mixed with 30 µl STEB and run on 1% agarose TAE gels with 10 µg ml^−1^ ethidium bromide at 80 V. Gels were visualized and analysed as described for the supercoiling inhibition assays.

### Site-directed mutagenesis of *gyrA*

Plasmid pET28-GyrATS encoding WT *gyrA* was a gift from Prof. Valerie Lamour (Institute of Genetics and of Molecular and Cellular Biology, University of Strasbourg). Point mutations were made using Gibson assembly as previously described^56^.Briefly, forward or reverse primers from the ColE1 site of *E.* *coli* were used in two separate PCR reactions with forward or reverse primers from the adjacent side of the plasmid at the point of mutagenesis to create two plasmid fragments. The PCR products were cleaned up with a DNA cleanup micro kit (Thermo) then ligated using NEBuilder Hifi DNA Assembly Master Mix. Primers were synthesized by Merck and mutations were verified by whole plasmid sequencing (Plasmidsaurus). GyrA^S97L^ and GyrA^S172A^ variants were purified as previously described^37^.

### Checkerboard assay

Dilution series of both the test compound and potential synergist (PMBN or CIP) were prepared in CAMHB medium. To evaluate synergy, 25 µl of test compound solutions were added to wells containing 25 µl of potential synergist solution. This was replicated in three columns for each combination so as to obtain triplicates. Then, 50 µl of bacterial stock (0.5 × 10^5^ c.f.u. ml^−1^) was added and the plates were sealed with breathable membranes. After incubation for 20 h at 37 °C while shaking at 600 r.p.m., the breathable seals were removed and the plates shaken using a bench top shaker to ensure homogeneous bacterial suspensions. The plates were then transferred to a Tecan Spark plate reader and following another brief shaking (20 s) the OD_600_ was measured. The resulting OD_600_ values were transformed to a two-dimensional (2D) gradient to visualize the growth/no-growth results. The fractional inhibitory concentration index (FICI) was calculated using the following formula with FICI ≤0.5 (ref. ^57^) indicating synergy: FICI = (MSC_ant_) / MIC_ant_ + (MSC_syn_) / MIC_syn_, where MSC_ant_ is the MIC of antibiotic in combination with synergist, MIC_ant_ is the MIC of antibiotic alone, MSC_syn_ is the MIC of synergist in combination with antibiotic and MIC_syn_ is the MIC of synergist alone. In cases where the MIC of the antibiotic or synergist was found to exceed the highest concentration tested, the highest concentration in the dilution series was used to determine the FICI and the result reported as ≤ the calculated value.

### Cryo-EM sample preparation

Plasmids for the expression of *E.* *coli* GyrA and GyrB (pET28-GyrATS and pET28-GyrBTS) were previously described^24^ and obtained as a gift of Prof. Valerie Lamour (IGBMC, University of Strasbourg). GyrA and GyrB were purified as described^24^. As a substrate, 217-bp DNA (a strong gyrase binding site of phage Mu) was used^37^. *E.* *coli* GyrA and GyrB subunits were mixed in equimolar proportions on ice for 1 h to reconstitute the full DNA gyrase enzyme that was additionally purified by gel filtration (Sephadex 200 increase 10/300, Cytiva) in the following buffer: Na–HEPES 20 mM pH 8, tris(2-carboxyethyl)phosphine 0.5 mM, EDTA 1 mM, KCl 150 mM and glycerol 10%. Purified gyrase complex was concentrated to ~16 mg ml^−1^ and stored at −80 °C. Then, 217-bp DNA was added to the complex in a 1:1 molar ratio and the complex was buffer exchanged using dialysis at 4 °C overnight to cryo-EM buffer (25 mM Na–HEPES pH 8, 30 mM potassium acetate, 2.5 mM magnesium acetate and 0.5 mM tris(2-carboxyethyl)phosphine). After buffer exchange, the sample was concentrated to ~20 µM. The sample was supplemented with 100 µM LEI-800 compound, 1 mM ADPNP and incubated for 30 min at 37 °C. Next, 8 mM CHAPSO was added and the sample was centrifuged (60 min at 21,000*g*) to remove potential aggregates.

### Cryo-EM data collection and analysis

Aliquots of 4 μl of reconstituted complexes were applied to glow-discharged (Leica, 60 s 8 mA^−1^) Quantifoil holey carbon grids (R2/1, 300 copper mesh). After 30 s of incubation with 95% chamber humidity at 10 °C, the grids were blotted for 3.5 s and plunge-frozen in liquid ethane using a Vitrobot mark IV (FEI). Cryo-EM data were collected at Astbury Biostructure Laboratory Cryo-EM facility (University of Leeds, UK) on a Krios G2 microscope (Thermo Fisher Scientific) operated at 300 kV and nominal magnification of 120 kx. Movie frames were collected at the calibrated physical pixel size of 0.68 Å per pixel with a defocus range of −2.8 to −0.8 μm. Movies were recorded in counting mode on a Falcon 4i direct electron detector (Thermo) in electron event representation (EER) format using EPU v3.4. A dose rate and exposure time was set to generate a total dose of ∼40 electrons Å^−2^. Statistics for cryo-EM data collection are listed in Supplementary Table 18.

All processing was done in cryoSPARC 4.2.1 (ref. ^58^). A total of 17,017 movies were dose weighted and motion and contrast transfer function (CTF) corrected in patch mode. Particles were picked with cryoSPARC template picker and 2 × 2 binned particles (1,810,321) were subjected to several rounds of 2D classification (50 iterations, 20 full iterations and batchsize 300). Cleaned particles representing holoenzymes with secondary structure elements visible (169,234) underwent three-dimensional classification with two classes (ab initio, 143,883 particles retained) and was refined to 3.05 Å resolution using a non-uniform refinement procedure^59^. Particles were re-extracted as unbinned using updated coordinates and downsampled to 1.02 Å per pixel. Further non-uniform refinement of this particle set with local CTF correction^60^, global CTF refinement (tilt, trefoil, spherical aberration anisotropic magnification) and Ewald sphere correction^61^ resulted in a final map of estimated 2.88 Å global resolution and 2.7 Å local resolution around the drug binding site allowing clear visualization of LEI-800, DNA and surrounding sidechains. Attempts were made to improve the resolution in different regions, that is, the GyrB TOPRIM insert by further focussed classification but they did not bring further improvement nor indicate particular heterogeneity in the dataset. Post-processing by DeepEMhancer^62^ was used to help model building for regions with poorer density.

### Model building and refinement

The closest available structure of *E.* *coli* gyrase (PDB: 7Z9C) was used as a starting point for model building. The initial model was rigid-body fitted in ChimeraX^63^ and manually adjusted in Coot^64^ and ISOLDE^65^. Real-space refinement was performed in phenix.refine^66^ (using Ramachandran restrains, secondary structure restraints for protein and DNA and NCS (non-crystallographic symmetry) restraints). The model and restraints for LEI-800 were obtained using Grade server (http://grade.globalphasing.org). After building protein and DNA, LEI-800 was automatically placed into the density by phenix.ligandfit and further refined in Phenix with the rest of the model. The model was refined against the 2.88 Å map auto-sharpened by cryoSPARC (B factor −66.4) described above. MolProbity^67^ and MTriage^68^ were used to validate the structure. Statistics for the final model are reported in Supplementary Table 18.

### Reporting summary

Further information on research design is available in the Nature Portfolio Reporting Summary linked to this article.

## Online content

Any methods, additional references, Nature Portfolio reporting summaries, source data, extended data, supplementary information, acknowledgements, peer review information; details of author contributions and competing interests; and statements of data and code availability are available at 10.1038/s41557-024-01516-x.

## Supplementary information

Supplementary Information Supplementary Tables 1–18, Figs. 1–12, synthetic procedures and NMR spectra key compounds, and source data uncropped gels.

Reporting Summary

Supplementary Tables Table 1. Initial library screen. Table 2. H-89 derivatives rescreen. Table 3. SAR overview. Table 4. BCP features. Table 5. WGS results. Table 6. Source data Supplementary Fig. 1a/b. Table 7. Source data Supplementary Fig. 2. Table 8. Source data Supplementary Fig. 3. Table 9. Source data Supplementary Fig. 4.

## Source data

Source Data Fig. 4 Uncropped gels used for quantification in Fig. 4c and example gels in Fig. 4d.

Source Data Fig. 4 Data for the PMBN synergy assay.

## Figures and Tables

**Extended Data Table 1:**
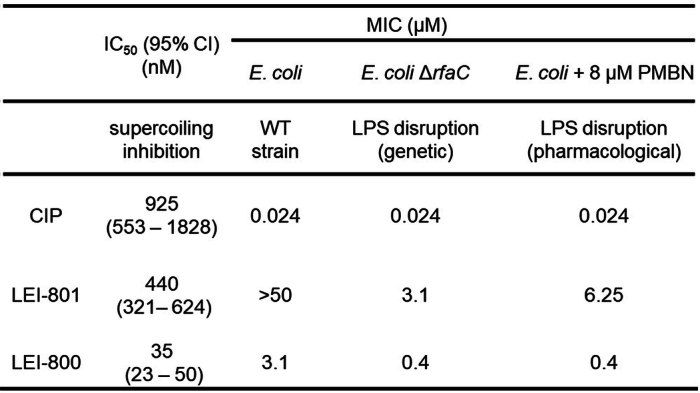
Supercoiling inhibition alongside antimicrobial activity in WT *E. coli* and in genetic and pharmacological LPS disruption backgrounds

**Extended Data Table 2:**
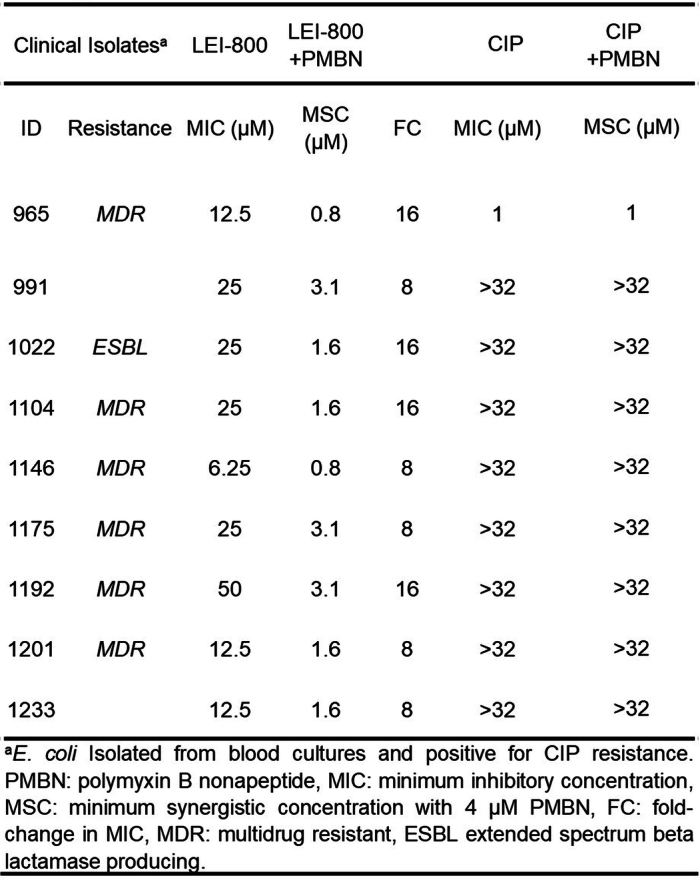
Antibacterial activity of LEI-800 against CIP resistant clinical isolates of *E. coli*

## Data Availability

Source data for figures are provided as separate Source data files. All source data for supplementary figures are listed in the Supplementary Data file, except for uncropped gels which are listed at the end of the Supplementary Information. The raw microscopy images used for the BCP morphological analysis and quantification are available from BioImage Archive (S-BIAD1079). WGS data are available at the National Center for Biotechnology Information through the BioProject accession number PRJNA855320. The Gyr–LEI-800 coordinates have been submitted to the Protein Data Bank (https://www.rcsb.org/) with ID 8QQI. The corresponding EM maps have been submitted to the Electron Microscopy Data Bank (https://www.ebi.ac.uk/pdbe/emdb/) with ID EMD-18592. The raw data were submitted the Electron Microscopy Public Image Archive (https://www.ebi.ac.uk/pdbe/emdb/empiar/) with ID EMPIAR-11884. Source data are provided with this paper.

## Code availability

The custom ImageJ macros that were used to analyse nucleoids and DAPI/SYTOX intensity in the BCP assay are available from GitHub.
