# Supplementary material for: Discovery of isoquinoline sulfonamides as allosteric gyrase inhibitors with activity against fluoroquinolone-resistant bacteria
Source: Nat Chem. 2024 Jun 19;16(9):1462–72. doi: 10.1038/s41557-024-01516-x (PMC11374673; doi:10.1038/s41557-024-01516-x)

nature chemistry

Supplementary information

https://doi.org/10.1038/s41557-024-01516-x

# Discovery of isoquinoline sulfonamides as allosteric gyrase inhibitors with activity against fluoroquinolone-resistant bacteria

In the format provided by the authors and unedited

## Table of contents

**Supplementary Tables** ........ 3
Supplementary Table 1. ........ 3
Supplementary Table 2. ........ 4
Supplementary Table 3. ........ 5
Supplementary Table 4. ........ 6
Supplementary Table 5. ........ 7
Supplementary Table 6. ........ 8
Supplementary Table 7. ........ 9
Supplementary Table 8. ........ 9
Supplementary Table 9. ........ 10
Supplementary Table 10. ........ 10
Supplementary Table 11 ........ 11
Supplementary Table 12 ........ 11
Supplementary Table 13. ........ 11
Supplementary Table 14a........ 11
Supplementary Table 14b. ........ 12
Supplementary Table 15. ........ 12
Supplementary Table 16. ........ 13
Supplementary Table 17. ........ 13
Supplementary Table 18. ........ 14
**Supplementary Figures** ........ 15
Supplementary Figure 1a. ........ 15
Supplementary Figure 1b ........ 16
Supplementary Figure 2. ........ 16
Supplementary Figure 3. ........ 17
Supplementary Figure 4. ........ 17
Supplementary Figure 5. ........ 18
Supplementary Figure 6. ........ 19
Supplementary Figure 7. ........ 20
Supplementary Figure 8. ........ 22
Supplementary Figure 9. ........ 23
Supplementary Figure 10. ........ 24
Supplementary Figure 11. ........ 25

Supplementary Figure 12. .......... 26
**Synthetic procedures** .......... 28
General remarks .......... 28
Compound characterization .......... 31
**NMR spectra of key compounds** .......... 64
**References** .......... 69
**Source data uncropped gels** .......... 70
Source data for the Supplementary Figure 5 .......... 70
Source data for the Supplementary Figure 8 (a) .......... 71
Source data for the Supplementary Figure 8 (b,c) .......... 72
Source data for the Supplementary Figure 8 (b,c) .......... 73
Source Data for the Supplementary Figure 9 (a,b) .......... 74
Source Data for the Supplementary Figure 9 (a,b) .......... 75
Source data for Supplementary Figure 11 (c) .......... 76
Source data for Supplementary Figure 11 (c) .......... 77
Source Data for the Supplementary Figure 12 (e) .......... 78
Source Data for the Supplementary Figure 12 (e) .......... 79

## Supplementary Tables

**Supplementary Table 1.** Structures of hit **1** and tested kinase inhibitor analogues **2**-**46**. Analogues **2**-**46** showed no antibacterial activity against *E. coli* at 50 µM and lower.

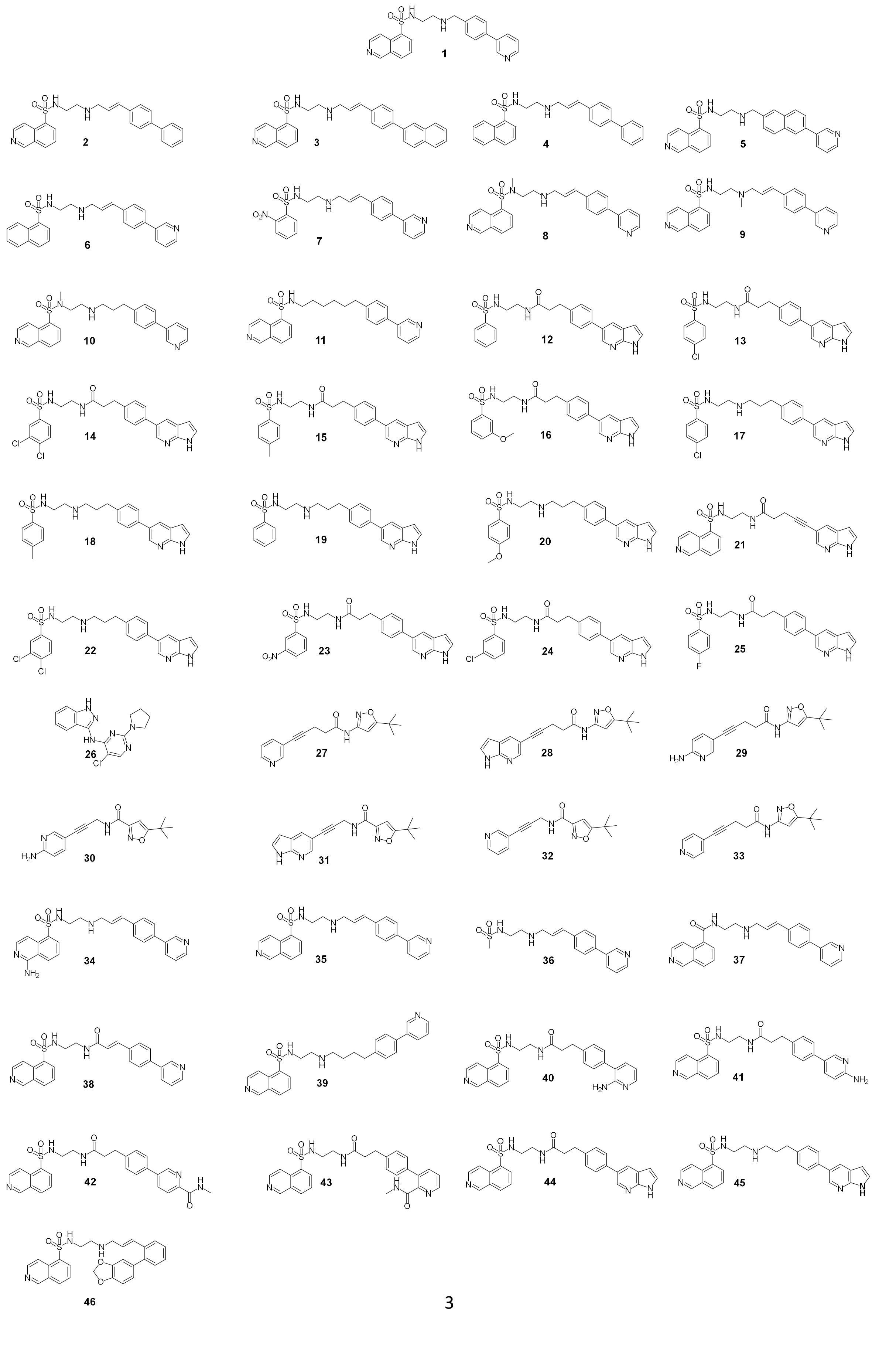

**Supplementary Table 2.** MIC values of isoquinoline derivatives of hit **1**.

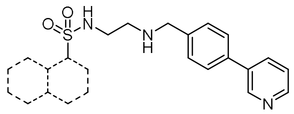


| ID | R | MIC (μM) | | ID | R | MIC (μM) | |
|---|---|---|---|---|---|---|---|
| | | *E. coli* | *K. pneumoniae* | | | *E. coli* | *K. pneumoniae* |
| **1** | 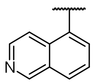 | 6.25 | 12.5 | **51** | 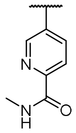 | >50 | >50 |
| **47** | 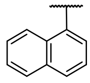 | 25 | >50 | **52** | 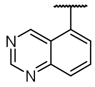 | >50 | >50 |
| **48** | 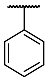 | >50 | >50 | **53** | 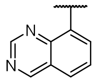 | >50 | >50 |
| **49** | 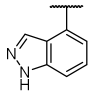 | >50 | >50 | **54** | 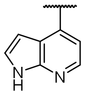 | >50 | >50 |
| **50** | 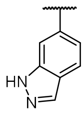 | >50 | >50 | **55** | 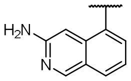 | >50 | >50 |

**Supplementary Table 3.** MIC values of outer ring derivatives of hit **1**.

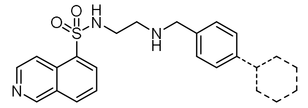

| ID | R | MIC (µM) | | ID | R | MIC (µM) | |
|---|---|---|---|---|---|---|---|
| | | *E. coli* | *K. pneumoniae* | | | *E. coli* | *K. pneumoniae* |
| **1** | 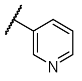 | 6.25 | 12.5 | **67** | 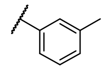 | >50 | >50 |
| **56** | 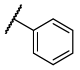 | 6.25 | 12.5 | **68** | 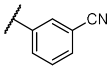 | 12.5 | 25 |
| **57** | 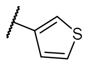 | 6.25 | 12.5 | **69** | 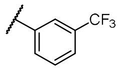 | 25 | >50 |
| **58** | 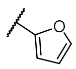 | 25 | 25 | **70** | 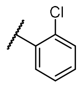 | 25 | 50 |
| **59** | 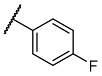 | 3.1 | 6.25 | **71** | 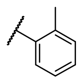 | 12.5 | 50 |
| **60** | 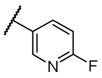 | 6.25 | 6.25 | **72** | 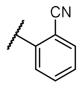 | 25 | 50 |
| **61** | 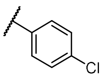 | 6.25 | >50 | **73** | 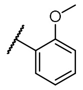 | 25 | 50 |
| **62** | 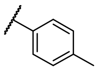 | >50 | >50 | **74** | 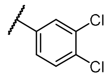 | 25 | >50 |
| **63** | 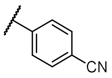 | 6.25 | 12.5 | **75** | 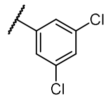 | >50 | >50 |
| **64** | 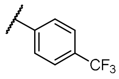 | >50 | >50 | **76** | 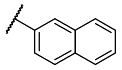 | >50 | >50 |
| **65** | 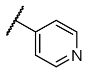 | 12.5 | 25 | **77** | 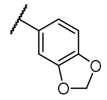 | >50 | >50 |
| **66** | 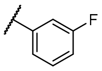 | 12.5 | 25 | **78** | 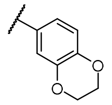 | >50 | >50 |

**Supplementary Table 4.** MIC values of inner ring derivatives of hit **1**.

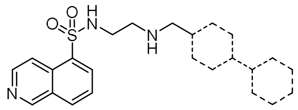

| ID | R | MIC (μM) *E. coli* | MIC (μM) *K. pneumoniae* | ID | R | MIC (μM) *E. coli* | MIC (μM) *K. pneumoniae* |
|---|---|---|---|---|---|---|---|
| **1** | 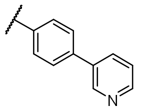 | 6.25 | 12.5 | **83** | 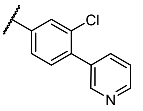 | >50 | >50 |
| **56** | 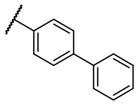 | 6.25 | 12.5 | **84** | 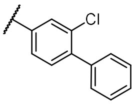 | >50 | >50 |
| **60** | 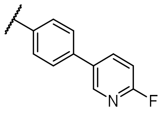 | 6.25 | 6.25 | **85** | 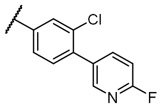 | >50 | >50 |
| **59** | 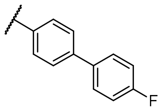 | 3.1 | 6.25 | **86** | 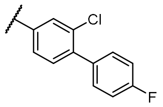 | 50 | >50 |
| **79** | 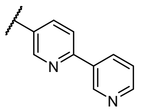 | >50 | >50 | **87** | 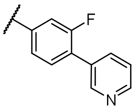 | 25 | 50 |
| **80** | 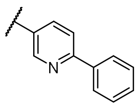 | >50 | >50 | **88** | 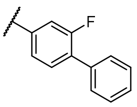 | 12.5 | 25 |
| **81** | 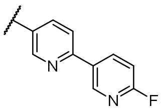 | >50 | >50 | **89** | 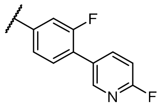 | 6.25 | 12.5 |
| **82** | 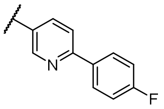 | >50 | >50 | **90** | 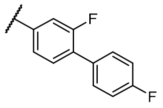 | 12.5 | 12.5 |

**Supplementary Table 5.** MIC values of linker derivatives of hit **1**.

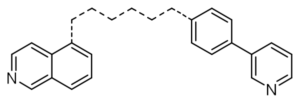

| ID | R | MIC (µM) | |
|---|---|---|---|
| | | *E. coli* | *K. pneumoniae* |
| **1** | 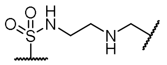 | 6.25 | 12.5 |
| **91** | 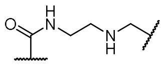 | >50 | >50 |
| **92** | 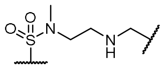 | >50 | >50 |
| **93** | 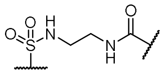 | >50 | >50 |
| **94** | 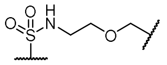 | >50 | >50 |
| **95** | 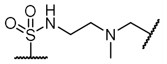 | >50 | 50 |

**Supplementary Table 6.** Cytotoxicity data derivatives hit **1**.

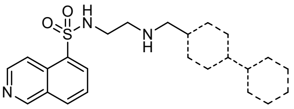


| ID | R | MIC (μM) | | $IC_{50}$ (μM) | |
|---|---|---|---|---|---|
| | | *E. coli* | *K. pneumoniae* | HEK293T | HepG2 |
| **56** | 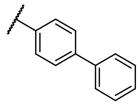 | 6.25 | 12.5 | 10.4 | 16.7 |
| **57** | 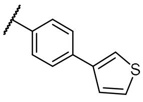  | 6.25 | 12.5 | 6.6 | 8.2 |
| **59** | 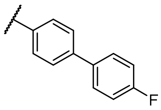  | 3.1 | 6.25 | 2.6 | 1.7 |
| **60** | 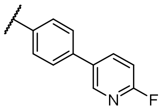  | 6.25 | 6.25 | 51 | >100 |
| **61** | 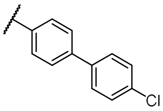  | 6.25 | 50 | 1.0 | 2.8 |
| **63** | 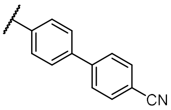  | 6.25 | 12.5 | 14.7 | 38 |
| **89** | 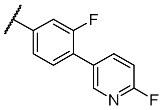  | 6.25 | 12.5 | 8.9 | 6.8 |

**Supplementary Table 7.** Antimicrobial spectrum of isoquinoline sulfonamides.

| Strain | | MIC (µM) **1** | **60** | **101** | **102** (LEI-800) | CIP |
|---|---|---|---|---|---|---|
| *K. pneumoniae* | ATCC 29665 | 12.5 | 25 | 3.1 | 6.25 | 0.012 |
| *P. aeruginosa* | ATCC 27853 | >50 | >50 | >50 | >50 | 0.377 |
| *A. baumannii* | ATCC BAA747 | >50 | >50 | 25 | 25 | 0.755 |
| *E. coli* | | | | | | 0.024 |
| | W3110 | 6.25 | 6.25 | 1.6 | 3.1 | 0.024 |
| | BW25113 | 12.5 | 6.25 | 1.6 | 3.1 | 0.024 |
| | ATCC 25922 | 25 | 12.5 | 3.1 | 6.25 | 0.024 |
| *S. aureus* | | | | | | |
| | ATCC 29213 | >50 | >50 | >50 | >50 | 0.755 |
| | ATCC BAA1717 | >50 | >50 | >50 | >50 | >0.755 |

**Supplementary Table 8.** MIC (µM) of selection of compounds against clinical isolates of *E. coli* and potentiation by addition of 4 µM PMBN.

| ***E. coli*** **strain** | **Resistance profile** | **60** | **60 + PMBN** | | **101** | **101 + PMBN** | | **102** | **102 + PMBN** | | **CIP** | **CIP + PMBN** |
|---|---|---|---|---|---|---|---|---|---|---|---|---|
| | | MIC | MSC | FC | MIC | MSC | FC | MIC | MSC | FC | MIC | MSC |
| mcr-1 | *MCR* | 50 | 12.5 | *4* | 12.5 | 3.1 | *4* | 25 | 6.25 | *4* | >32 | >32 |
| NCTC13463 | *ESBL* | 12.5 | 1.6 | *8* | 3.1 | 0.4 | *8* | 3.1 | 0.4 | *8* | 1 | 1 |
| NCTC13846 | *MDR (mcr-1)* | >50 | 25 | *>2* | 12.5 | 3.1 | *4* | 50 | 12.5 | *4* | >32 | >32 |
| MVAST0072# | *MDR* | 50 | 3.1 | *16* | 12.5 | 0.8 | *16* | 25 | 1.5 | *16* | >32 | >32 |
| 552059.1# | | 50 | 3.1 | *16* | 12.5 | 0.8 | *16* | 25 | 0.78 | 32 | 1 | 1 |
| 552060.1# | | 50 | 3.1 | *16* | 12.5 | 0.8 | *16* | 25 | 1.5 | *16* | 1 | 1 |
| 1075 | *MDR* | >50 | 25 | *>2* | 50 | 6.25 | *8* | >50 | 12.5 | *>4* | 0.13 | 0.13 |

PMBN: Polymyxin B nonapeptide, CIP: ciprofloxacin, MSC: minimum synergistic concentration, FC: fold change in MIC after addition of 4 µM PMBN, MCR: mobile colistin resistance, MDR: multidrug resistant, ESBL: extended spectrum beta lactamase, # urinary tract infection isolates. Compound **102** is generally referred to as LEI-800.

**Supplementary Table 9.** Parameters used during image analysis

| Software | Analysis | Parameters/Command |
|---|---|---|
| ImageJ[1] (v 1.53m) | Qualitative BCP – DAPI channel | run("Subtract Background...", "rolling=15"); run("Mean...", "radius=1"); |
| | Qualitative BCP – FM4-64 channel | run("Subtract Background...", "rolling=5"); run("Enhance Contrast...", "saturated=0.3"); run("Mean...", "radius=1"); |
| | Qualitative BCP – DIC channel | No preprocessing |
| | Quantitative BCP – DIC channel | run("Gaussian...", "radius=1.5"); |
| | Quantitative BCP – DAPI shape descriptors | Custom made script (available from GitHub) |
| | Quantitative BCP – DAPI and SYTOX Green intensity | Custom made script (available from GitHub) |
| Illastik[2] (v 1.3.3) | DIC image segmentation | All features selected<br>Classifier trained for label "cell" or "background" |
| MicrobeJ[3] (v 5.13l) | Quantitative BCP – DIC shapes | Image used for bacteria detection: DIC channel<br>Segmentation based on: binary mask generated by Illastik.<br>Bacteria detection = fit shape (rod shaped)<br>Attributes: Area (1.5 – max); Length (1.5 – max); Width (0.5 – max); Circularity (0 – max)<br>Enabled options: Exclude on Edges; Shape descriptors; Segmentation (default settings); Intensity; Shape; Profile (medial, default settings) |

**Supplementary Table 10.** MIC of known antibiotics and compounds against selected 60-resistant (60-r) selected isolates of W3110.

| Antibiotic | Mode of Action | MIC µM (µg/mL) *E. coli* | | | |
|---|---|---|---|---|---|
| | | W3110 | 60-r1 | 60-r3 | 60-r6 |
| **1** | | 6.25 | >100 | 100 | >100 |
| **60** | | 6.25 | >100 | 100 | >100 |
| **101** | | 1.6 | >50 | 6.25 | 3.1 |
| **102** (LEI-800) | | 3.1 | >50 | 12.5 | 12.5 |
| Imipenem | PBP - cell wall | 0.375 (0.1) | 0.75 (0.2) | 0.375 (0.1) | 0.375 (0.1) |
| Meropenem | PBP - cell wall | 0.05 (0.02) | 0.09 (0.04) | 0.05 (0.02) | 0.05 (0.02) |
| Ceftazidime | PBP - cell wall | 0.75 (0.4) | 0.375 (0.2) | 0.75 (0.4) | 0.75 (0.4) |
| Colistin | Outer membrane | 0.19 (0.5) | 0.375 (1) | 0.375 (1) | 0.19 (0.5) |
| Trimethoprim | Folic acid synthesis | 0.375 (0.16) | 0.375 (0.16) | 0.18 (0.08) | 0.375 (0.16) |
| Neomycin | Protein synthesis (30S) | 1.5 (1) | 0.75 (0.5) | 1.5 (1) | 0.75 (0.5) |
| Chloramphenicol | Protein synthesis (50S) | 12.4 (4) | 12.4 (4) | 12.4 (4) | 12.4 (4) |
| Ciprofloxacin | DNA replication | 0.024 (0.0078) | 0.024 (0.0078) | 0.024 (0.0078) | 0.024 (0.0078) |

**Supplementary Table 11**. CRISPR-*gyrA* editing. Target validation by introduction of point mutations with CRISPR/Cas9 on an *E. coli* W3110 background. MIC values are in agreement with sequenced spontaneous mutants.

| *E. coli* W3110 | | MIC (µM) | | | |
|---|---|---|---|---|---|
| Mutation | Note | **60** | **101** | **102** (LEI-800) | CIP |
| WT | WT | 6.25 | 1.6 | 3.1 | 0.024 |
| S97L | LEI-800-resistant | >50 | >50 | >50 | 0.024 |
| S83L | FQ-resistant 1 | 25 | 3.1 | 6.25 | >1.5 |
| D87N | FQ-resistant 2 | 50 | 3.1 | 6.25 | >1.5 |

**Supplementary Table 12**. MIC (µM) on *E. coli* strains of the Keio collection.

| Strain | Deletion | CIP | **99** (LEI-801) | **102** (LEI-800) |
|---|---|---|---|---|
| BW25113 | parent | 0.024 | >50 | 3.1 |
| JW5503 | Δ*TolC* | 0.024 | >50 | 3.1 |
| JW3600 | Δ*rfaY* | 0.024 | >50 | 1.6 |
| JW3602 | Δ*rfaI* | 0.024 | >50 | 1.6 |
| JW3605 | Δ*rfaP* | 0.048 | 12.5 | 0.4 |
| JW3594 | Δ*rfaD* | >0.048 | 3.1 | 0.4 |
| JW3596 | Δ*rfaC* | 0.024 | 3.1 | 0.4 |

**Supplementary Table 13.** Frequency of resistance for LEI-800, CIP, and LEI-800+Cip combination with *E. coli* 3110 .

| Compound | Concentration | |
|---|---|---|
| | 5 x MIC | 10 x MIC |
| LEI-800 | $(4.7 \pm 1.9)\cdot 10^{-7}$ | $(1.29 \pm 0.2)\cdot 10^{-7}$ |
| CIP | $(1.28 \pm 0.1)\cdot 10^{-6}$ | $(1.4 \pm 0.0)\cdot 10^{-8}$ |
| LEI-800+CIP | $< 1\cdot 10^{-8}$ | n.t. |

Results are represented as the average of three experiments with standard deviation. LEI-800 is also denoted as **102**. MIC of LEI-800 3.1 µM and CIP 0.024 µM. n.t.: not tested.

**Supplementary Table 14a.** Homology DNA gyrase subunit A (*E. coli*).

| Organism | Strain | Uniprot ID | Protein | Gene | Homology[a] |
|---|---|---|---|---|---|
| *E. coli* | K12 | GYRA_ECOLI | DNA gyrase subunit A | gyrA | 100% |
| *K. pneumoniae* | 342 | B5XNZ4_KLEP3 | DNA gyrase subunit A | gyrA | 92.0% |
| *E. faecium* | ATCC 700802 | H7C793_ENTFA | DNA gyrase subunit A | gyrA | 47.1% |
| *S. aureus* | - | GYRA_STAA3 | DNA gyrase subunit A | gyrA | 46.3% |
| *A. baumannii* | - | Q2FCU6_ACIBA | DNA gyrase subunit A | gyrA | 59.5% |
| *P. aeruginosa* | ATCC 15692 | GYRA_PSEAE | DNA gyrase subunit A | gyrA | 63.0% |
| *Homo sapiens* | | TOP2A_HUMAN | DNA topoisomerase 2-alpha | TOP2A | 23.4% |
| *Homo sapiens* | | TOP2B_HUMAN | DNA topoisomerase 2-beta | TOP2B | 25.6% |

[a]Calculated by Uniprot Align function

| **Supplementary Table 14b.** Homology DNA gyrase subunit B (*E. coli*). | | | | | |
|---|---|---|---|---|---|
| Organism | Strain | Uniprot ID | Protein | Gene | Homology[a] |
| *E. coli* | K12 | GYRB_ECOLI | DNA gyrase subunit B | gyrB | 100% |
| *K. pneumoniae* | 342 | J2E0G5_KLEPN | DNA gyrase subunit B | gyrB | 92.0% |
| *E. faecium* | ATCC 700802 | Q93HU9_ENTFL | DNA gyrase subunit B | gyrB | 44.0% |
| *S. aureus* | - | GYRB_STAA3 | DNA gyrase subunit B | gyrB | 43.6% |
| *A. baumannii* | - | GYRB_ACIBA | DNA gyrase subunit B | gyrB | 35.7% |
| *P. aeruginosa* | ATCC 15692 | GYRB_PSEAE | DNA gyrase subunit B | gyrB | 68.2% |
| *Homo sapiens* | | TOP2A_HUMAN | DNA topoisomerase 2-alpha | TOP2A | 25.8% |
| *Homo sapiens* | | TOP2B_HUMAN | DNA topoisomerase 2-beta | TOP2B | 25.8% |
| [a]Calculated by Uniprot Align function | | | | | |

| **Supplementary Table 15.** Strains and plasmids used for the construction of the *gyrA* mutants. | | |
|---|---|---|
| Plasmid | Characteristics | Source or reference |
| *E. coli* W3110 | Wild type ($F^{-}\lambda^{-}$rph-1 IN(rrnD, rrnE)) | Jensen, 1993[4] |
| *E. coli* DH5α | fhuA2 Δ (argF-lacZ)U169 phoA glnV44 Φ80 Δ(lacZ)M15 gyrA96 recA1 relA1 endA1 thi-1 hsdR17 | Meselson & Yuan, 1968[5] |
| pCas | repA101(Ts), kan$^{r}$ , $P_{cas}$-cas9, $P_{araB}$-Red, lacI$^{q}$, $P_{trc}$-sgRNA-pMB1; temperature-sensitive replication vector | Jiang et al, 2015[6] |
| pTargetF | pMB1, *aadA*, sgRNA-fts | Jiang et al, 2015[6] |
| psgRNA-gyrA-M5 | Plasmid used to insert the S97L mutation in *gyrA* | This study |
| psgRNA-gyrA-S83L | Plasmid used to insert the S83L mutation in *gyrA* | This study |
| psgRNA-gyrA-D87N | Plasmid used to insert the D97N mutation in *gyrA* | This study |
| pET28b-EcGyrATWS | Purification of 10xHis- and 2xSTREP-tagged *E. coli* GyrA | Gift of Dr Valérie Lamour (University of Strasbourg) |
| pET28b-EcGyrBTWS | Purification of 10xHis- and 2xSTREP-tagged *E. coli* GyrB | Gift of Dr Valérie Lamour (University of Strasbourg) |
| pET28b-EcGyrA-S97L-TWS | Purification of 10xHis- and 2xSTREP-tagged *E. coli* GyrA-S97L | This study |
| pET28b-EcGyrA-S172A-TWS | Purification of 10xHis- and 2xSTREP-tagged *E. coli* GyrA-S172A | This study |

| **Supplementary Table 16.** Primers used for the construction of the *gyrA* mutants. | |
|---|---|
| Primers | Sequence (5' to 3') |
| gyrA_P05 | TTCTCTAGAGTCGACCTGCAGGAAGTCCGGCCCCGGGATGTGT |
| gyrA_P08 | TCGAGTAGGGATAACAGGGTAATATCTAGAGGTTTACCGGCGATTTTTCGGCATTCAT |
| gyrA_P09 | ATCAGCCCTTCAATGCTGATG |
| gyrA_P10 | TCCGTAATTGGCAAGACAAAC |
| gyrA_P11 | CTCAGTCCTAGGTATAATACTAGTAGCATATAACGCAGCGAGAAGTTTTAGAGCTAGAAATAGCAAGTTAAAATAAG |
| gyrA_P12 | CTGCAGGTCGACTCTAGAGAATTCAAAAAAAGCACCGACTC |
| gyrA_P13 | TTTACTACGTTACATGCTGGTAGACGGTCAGGGTAACTTCG |
| gyrA_P14 | CCAGCATGTAACGTAGTAAAAATGGCTGCGCCATGCGGACGATC |
| gyrA_P17 | CTCAGTCCTAGGTATAATACTAGTCATAGACCGCCGAGTCACCAGTTTTAGAGCTAGAAATAGCAAGTTAAAATAAG |
| gyrA_P18 | GGAGATCTGGCTGTTTATGACACGATCGTCCGCATGGCGCAG |
| gyrA_P19 | GTCATAAACAGCCAGATCTCCATGGGGATGGTATTTACCGATTACGTCACCAACGAC |
| gyrA_P20 | CTCAGTCCTAGGTATAATACTAGCTATGACACGATCGTCCGCAGTTTTAGAGCTAGAAATAGCAAGTTAAAATAAG |
| gyrA_P21 | CTATAACACAATAGTAAGGATGGCGCAGCCATTCTCGCTGCG |
| gyrA_P22 | GCCATCCTTACTATTGTGTTATAGACCGCCGAGTCACCATGGGGATGG |
| | |

| **Primers used for construction of S97L and S172A mutations:** | | |
|---|---|---|
| GyrA-S97L-for | GCGCAGCCATTCTTGCTGCGTTATATGCTG | Introducing mutation in *E. coli gyrA* for residue S97 |
| GyrA-S97L-rev | CAGCATATAACGCAGC**A**AGAATGGCTGCGC | Introducing mutation in *E. coli gyrA* for residue S97 |
| GyrA-S172A-for | GTGAACGGTTCT**G**CCGGTATCGCCGTAG | Introducing mutation in *E. coli gyrA* for residue S172 |
| GyrA-S172A-rev | CTACGGCGATACCGG**C**AGAACCGTTCAC | Introducing mutation in *E. coli gyrA* for residue S172 |
| ColE1-for | GGAGCGAACGACCTACACCGAACTGAGATACCTACAGCG | Introducing point mutations in *E. coli gyrA* and *E. coli gyrB* |
| ColE1-rev | CGCTGTAGGTATCTCAGTTCGGTGTAGGTCGTTCGCTCC | Introducing point mutations in *E. coli gyrA* and *E. coli gyrB* |

**Supplementary Table 17.** Results from checkerboard assays of antibiotics and PMBN on *E. coli* W3110.

| | MSC (µM) | MIC (µM) | FICI (PMBN MIC>32 µM) |
|---|---|---|---|
| Ciprofloxacin | 0.025 | 0.05 | 1.5-2 |
| **99** (LEI-801) | 6.25 | 200 | ≤0.28 |
| **102** (LEI-800) | 0.39 | 3.13 | ≤0.25 |

*MSC: minimum synergistic concentration, MIC minimum inhibitory concentration, FICI: Fractional Inhibitory Concentration Index, PMBN: Polymyxin B nonapeptide.*

**Supplementary Table 18.** Data collection and refinement statistics.

| | **EcGyr-Mu217-LEI-800** |
|---|---|
| **Data collection and processing** | |
| Microscope | ThermoFisher Krios G2 |
| Magnification | 120,000× |
| Voltage (kV) | 300 |
| Electron dose ($e^-$/Å$^2$) | 39.58 |
| Detector | Falcon 4i |
| Defocus range (-µm) | 2.8-0.8 |
| Pixel size (Å) | 0.68 |
| Symmetry imposed | C1 |
| Micrographs (no.) | 17,017 |
| Initial particle images (no.) | 1,810,321 |
| Final particle images (no.) | 139,848 |
| Global map resolution (Å) | 2.9 |
| FSC threshold | 0.143 |
| **Refinement** | |
| Model resolution (Å)<br>FSC threshold | 3.14<br>0.5 |
| Map sharpening *B* factor (Å$^2$) | 66.4 |
| *Model composition*<br>Non-hydrogen atoms<br>Protein residues<br>Nucleotides<br>Ligands | <br>15,652<br>1832<br>52<br>4 |
| *Mean B factors (Å$^2$)*<br>Protein<br>Nucleotide<br>Ligands | <br>35.05<br>18.04<br>5.69 |
| *R.m.s. deviations*<br>Bond lengths (Å)<br>Bond angles (°) | <br>0.003<br>0.589 |
| **Validation** | |
| MolProbity score | 1.15 |
| Clashscore | 1.17 |
| *Ramachandran plot* Favored (%)<br>Allowed (%)<br>Disallowed (%) | 95.39<br>4.61<br>0.00 |

## Supplementary Figures

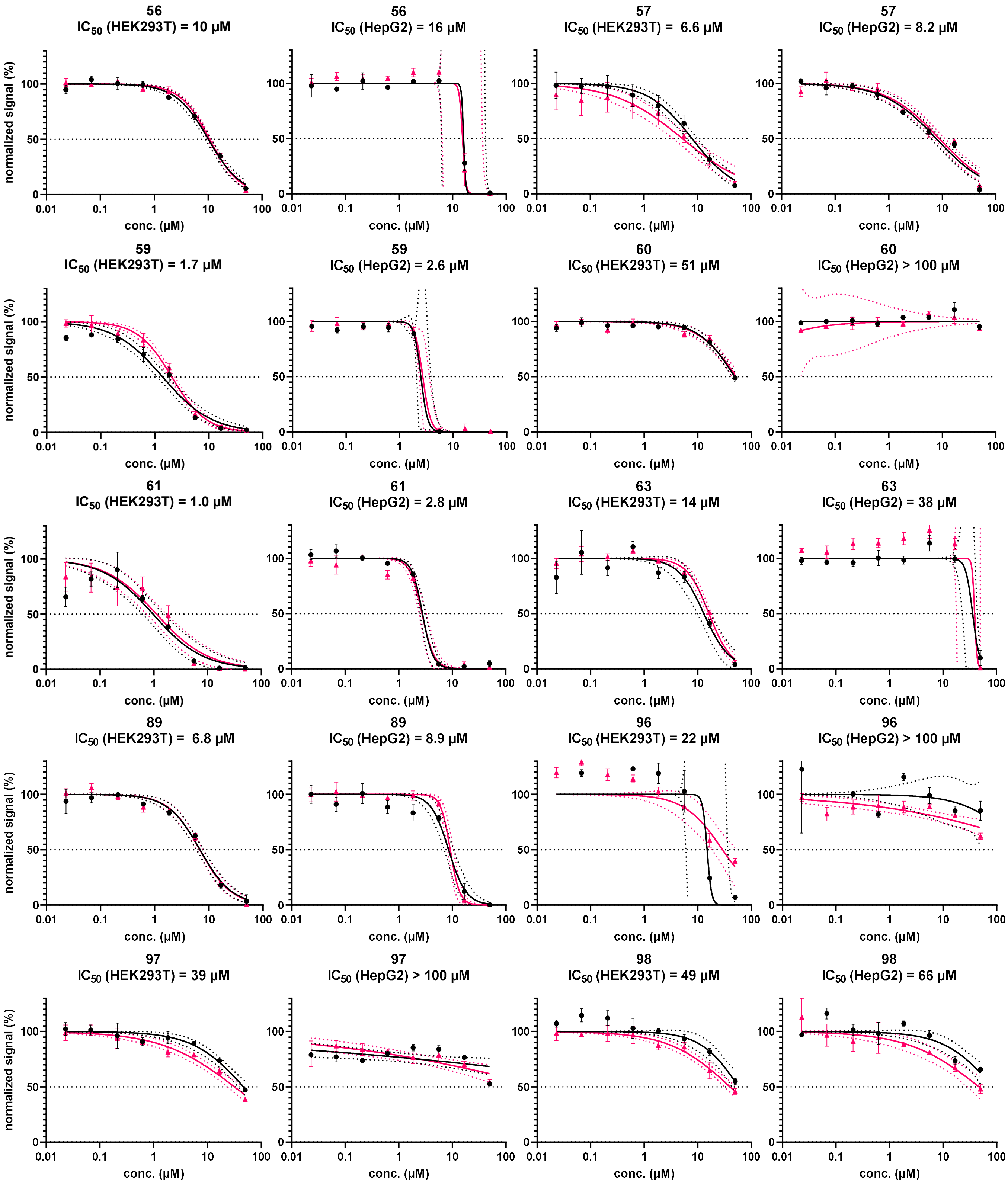


**Supplementary Figure 1a.** Non-linear regression curves showing the percentage of viable cells determined by MTT assay after treatment with isoquinoline sulfonamide analogues. Continues on next page.

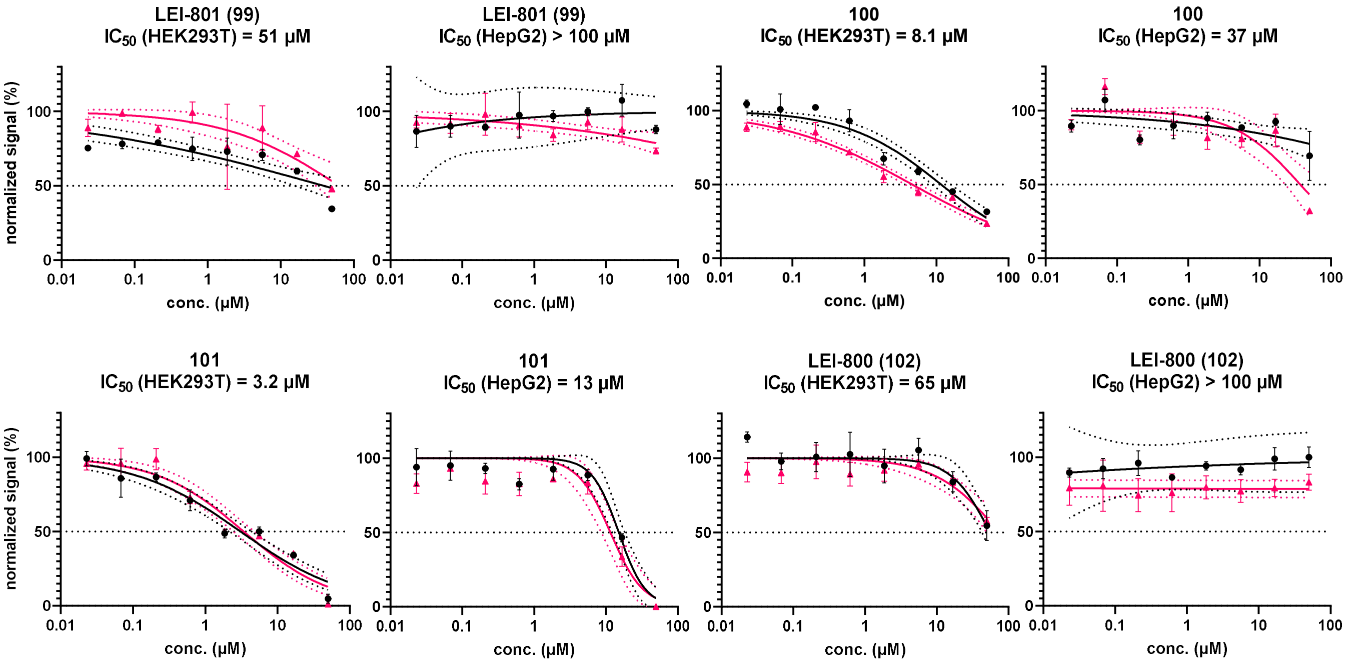


**Supplementary Figure 1b.** Non-linear regression curves showing the percentage of viable cells determined by MTT assay after treatment with isoquinoline sulfonamide analogues. Graphs representing the normalized absorbance relative to compound concentration. HEK293T: Human kidney, and HepG2: liver cell lines. The mean is shown with error bars and dotted lines representing the standard deviation at each concentration and the 95% CI respectively. n=2 biologically independent cell experiments executed with technical triplicates.

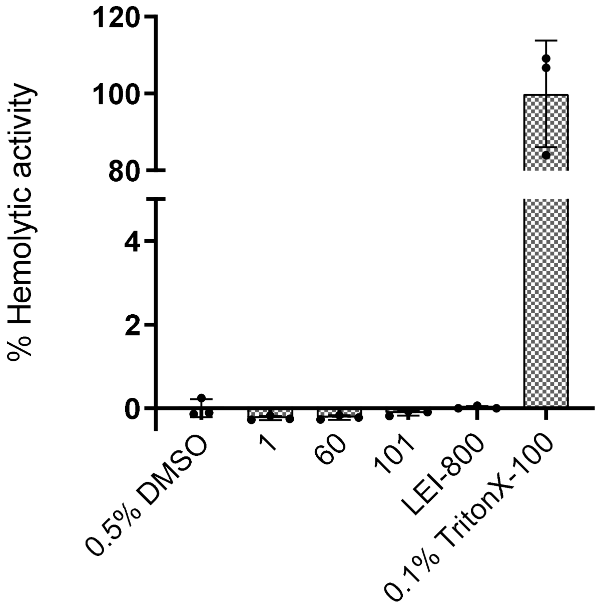


**Supplementary Figure 2.** Compounds show no hemolytic activity after 20 h incubation with sheep blood cells. Data are presented as mean values +/- SD of technical triplicates.

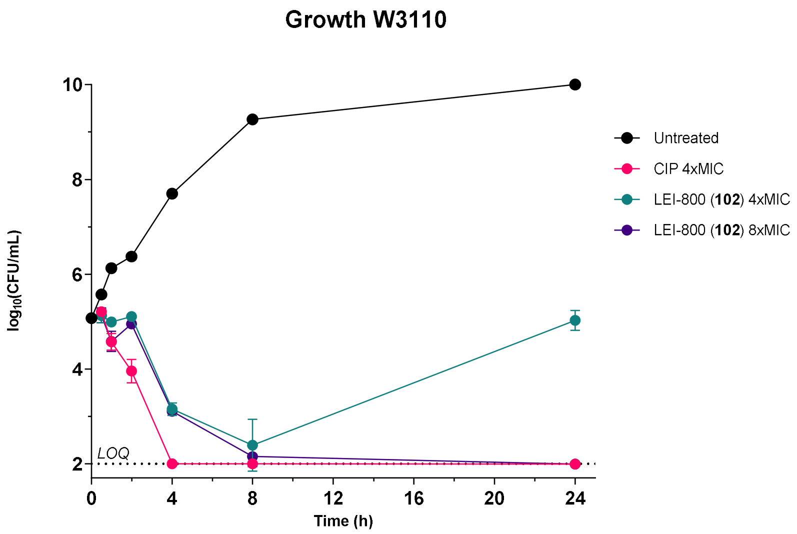


**Supplementary Figure 3.** Time-kill kinetics of LEI-800 (compound **102**) and CIP against *E. coli* W3110. Points represent mean values from n=2 biologically independent experiments, LOQ = limit of quantification.

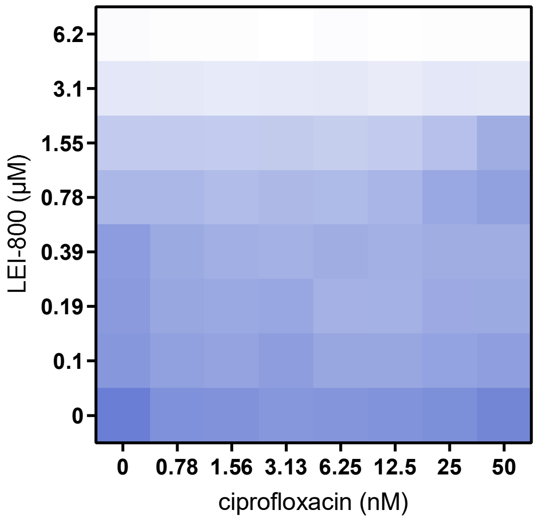


**Supplementary Figure 4.** Checkerboard broth microdilution assay between LEI-800 and CIP showing the absence of any synergistic effect.

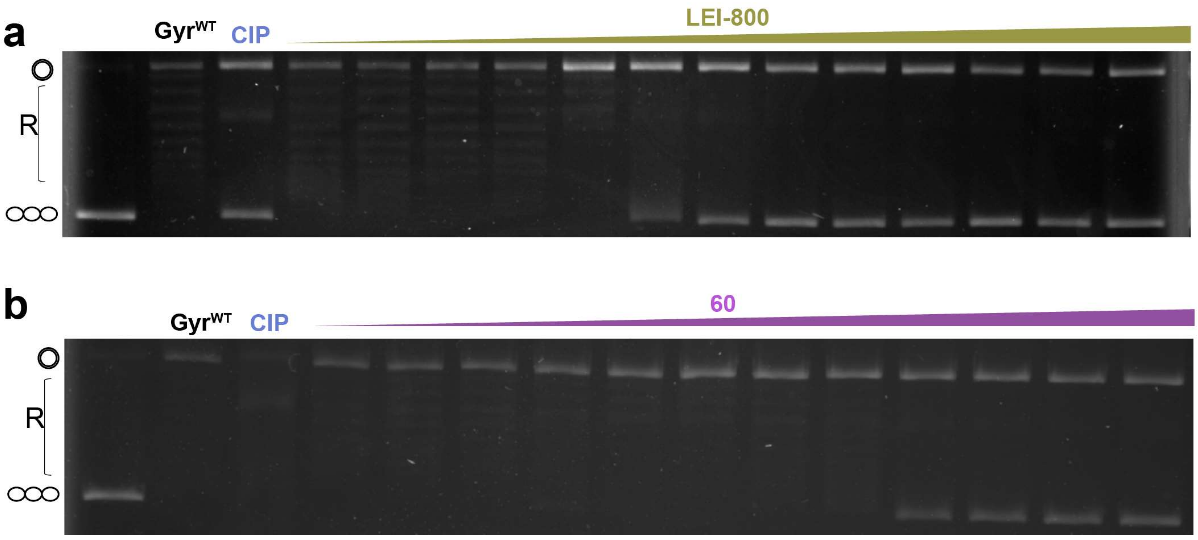


**Supplementary Figure 5. Effects of LEI-800 and compound 60 on ATP-independent relaxation of negatively supercoiled DNA by gyrase. a,** Relaxation of negatively supercoiled DNA in the presence of increasing concentrations of LEI-800. Lane 1: negatively supercoiled pBR322; lane 2: relaxation by 200 nM gyrase; lane 3: DNA relaxation in the presence of CIP (30 µM); subsequent lanes: the effect of increasing concentrations of LEI-800 (0.0005, 0.0015, 0.005, 0.015, 0.05, 0.15, 0.5, 1.5, 5, 10, 15, 20, 30 µM). **b,** Relaxation of negatively supercoiled DNA in a presence increasing concentrations of compound **60**. Lane 1: negatively supercoiled pBR322; lane 2: relaxation by 200 nM gyrase; lane 3: DNA relaxation in the presence of CIP (10 µM); subsequent lanes: the effect of increasing concentrations of compound **60** (0.0005, 0.0015, 0.005, 0.015, 0.05, 0.1, 0.15, 0.25, 0.5, 1.5, 5, 10 µM).

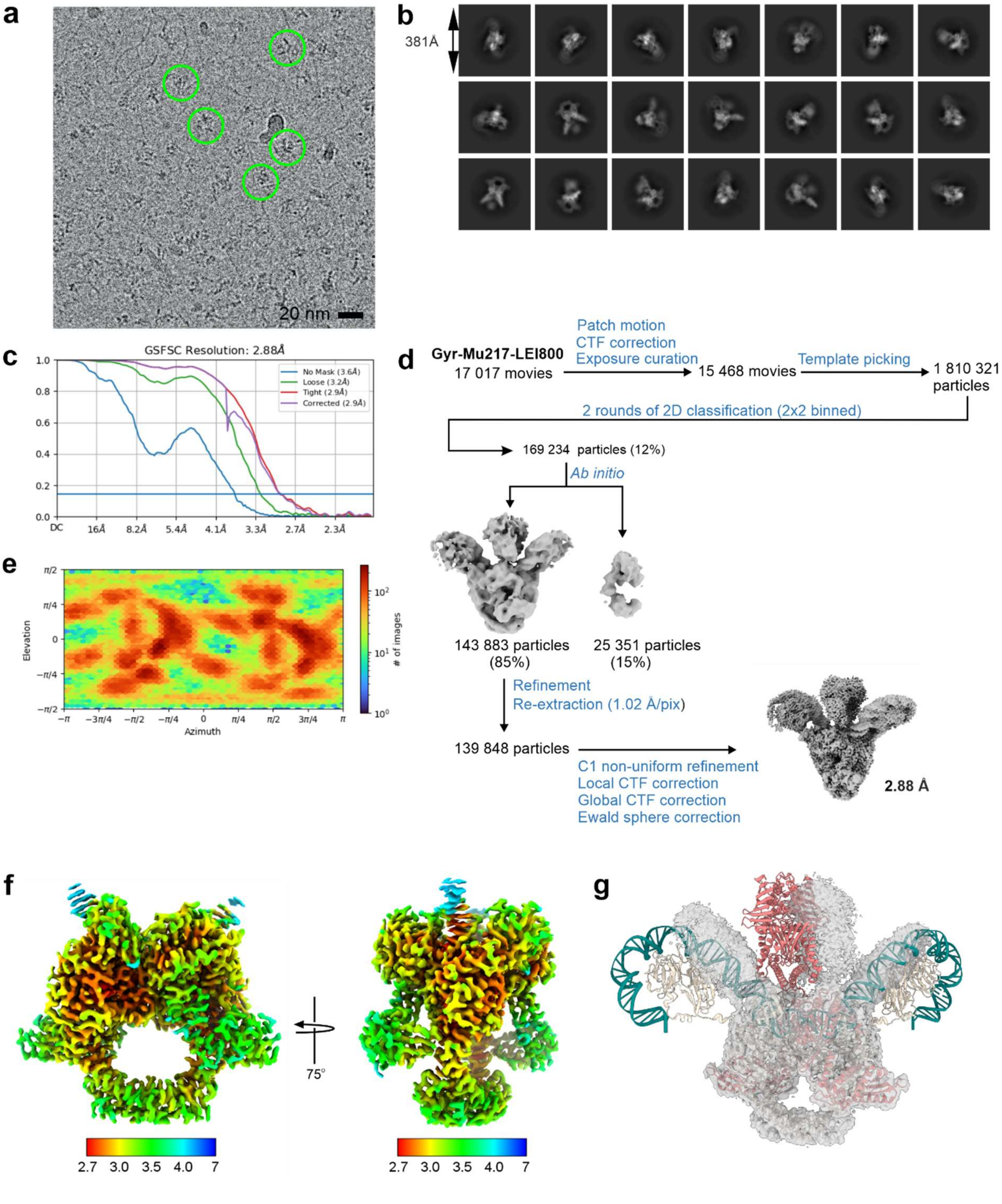


**Supplementary Figure 6. Cryo-EM data processing for Gyr-Mu217-LEI800. a,** A representative motion-corrected micrograph, gyrase particles are encircled. **b**, A selection of 2D classes, box size in angstroms indicated. **c,** FSC curve for the final reconstruction as output by cryoSPARC. **d**, Processing scheme (see *Methods* for description). **e,** Euler angle distribution as output by cryoSPARC. **f**, Local resolution maps illustrate resolution distribution from 2.7 Å next to the DNA and compound, to >7 Å towards the ends of the cleavage-reunion domain. **g**, Comparison of **Gyr-Mu217-LEI800** map (grey surface) to the gepotidacin EM structure PDB:6RKW (cartoon model). Note the significant change in the position of the CTDs and DNA.

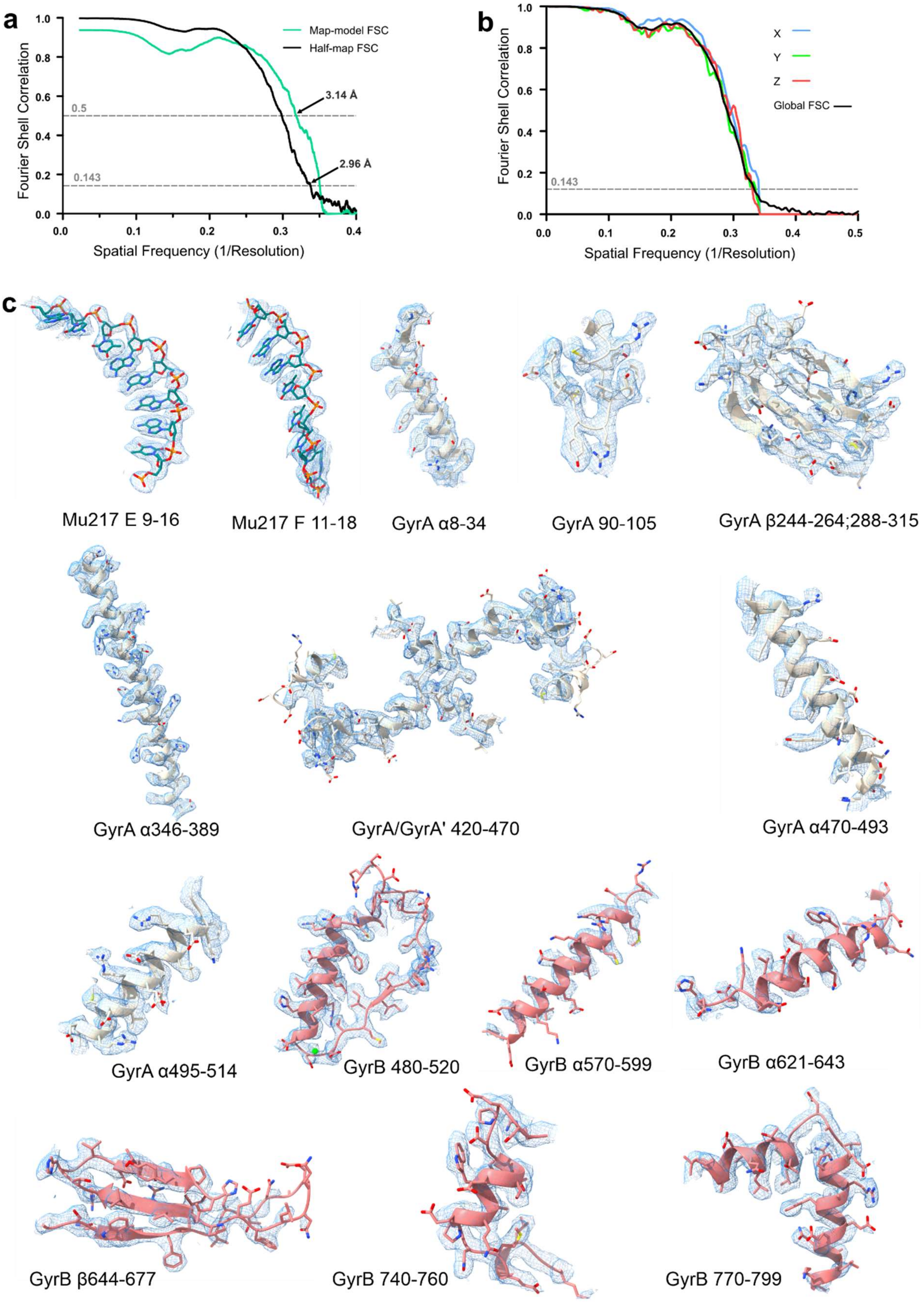


**Supplementary Figure 7. Validation of Gyr-Mu217-LEI800 cryo-EM reconstruction**. **a,** Map-to-model FSC curve (green) compared with half-maps FSC curve (black) as output by Phenix (soft mask based on the atomic model is used). FSC=0.5 (for map-to-model FSC) and FSC=0.143 (for half-map FSC) values are indicated with arrows. **b,** a 3D-FSC anisotropy plot as output by the 3DFSC processing server.[7] The global FSC curve is represented in black.

The directional FSCs along the x, y and z axes are displayed in blue, green and pink, respectively. The reported sphericity value was 0.965/1. **c,** density fits for representative regions of the model (α-helices, β-sheets, DNA nucleotides, loops) along GyrA and GyrB proteins. GyrA and GyrB are shown as beige and coral cartoon representations respectively; cryo-EM density as blue mesh.

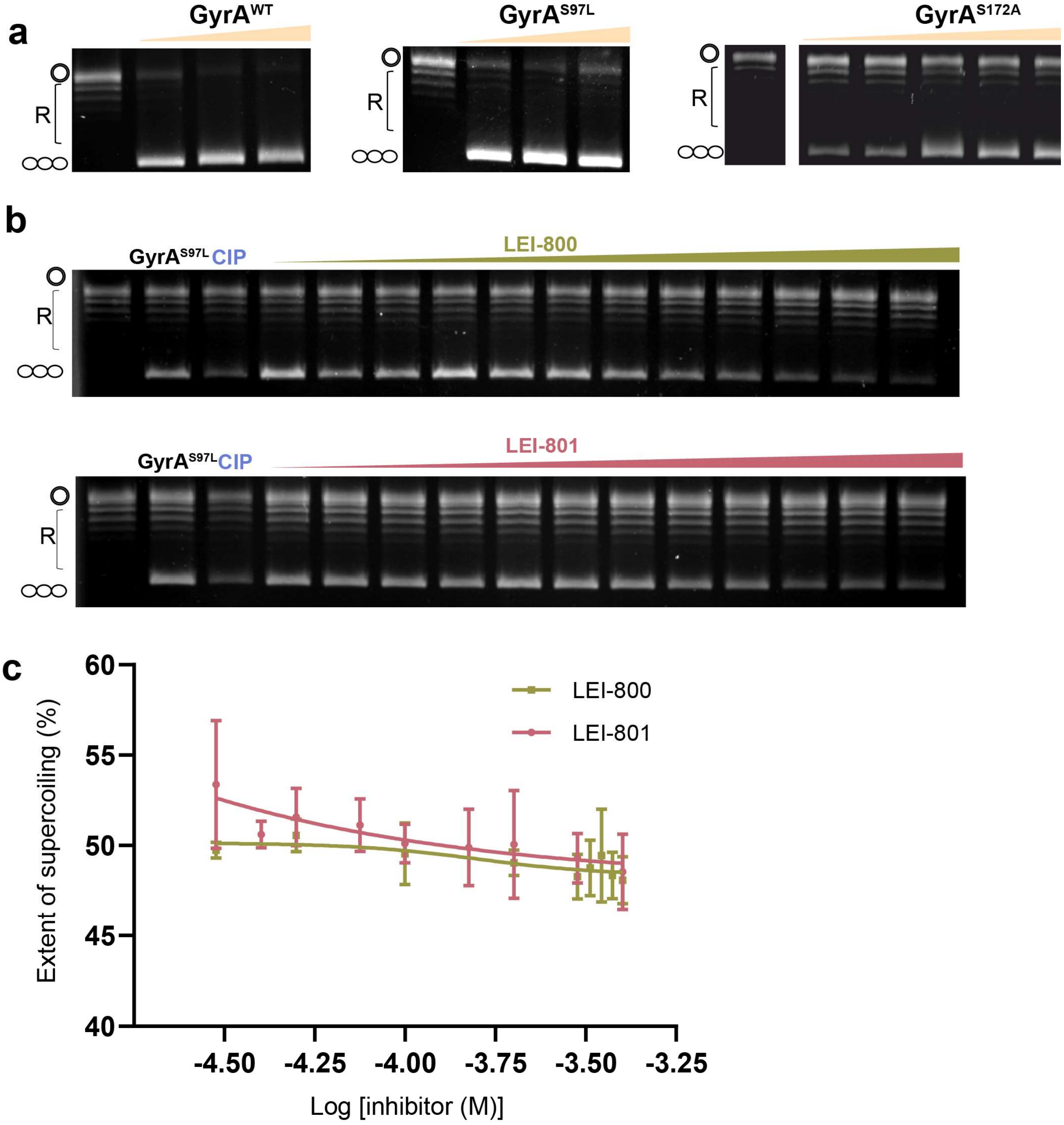


**Supplementary Figure 8. Activity of LEI-800 and LEI-801 against reconstituted EcGyrA$^{S97A}$/GyrB complex. a,** Plasmid supercoiling assays showing the activity of WT ($GyrA_2B_2$) gyrase and GyrA$^{S97L}$ and GyrA$^{S172A}$ variants produced in this work. First lane: relaxed pBR322, subsequent lanes: effect of increasing enzyme concentration (5, 10, 15 nM) on relaxed plasmid. Positions of nicked, relaxed and supercoiled DNA are indicated to the left of each gel. In GyrA$^{S172A}$ assay higher enzyme concentrations were used (15, 20, 25, 30, 40, 50 nM). **b,** Plasmid supercoiling assay showing the inhibitory activity of LEI-800 and LEI-801 against GyrA$^{S97A}$/GyrB complex. First lane: relaxed pBR322, second lane: relaxed pBR322 with 25 nM reconstituted ($A_2B_2$) GyrA$^{S97A}$/GyrB, third lane: effect of 10 µM ciprofloxacin (CIP) on gyrase activity, subsequent lanes: effect of increasing compound on GyrA$^{S97A}$/GyrB complex activity. Positions of nicked, relaxed and supercoiled DNA are indicated to the left of each gel. When absent, compound was replaced with the corresponding amount of DMSO. Concentrations used for LEI-800: 30, 50, 100, 200, 300, 325, 350, 375, 400; for LEI-801: 30, 40, 50, 75, 100, 150, 200, 300, 400. **c,** Dose-response curves of DNA gyrase supercoiling inhibition. Values are plotted as mean values ± SD (n = 3 independent biochemical experiments).

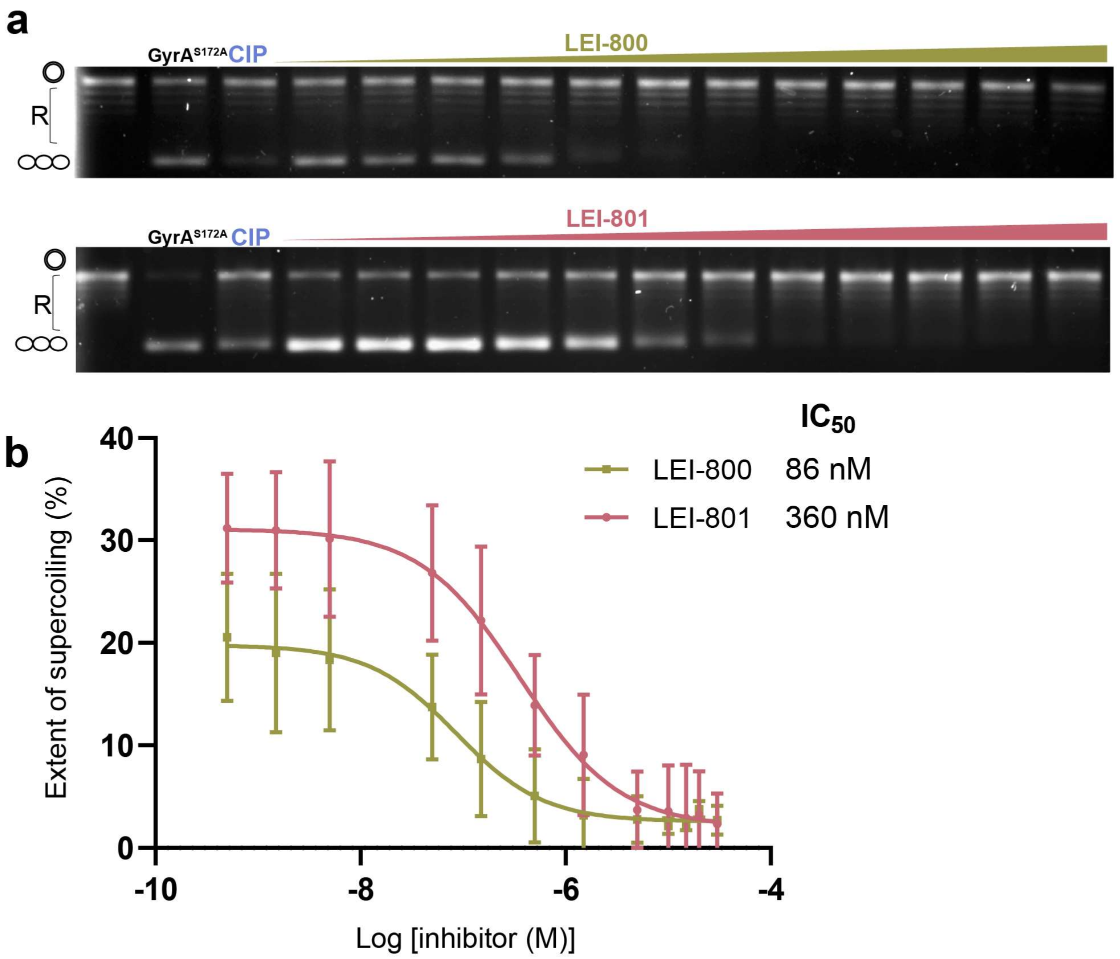


**Supplementary Figure 9. Activity of LEI-800, LEI-801 and compound 60 against reconstituted EcGyrA$^{S172A}$/GyrB complex. a,** Plasmid supercoiling assay showing the inhibitory activity of LEI-800 and LEI-801 against GyrA$^{S172A}$/GyrB complex. First lane: relaxed pBR322, second lane: relaxed pBR322 with 30 nM reconstituted ($A_2B_2$) GyrA$^{S172A}$/GyrB gyrase complex, third lane: effect of 10 µM ciprofloxacin (CIP) on gyrase activity, subsequent lanes: effect of increasing compound concentration (0.0005, 0.0015, 0.005, 0.05, 0.15, 0.5, 1.5, 5, 10, 15, 20, 30 µM) on GyrA$^{S172A}$/GyrB complex activity. Positions of nicked, relaxed and supercoiled DNA are indicated to the left of each gel. When absent, compound was replaced with the corresponding amount of DMSO. **b,** Dose-response curves of DNA gyrase supercoiling inhibition, based on n = 3 independent biochemical experiments. Mean values are plotted, with error bars representing the standard deviation of the mean at each concentration.

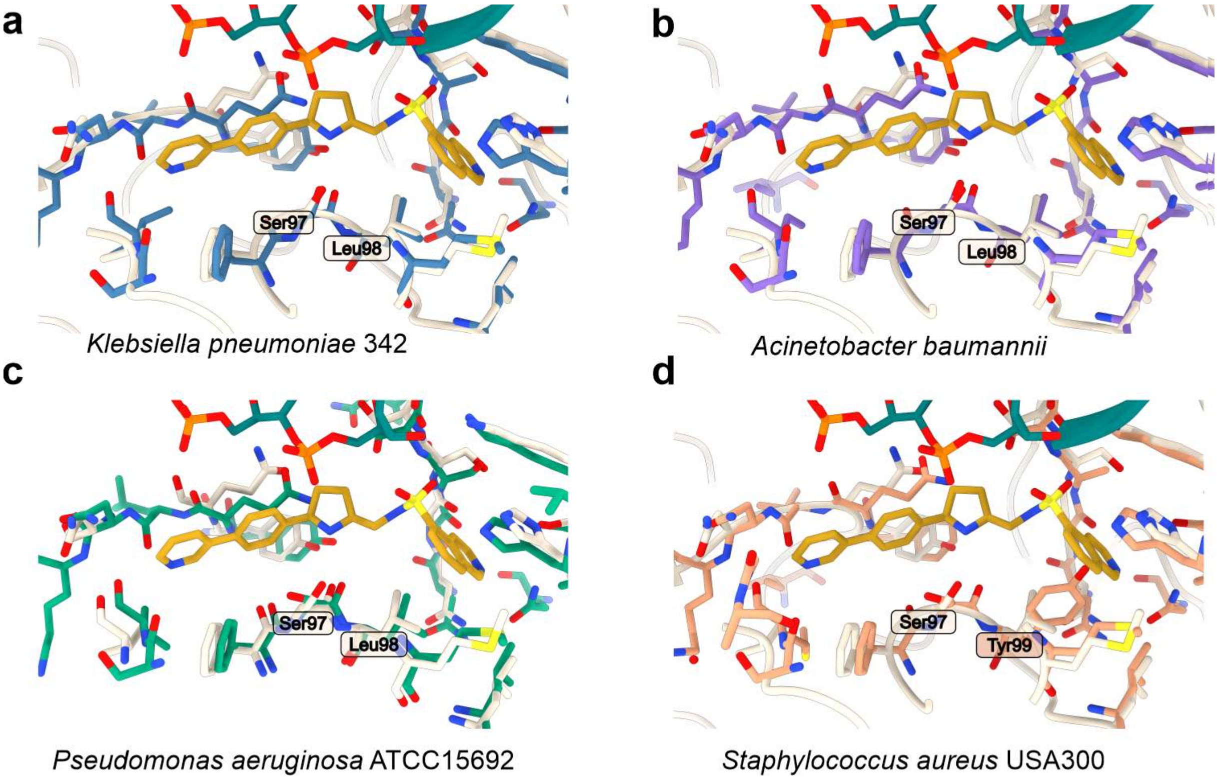


**Supplementary Figure 10. Comparison of LEI-800 binding pocket between Gram-negative and Gram-positive species. a,** Comparison of LEI800 binding pocket in *E. coli* (this paper, GyrA coloured beige, DNA coloured teal) and AlphaFold generated model of *K. pneumoniae* 312(UniProt: B5XNZ4_KLEP3; AF-DB: B5XNZ4) GyrA (blue). Ser97 and Leu98 are indicated. No significant differences are found in the neighbouring residues. **b,** Comparison of LEI800 binding pocket in *E. coli* and AlphaFold generated model of *A. baumanii* GyrA (UniProt: GYRA_ACIBA; AF-DB: Q2FCU6) coloured purple. No significant differences are found around the binding pocket. **c,** Comparison of LEI800 binding pocket in *E. coli* and AlphaFold generated model of GyrA from *P. aeruginosa* ATCC15692 (GYRA_PSEAE; AF-DB: P48372) coloured green. No significant differences are found around the binding pocket. **d,** Comparison of LEI800 binding pocket in *E. coli* and an AlphaFold generated model of *S. aureus* USA300 GyrA (UniProt: GYRA_STAA3; AF-DB: Q2FKQ0) coloured tan. Note Leu98->Tyr99 change (Ser97 is conserved) that might prevent LEI-800 activity against Gram-positive gyrase. For all comparisons, models were superimposed using ChimeraX *matchmaker* function.

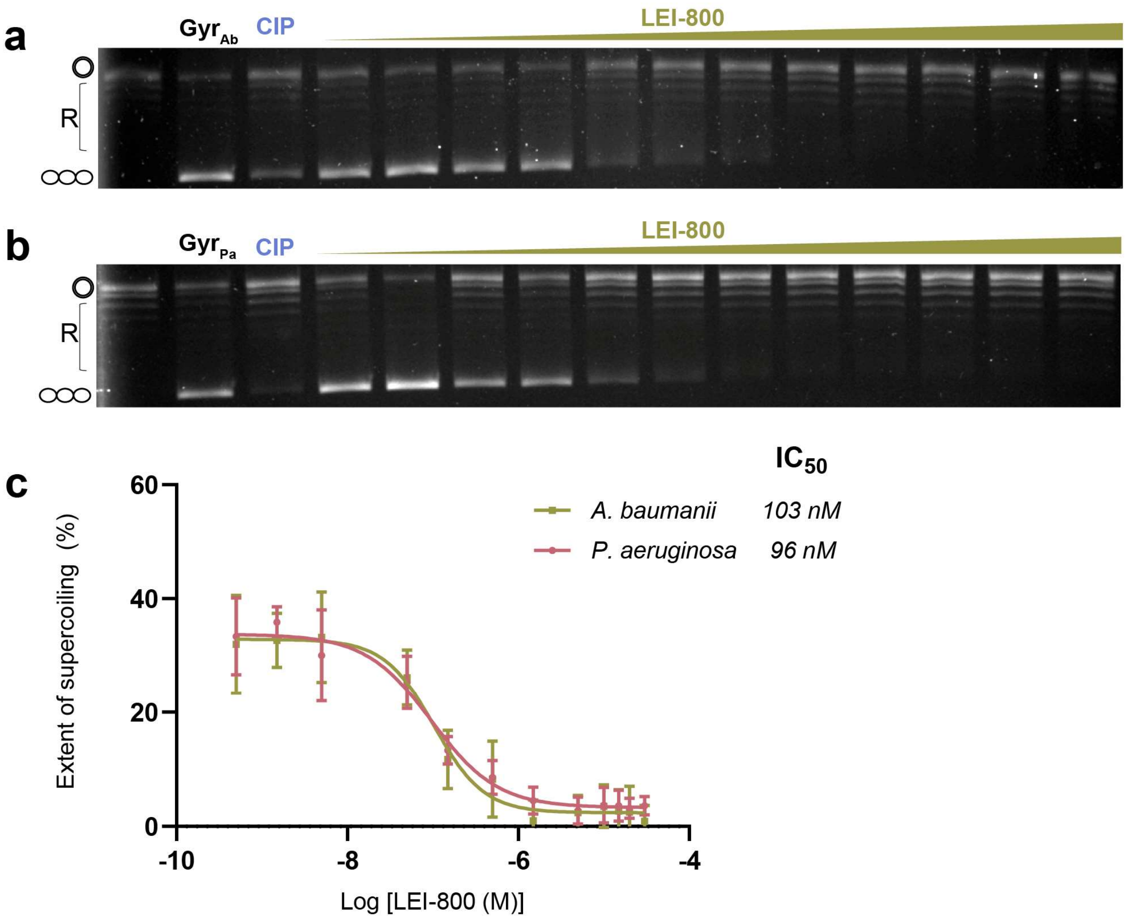


**Supplementary Figure 11. Activity of LEI-800 against purified *A. baumannii* and *P. aeuruginosa* gyrases. a,** Plasmid supercoiling assay showing the inhibitory activity of LEI-800 against *A. baumannii* gyrase. First lane: relaxed pBR322, second lane: relaxed pBR322 with 5 U *A. baumannii* gyrase, third lane: effect of 10 µM ciprofloxacin (CIP) on WT gyrase (5 U) activity, subsequent lanes: effect of increasing LEI-800 concentration (0.0005, 0.0015, 0.005, 0.05, 0.15, 0.5, 1.5, 5, 10, 15, 20, 30 µM) on WT gyrase activity. Positions of nicked, relaxed and supercoiled DNA are indicated to the left of each gel. When absent, compound was replaced with the corresponding amount of DMSO. **b,** Plasmid supercoiling assay showing the inhibitory activity of LEI-800 against *P. aeruginosa* gyrase. First lane: relaxed pBR322, second lane: relaxed pBR322 with 5 U *P. aeruginosa* gyrase, third lane: effect of 10 µM ciprofloxacin (CIP) on WT gyrase (5 U) activity, subsequent lanes: effect of increasing LEI-800 concentration (0.0005, 0.0015, 0.005, 0.05, 0.15, 0.5, 1.5, 5, 10, 15, 20, 30 µM) on WT gyrase activity. Positions of nicked, relaxed and supercoiled DNA are indicated to the left of each gel. When absent, compound was replaced with the corresponding amount of DMSO. **c,** Dose-response curves of DNA gyrase supercoiling inhibition, based on n = 3 independent biochemical experiments. Mean values are plotted, with the error bars representing the standard deviation of the mean at each concentration.

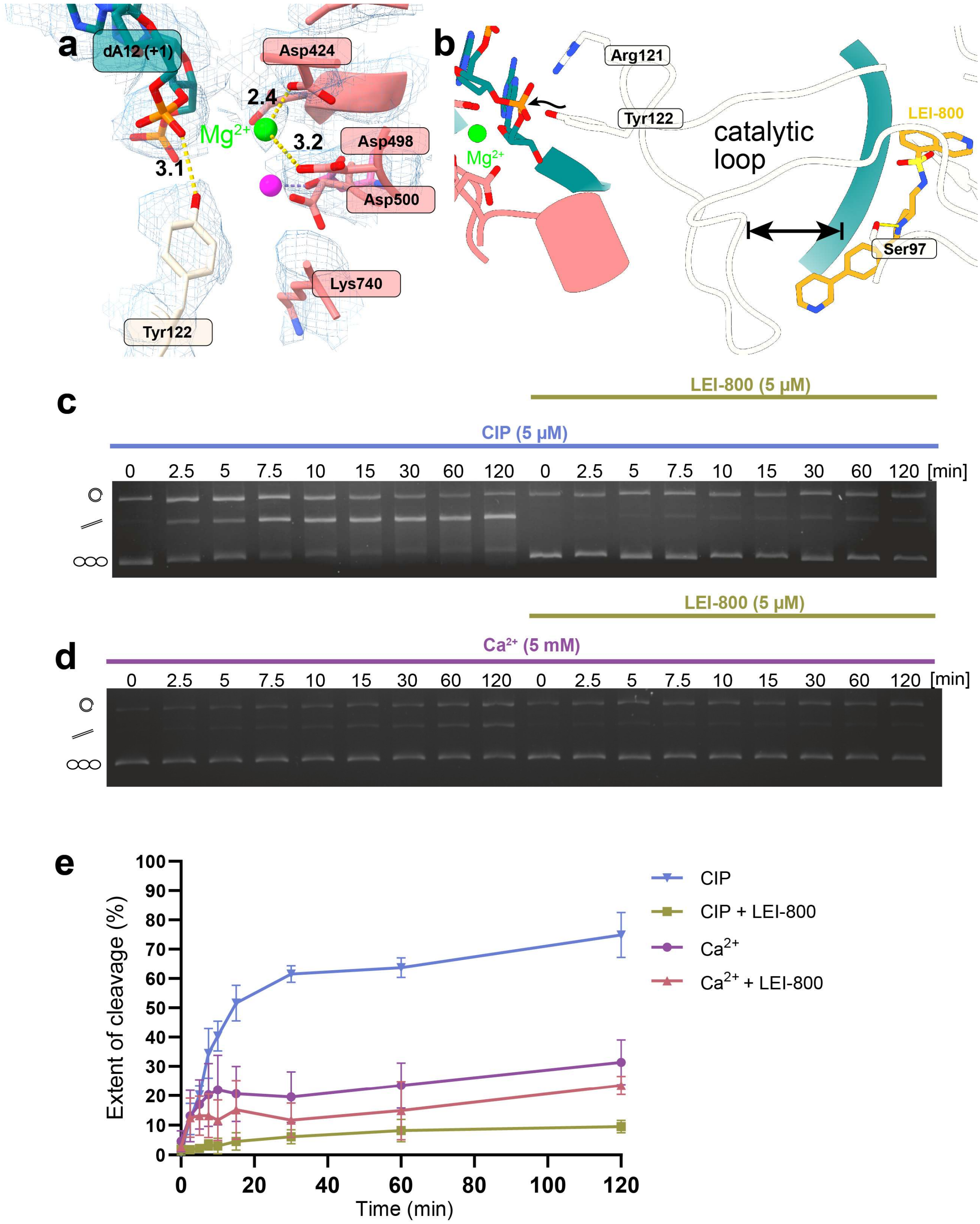


**Supplementary Figure 12. LEI-800 is an allosteric DNA cleavage inhibitor. a**, Metal binding site in Gyr-Mu217-LEI-800 complex. Catalytic tyrosine (Tyr122), scissile phosphate (+1), and neighbouring GyrB residues (within 6 Å distance from the metal, shown as lime sphere) are shown as stick representations. GyrA is coloured beige, GyrB – coral, DNA-teal. Map density is shown as blue mesh and is contoured at 9σ level. Distances in Å between the catalytic tyrosine and phosphate and between the metal and the closest Glu side chains are indicated. To illustrate metal movement after DNA cleavage (from 'A' to 'B' configuration), comparable structure with cleaved DNA (PDB:7Z9C) is superimposed, and metal ion shown as magenta sphere. **b,** proposed LEI-800 allosteric mechanism. a cartoon representation of a part of the Gyr-Mu217-LEI800 model. LEI-800 is shown as golden stick representation, GyrA as beige cartoon, GyrB as coral cartoon, DNA as teal. Catalytic residues, Ser97 and scissile phosphate are indicated. We propose that binding of LEI-800 hinders loop movement preventing formation of a phosphotyrosine bond and DNA cleavage, inhibiting the enzyme. **c,** A time-course of DNA cleavage by ciprofloxacin and effect of LEI-800. The reactions contained 5 µM ciprofloxacin and 5

µM LEI-800 (as indicated). After completion, the reactions were run on a gel with EtBr. The amount of cleaved DNA is reduced when LEI-800 is present. present. **d,** A time-course of DNA cleavage by $Ca^{2+}$ and effect of LEI-800. The reactions contained 5 mM $Ca^{2+}$and 5 µM LEI-800 (as indicated). After completion, the reactions were run on a gel with EtBr. The amount of cleaved DNA is reduced when LEI-800 is present. **e,** Linear DNA was quantified and mean values plotted based on n = 3 independent biochemical experiments. Error bars represent the SD of the mean.

## Synthetic procedures

### General remarks

All chemicals (Sigma-Aldrich, Fluka, Acros, Merck, Combi-Blocks, Fluorochem, TCI) were used as received. All solvents used for reactions were of analytical grade. THF, $Et_2O$, DMF, ACN and DCM were dried over activated 4 Å molecular sieves, MeOH over 3 Å molecular sieves. $H_2O$ used in synthesis procedures was of Milli-Q-grade quality. Column chromatography was performed on silica gel (Screening Devices BV, 40-63 µm, 60 Å). The eluent EtOAc was of technical grade and distilled before use. Triethylamine was distilled over KOH, and triethylamine and pyridine were stored over KOH pellets. Starting materials were coevaporated with toluene (3×) before use in water-sensitive reactions.

Reactions were monitored by thin layer chromatography (TLC) analysis using Merck aluminium sheets (Silica gel 60, F254). Compounds were visualized by UV-absorption (254 nm) and spraying for general compounds: $KMnO_4$ (20 g/L) and $K_2CO_3$ (10 g/L) in $H_2O$, or for amines: ninhydrin (0.75 g/L) and acetic acid (12.5 mL/L) in ethanol, followed by charring at 150°C. $^1H$ and $^{13}C$ NMR experiments were recorded on a Bruker AV-300 (300/75 MHz), Bruker AV-400 (400/101 MHz), Bruker DMX-400 (400/101 MHz), Bruker AV- 500 (500/126 MHz) and Bruker AV-600 (600/151 MHz). Chemical shifts are given in ppm (δ) relative to tetramethylsilane, as internal standard. Multiplicity: s = singlet, bs = broad singlet, d = doublet, dd = doublet of doublet, t = triplet, q = quartet, quint = quintet, non = nonet m = multiplet. Coupling constants (*J*) are given in Hz. LC-MS measurements were performed on a Thermo Finnigan LCQ Advantage MAX ion-trap mass spectrometer (ESI+) coupled to a Surveyor HPLC system (Thermo Finnigan) equipped with a standard C18 (Gemini, 4.6 mm D x 50 mm L, 5 µm particle size, Phenomenex) analytical column and buffers A: $H_2O$, B: ACN, C: 0.1% aq. TFA. High resolution mass spectra were recorded on a LTQ Orbitrap (Thermo Finnigan) mass spectrometer or a Synapt G2-Si high definition mass spectrometer (Waters) equipped with an electrospray ion source in positive mode (source voltage 3.5 kV, sheath gas flow 10 mL/min, capillary temperature 250°C) with resolution R = 60000 at m/z 400 (mass range m/z = 150-2000) and dioctylphtalate (m/z = 391.28428) as a lock mass. Preparative HPLC was performed on a Waters Acquity Ultra Performance LC with a C18 column (Gemini, 150 x 21.2 mm, Phenomenex) using a ACN in $H_2O$ (+0.2% TFA) gradient. All final compounds were determined to be > 95% pure by LC-UV analysis.

### General procedure A: Boc deprotection

The Boc protected compound (1 equiv.) was dissolved in DCM (0.1 M). TFA was added dropwise (17% v/v) at 0°C and the mixture was allowed to steer at room temperature for 4 h. The reaction was quenched with sat. aq. $Na_2CO_3$, diluted with water and extracted with DCM (3×). The combined organic layers were dried with $MgSO_4$, filtered and concentrated *in vacuo*. Purification of the crude material by column chromatography (0% → 10% MeOH (10% aq. $NH_3$) in DCM) afforded the pure product.

### General procedure B: sulfonation with sulfonyl chloride

A solution of amine (1 equiv.) and triethylamine (2 equiv.) in DCM (0.1 M) was cooled to 0°C after which dropwise a solution of the corresponding sulfonyl chloride (1.5 equiv.) in DCM (0.1 M) was added. The reaction mixture was allowed to warm to room temperature and stirred for 1 h before sat. aq. $Na_2CO_3$ was added. The mixture was extracted with DCM (2×), and the combined organic layers were dried over $Mg_2SO_4$, filtrated and concentrated *in vacuo*. The residue was purified by column chromatography (2% → 5% MeOH (10% aq. $NH_3$) in DCM) to afford the product.

### General procedure C: sulfonation with arylbromide

To a microwave reaction tube equipped with a magnetic stir bar was added potassium metabisulfite (2 equiv.), TBAB (1.2 equiv.), sodium formate (2.2 equiv.), palladium acetate (0.1 equiv.), triphenylphosphine (0.3 equiv.), 1,10-phenathroline (0.3 equiv.) and DMSO (0.25 M). The mixture was put under nitrogen flow for 10 min before the corresponding bromide (1 equiv.) was added. After that, the reaction vessel was immersed in a 70°C

preheated heating block for 4 h. After cooling, DIPEA (1.5 equiv.) the amine (1.5 equiv.) and THF (0.5 M) were added to the reaction mixture. Subsequently, a solution of *N*-chlorosuccinimide (2 equiv.) in THF (0.5 M) was added, the reaction was stirred at room temperature overnight. The mixture was then diluted with water, extracted with ethyl acetate. The organic layer was washed with brine and dried over $Na_2SO_4$. After filtration, the filtrate was concentrated under reduced pressure. The crude product was purified by column chromatography (2% → 5% MeOH (10% aq. $NH_3$) in DCM).

**General procedure D: Suzuki-Miyaura cross-coupling**

The bromobenzyl compound (1 equiv.) was reacted with the corresponding boronic acid (1.2 equiv.), $Pd(PPh_3)_4$ (0.015 equiv.), $K_2CO_3$ (4 equiv.) in 1,4-dioxane and water (1:3, 0.1 M) in a sealed microwave tube. The reaction mixture was degassed with under nitrogen flow for 15 min and then stirred overnight at 90°C. The reaction mixture was then filtered over a silica gel pad with EtOAc and concentrated *in vacuo*. The crude product was purified by column chromatography (1% → 10% MeOH (10% aq. $NH_3$) in DCM) to give the pure product.

**General procedure E: TIPS protection**

The prolinol was co-evaporated *in vacuo* twice with toluene. The prolinol (1 equiv.), imidazole (1.5 equiv.) and DMAP (0.05 equiv.) were dissolved in dry DCM (0.5 M). TIPS-Cl was added dropwise at 0°C after which the reaction mixture was allowed to warm to RT and was stirred for 16 h. The mixture was then poured onto sat. aq. $NH_4Cl$ and extracted with DCM (4×). The combined organic layers were washed with brine (2×), dried with $MgSO_4$, filtered and concentrated *in vacuo*. Purification of the crude material by column chromatography (80% → 100% $Et_2O$ in pentane with 1% triethylamine) afforded the pure product.

**General procedure F: pyrrolidine α-arylation**

The amine (1 equiv.) and benzophenone (1.2 equiv.) were combined and co-evaporated *in vacuo* twice with toluene. These were then dissolved in dry $Et_2O$ (0.5 M) and transferred to a flame dried flask under argon atmosphere. *n*-Butyllithium (1 equiv.) was added dropwise at -78°C and the solution was steered for 10 min. Subsequently, the corresponding aryllithium solution (1.5 equiv, prepared according to general procedure G) was dropwise added at -78°C after which the mixture was removed from the cooling bath and left to stir for 2 h while reaching room temperature. The mixture was then quenched with MeOH at -78°C and diluted with $Et_2O$. This was then washed with water and brine. The aqueous layer was extracted with $Et_2O$ (3×) and the combined organic layers were dried with $MgSO_4$, filtered and concentrated *in vacuo*. Purification of the crude material by column chromatography (2% → 20% $Et_2O$ in pentane with 1% triethylamine and 10% toluene) afforded the separate stereoisomers as crude products.

**General procedure G: preparation of aryllithium**

1,4-Dibromobenzene (1.5 equiv. to amine general procedure F) was co-evaporated *in vacuo* twice with toluene. This was then dissolved in dry $Et_2O$ (0.75 M) and transferred to a flame dried flask under argon atmosphere. *n*-butyllithium (1.5 equiv. to amine) was added dropwise at -78°C and the mixture was steered for 10 min. The mixture was then warmed to room temperature and steered for an additional 30 min before addition to the appropriate solution with imine intermediate.

**General procedure H: Boc protection**

The crude product of the pyrrolidine alkylation reaction (1 equiv.) was dissolved with di-*tert*-butyl dicarbonate (2.5 equiv.) and triethylamine (2 equiv.) in DCM (0.06 M) and the mixture was steered at room temperature. Upon completion, the reaction was diluted with DCM and washed with water and brine. The organic layer was dried with $MgSO_4$, filtered and concentrated *in vacuo*. Purification of the crude material by column chromatography (1% → 3% EtOAc in pentane) afforded the pure product.

**General procedure I: TIPS deprotection**

The TIPS protected starting material (1 equiv.) was dissolved in ACN (0.09 M). To this was added TBAF (5 equiv., 1 M in THF) and the mixture was steered at room temperature. The mixture was subsequently concentrated *in vacuo* on silica. Purification of the crude material by column chromatography (5% → 40% EtOAc in pentane) afforded the pure product.

**General procedure J: mesylation**

The primary alcohol (1 equiv.) and triethylamine (3 equiv.) were dissolved in dry DCM (0.05 M). A solution of MsCl (1.5 equiv.) in dry DCM (0.05 M) was added dropwise at 0°C and the mixture was steered at room temperature for 1.5 h. The mixture was quenched with the addition of water and extracted with DCM (3×). The combined organic layers were washed with brine, dried with $MgSO_4$, filtered and concentrated *in vacuo*. After characterization, the crude product was immediately used for further reactions.

**General procedure K: azide substitution**

The crude mesylated starting material (1 equiv.) and sodium azide (6 equiv.) were dissolved in dry DMF (0.1 M). The mixture was heated to 65°C and left to steer overnight. The reaction was diluted with $Et_2O$ and washed with water (3×) and brine. The organic layer was dried with $MgSO_4$, filtered and concentrated *in vacuo*. Purification of the crude material by column chromatography (15% → 20% EtOAc in pentane) afforded the pure product.

**General procedure L: Staudinger reduction of azide**

The azide compound (1 equiv.), $(Ph)_3P$ (2 equiv.) and water (2 equiv.) were dissolved in THF (0.1 M). The mixture was then refluxed at 60°C for 64 h. The reaction mixture was concentrated *in vacuo* and redissolved in $Et_2O$. This solution was thereafter extracted with 1 M aq. HCl (2×) and the aqueous layer washed with $Et_2O$ (2×). The pH of the aqueous layer was then adjusted to >12 with 2 M aq. NaOH and extracted with DCM (6×). The combined organic layers were dried with $MgSO_4$, filtered and concentrated *in vacuo*. Purification of the crude material by column chromatography (5% → 50% MeOH (10% aq. $NH_3$) in EtOAc) afforded the pure product.

**Compound characterization**

***N*-(2-((4-(Pyridin-3-yl)benzyl)amino)ethyl)isoquinoline-5-sulfonamide (1)**

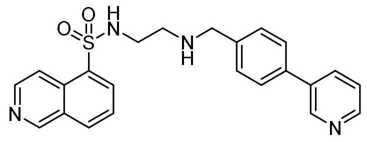

**1** (12 mg, 29 µmol, 62%) was synthesized from **109** (20 mg, 38 µmol) according to general procedure A.

$^1$H NMR (400 MHz, $CDCl_3$) δ 9.33 (d, *J* = 1.1 Hz, 1H), 8.79 (dd, *J* = 2.4, 0.9 Hz, 1H), 8.63 (d, *J* = 6.1 Hz, 1H), 8.58 (dd, *J* = 4.8, 1.6 Hz, 1H), 8.50 – 8.42 (m, 2H), 8.17 (dt, *J* = 8.4, 1.1 Hz, 1H), 7.85 (ddd, *J* = 7.9, 2.4, 1.6 Hz, 1H), 7.68 (dd, *J* = 8.2, 7.3 Hz, 1H), 7.45 (d, *J* = 8.6 Hz, 2H), 7.37 (ddd, *J* = 7.9, 4.8, 0.9 Hz, 1H), 7.22 (d, *J* = 8.6 Hz, 2H), 3.59 (s, 2H), 3.04 (t, *J* = 5.7 Hz, 2H), 2.69 (t, *J* = 6.1 Hz, 2H).

$^{13}$C NMR (101 MHz, $CDCl_3$) δ 153.37, 148.40, 148.10, 145.14, 139.67, 136.58, 136.30, 134.46, 134.41, 133.55, 133.35, 131.31, 129.08, 128.69, 127.19, 126.01, 123.74, 117.33, 52.78, 47.60, 42.56.

HRMS $[C_{23}H_{22}N_4O_2S +H]^+$: 419.15335 calculated, 419.15335 found.

***N*-(2-((4-(Pyridin-3-yl)benzyl)amino)ethyl)naphthalene-1-sulfonamide (47)**

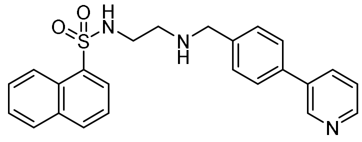

**47** (50 mg, 39 µmol, 53% over two steps) was synthesized from **108** (24 mg, 73 µmol) and naphtalene 1-sulfonyl chloride (25 mg, 0.11 mmol) according to general procedure B, followed by general procedure A.

$^1$H NMR (400 MHz, $CDCl_3$) δ 8.81 (dd, *J* = 2.4, 0.9 Hz, 1H), 8.68 (dt, *J* = 8.6, 1.0 Hz, 1H), 8.59 (dd, *J* = 4.8, 1.6 Hz, 1H), 8.28 (dd, *J* = 7.3, 1.3 Hz, 1H), 8.06 (dt, *J* = 8.2, 1.1 Hz, 1H), 7.97 – 7.91 (m, 1H), 7.85 (ddd, *J* = 7.9, 2.4, 1.6 Hz, 1H), 7.69 – 7.50 (m, 3H), 7.48 – 7.42 (m, 2H), 7.37 (ddd, *J* = 7.9, 4.8, 0.9 Hz, 1H), 7.23 – 7.16 (m, 2H), 3.52 (s, 2H), 3.02 – 2.94 (m, 2H), 2.67 – 2.58 (m, 2H).

$^{13}$C NMR (101 MHz, $CDCl_3$) δ 148.55, 148.30, 139.77, 136.70, 136.40, 134.53, 134.39, 134.36, 129.91, 129.27, 128.76, 128.50, 128.26, 127.24, 127.00, 124.46, 124.31, 123.72, 52.73, 47.37, 42.60.

HRMS $[C_{24}H_{23}N_3O_2S+H]^+$: 418.15837 calculated, 418.15767 found.

***N*-(2-((4-(Pyridin-3-yl)benzyl)amino)ethyl)benzenesulfonamide (48)**

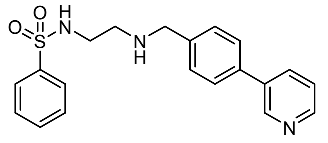

**108** (24 mg, 73 µmol) and benzenesulfonyl chloride (19 mg, 0.11 mmol) were reacted according to general procedure B, followed by general procedure A to yield title compound **48** (8.8 mg, 24 µmol, 33% over two steps).

$^1$H NMR (400 MHz, $CDCl_3$) δ 8.83 (dd, *J* = 2.4, 0.9 Hz, 1H), 8.59 (dd, *J* = 4.8, 1.6 Hz, 1H), 7.91 – 7.83 (m, 3H), 7.61 – 7.46 (m, 5H), 7.41 – 7.31 (m, 3H), 3.72 (s, 2H), 3.08 – 3.02 (m, 2H), 2.79 – 2.71 (m, 2H).

$^{13}$C NMR (101 MHz, $CDCl_3$) δ 148.55, 148.29, 139.89, 139.85, 136.81, 136.39, 134.41, 132.73, 129.22, 128.91, 127.35, 127.16, 123.73, 52.90, 47.58, 42.56.

HRMS $[C_{20}H_{21}N_3O_2S +H]^+$: 368.14272 calculated, 368.14201 found.

***N*-(2-((4-(Pyridin-3-yl)benzyl)amino)ethyl)-1H-indazole-4-sulfonamide (49)**

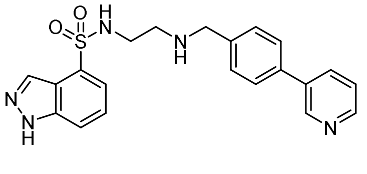

**49** (8.0 mg, 20 µmol, 13% over two steps) was synthesized from **108** (50 mg, 0.15 mmol) and 4-bromo-1*H*-indazole (25 mg, 0.12 mmol) according to general procedure C followed by general procedure A.

$^{1}H$ NMR (400 MHz, MeOD) δ 8.91 (d, *J* = 2.3 Hz, 1H), 8.63 (dd, *J* = 5.1, 1.5 Hz, 1H), 8.41 (d, *J* = 1.0 Hz, 1H), 8.30 (dt, *J* = 8.2, 1.9 Hz, 1H), 7.88 (d, *J* = 8.4 Hz, 1H), 7.85 – 7.80 (m, 3H), 7.72 (d, *J* = 7.1 Hz, 1H), 7.69 – 7.64 (m, 2H), 7.57 (dd, *J* = 8.5, 7.2 Hz, 1H), 4.34 (s, 2H), 3.23 – 3.17 (m, 4H).

HRMS $[C_{21}H_{21}N_5O_2S+H]^+$: 408.14887 calculated, 408.14866 found

***N*-(2-((4-(Pyridin-3-yl)benzyl)amino)ethyl)-1H-indazole-6-sulfonamide (50)**

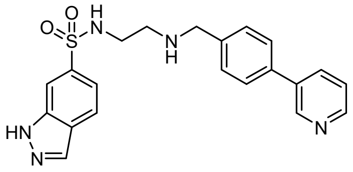

**50** (5.3 mg, 13 µmol, 9% over two steps) was synthesized from **108** (50 mg, 0.15 mmol) and 6-bromo-1*H*-indazole (47 mg, 0.24 mmol) according to general procedure C followed by general procedure A.

$^{1}H$ NMR (400 MHz, MeOD) δ 8.80 (dd, *J* = 2.4, 0.9 Hz, 1H), 8.51 (dd, *J* = 4.9, 1.6 Hz, 1H), 8.37 (d, *J* = 5.0 Hz, 1H), 8.10 (ddd, *J* = 8.0, 2.3, 1.6 Hz, 1H), 7.61 (dd, *J* = 6.0, 2.3 Hz, 3H), 7.55 – 7.48 (m, 2H), 7.37 – 7.30 (m, 2H), 6.92 (d, *J* = 3.5 Hz, 1H), 3.67 (s, 2H), 3.07 (t, *J* = 6.3 Hz, 2H), 2.62 (t, *J* = 6.3 Hz, 2H).

HRMS $[C_{21}H_{21}N_5O_2S+H]^+$: 408.14887 calculated, 408.14838 found.

***N*-Methyl-5-(*N*-(2-((4-(pyridin-3-yl)benzyl)amino)ethyl)sulfamoyl)picolinamide (51)**

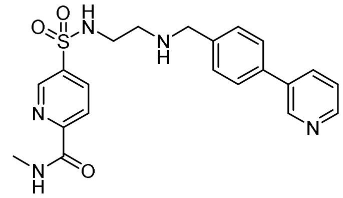

**51** (4.0 mg, 9.0 µmol, 6% over two steps) was synthesized from **108** (50 mg, 0.15 mmol) and 5-bromo-*N*-methylpicolinamide (52 mg, 0.24 mmol) according to general procedure C followed by general procedure A.

$^{1}H$ NMR (400 MHz, MeOD) δ 9.02 (dd, *J* = 2.3, 0.8 Hz, 1H), 8.80 (dd, *J* = 2.4, 0.9 Hz, 1H), 8.51 (dd, *J* = 4.9, 1.6 Hz, 1H), 8.36 (dd, *J* = 8.2, 2.3 Hz, 1H), 8.22 (dd, *J* = 8.2, 0.9 Hz, 1H), 8.10 (ddd, *J* = 8.0, 2.4, 1.6 Hz, 1H), 7.65 – 7.60 (m, 2H), 7.53 (ddd, *J* = 8.0, 4.9, 0.9 Hz, 1H), 7.44 – 7.38 (m, 2H), 7.24 – 7.09 (m, 2H), 3.77 (s, 2H), 3.09 (t, *J* = 6.4 Hz, 2H), 2.95 (s, 3H), 2.68 (t, *J* = 6.4 Hz, 2H).

HRMS $[C_{21}H_{23}N_5O_3S+H]^+$: 426.15944 calculated, 426.15879 found.

***N*-(2-((4-(Pyridin-3-yl)benzyl)amino)ethyl)quinazoline-6-sulfonamide (52)**

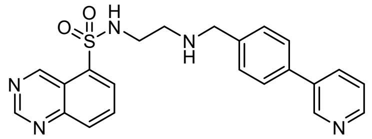

**52** (12 mg, 29 µmol, 19% over two steps) was synthesized from **108** (50 mg, 0.15 mmol) and 5-bromoquinazoline (25 mg, 0.12 mmol) according to general procedure C followed by general procedure A.

$^{1}H$ NMR (400 MHz, $CDCl_3$) δ 9.51 (d, *J* = 0.8 Hz, 1H), 9.45 (s, 1H), 8.82 (dd, *J* = 2.3, 0.9 Hz, 1H), 8.60 (dd, *J* = 4.8, 1.6 Hz, 1H), 8.55 (dd, *J* = 2.0, 0.6 Hz, 1H), 8.27 (dd, *J* = 8.9, 2.0 Hz, 1H), 8.18 – 8.13 (m, 1H), 7.86 (ddd, *J* = 7.9, 2.4, 1.6 Hz, 1H), 7.53 – 7.48 (m, 2H), 7.38 (ddd, *J* = 7.9, 4.8, 0.9 Hz, 1H), 7.35 – 7.30 (m, 2H), 3.73 (s, 2H), 3.17 – 3.06 (m, 2H), 2.83 – 2.72 (m, 2H).

$^{13}C$ NMR (101 MHz, $CDCl_3$) δ 161.48, 157.48, 151.46, 148.64, 148.30, 139.65, 139.50, 136.96, 136.27, 134.40, 130.89, 130.45, 128.83, 127.81, 127.40, 124.14, 123.75, 52.94, 47.60, 42.63.

HRMS $[C_{22}H_{21}N_5O_2S+H]^+$: 420.14887 calculated, 420.14850 found.

***N*-(2-((4-(Pyridin-3-yl)benzyl)amino)ethyl)quinazoline-7-sulfonamide (53)**

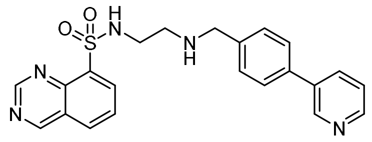

**108** (50 mg, 0.15 mmol) and 8-bromoquinazoline (27 g, 0.13 µmol) were reacted according to general procedure C, followed by general procedure A to yield title compound **53** (13 mg, 31 µmol, 5% over two steps).

$^{1}H$ NMR (400 MHz, MeOD) δ 9.62 (d, *J* = 0.9 Hz, 1H), 9.36 (s, 1H), 8.78 (dd, *J* = 2.4, 0.9 Hz, 1H), 8.54 – 8.48 (m, 2H), 8.31 (dd, *J* = 8.6, 0.7 Hz, 1H), 8.12 (dd, *J* = 8.6, 1.7 Hz, 1H), 8.08 (ddd, *J* = 8.0, 2.4, 1.6 Hz, 1H), 7.61 – 7.56 (m, 2H), 7.56 – 7.50 (m, 1H), 7.42 – 7.37 (m, 2H), 3.75 (s, 2H), 3.11 (t, *J* = 6.4 Hz, 2H), 2.70 (t, *J* = 6.4 Hz, 2H).

HRMS $[C_{22}H_{21}N_5O_2S+H]^+$: 420.14887 calculated, 420.14857 found.

***N*-(2-((4-(Pyridin-3-yl)benzyl)amino)ethyl)-1*H*-pyrrolo[2,3-b]pyridine-4-sulfonamide (54)**

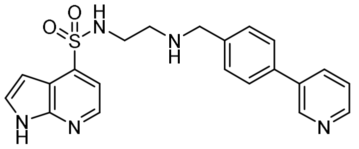

**108** (50 mg, 0.15 mmol) and 4-bromo-7-azaindole (47 mg, 0.24 mmol) were reacted according to general procedure C, followed by general procedure A to yield title compound **54** (1.8 mg, 4.6 µmol, 3% over two steps).

$^1H$ NMR (400 MHz, MeOD) δ 8.97 (s, 1H), 8.67 (d, *J* = 5.0 Hz, 1H), 8.41 (d, *J* = 5.0 Hz, 2H), 7.85 (d, *J* = 8.2 Hz, 2H), 7.76 (d, *J* = 6.1 Hz, 1H), 7.71 – 7.62 (m, 3H), 7.55 (d, *J* = 5.0 Hz, 1H), 6.93 (d, *J* = 3.5 Hz, 1H), 4.34 (s, 2H), 3.25 – 3.16 (m, 4H).

HRMS $[C_{21}H_{21}N_5O_2S+H]^+$: 408.14887 calculated, 408.14856 found.

**3-Amino-*N*-(2-((4-(pyridin-3-yl)benzyl)amino)ethyl)isoquinoline-5-sulfonamide (55)**

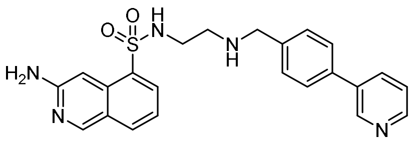

**108** (50 mg, 0.15 mmol) and 5-bromoisoquinolin-3-amine (54 g, 0.24 mmol) were reacted according to general procedure C, followed by general procedure A to yield title compound **55** (5.3 mg, 12 µmol, 8% over two steps).

$^1H$ NMR (400 MHz, MeOD) δ 8.86 (d, *J* = 0.9 Hz, 1H), 8.79 (dd, *J* = 2.3, 0.8 Hz, 1H), 8.52 (dd, *J* = 4.9, 1.6 Hz, 1H), 8.20 (dd, *J* = 7.3, 1.3 Hz, 1H), 8.09 (dt, *J* = 8.0, 2.0 Hz, 1H), 8.03 (dt, *J* = 8.2, 1.2 Hz, 1H), 7.61 – 7.55 (m, 2H), 7.54 – 7.50 (m, 1H), 7.40 (d, *J* = 1.1 Hz, 1H), 7.32 – 7.05 (m, 5H), 3.59 (s, 2H), 3.02 (t, *J* = 6.2 Hz, 2H), 2.57 (t, *J* = 6.2 Hz, 2H).

$^{13}C$ NMR (101 MHz, MeOD) δ 154.08, 148.69, 148.27, 140.76, 138.36, 137.37, 136.45, 135.54, 135.39, 135.23, 132.96, 130.43, 130.19, 128.17, 125.50, 124.89, 121.58, 97.10, 53.45, 48.94, 43.08.

HRMS $[C_{23}H_{24}N_5O_2S+H]^+$: 434.16452 calculated, 434.16422 found.

***N*-(2-(([1,1'-Biphenyl]-4-ylmethyl)amino)ethyl)isoquinoline-5-sulfonamide (56)**

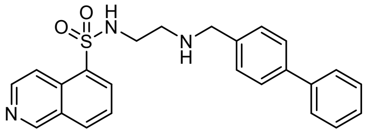

Phenylboronic acid (28 mg, 0.23 mmol) was subjected to general procedure D with **113a** (0.10 g, 0.19 mmol) followed by general procedure A to yield the title compound **56** (60 mg, 33 µmol, 17% over two steps).

$^1H$ NMR (400 MHz, $CDCl_3$) δ 9.32 (d, *J* = 1.0 Hz, 1H), 8.66 (d, *J* = 6.1 Hz, 1H), 8.44 (ddd, *J* = 7.4, 5.8, 1.1 Hz, 2H), 8.15 (dt, *J* = 8.2, 1.1 Hz, 1H), 7.66 (dd, *J* = 8.2, 7.3 Hz, 1H), 7.59 – 7.53 (m, 2H), 7.50 – 7.40 (m, 4H), 7.39 – 7.31 (m, 1H), 7.19 – 7.14 (m, 2H), 3.57 (s, 2H), 3.07 – 2.94 (m, 2H), 2.71 – 2.62 (m, 2H).

$^{13}C$ NMR (101 MHz, $CDCl_3$) δ 153.45, 145.31, 140.78, 140.23, 138.59, 134.36, 133.65, 133.42, 131.37, 129.12, 128.91, 128.44, 127.43, 127.28, 127.12, 126.01, 117.31, 52.86, 47.39, 42.52.

HRMS $[C_{24}H_{23}N_3O_2S+H]^+$: 418.15837 calculated, 418.15782 found.

***N*-(2-((4-(Thiophen-3-yl)benzyl)amino)ethyl)isoquinoline-5-sulfonamide (57)**

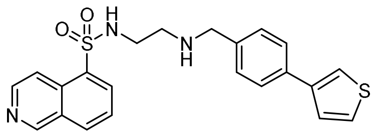

Thien-3-ylboronic acid (31 mg, 0.23 mmol) was subjected to general procedure D with **113a** (0.10 g, 0.19 mmol) followed by general procedure A to provide **57** (39 mg, 90 µmol, 47% over two steps).

$^1H$ NMR (400 MHz, $CDCl_3$) δ 9.33 (d, *J* = 1.0 Hz, 1H), 8.67 (d, *J* = 6.1 Hz, 1H), 8.45 – 8.41 (m, 2H), 8.16 (dt, *J* = 8.2, 1.1 Hz, 1H), 7.67 (dd, *J* = 8.2, 7.3 Hz, 1H), 7.51 – 7.45 (m, 2H), 7.43 (dd, *J* = 2.9, 1.4 Hz, 1H), 7.41 – 7.35 (m, 2H), 7.16 – 7.09 (m, 2H), 3.54 (s, 2H), 3.02 – 2.94 (m, 2H), 2.70 – 2.62 (m, 2H).

$^{13}C$ NMR (101 MHz, $CDCl_3$) δ 153.46, 145.34, 141.97, 138.42, 134.93, 134.31, 133.67, 133.45, 131.36, 129.13, 128.49, 126.59, 126.42, 126.36, 126.01, 120.35, 117.30, 52.89, 47.34, 42.50.

HRMS $[C_{22}H_{21}N_3O_2S_2+H]^+$: 424.11479 calculated, 424.11473 found.

***N*-(2-((4-(Furan-3-yl)benzyl)amino)ethyl)isoquinoline-5-sulfonamide (58)**

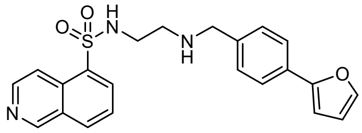


Furan-3-ylboronic acid (26 mg, 0.23 mmol) was subjected to general procedure D with **113a** (0.10 g, 0.19 mmol) followed by general procedure A to provide **58** (6.0 mg, 15 µmol, 8% over two steps).

$^1H$ NMR (400 MHz, $CDCl_3$) δ 9.34 (d, *J* = 1.0 Hz, 1H), 8.68 (d, *J* = 6.1 Hz, 1H), 8.48 – 8.39 (m, 2H), 8.18 (dt, *J* = 8.4, 1.1 Hz, 1H), 7.72 (dd, *J* = 1.5, 0.9 Hz, 1H), 7.68 (dd, *J* = 8.2, 7.4 Hz, 1H), 7.48 (t, *J* = 1.7 Hz, 1H), 7.41 – 7.36 (m, 2H), 7.17 – 7.09 (m, 2H), 6.69 (dd, *J* = 1.9, 0.9 Hz, 1H), 3.56 (s, 2H), 3.04 – 2.95 (m, 2H), 2.71 – 2.62 (m, 2H).

$^{13}C$ NMR (101 MHz, $CDCl_3$) δ 153.50, 145.41, 143.85, 138.59, 138.33, 134.32, 133.68, 133.46, 131.52, 131.38, 129.14, 128.49, 126.18, 126.05, 126.01, 117.28, 108.91, 52.90, 47.26, 42.50.

HRMS $[C_{22}H_{21}N_3O_3S+H]^+$: 408.13764 calculated, 408.13725 found.

***N*-(2-(((4'-Fluoro-[1,1'-biphenyl]-4-yl)methyl)amino)ethyl)isoquinoline-5-sulfonamide (59)**

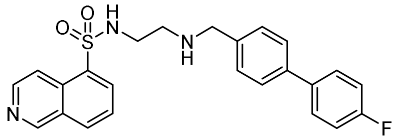


(4-Fluorophenyl)boronic acid (32 mg, 0.23 mmol) was subjected to general procedure D with **113a** (0.10 g, 0.19 mmol) followed by general procedure A to provide **59** (41 mg, 99 µmol, 52% over two steps).

$^1H$ NMR (400 MHz, $CDCl_3$) δ 9.33 (d, *J* = 0.9 Hz, 1H), 8.67 (d, *J* = 6.1 Hz, 1H), 8.47 – 8.41 (m, 2H), 8.17 (dt, *J* = 8.3, 1.2 Hz, 1H), 7.67 (dd, *J* = 8.2, 7.3 Hz, 1H), 7.55 – 7.48 (m, 2H), 7.46 – 7.40 (m, 2H), 7.20 – 7.08 (m, 4H), 3.57 (s, 2H), 3.03 – 2.97 (m, 2H), 2.70 – 2.65 (m, 2H).

$^{13}C$ NMR (101 MHz, $CDCl_3$) δ 162.57 (d, *J* = 243 Hz), 161.34, 153.46, 145.34, 139.27, 138.52, 136.91, 136.88, 134.31, 133.66, 133.44, 131.35, 129.12, 128.66 (d, *J* = 8.0 Hz), 128.52, 127.15, 126.01, 117.29, 115.76 (d, *J* = 23 Hz), 52.78, 47.35, 42.47.

HRMS $[C_{24}H_{22}FN_3O_2S+H]^+$: 436.14895 calculated, 436.14842 found.

***N*-(2-((4-(6-Fluoropyridin-3-yl)benzyl)amino)ethyl)isoquinoline-5-sulfonamide (60)**

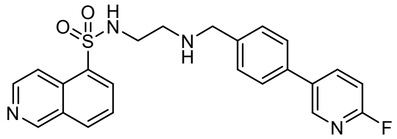


(6-Fluoropyridin-3-yl)boronic acid (32 mg, 0.23 mmol) was subjected to general procedure D with **113a** (0.10 g, 0.19 mmol) followed by general procedure A to provide **60** (26 mg, 60 µmol, 32% over two steps).

$^1H$ NMR (400 MHz, $CDCl_3$) δ 9.34 (d, *J* = 0.9 Hz, 1H), 8.67 (d, *J* = 6.1 Hz, 1H), 8.45 (dt, *J* = 7.1, 1.3 Hz, 2H), 8.39 (dt, *J* = 2.7, 0.9 Hz, 1H), 8.19 (dt, *J* = 8.2, 1.1 Hz, 1H), 7.95 (ddd, *J* = 8.5, 7.6, 2.6 Hz, 1H), 7.69 (dd, *J* = 8.2, 7.3 Hz, 1H), 7.46 – 7.41 (m, 2H), 7.26 – 7.21 (m, 2H), 7.01 (ddd, *J* = 8.5, 3.0, 0.7 Hz, 1H), 3.60 (s, 2H), 3.05 – 2.98 (m, 2H), 2.73 – 2.65 (m, 2H).

$^{13}C$ NMR (101 MHz, $CDCl_3$) δ 163.22 (d, *J* = 243 Hz), 153.47, 145.80 (d, *J* = 14.5 Hz) 145.34, 139.76 (d, *J* = 8.1 Hz), 139.69, 135.69, 134.52 (d, *J* = 4.5 Hz), 134.30, 133.69, 133.48, 131.35, 129.14, 128.79, 127.21, 126.04, 117.27, 109.61 (d, *J* = 23 Hz), 52.76, 47.41, 42.51.

HRMS $[C_{23}H_{21}FN_4O_2S+H]^+$: 437.14420 calculated, 437.14402 found.

**_N_-(2-(((4'-Chloro-[1,1'-biphenyl]-4-yl)methyl)amino)ethyl)isoquinoline-5-sulfonamide (61)**

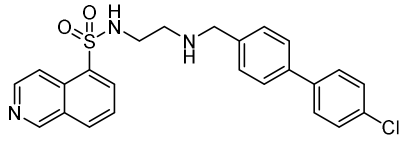

(4-Chlorophenyl)boronic acid (36 mg, 0.23 mmol) was subjected to general procedure D with **113a** (0.10 g, 0.19 mmol) followed by general procedure A to provide **61** (80 mg, 0.14 mmol, 74% over two steps).

$^1$H NMR (400 MHz, $CDCl_3$) δ 9.32 (d, *J* = 1.0 Hz, 1H), 8.68 – 8.62 (m, 1H), 8.49 – 8.41 (m, 2H), 8.20 – 8.11 (m, 1H), 7.70 – 7.50 (m, 2H), 7.50 – 7.35 (m, 6H), 7.24 – 7.14 (m, 2H), 3.59 (d, *J* = 6.9 Hz, 2H), 3.05 – 2.95 (m, 2H), 2.73 – 2.60 (m, 2H).

$^{13}$C NMR (101 MHz, $CDCl_3$) δ 153.42, 145.27, 139.17, 138.97, 138.73, 134.31, 133.65, 133.48, 133.41, 131.33, 129.10, 129.03, 128.60, 128.33, 127.09, 126.01, 117.31, 52.74, 47.40, 42.43.

HRMS $[C_{24}H_{22}ClN_3O_2S+H]^+$: 452.11940 calculated, 452.11913 found.

**_N_-(2-(((4'-Methyl-[1,1'-biphenyl]-4-yl)methyl)amino)ethyl)isoquinoline-5-sulfonamide (62)**

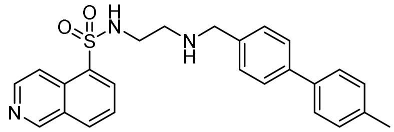

p-Tolylboronic acid (31 mg, 0.23 mmol) was subjected to general procedure D with **113a** (0.10 g, 0.19 mmol) followed by general procedure A to provide **62** (46 mg, 0.11 mmol, 58% over two steps).

$^1$H NMR (400 MHz, $CDCl_3$) δ 9.32 (d, *J* = 1.1 Hz, 1H), 8.67 (d, *J* = 6.1 Hz, 1H), 8.47 – 8.39 (m, 2H), 8.16 (dt, *J* = 8.2, 1.1 Hz, 1H), 7.66 (dd, *J* = 8.2, 7.3 Hz, 1H), 7.49 – 7.42 (m, 4H), 7.28 – 7.21 (m, 2H), 7.18 – 7.12 (m, 2H), 3.55 (s, 2H), 3.02 – 2.96 (m, 2H), 2.69 – 2.63 (m, 2H), 2.40 (s, 3H).

$^{13}$C NMR (101 MHz, $CDCl_3$) δ 153.46, 145.35, 140.17, 138.29, 137.89, 137.22, 134.34, 133.66, 133.42, 131.37, 129.63, 129.13, 128.41, 127.08, 126.96, 126.00, 117.30, 52.87, 47.34, 42.51, 21.22.

HRMS $[C_{25}H_{25}N_3O_2S+H]^+$: 432.17402 calculated, 432.17384 found.

**_N_-(2-(((4'-Cyano-[1,1'-biphenyl]-4-yl)methyl)amino)ethyl)isoquinoline-5-sulfonamide (63)**

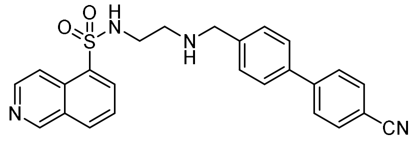

(4-Cyanophenyl)boronic acid (34 mg, 0.23 mmol) was subjected to general procedure D with **113a** (0.10 g, 0.19 mmol) followed by general procedure A to provide **63** (17 mg, 40 µmol, 21% over two steps).

$^1$H NMR (400 MHz, $CDCl_3$) δ 9.33 (d, *J* = 1.0 Hz, 1H), 8.65 (dd, *J* = 6.1, 1.2 Hz, 1H), 8.45 (ddd, *J* = 7.4, 2.2, 1.1 Hz, 2H), 8.18 (dt, *J* = 8.2, 1.0 Hz, 1H), 7.74 – 7.62 (m, 5H), 7.51 – 7.45 (m, 2H), 7.26 – 7.20 (m, 2H), 3.61 (s, 2H), 3.06 – 2.99 (m, 2H), 2.73 – 2.61 (m, 2H).

$^{13}$C NMR (101 MHz, $CDCl_3$) δ 153.40, 145.22, 145.20, 140.23, 138.03, 134.29, 133.65, 133.44, 132.69, 131.30, 129.09, 128.70, 127.64, 127.34, 126.03, 119.03, 117.29, 110.87, 52.72, 47.49, 42.51.

HRMS $[C_{25}H_{22}N_4O_2S+H]^+$: 443.15362 calculated, 443.15333 found.

**_N_-(2-(((4'-(Trifluoromethyl)-[1,1'-biphenyl]-4-yl)methyl)amino)ethyl)isoquinoline-5-sulfonamide (64)**

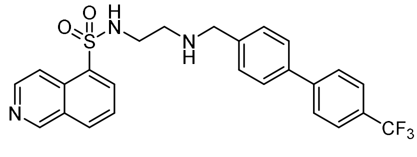

(4-(Trifluoromethyl)phenyl)boronic acid (44 mg, 0.23 mmol) was subjected to general procedure D with **113a** (0.10 g, 0.19 mmol) followed by general procedure A to provide **64** (64 mg, 0.13 mmol, 68% over two steps).

$^1$H NMR (400 MHz, $CDCl_3$) δ 9.33 (d, *J* = 1.0 Hz, 1H), 8.67 (d, *J* = 6.1 Hz, 1H), 8.48 – 8.42 (m, 2H), 8.18 (dt, *J* = 8.2, 1.1 Hz, 1H), 7.72 – 7.63 (m, 5H), 7.52 – 7.47 (m, 2H), 7.24 – 7.18 (m, 2H), 3.59 (s, 2H), 3.04 – 2.96 (m, 2H), 2.72 – 2.64 (m, 2H).

$^{13}C$ NMR (101 MHz, $CDCl_3$) δ 153.44, 145.27, 144.30, 139.70, 138.68, 134.29, 133.66, 133.46, 131.33, 129.36 (q, *J* = 32 Hz), 129.12, 128.60, 127.40, 127.36, 126.03, 125.82 (q, *J* = 3.9 Hz), 124.40 (q, *J* = 273 Hz), 117.28, 52.77, 47.42, 42.51.

HRMS $[C_{25}H_{22}F_3N_3O_2S+H]^+$: 486.14576 calculated, 486.14555 found.

***N*-(2-((4-(Pyridin-4-yl)benzyl)amino)ethyl)isoquinoline-5-sulfonamide (65)**

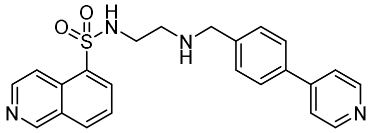

Pyridin-4-ylboronic acid (28 mg, 0.23 mmol) was subjected to general procedure D with **113a** (0.10 g, 0.19 mmol) followed by general procedure A to provide **65** (38 mg, 91 µmol, 48% over two steps).

$^1H$ NMR (400 MHz, $CDCl_3$) δ 9.33 (d, *J* = 1.0 Hz, 1H), 8.66 – 8.60 (m, 3H), 8.48 – 8.42 (m, 2H), 8.18 (dt, *J* = 8.3, 1.2 Hz, 1H), 7.69 (dd, *J* = 8.2, 7.3 Hz, 1H), 7.55 – 7.50 (m, 2H), 7.49 – 7.45 (m, 2H), 7.26 – 7.21 (m, 2H), 3.61 (s, 2H), 3.08 – 2.99 (m, 2H), 2.74 – 2.65 (m, 2H).

$^{13}C$ NMR (101 MHz, $CDCl_3$) δ 153.41, 150.22, 148.04, 145.21, 140.77, 136.91, 134.43, 133.61, 133.42, 131.34, 129.11, 128.74, 127.12, 126.04, 121.61, 117.33, 52.80, 47.59, 42.56.

HRMS $[C_{23}H_{22}N_4O_2S+H]^+$: 419.15362 calculated, 419.15335 found.

***N*-(2-(((3'-Fluoro-[1,1'-biphenyl]-4-yl)methyl)amino)ethyl)isoquinoline-5-sulfonamide (66)**

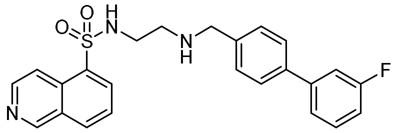

(3-Fluorophenyl)boronic acid (32 mg, 0.23 mmol) was subjected to general procedure D with **113a** (0.10 g, 0.19 mmol) followed by general procedure A to provide **66** (29 mg, 70 µmol, 37% over two steps).

$^1H$ NMR (400 MHz, $CDCl_3$) δ 9.34 (d, *J* = 1.0 Hz, 1H), 8.68 (d, *J* = 6.1 Hz, 1H), 8.47 – 8.39 (m, 2H), 8.18 (dt, *J* = 8.1, 1.1 Hz, 1H), 7.68 (dd, *J* = 8.2, 7.3 Hz, 1H), 7.50 – 7.44 (m, 2H), 7.44 – 7.31 (m, 2H), 7.28 – 7.23 (m, 1H), 7.22 – 7.16 (m, 2H), 7.07 – 7.00 (m, 1H), 3.58 (s, 2H), 3.04 – 2.95 (m, 2H), 2.71 – 2.64 (m, 2H).

$^{13}C$ NMR (101 MHz, $CDCl_3$) δ 164.53, 153.49, 145.40, 143.08 (d, *J* = 8.7 Hz), 139.24, 138.98, 134.30, 133.70, 133.49, 131.38, 130.40 (d, *J* = 8.7 Hz), 129.15, 128.56, 127.28, 126.02, 122.76 (d, *J* = 2.8 Hz), 117.29, 114.22 (d, *J* = 23 Hz), 114.00 (d, *J* = 23 Hz), 52.82, 47.36, 42.50.

HRMS $[C_{24}H_{22}FN_3O_2S +H]^+$: 436.14895 calculated, 436.14864 found.

***N*-(2-(((3'-Methyl-[1,1'-biphenyl]-4-yl)methyl)amino)ethyl)isoquinoline-5-sulfonamide (67)**

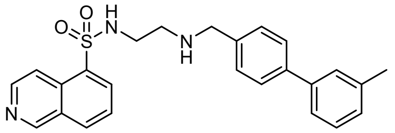

*m*-Tolylboronic acid (31 mg, 0.23 mmol) was subjected to general procedure D with **113a** (0.10 g, 0.19 mmol) followed by general procedure A to provide **67** (59 mg, 0.14 mmol, 74% over two steps).

$^1H$ NMR (400 MHz, $CDCl_3$) δ 9.31 (d, *J* = 1.0 Hz, 1H), 8.65 (d, *J* = 6.1 Hz, 1H), 8.49 – 8.39 (m, 2H), 8.14 (dt, *J* = 8.2, 1.1 Hz, 1H), 7.65 (dd, *J* = 8.2, 7.3 Hz, 1H), 7.50 – 7.41 (m, 2H), 7.39 – 7.29 (m, 3H), 7.19 – 7.10 (m, 3H), 3.56 (s, 2H), 3.03 – 2.97 (m, 2H), 2.70 – 2.63 (m, 2H), 2.42 (s, 3H).

$^{13}C$ NMR (101 MHz, $CDCl_3$) δ 153.40, 145.23, 140.72, 140.29, 138.46, 138.44, 134.36, 133.62, 133.38, 131.34, 129.09, 128.79, 128.38, 128.15, 127.88, 127.25, 126.00, 124.19, 117.32, 52.84, 47.41, 42.50, 21.64.

HRMS $[C_{25}H_{25}N_3O_2S+H]^+$: 432.17402 calculated, 432.17371 found.

***N*-(2-(((3'-Cyano-[1,1'-biphenyl]-4-yl)methyl)amino)ethyl)isoquinoline-5-sulfonamide (68)**

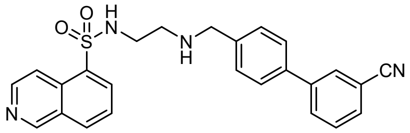

(3-Cyanophenyl)boronic acid (34 mg, 0.23 mmol) was subjected to general procedure D with **113a** (0.10 g, 0.19 mmol) followed by general procedure A to provide **68** (21 mg, 50 µmol, 26% over two steps).

$^{1}$H NMR (400 MHz, $CDCl_3$) δ 9.35 (d, *J* = 1.0 Hz, 1H), 8.69 (d, *J* = 6.1 Hz, 1H), 8.45 (ddd, *J* = 7.1, 4.9, 1.1 Hz, 2H), 8.20 (dt, *J* = 8.2, 1.1 Hz, 1H), 7.86 – 7.82 (m, 1H), 7.80 (ddd, *J* = 7.8, 1.9, 1.2 Hz, 1H), 7.70 (dd, *J* = 8.2, 7.4 Hz, 1H), 7.64 (dt, *J* = 7.7, 1.4 Hz, 1H), 7.55 (d, *J* = 7.7, 1H), 7.50 – 7.44 (m, 2H), 7.25 – 7.20 (m, 2H), 3.61 (s, 2H), 3.05 – 2.96 (m, 2H), 2.74 – 2.64 (m, 2H).

$^{13}$C NMR (101 MHz, $CDCl_3$) δ 153.51, 145.43, 142.08, 139.86, 137.96, 134.27, 133.73, 133.53, 131.52, 131.38, 130.86, 130.71, 129.79, 129.16, 128.79, 127.31, 126.04, 118.97, 117.27, 113.10, 52.76, 47.38, 42.48.

HRMS $[C_{25}H_{22}N_4O_2S+H]^+$: 443.15362 calculated, 443.15321 found.

***N*-(2-(((3'-(Trifluoromethyl)-[1,1'-biphenyl]-4-yl)methyl)amino)ethyl)isoquinoline-5-sulfonamide (69)**

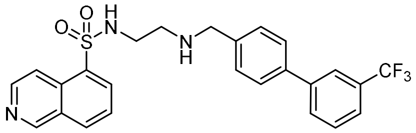

(3-(Trifluoromethyl)phenyl)boronic acid (43 mg, 0.23 mmol) was subjected to general procedure D followed by general procedure A to provide **69** (37 mg, 80 µmol, 42% over two steps).

$^{1}$H NMR (400 MHz, $CDCl_3$) δ 9.33 (d, *J* = 1.0 Hz, 1H), 8.68 (d, *J* = 6.1 Hz, 1H), 8.48 – 8.42 (m, 2H), 8.17 (dt, *J* = 8.3, 1.1 Hz, 1H), 7.79 (d, *J* = 1.8 Hz, 1H), 7.74 (dt, *J* = 7.5, 1.7 Hz, 1H), 7.68 (dd, *J* = 8.2, 7.3 Hz, 1H), 7.62 – 7.53 (m, 2H), 7.51 – 7.45 (m, 2H), 7.24 – 7.19 (m, 2H), 3.59 (s, 2H), 3.04 – 2.97 (m, 2H), 2.72 – 2.63 (m, 2H).

$^{13}$C NMR (101 MHz, $CDCl_3$) δ 153.45, 145.31, 141.59, 139.52, 138.71, 134.31, 133.65, 133.45, 131.35, 131.21 (q, *J* = 32 Hz), 130.41, 129.39, 129.12, 128.63, 127.32, 126.02, 124.27 (q, *J* = 273 Hz), 124.06, 123.87, 117.27, 52.79, 47.41, 42.52.

HRMS $[C_{25}H_{22}F_3N_3O_2S+H]^+$: 486.14576 calculated, 486.14540 found.

***N*-(2-(((2'-Chloro-[1,1'-biphenyl]-4-yl)methyl)amino)ethyl)isoquinoline-5-sulfonamide (70)**

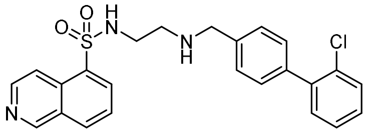

(2-Chlorophenyl)boronic acid (36 mg, 0.23 mmol) was subjected to general procedure D with **113a** (0.10 g, 0.19 mmol) followed by general procedure A to provide **70** (11 mg, 24 µmol, 13% over two steps).

$^{1}$H NMR (400 MHz, $CDCl_3$) δ 9.35 (d, *J* = 1.0 Hz, 1H), 8.68 (d, *J* = 6.1 Hz, 1H), 8.49 – 8.42 (m, 2H), 8.19 (dt, *J* = 8.3, 1.1 Hz, 1H), 7.69 (dd, *J* = 8.2, 7.4 Hz, 1H), 7.47 (dt, *J* = 7.1, 1.3 Hz, 1H), 7.39 – 7.23 (m, 6H), 7.23 – 7.16 (m, 2H), 3.61 (s, 2H), 3.06 – 2.99 (m, 2H), 2.77 – 2.67 (m, 2H).

$^{13}$C NMR (101 MHz, $CDCl_3$) δ 153.47, 145.38, 140.16, 138.95, 138.42, 134.32, 133.68, 133.46, 132.53, 131.45, 131.37, 130.08, 129.68, 129.55, 129.14, 128.70, 127.66, 127.36, 127.00, 126.03, 117.29, 52.90, 47.40, 42.50.

HRMS $[C_{24}H_{22}ClN_3O_2S+H]^+$: 452.11940 calculated, 452.11901 found.

***N*-(2-(((2'-Methyl-[1,1'-biphenyl]-4-yl)methyl)amino)ethyl)isoquinoline-5-sulfonamide (71)**

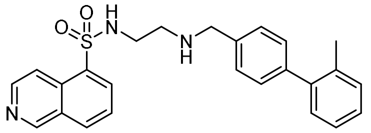

*o*-Tolylboronic acid (31 mg, 0.23 mmol) was subjected to general procedure D with **113a** (0.10 g, 0.19 mmol) followed by general procedure A to provide **71** (37 mg, 80 µmol, 42% over two steps).

$^{1}$H NMR (400 MHz, $CDCl_3$) δ 9.35 (d, *J* = 1.1 Hz, 1H), 8.68 (d, *J* = 6.1 Hz, 1H), 8.48 – 8.43 (m, 2H), 8.19 (dt, *J* = 8.2, 1.1 Hz, 1H), 7.69 (dd, *J* = 8.2, 7.3 Hz, 1H), 7.30 – 7.12 (m, 8H), 3.58 (s, 2H), 3.03 – 2.98 (m, 2H), 2.72 – 2.67 (m, 2H), 2.26 (s, 3H).

$^{13}C$ NMR (101 MHz, $CDCl_3$) δ 153.47, 145.37, 141.54, 141.03, 137.97, 135.41, 134.31, 133.69, 133.47, 131.38, 130.47, 129.87, 129.42, 129.15, 127.74, 127.41, 126.02, 125.91, 117.30, 52.93, 47.38, 42.47, 20.60.

HRMS $[C_{25}H_{25}N_3O_2S+H]^+$: 432.17402 calculated, 432.17368 found.

**_N_-(2-(((2'-Cyano-[1,1'-biphenyl]-4-yl)methyl)amino)ethyl)isoquinoline-5-sulfonamide (72)**

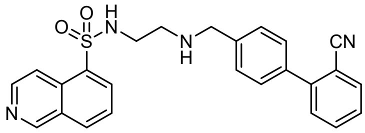


(2-Cyanophenyl)boronic acid (34 mg, 0.23 mmol) was subjected to general procedure D with **113a** (0.10 g, 0.19 mmol) followed by general procedure A to provide **72** (44 mg, 0.10 mmol, 53% over two steps).

$^1H$ NMR (400 MHz, $CDCl_3$) δ 9.34 (d, *J* = 1.0 Hz, 1H), 8.67 (d, *J* = 6.1 Hz, 1H), 8.49 – 8.42 (m, 2H), 8.19 (dt, *J* = 8.2, 1.1 Hz, 1H), 7.76 (ddd, *J* = 7.8, 1.3, 0.6 Hz, 1H), 7.72 – 7.59 (m, 2H), 7.52 – 7.41 (m, 4H), 7.25 – 7.19 (m, 2H), 3.59 (s, 2H), 3.06 – 2.97 (m, 2H), 2.72 – 2.63 (m, 2H).

$^{13}C$ NMR (101 MHz, $CDCl_3$) δ 153.44, 145.30, 145.17, 140.10, 137.14, 134.31, 133.84, 133.69, 133.44, 133.01, 131.34, 130.11, 129.12, 128.97, 128.30, 127.69, 126.05, 118.88, 117.31, 111.22, 52.75, 47.45, 42.49.

HRMS $[C_{25}H_{22}N_4O_2S+H]^+$: 443.15362 calculated, 443.15325 found.

**_N_-(2-(((2'-Methoxy-[1,1'-biphenyl]-4-yl)methyl)amino)ethyl)isoquinoline-5-sulfonamide (73)**

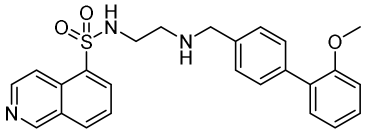


(2-Methoxyphenyl)boronic acid (35 mg, 0.23 mmol) was subjected to general procedure D with **113a** (0.10 g, 0.19 mmol) followed by general procedure A to provide **73** (28 mg, 80 µmol, 42% over two steps).

$^1H$ NMR (400 MHz, $CDCl_3$) δ 9.33 (d, *J* = 1.0 Hz, 1H), 8.68 (d, *J* = 6.1 Hz, 1H), 8.48 – 8.40 (m, 2H), 8.17 (dt, *J* = 8.2, 1.1 Hz, 1H), 7.67 (dd, *J* = 8.2, 7.3 Hz, 1H), 7.48 – 7.41 (m, 2H), 7.36 – 7.27 (m, 2H), 7.17 – 7.12 (m, 2H), 7.05 – 6.96 (m, 2H), 3.81 (s, 3H), 3.56 (s, 2H), 3.04 – 2.97 (m, 2H), 2.71 – 2.65 (m, 2H).

$^{13}C$ NMR (101 MHz, $CDCl_3$) δ 156.52, 153.46, 145.39, 138.04, 137.62, 134.34, 133.66, 133.42, 131.38, 130.89, 130.27, 129.75, 129.13, 128.80, 127.68, 126.00, 120.96, 117.31, 111.29, 55.62, 52.92, 47.30, 42.46.

HRMS $[C_{25}H_{25}N_3O_3S+H]^+$: 448.16894 calculated, 448.16854 found.

**_N_-(2-(((3',4'-Dichloro-[1,1'-biphenyl]-4-yl)methyl)amino)ethyl)isoquinoline-5-sulfonamide (74)**

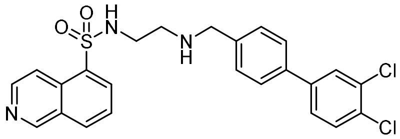


(3,4-Dichlorophenyl)boronic acid (44 mg, 0.23 mmol) was subjected to general procedure D with **113a** (0.10 g, 0.19 mmol) followed by general procedure A to provide **74** (13 mg, 27 µmol, 14% over two steps).

$^1H$ NMR (400 MHz, $CDCl_3$) δ 9.34 (d, *J* = 1.0 Hz, 1H), 8.68 (d, *J* = 6.1 Hz, 1H), 8.48 – 8.41 (m, 2H), 8.19 (dt, *J* = 8.3, 1.1 Hz, 1H), 7.69 (dd, *J* = 8.2, 7.3 Hz, 1H), 7.64 (d, *J* = 2.1 Hz, 1H), 7.50 (d, *J* = 8.3 Hz, 1H), 7.46 – 7.41 (m, 2H), 7.39 (dd, *J* = 8.3, 2.2 Hz, 1H), 7.22 – 7.17 (m, 2H), 3.58 (s, 2H), 3.04 – 2.92 (m, 2H), 2.74 – 2.63 (m, 2H).

$^{13}C$ NMR (101 MHz, $CDCl_3$) δ 153.50, 145.42, 140.85, 139.61, 137.82, 134.29, 133.71, 133.51, 131.58, 131.38, 130.85, 129.16, 128.95, 128.66, 127.15, 126.38, 126.03, 117.27, 52.79, 47.36, 42.50.

HRMS: found $[C_{24}H_{21}Cl_2N_3O_2S+H]^+$ calculated for $[C_{24}H_{21}Cl_2N_3O_2S+H]^+$ 486.08043 calculated, 486.07992 found.

***N*-(2-(((3',5'-Dichloro-[1,1'-biphenyl]-4-yl)methyl)amino)ethyl)isoquinoline-5-sulfonamide (75)**

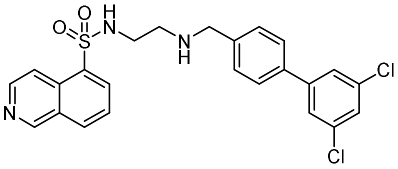

(3,5-Dichlorophenyl)boronic acid (44 mg, 0.23 mmol) was subjected to general procedure D with **113a** (0.10 g, 0.19 mmol) followed by general procedure A to provide **75** (5.0 mg, 10 µmol, 5% over two steps).

$^{1}$H NMR (400 MHz, CDCl$_3$) δ 9.35 (d, *J* = 1.0 Hz, 1H), 8.70 (d, *J* = 6.1 Hz, 1H), 8.48 – 8.41 (m, 2H), 8.20 (dt, *J* = 8.2, 1.1 Hz, 1H), 7.70 (dd, *J* = 8.2, 7.3 Hz, 1H), 7.46 – 7.41 (m, 4H), 7.34 (t, *J* = 1.9 Hz, 1H), 7.23 – 7.18 (m, 2H), 3.59 (s, 2H), 3.02 – 2.96 (m, 2H), 2.71 – 2.65 (m, 2H).

HRMS [$C_{24}H_{21}Cl_2N_3O_2S$+H]$^+$: 486.08043 calculated, 486.08022 found.

***N*-(2-((4-(Naphthalen-2-yl)benzyl)amino)ethyl)isoquinoline-5-sulfonamide (76)**

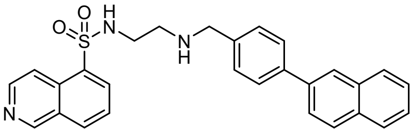

Naphthalen-2-ylboronic acid (40 mg, 0.23 mmol) was subjected to general procedure D with **113a** (0.10 g, 0.19 mmol) followed by general procedure A to provide **76** (80 mg, 0.17 mmol, 89% over two steps).

$^{1}$H NMR (400 MHz, CDCl$_3$) δ 9.28 (d, *J* = 1.1 Hz, 1H), 8.65 (dd, *J* = 6.2, 1.0 Hz, 1H), 8.46 (dd, *J* = 6.1, 1.0 Hz, 1H), 8.41 (dd, *J* = 7.4, 1.2 Hz, 1H), 8.09 (dq, *J* = 8.2, 1.4 Hz, 1H), 7.98 (d, *J* = 1.8 Hz, 1H), 7.91 – 7.81 (m, 3H), 7.68 (dd, *J* = 8.6, 1.9 Hz, 1H), 7.65 – 7.56 (m, 3H), 7.53 – 7.40 (m, 2H), 7.22 – 7.15 (m, 2H), 3.58 (s, 2H), 3.04 – 2.94 (m, 2H), 2.73 – 2.55 (m, 2H).

$^{13}$C NMR (101 MHz, CDCl$_3$) δ 153.37, 145.18, 140.01, 138.45, 137.98, 134.35, 133.70, 133.59, 133.33, 132.66, 131.31, 129.06, 128.58, 128.54, 128.25, 127.71, 127.46, 126.43, 126.06, 125.98, 125.67, 125.44, 117.35, 52.79, 47.45, 42.44.

HRMS [$C_{28}H_{25}N_3O_2S$+H]$^+$: 468.17402 calculated, 468.17387 found.

***N*-(2-((4-(Benzo[*d*][1,3]dioxol-5-yl)benzyl)amino)ethyl)isoquinoline-5-sulfonamide (77)**

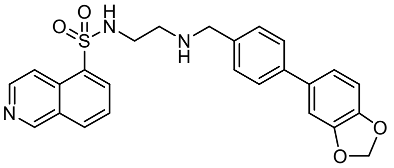

Benzo[*d*][1,3]dioxol-5-ylboronic acid (38 mg, 0.23 mmol) was subjected to general procedure D with **113a** (0.10 g, 0.19 mmol) followed by general procedure A to provide **77** (24 mg, 50 µmol, 26% over two steps).

$^{1}$H NMR (400 MHz, CDCl$_3$) δ 9.34 (d, *J* = 1.0 Hz, 1H), 8.68 (d, *J* = 6.1 Hz, 1H), 8.47 – 8.40 (m, 2H), 8.18 (dt, *J* = 8.2, 1.1 Hz, 1H), 7.68 (dd, *J* = 8.2, 7.3 Hz, 1H), 7.45 – 7.38 (m, 2H), 7.18 – 7.11 (m, 2H), 7.06 – 7.00 (m, 2H), 6.93 – 6.85 (m, 1H), 6.01 (s, 2H), 3.56 (s, 2H), 3.05 – 2.96 (m, 2H), 2.74 – 2.62 (m, 2H).

$^{13}$C NMR (101 MHz, CDCl$_3$) δ 153.49, 148.27, 147.24, 145.44, 140.06, 138.07, 135.18, 134.31, 133.69, 133.47, 131.38, 129.15, 128.48, 127.06, 126.01, 120.65, 117.29, 108.74, 107.68, 101.30, 52.80, 47.28, 42.44.

HRMS [$C_{25}H_{23}N_3O_4S$+H]$^+$: 462.14820 calculated, 462.14761 found.

***N*-(2-((4-(2,3-Dihydrobenzo[*b*][1,4]dioxin-6-yl)benzyl)amino)ethyl)isoquinoline-5-sulfonamide (78)**

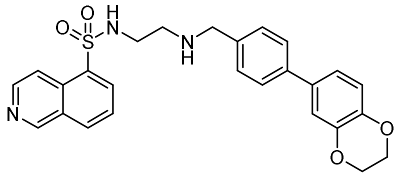

(2,3-Dihydrobenzo[b][1,4]dioxin-6-yl)boronic acid (41 mg, 0.23 mmol) was subjected to general procedure D with **113a** (0.10 g, 0.19 mmol,) followed by general procedure A to provide **78** (20 mg, 42 µmol, 22% over two steps).

$^{1}$H NMR (400 MHz, CDCl$_3$) δ 9.33 (d, *J* = 1.0 Hz, 1H), 8.68 (d, *J* = 6.1 Hz, 1H), 8.48 – 8.40 (m, 2H), 8.17 (dt, *J* = 8.1, 1.1 Hz, 1H), 7.67 (dd, *J* = 8.2, 7.3 Hz, 1H), 7.44 – 7.38 (m, 2H), 7.16 – 7.10 (m, 2H), 7.10 – 7.03 (m, 2H), 6.93 (d, *J* = 8.3 Hz, 1H), 4.30 (s, 4H), 3.55 (s, 2H), 3.02 – 2.96 (m, 2H), 2.70 – 2.62 (m, 2H).

$^{13}$C NMR (101 MHz, CDCl$_3$) δ 153.48, 145.41, 143.82, 143.32, 139.69, 137.96, 134.37, 134.33, 133.68, 133.43, 131.38, 129.14, 128.46, 126.90, 126.00, 120.16, 117.71, 117.29, 115.85, 64.60, 64.57, 52.81, 47.28, 42.45.

HRMS $[C_{26}H_{25}N_3O_4S+H]^+$: 476.16385 calculated, 476.16340 found.

***N*-(2-(([2,3'-Bipyridin]-5-ylmethyl)amino)ethyl)isoquinoline-5-sulfonamide (79)**

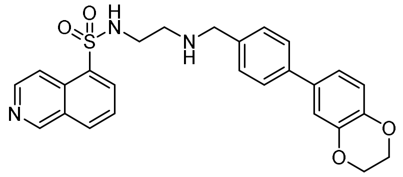

Pyridin-3-ylboronic acid (27 mg, 0.23 mmol) was subjected to general procedure D with **113b** (0.10 g, 0.21 mmol) followed by general procedure A to provide **79** (10 mg, 24 µmol, 11% over two steps).

$^1$H NMR (400 MHz, $CDCl_3$) δ 9.33 (d, *J* = 1.0 Hz, 1H), 9.15 (dd, *J* = 2.4, 0.9 Hz, 1H), 8.68 – 8.61 (m, 2H), 8.49 (dd, *J* = 2.3, 0.9 Hz, 1H), 8.47 – 8.43 (m, 2H), 8.29 (ddd, *J* = 8.0, 2.4, 1.7 Hz, 1H), 8.18 (d, 8.3 Hz), 7.72 – 7.55 (m, 3H), 7.41 (ddd, *J* = 8.0, 4.8, 0.9 Hz, 1H), 3.64 (s, 2H), 3.12 – 2.98 (m, 2H), 2.77 – 2.63 (m, 2H).

$^{13}$C NMR (101 MHz, $CDCl_3$) δ 153.78, 153.46, 149.92, 149.79, 148.18, 145.28, 136.73, 134.65, 134.41, 134.39, 134.30, 133.68, 133.44, 131.32, 129.11, 126.06, 123.78, 120.40, 117.26, 50.33, 47.75, 42.60.

HRMS $[C_{22}H_{21}N_5O_2S+H]^+$: 420.14887 calculated, 420.14839 found.

***N*-(2-(((6-Phenylpyridin-3-yl)methyl)amino)ethyl)isoquinoline-5-sulfonamide (80)**

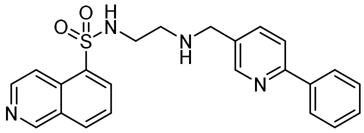

Phenylboronic acid (27 mg, 0.23 mmol) was subjected to general procedure D with **113b** (0.10 g, 0.21 mmol) followed by general procedure A to provide **80** (24 mg, 57 µmol, 27% over two steps).

$^1$H NMR (400 MHz, $CDCl_3$) δ 9.32 (d, *J* = 1.0 Hz, 1H), 8.66 (d, *J* = 6.1 Hz, 1H), 8.47 – 8.41 (m, 1H), 8.16 (dt, *J* = 8.2, 1.1 Hz, 1H), 7.97 – 7.92 (m, 2H), 7.67 (dd, *J* = 8.2, 7.3 Hz, 1H), 7.61 (dd, *J* = 8.1, 0.9 Hz, 1H), 7.54 – 7.37 (m, 4H), 3.59 (s, 2H), 3.10 – 2.95 (m, 2H), 2.71 – 2.61 (m, 2H).

$^{13}$C NMR (101 MHz, $CDCl_3$) δ 156.60, 153.47, 149.36, 145.30, 139.08, 136.57, 134.33, 133.71, 133.45, 133.37, 131.32, 129.10, 128.89, 126.94, 126.05, 120.42, 117.26, 50.35, 47.58, 42.58.

HRMS $[C_{23}H_{22}N_4O_2S+H]^+$: 419.15362 calculated, 419.15334 found.

***N*-(2-(((6'-Fluoro-[2,3'-bipyridin]-5-yl)methyl)amino)ethyl)isoquinoline-5-sulfonamide (81)**

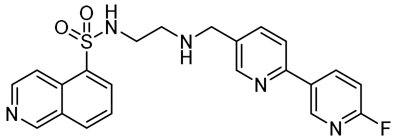

(6-Fluoropyridin-3-yl)boronic acid (30 mg, 0.23 mmol) was subjected to general procedure D with **113b** (0.10 g, 0.21 mmol) followed by general procedure A to provide **81** (20 mg, 46 µmol, 22% over two steps).

$^1$H NMR (400 MHz, $CDCl_3$) δ 9.34 (d, *J* = 1.0 Hz, 1H), 8.77 (dt, *J* = 2.6, 0.8 Hz, 1H), 8.68 (d, *J* = 6.1 Hz, 1H), 8.50 – 8.39 (m, 4H), 8.20 (dt, *J* = 8.2, 1.1 Hz, 1H), 7.70 (dd, *J* = 8.2, 7.3 Hz, 1H), 7.65 – 7.56 (m, 2H), 7.04 (ddd, *J* = 8.6, 3.0, 0.7 Hz, 1H), 3.64 (s, 2H), 3.08 – 3.00 (m, 2H), 2.73 – 2.67 (m, 2H).

$^{13}$C NMR (101 MHz, $CDCl_3$) δ 164.06, (d, *J* = 243 Hz), 153.50, 152.83, 149.77, 146.20 (d, *J* = 14.5 Hz), 145.36, 139.88 (d, *J* = 8.1 Hz), 136.80, 134.27 (d, *J* = 4.5 Hz), 133.76, 133.52, 132.93, 131.32, 129.13, 126.07, 120.13, 117.21, 109.70 (d, *J* = 23 Hz), 50.29, 47.63, 42.57.

HRMS $[C_{22}H_{20}FN_5O_2S+H]^+$: 438.13945 calculated, 438.13902 found.

***N*-(2-(((6-(4-Fluorophenyl)pyridin-3-yl)methyl)amino)ethyl)isoquinoline-5-sulfonamide (82)**

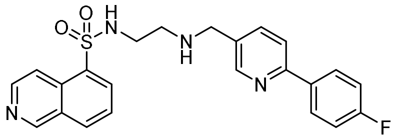

(4-Fluorophenyl)boronic acid (30 mg, 0.23 mmol) was subjected to general procedure D with **113b** (0.10 g, 0.21 mmol) followed by general procedure A to provide **82** (25 mg, 57 µmol, 27% over two steps).

$^1$H NMR (400 MHz, $CDCl_3$) δ 9.34 (d, *J* = 1.0 Hz, 1H), 8.68 (d, *J* = 6.1 Hz, 1H), 8.48 – 8.40 (m, 3H), 8.18 (dt, *J* = 8.2, 1.1 Hz, 1H), 7.99 – 7.92 (m, 2H), 7.69 (dd, *J* = 8.2, 7.4 Hz, 1H), 7.59 (dd, *J* = 8.1, 0.9 Hz, 1H), 7.52 (dd, *J* = 8.2, 2.3 Hz, 1H), 7.19 – 7.09 (m, 2H), 3.60 (s, 2H), 3.05 – 2.98 (m, 2H), 2.70 – 2.64 (m, 2H).

$^{13}$C NMR (101 MHz, $CDCl_3$) δ 163.63 (d, *J* = 243 Hz), 155.65, 153.52, 149.38, 145.39, 136.61, 135.26, 134.25, 133.76, 133.51, 133.28, 131.33, 129.13, 128.75 (d, *J* = 8.0 Hz), 126.05, 120.08, 117.22, 115.81 (d, *J* = 23 Hz), 50.33, 47.52, 42.56.

HRMS $[C_{23}H_{21}FN_4O_2S+H]^+$: 437.14420 calculated, 437.14383 found.

***N*-(2-((3-Chloro-4-(pyridin-3-yl)benzyl)amino)ethyl)isoquinoline-5-sulfonamide (83)**

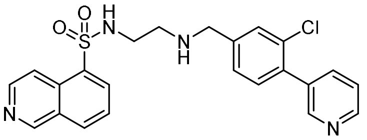


Pyridin-3-ylboronic acid (27 mg, 0.23 mmol) was subjected to general procedure D with **113c** (0.10 g, 0.23 mmol) followed by general procedure A to provide **83** (11 mg, 24 µmol, 10% over two steps).

$^1$H NMR (400 MHz, $CDCl_3$) δ 9.35 (d, *J* = 1.0 Hz, 1H), 8.69 (d, *J* = 6.1 Hz, 1H), 8.66 (dd, *J* = 2.2, 0.9 Hz, 1H), 8.63 (dd, *J* = 4.9, 1.7 Hz, 1H), 8.49 – 8.43 (m, 2H), 8.21 (dt, *J* = 8.2, 1.1 Hz, 1H), 7.82 – 7.76 (m, 1H), 7.71 (dd, *J* = 8.2, 7.3 Hz, 1H), 7.38 (ddd, *J* = 7.9, 4.9, 0.9 Hz, 1H), 7.31 (d, *J* = 1.7 Hz, 1H), 7.25 (dd, *J* = 7.5, 1.5 Hz, 2H), 7.20 – 7.07 (m, 2H), 3.59 (s, 2H), 3.07 – 2.98 (m, 2H), 2.73 – 2.64 (m, 2H).

$^{13}$C NMR (101 MHz, $CDCl_3$) δ 153.51, 150.08, 148.90, 145.43, 141.53, 137.03, 135.83, 134.92, 134.29, 133.76, 133.54, 132.87, 131.41, 131.37, 129.54, 129.16, 126.72, 126.06, 123.06, 117.23, 52.33, 47.49, 42.56.

HRMS $[C_{23}H_{21}ClN_4O_2S+H]^+$: 453.11466 calculated, 453.11422 found.

***N*-(2-(((2-Chloro-[1,1'-biphenyl]-4-yl)methyl)amino)ethyl)isoquinoline-5-sulfonamide (84)**

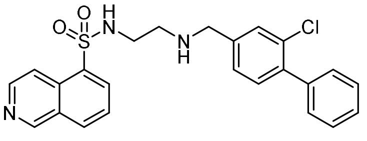


Phenylboronic acid (27 mg, 0.23 mmol) was subjected to general procedure D with **113c** (0.10 g, 0.23 mmol) followed by general procedure A to provide **84** (23 mg, 51 µmol, 22% over two steps).

$^1$H NMR (400 MHz, $CDCl_3$) δ 9.41 – 9.29 (m, 1H), 8.70 (d, *J* = 6.1 Hz, 1H), 8.50 – 8.39 (m, 2H), 8.20 (d, *J* = 8.2 Hz, 1H), 7.70 (t, *J* = 7.8 Hz, 1H), 7.48 – 7.35 (m, 5H), 7.32 – 7.18 (m, 2H), 7.07 (dd, *J* = 7.8, 1.7 Hz, 1H), 3.56 (s, 2H), 3.07 – 2.97 (m, 2H), 2.72 – 2.62 (m, 2H).

$^{13}$C NMR (101 MHz, $CDCl_3$) δ 153.50, 145.43, 140.42, 139.43, 139.12, 134.28, 133.75, 133.51, 132.58, 131.53, 131.36, 129.54, 129.34, 129.14, 128.19, 127.76, 126.43, 126.04, 117.23, 52.33, 47.39, 42.53.

HRMS $[C_{24}H_{22}ClN_3O_2S+H]^+$: 452.11940 calculated, 452.11890 found.

***N*-(2-((3-Chloro-4-(6-fluoropyridin-3-yl)benzyl)amino)ethyl)isoquinoline-5-sulfonamide (85)**

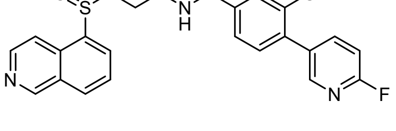


(6-Fluoropyridin-3-yl)boronic acid (30 mg, 0.23 mmol) was subjected to general procedure D with **113c** (0.10 g, 0.23 mmol) followed by general procedure A to provide **85** (16 mg, 34 µmol, 15% over two steps).

$^1$H NMR (400 MHz, $CDCl_3$) δ 9.34 (d, *J* = 1.0 Hz, 1H), 8.68 (d, *J* = 6.1 Hz, 1H), 8.49 – 8.42 (m, 2H), 8.27 – 8.23 (m, 1H), 8.20 (dt, *J* = 8.3, 1.1 Hz, 1H), 7.89 (ddd, *J* = 8.5, 7.6, 2.5 Hz, 1H), 7.70 (dd, *J* = 8.2, 7.4 Hz, 1H), 7.33 – 7.08 (m, 3H), 7.00 (ddd, *J* = 8.4, 3.0, 0.7 Hz, 1H), 3.57 (s, 2H), 3.06 – 2.97 (m, 2H), 2.71 – 2.63 (m, 2H).

$^{13}$C NMR (101 MHz, $CDCl_3$) δ 163.11 (d, *J* = 243 Hz), 153.51, 147.92 (d, *J* = 14.5 Hz), 145.42, 142.32 (d, *J* = 8.1 Hz), 141.78, 137.99, 134.68, 134.24, 133.77, 133.55, 132.91, 131.34, 129.57, 129.16, 128.35, 126.76, 126.05, 125.42, 117.20, 108.99 (d, *J* = 23 Hz), 52.28, 47.47, 42.55.

HRMS $[C_{23}H_{20}ClFN_4O_2S+H]^+$: 471.10523 calculated, 471.10486 found.

***N*-(2-(((2-Chloro-4'-fluoro-[1,1'-biphenyl]-4-yl)methyl)amino)ethyl)isoquinoline-5-sulfonamide (86)**

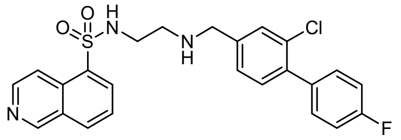

(4-Fluorophenyl)boronic acid (30 mg, 0.23 mmol) was subjected to general procedure D with **113c** (0.10 g, 0.23 mmol) followed by general procedure A to provide **86** (20 mg, 43 µmol, 19% over two steps).

$^{1}$H NMR (400 MHz, $CDCl_3$) δ 9.35 (d, *J* = 1.0 Hz, 1H), 8.71 (d, *J* = 6.1 Hz, 1H), 8.49 – 8.41 (m, 2H), 8.21 (dt, *J* = 8.3, 1.2 Hz, 1H), 7.71 (dd, *J* = 8.2, 7.4 Hz, 1H), 7.43 – 7.36 (m, 2H), 7.25 – 7.16 (m, 2H), 7.16 – 7.09 (m, 2H), 7.07 (dd, *J* = 7.8, 1.8 Hz, 1H), 3.56 (s, 2H), 3.05 – 2.96 (m, 2H), 2.72 – 2.61 (m, 2H).

$^{13}$C NMR (101 MHz, $CDCl_3$) δ 163.73 (d, *J* = 243 Hz), 153.53, 145.50, 140.62, 138.59, 135.13, 134.25, 133.78, 133.57, 131.50, 131.37, 131.26 (d, *J* = 8.0 Hz), 129.40, 129.16, 126.49, 126.04, 117.21, 115.20 (d, *J* = 23 Hz), 52.32, 47.37, 42.52.

HRMS $[C_{24}H_{21}ClFN_3O_2S+H]^+$: 470.10998 calculated, 470.10941 found.

***N*-(2-((3-Fluoro-4-(pyridin-3-yl)benzyl)amino)ethyl)isoquinoline-5-sulfonamide (87)**

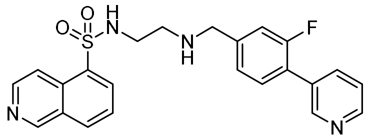

Pyridin-3-ylboronic acid (27 mg, 0.23 mmol) was subjected to general procedure D with **113d** (0.10 g, 0.20 mmol) followed by general procedure A to provide **87** (10 mg, 23 µmol, 12% over two steps).

$^{1}$H NMR (400 MHz, $CDCl_3$) δ 9.35 (d, *J* = 1.0 Hz, 1H), 8.77 (dq, *J* = 2.3, 1.0 Hz, 1H), 8.70 (d, *J* = 6.1 Hz, 1H), 8.61 (dd, *J* = 4.8, 1.7 Hz, 1H), 8.50 – 8.42 (m, 2H), 8.21 (dt, *J* = 8.2, 1.1 Hz, 1H), 7.87 (ddt, *J* = 7.9, 2.3, 1.7 Hz, 1H), 7.71 (dd, *J* = 8.2, 7.3 Hz, 1H), 7.42 – 7.31 (m, 2H), 7.05 – 6.97 (m, 2H), 3.61 (s, 2H), 3.04 – 2.98 (m, 2H), 2.71 – 2.67 (m, 2H).

HRMS $[C_{23}H_{21}FN_4O_2S+H]^+$: 437.14420 calculated, 437.14402 found.

***N*-(2-(((2-Fluoro-[1,1'-biphenyl]-4-yl)methyl)amino)ethyl)isoquinoline-5-sulfonamide (88)**

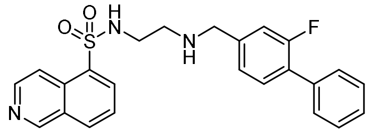

Phenylboronic acid (27 mg, 0.23 mmol) was subjected to general procedure D with **113d** (0.10 g, 0.20 mmol) followed by general procedure A to provide **88** (34 mg, 78 µmol, 39% over two steps).

$^{1}$H NMR (400 MHz, $CDCl_3$) δ 9.34 (d, *J* = 1.0 Hz, 1H), 8.68 (d, *J* = 6.1 Hz, 1H), 8.50 – 8.40 (m, 2H), 8.18 (dt, *J* = 8.3, 1.1 Hz, 1H), 7.68 (dd, *J* = 8.2, 7.4 Hz, 1H), 7.52 (dt, *J* = 8.2, 1.5 Hz, 2H), 7.48 – 7.41 (m, 2H), 7.41 – 7.28 (m, 2H), 7.01 – 6.92 (m, 2H), 3.57 (s, 2H), 3.08 – 2.95 (m, 2H), 2.74 – 2.62 (m, 2H).

$^{13}$C NMR (101 MHz, $CDCl_3$) δ 159.77 (d, *J* = 250 Hz), 153.48, 145.37, 141.23 (d, *J* = 7.2 Hz), 135.57, 134.30, 133.72, 133.48, 131.35, 130.82 (d, *J* = 3.9 Hz), 129.04 (d, *J* = 3.0 Hz), 129.13, 128.59, 127.93, 127.80, 126.03, 123.78 (d, *J* = 3.4 Hz), 117.25, 115.49 (d, *J* = 23 Hz), 52.42, 47.40, 42.55.

HRMS $[C_{24}H_{22}FN_3O_2S +H]^+$: 436.14895 calculated, 436.14876 found.

***N*-(2-((3-Fluoro-4-(6-fluoropyridin-3-yl)benzyl)amino)ethyl)isoquinoline-5-sulfonamide (89)**

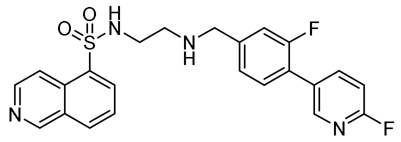

(6-Fluoropyridin-3-yl)boronic acid (30 mg, 0.23 mmol) was subjected to general procedure D with **113d** (0.10 g, 0.20 mmol) followed by general procedure A to provide **89** (18 mg, 40 µmol, 20% over two steps).

$^{1}$H NMR (400 MHz, $CDCl_3$) δ 9.35 (d, *J* = 0.9 Hz, 1H), 8.69 (d, *J* = 6.1 Hz, 1H), 8.45 (td, *J* = 7.3, 1.1 Hz, 2H), 8.36 (dq, *J* = 2.1, 1.0 Hz, 1H), 8.21 (dt, *J* = 8.3, 1.1 Hz, 1H), 7.97 (dddd, *J* = 8.5, 7.6, 2.6, 1.6 Hz, 1H), 7.70 (dd, *J* = 8.2, 7.4 Hz, 1H), 7.31 (t, *J* = 8.0 Hz, 1H), 7.07 – 6.97 (m, 3H), 3.60 (s, 2H), 3.04 – 2.98 (m, 2H), 2.71 – 2.64 (m, 2H).

$^{13}$C NMR (101 MHz, $CDCl_3$) δ 163.14 (d, *J* = 243 Hz), 159.79 (d, *J* = 250 Hz), 153.52, 147.45 (dd, *J* = 15, 3.2 Hz), 145.43, 142.67, 141.65 (dd, *J* = 8.2, 3.7 Hz), 134.25, 133.76, 133.54, 131.36, 130.38, 129.44 (dd, *J* = 4.4, 1.4 Hz), 129.15, 126.05, 124.14, 123.31 (d, *J* = 14 Hz), 117.21, 115.69 (d, *J* = 23 Hz), 109.43 (d, *J* = 38 Hz), 52.37, 47.46, 42.57.

HRMS $[C_{23}H_{20}F_2N_4O_2S+H]^+$: 455.13478 calculated, 455.13426 found.

***N*-(2-(((2,4'-Difluoro-[1,1'-biphenyl]-4-yl)methyl)amino)ethyl)isoquinoline-5-sulfonamide (90)**

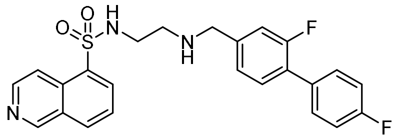

(4-Fluorophenyl)boronic acid (30 mg, 0.23 mmol) was subjected to general procedure D with **113d** (0.10 g, 0.20 mmol) followed by general procedure A to provide **90** (27 mg, 60 µmol, 30% over two steps).

$^{1}$H NMR (400 MHz, $CDCl_3$) δ 9.34 (d, *J* = 0.9 Hz, 1H), 8.69 (d, *J* = 6.1 Hz, 1H), 8.48 – 8.41 (m, 2H), 8.20 (dt, *J* = 8.3, 1.2 Hz, 1H), 7.70 (dd, *J* = 8.2, 7.4 Hz, 1H), 7.53 – 7.45 (m, 2H), 7.29 (t, *J* = 8.0 Hz, 1H), 7.16 – 7.10 (m, 2H), 7.00 – 6.90 (m, 2H), 3.57 (s, 2H), 3.06 – 2.97 (m, 2H), 2.71 – 2.63 (m, 2H).

$^{13}$C NMR (101 MHz, $CDCl_3$) δ 162.35 (d, *J* = 288 Hz), 159.89 (d, *J* = 288 Hz), 153.51, 145.43, 141.34 (d, *J* = 7.1 Hz), 134.26, 133.75, 133.53, 131.55 (d, *J* = 1.9 Hz), 131.36, 130.75 (d, *J* = 3.0 Hz), 130.67 (d, *J* = 2.9 Hz), 129.15, 126.92 (d, *J* = 13 Hz), 126.03, 123.84 (d, *J* = 3.3 Hz), 117.23, 115.67 (d, *J* = 1.8 Hz), 115.44 (d, *J* = 3.3 Hz), 52.40, 47.38, 42.54.

HRMS $[C_{23}H_{21}F_2N_3O_2S+H]^+$: 454.13953 calculated, 454.13915 found.

***N*-(2-((4-(Pyridin-3-yl)benzyl)amino)ethyl)isoquinoline-5-carboxamide (91)**

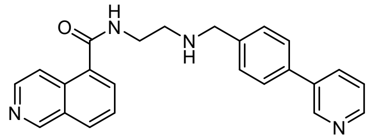

To a solution of **108** (14 mg, 44 µmol), DIPEA (11.5 µL, 66 µmol) and the isoquinoline-5-carboxylic acid (8.3 mg, 48 µmol.) in DCM (2 mL) was added HATU (17 mg, 44 µmol). The reaction mixture was stirred at room temperature overnight. after which the mixture was washed with water. The organic layer was dried over $Na_2SO_4$ and concentrated *in vacuo*, after which the residue was purified by column chromatography (5% MeOH (10% aq. $NH_3$) in DCM). The product was then subjected to general procedure A to yield title compound **91** (13 mg, 33 µmol, 81%).

$^{1}$H NMR (400 MHz, $CDCl_3$) δ 9.26 (d, *J* = 1.0 Hz, 1H), 8.80 (dd, *J* = 2.4, 0.9 Hz, 1H), 8.61 – 8.52 (m, 2H), 8.20 (dt, *J* = 6.0, 1.0 Hz, 1H), 8.04 (dt, *J* = 8.3, 1.1 Hz, 1H), 7.88 – 7.80 (m, 2H), 7.58 (dd, *J* = 8.2, 7.1 Hz, 1H), 7.54 – 7.49 (m, 2H), 7.46 – 7.40 (m, 2H), 7.36 (ddd, *J* = 7.9, 4.8, 0.9 Hz, 1H), 6.81 (t, *J* = 5.3 Hz, 1H), 3.90 (s, 2H), 3.67 (q, *J* = 5.6 Hz, 2H), 2.99 (d, *J* = 6.3 Hz, 2H).

$^{13}$C NMR (101 MHz, $CDCl_3$) δ 168.31, 152.94, 148.61, 148.30, 144.32, 139.86, 136.93, 136.29, 134.34, 133.32, 133.17, 130.50, 129.37, 129.02, 128.82, 127.38, 126.34, 123.71, 118.36, 53.18, 48.10, 39.67.

HRMS $[C_{24}H_{22}N_4O +H]^+$: 383.18664 calculated, 383.18628 found.

***N*-Methyl-*N*-(2-((4-(pyridin-2-yl)benzyl)amino)ethyl)isoquinoline-5-sulfonamide (92)**

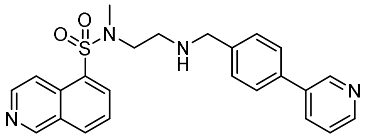

*tert*-Butyl (2-(isoquinoline-5-sulfonamido)ethyl)(4-(pyridin-2-yl)benzyl)carbamate (**109**) (7.0 mg, 38 µmol), iodomethane (3.0 µL, 46 µmol) and NaOH (1.0 mg, 19 µmol) were dissolved in DMF (1 mL). The reaction mixture was stirred overnight at 80°C and then diluted with brine (3 mL) and extracted with DCM (3×). The crude product was subjected to general procedure A to give **92 (**1.0 mg, 15% over 2 steps).

$^{1}$H NMR (400 MHz, $CDCl_3$) δ 9.33 (d, *J* = 1.0 Hz, 1H), 8.85 (dd, *J* = 2.4, 0.9 Hz, 1H), 8.67 (d, *J* = 6.2 Hz, 1H), 8.59 (dd, *J* = 4.8, 1.6 Hz, 1H), 8.51 (dt, *J* = 6.2, 0.9 Hz, 1H), 8.40 (dd, *J* = 7.4, 1.2 Hz, 1H), 8.20 (dt, *J* = 8.2, 1.1 Hz, 1H), 7.88 (ddd, *J* = 7.9, 2.4, 1.6 Hz, 1H), 7.71 (dd, *J* = 8.2, 7.4 Hz, 1H), 7.57 – 7.53 (m, 2H), 7.37 (m, 3H), 3.82 (s, 2H), 3.34 (t, *J* = 6.1 Hz, 2H), 2.86 (m, 5H).

$^{13}$C NMR (101 MHz, $CDCl_3$) δ 153.41, 148.60, 148.42, 145.35, 138.08, 136.93, 136.29, 134.40, 134.11, 133.80, 133.52, 131.42, 129.68, 128.91, 127.36, 126.01, 123.72, 117.78, 53.15, 49.64, 46.54, 34.88.

HRMS $[C_{24}H_{24}N_4O_2S+H]^+$: 433.16927 calculated, 433.16907 found.

***N*-(2-(Isoquinoline-5-sulfonamido)ethyl)-4-(pyridin-3-yl)benzamide (93)**

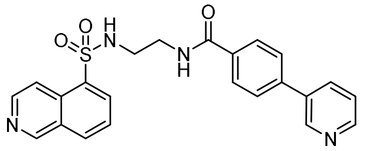

**93** (79 mg, 0.18 mmol, 79%) was synthesized from **114** (100 mg, 0.23 mmol) and pyridin-3-ylboronic acid (30 mg, 0.25 mmol) according to general procedure D.

$^{1}$H NMR (400 MHz, $CDCl_3$) δ 9.23 (s, 1H), 8.70 (d, *J* = 2.2 Hz, 1H), 8.54 (dd, *J* = 4.8, 1.6 Hz, 1H), 8.50 – 8.41 (m, 2H), 8.39 (dd, *J* = 7.4, 1.2 Hz, 1H), 8.08 (d, *J* = 8.2 Hz, 1H), 7.77 (dt, *J* = 8.0, 2.0 Hz, 1H), 7.71 (t, *J* = 8.6 Hz, 3H), 7.60 (t, *J* = 7.8 Hz, 1H), 7.43 – 7.37 (m, 2H), 7.32 (dd, *J* = 8.0, 4.8 Hz, 1H), 3.64 – 3.53 (m, 2H), 3.30 – 3.16 (m, 2H).

$^{13}$C NMR (101 MHz, $CDCl_3$) δ 167.82, 153.19, 148.83, 147.83, 144.78, 140.50, 135.39, 134.63, 134.41, 133.60, 133.25, 133.16, 131.14, 129.02, 127.86, 126.99, 126.06, 123.91, 117.44, 67.10, 42.78, 40.35.

HRMS $[C_{23}H_{20}N_4O_3S+H]^+$: 433.13289 calculated, 433.13252 found.

***N*-(2-((4-(Pyridin-3-yl)benzyl)oxy)ethyl)isoquinoline-5-sulfonamide (94)**

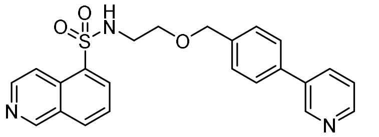

**94** (136 mg, 0.32 mmol, 76%) was synthesized from **119** (0.10 g, 0.44 mmol) and isoquinoline-5-sulfonyl chloride (106 mg, 0.40 µmol) according to general procedure B.

$^{1}$H NMR (400 MHz, $CDCl_3$) δ 9.34 (d, *J* = 1.0 Hz, 1H), 8.80 (dd, *J* = 2.4, 0.9 Hz, 1H), 8.63 (d, *J* = 6.1 Hz, 1H), 8.60 (dd, *J* = 4.8, 1.6 Hz, 1H), 8.48 – 8.42 (m, 2H), 8.17 (dt, *J* = 8.2, 1.1 Hz, 1H), 7.85 (ddd, *J* = 7.9, 2.4, 1.6 Hz, 1H), 7.67 (dd, *J* = 8.2, 7.3 Hz, 1H), 7.49 – 7.43 (m, 2H), 7.37 (ddd, *J* = 7.9, 4.8, 0.9 Hz, 1H), 7.22 (d, *J* = 8.2 Hz, 2H), 6.30 (t, *J* = 5.9 Hz, 1H), 4.33 (s, 2H), 3.46 (t, *J* = 5.0 Hz, 2H), 3.27 – 3.17 (m, 2H).

$^{13}$C NMR (101 MHz, $CDCl_3$) δ 153.36, 148.55, 148.17, 145.17, 137.45, 137.35, 136.22, 134.90, 134.47, 133.56, 133.13, 131.37, 129.11, 128.39, 127.22, 126.00, 123.74, 117.41, 72.79, 68.66, 43.15.

HRMS $[C_{23}H_{21}N_3O_3S +H]^+$: 420.13764 calculated, 420.13728 found.

***N*-(2-(Methyl(4-(pyridin-3-yl)benzyl)amino)ethyl)isoquinoline-5-sulfonamide (95)**

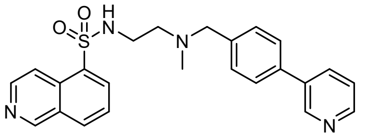

Pyridin-3-ylboronic acid (34 mg, 28 µmol) was subjected to general procedure D with **120** (0.10 g, 0.23 mmol) to provide **95** (45 mg, 0.10 mmol, 45%).

$^1$H NMR (400 MHz, $CDCl_3$) δ 9.32 (d, *J* = 1.0 Hz, 1H), 8.84 (dd, *J* = 2.4, 0.9 Hz, 1H), 8.65 (d, *J* = 6.1 Hz, 1H), 8.60 (dd, *J* = 4.8, 1.6 Hz, 1H), 8.48 – 8.41 (m, 2H), 8.18 (dt, *J* = 8.2, 1.1 Hz, 1H), 7.88 (ddd, *J* = 7.9, 2.4, 1.6 Hz, 1H), 7.68 (dd, *J* = 8.2, 7.3 Hz, 1H), 7.54 – 7.46 (m, 2H), 7.39 (ddd, *J* = 7.9, 4.9, 0.9 Hz, 1H), 7.29 – 7.23 (m, 2H), 3.38 (s, 2H), 3.06 – 2.97 (m, 2H), 2.49 – 2.38 (m, 2H), 1.91 (s, 3H).

$^{13}$C NMR (101 MHz, $CDCl_3$) δ 153.42, 148.57, 148.27, 145.27, 138.08, 136.93, 136.27, 134.41, 134.21, 133.60, 133.42, 131.36, 129.61, 129.08, 127.20, 125.97, 123.72, 117.27, 61.72, 55.03, 41.24, 40.24.

HRMS $[C_{24}H_{24}N_4O_2S+H]^+$: 433.16927 calculated, 433.16896 found.

***N*-(((2*S*,5*R*)-5-(4-(6-Fluoropyridin-3-yl)phenyl)pyrrolidin-2-yl)methyl)isoquinoline-5-sulfonamide (96)**

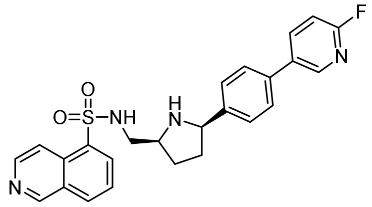


Following general procedure A, **130a** (53 mg, 94 µmol) was reacted with TFA to afford the title compound as a white solid (14 mg, 31 µmol, 33%).

$^1$H NMR (400 MHz, $CDCl_3$) δ 9.37 (s, 1H), 8.71 (d, *J* = 6.1 Hz, 1H), 8.49 – 8.47 (m, 1H), 8.47 (d, *J* = 1.2 Hz, 1H), 8.40 (d, *J* = 2.7 Hz, 1H), 8.22 (d, *J* = 8.2 Hz, 1H), 7.96 (ddd, *J* = 8.5, 7.6, 2.6 Hz, 1H), 7.75 – 7.68 (m, 1H), 7.42 (d, *J* = 8.3 Hz, 2H), 7.33 (d, *J* = 8.1 Hz, 2H), 7.02 (ddd, *J* = 8.5, 3.0, 0.7 Hz, 1H), 4.23 (dd, *J* = 8.7, 6.5 Hz, 1H), 3.50 – 3.42 (m, 1H), 3.04 (dd, *J* = 12.0, 4.2 Hz, 1H), 2.84 (dd, *J* = 12.0, 6.4 Hz, 1H), 2.14 – 2.05 (m, 1H), 1.96 – 1.86 (m, 1H), 1.63 – 1.49 (m, 2H).

$^{13}$C NMR (101 MHz, $CDCl_3$) δ 163.18 (d, *J* = 239 Hz), 153.56, 145.82 (d, *J* = 15 Hz), 145.40, 144.40, 139.76 (d, *J* = 8.0 Hz), 135.55, 134.65 (d, *J* = 4.8 Hz), 134.33, 133.70, 133.52, 131.38, 129.18, 127.18, 127.09, 126.08, 117.29, 109.61 (d, *J* = 37 Hz), 62.19, 56.05, 47.98, 34.48, 28.76.

HRMS $[C_{25}H_{23}FN_4O_2S + H]^+$: 463.15985 calculated, 463.15927 found.

$[\alpha]^{25}_{D}$ +47,9° (c = 1.00, $CHCl_3$).

***N*-(((2*R*,5*R*)-5-(4-(6-Fluoropyridin-3-yl)phenyl)pyrrolidin-2-yl)methyl)isoquinoline-5-sulfonamide (97)**

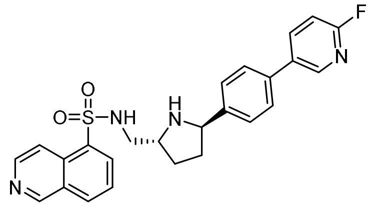


Following general procedure A, **130d** (36 mg, 64 mmol) was reacted with TFA to afford the title compound as a white solid (22 mg, 48 µmol, 74%).

$^1$H NMR (400 MHz, $CDCl_3$) δ 9.37 (d, *J* = 1.1 Hz, 1H), 8.70 (d, *J* = 6.1 Hz, 1H), 8.51 – 8.47 (m, 1H), 8.46 (dd, *J* = 4.7, 1.2 Hz, 1H), 8.39 (dt, *J* = 2.6, 0.9 Hz, 1H), 8.22 (d, *J* = 8.2 Hz, 1H), 7.95 (ddd, *J* = 8.5, 7.6, 2.6 Hz, 1H), 7.72 (dd, *J* = 8.2, 7.4 Hz, 1H), 7.47 (d, *J* = 8.3 Hz, 2H), 7.28 (d, *J* = 6.4 Hz, 2H), 7.01 (ddd, *J* = 8.5, 3.1, 0.7 Hz, 1H), 4.02 (dd, *J* = 8.4, 6.3 Hz, 1H), 3.60 – 3.49 (m, 1H), 3.01 (dd, *J* = 12.3, 4.4 Hz, 1H), 2.79 (dd, *J* = 12.3, 7.9 Hz, 1H), 2.23 – 2.13 (m, 1H), 2.08 – 1.96 (m, 1H), 1.75 – 1.63 (m, 1H), 1.54 – 1.41 (m, 1H).

$^{13}$C NMR (101 MHz, $CDCl_3$) δ 163.22 (d, *J* = 239 Hz), 153.50, 145.83 (d, *J* = 15 Hz), 145.39, 143.87, 139.74 (d, *J* = 8.0 Hz), 135.73, 134.52, 134.48, 133.69, 133.42, 131.39, 129.16, 127.33, 127.15, 126.06, 117.31, 109.62 (d, *J* = 38 Hz), 61.39, 57.24, 47.50, 34.76, 29.47.

HRMS $[C_{25}H_{23}FN_4O_2S + H]^+$: 463.15985 calculated, 463.15925 found.

$[\alpha]^{25}_{D}$ + 29.5° (c = 1.00, $CHCl_3$).

**N-(((2S,5S)-5-(4-(6-Fluoropyridin-3-yl)phenyl)pyrrolidin-2-yl)methyl)isoquinoline-5-sulfonamide (98)**

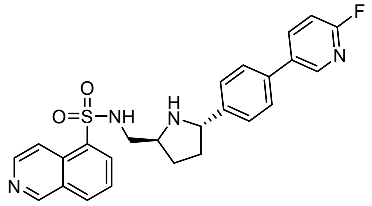

Following general procedure A, **130b** (5.8 mg, 10 µmol) was reacted with TFA to afford the title compound as a white solid (3.4 mg, 7.4 µmol, 71%).

$^{1}$H NMR (500 MHz, $CDCl_3$) δ 9.37 (s, 1H), 8.71 (d, *J* = 6.1 Hz, 1H), 8.47 (dd, *J* = 7.3, 1.2 Hz, 1H), 8.46 – 8.43 (m, 1H), 8.40 (dt, *J* = 2.7, 0.8 Hz, 1H), 8.22 (d, *J* = 8.2 Hz, 1H), 7.95 (ddd, *J* = 8.4, 7.6, 2.6 Hz, 1H), 7.72 (dd, *J* = 8.2, 7.3 Hz, 1H), 7.48 (d, *J* = 8.4 Hz, 2H), 7.28 (d, *J* = 7.9 Hz, 2H), 7.01 (ddd, *J* = 8.5, 3.0, 0.7 Hz, 1H), 4.03 (dd, *J* = 8.4, 6.3 Hz, 1H), 3.58 – 3.52 (m, 1H), 3.01 (dd, *J* = 12.3, 4.4 Hz, 1H), 2.79 (dd, *J* = 12.3, 7.9 Hz, 1H), 2.23 – 2.16 (m, 1H), 2.06 – 1.99 (m, 1H), 1.70 (dq, *J* = 12.5, 8.6 Hz, 1H), 1.53 – 1.44 (m, 1H).

$^{13}$C NMR (126 MHz, $CDCl_3$) δ 163.27 (d, *J* = 239 Hz), 153.53, 145.89 (d, *J* = 15 Hz), 145.48, 143.68, 139.75 (d, *J* = 8.0 Hz), 135.85, 134.52, 134.49, 133.71, 133.44, 131.43, 129.20, 127.39, 127.20, 126.06, 117.32, 109.64 (d, *J* = 38 Hz), 61.48, 57.30, 47.44, 34.72, 29.48.

HRMS $[C_{25}H_{23}FN_4O_2S + H]^+$: 463.15985 calculated, 463.15937 found.

$[\alpha]_D^{25}$ = - 23.5° (c = 0.31, $CHCl_3$).

**N-(((2R,5S)-5-(4-(6-Fluoropyridin-3-yl)phenyl)pyrrolidin-2-yl)methyl)isoquinoline-5-sulfonamide (99)**

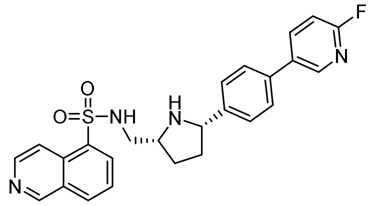

Following general procedure A, **130c** (96 mg, 0.18 mmol) was reacted with TFA to afford the title compound as a white solid (54 mg, 0.12 mmol, 68%).

$^{1}$H NMR (400 MHz, $CDCl_3$) δ 9.37 (s, 1H), 8.69 (d, *J* = 6.1 Hz, 1H), 8.51 – 8.47 (m, 1H), 8.47 – 8.45 (m, 1H), 8.39 (dt, *J* = 2.7, 0.9 Hz, 1H), 8.21 (d, *J* = 8.2 Hz, 1H), 7.96 (ddd, *J* = 8.5, 7.6, 2.6 Hz, 1H), 7.71 (dd, *J* = 8.2, 7.4 Hz, 1H), 7.41 (d, *J* = 8.3 Hz, 2H), 7.32 (d, *J* = 8.0 Hz, 2H), 7.02 (ddd, *J* = 8.5, 3.0, 0.7 Hz, 1H), 4.22 (dd, *J* = 8.6, 6.5 Hz, 1H), 3.46 (ddt, *J* = 8.8, 6.5, 4.4 Hz, 1H), 3.04 (dd, *J* = 12.1, 4.3 Hz, 1H), 2.84 (dd, *J* = 12.0, 6.5 Hz, 1H), 2.14 – 2.05 (m, 1H), 1.97 – 1.84 (m, 1H), 1.63 – 1.46 (m, 2H).

$^{13}$C NMR (101 MHz, $CDCl_3$) δ 163.13 (d, *J* = 239 Hz), 153.52, 145.76 (d, *J* = 15 Hz), 145.32, 144.46, 139.74 (d, *J* = 7.9 Hz), 135.45, 134.63 (d, *J* = 4.8 Hz), 134.33, 133.65, 133.47, 131.35, 129.14, 127.15, 127.03, 126.07, 117.29, 109.58 (d, *J* = 37 Hz), 62.13, 56.07, 48.03, 34.45, 28.74.

HRMS $[C_{25}H_{23}FN_4O_2S + H]^+$: 463.15985 calculated, 463.15918 found.

$[\alpha]_D^{25}$ – 51.2° (c = 1.00, $CHCl_3$).

**N-(((2S,5R)-5-([1,1'-Biphenyl]-4-yl)pyrrolidin-2-yl)methyl)isoquinoline-5-sulfonamide (100)**

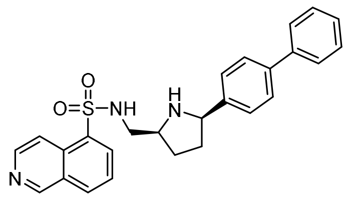

Following general procedure A, **130e** (7.4 mg, 14 µmol) was reacted with TFA to afford the title compound as a white solid (6.4 mg, 9.9 µmol, 73%).

$^{1}$H NMR (850 MHz, $CDCl_3$) δ 9.37 (s, 1H), 8.72 (d, *J* = 6.0 Hz, 1H), 8.47 (d, *J* = 6.1 Hz, 2H), 8.21 (d, *J* = 8.2 Hz, 1H), 7.71 (t, *J* = 7.8 Hz, 1H), 7.58 (d, *J* = 6.9 Hz, 2H), 7.48 (d, *J* = 8.1 Hz, 2H), 7.47 – 7.44 (m, 2H), 7.37 – 7.34 (m, 1H), 7.29 (d, *J* = 8.1 Hz, 2H), 4.22 (dd, *J* = 8.7, 6.5 Hz, 1H), 3.48 – 3.45 (m, 1H), 3.03 (dd, *J* = 12.1, 4.2 Hz, 1H), 2.84 (dd, *J* = 12.1, 6.4 Hz, 1H), 2.11 – 2.07 (m, 1H), 1.93 – 1.88 (m, 1H), 1.61 – 1.55 (m, 2H).

$^{13}$C NMR (214 MHz, $CDCl_3$) δ 153.57, 145.47, 143.05, 140.96, 140.22, 134.31, 133.70, 133.50, 131.39, 129.18, 128.93, 127.37, 127.22, 127.19, 126.83, 126.05, 117.30, 62.36, 55.95, 47.90, 34.39, 28.78.

HRMS $[C_{26}H_{25}N_3O_2S + H]^+$: 444.17402 calculated, 444.17390 found.

**N-(((2S,5R)-5-(4'-Fluoro-[1,1'-biphenyl]-4-yl)pyrrolidin-2-yl)methyl)isoquinoline-5-sulfonamide (101)**

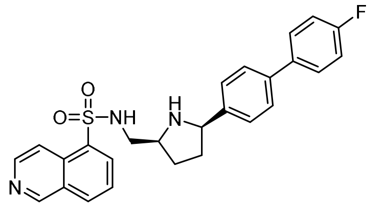

Following general procedure A, **130f** (9.0 mg, 16 µmol) was reacted with TFA to afford the title compound as a white solid (1.5 mg, 3.2 µmol, 20%).

$^{1}$H NMR (400 MHz, $CDCl_3$) δ 9.37 (d, *J* = 1.0 Hz, 1H), 8.71 (d, *J* = 6.1 Hz, 1H), 8.47 (m, 1H), 8.46 (m, 1H), 8.21 (d, *J* = 8.2 Hz, 1H), 7.71 (dd, *J* = 8.2, 7.3 Hz, 1H), 7.58 – 7.48 (m, 2H), 7.45 – 7.39 (m, 2H), 7.31 – 7.24 (m, 2H), 7.18 – 7.09 (m, 2H), 4.20 (dd, *J* = 8.8, 6.5 Hz, 1H), 3.51 – 3.39 (m, 1H), 3.03 (dd, *J* = 12.0, 4.2 Hz, 1H), 2.83 (dd, *J* = 11.9, 6.4 Hz, 1H), 2.13 – 2.03 (m, 1H), 1.97 – 1.82 (m, 1H), 1.63 – 1.51 (m, 2H).

$^{13}$C NMR (101 MHz, $CDCl_3$) δ 162.55 (d, *J* = 246 Hz), 153.56, 145.42, 143.28, 139.17, 137.08 (d, *J* = 3.3 Hz), 134.33, 133.68, 133.51, 131.39, 129.18, 128.69 (d, *J* = 8.0 Hz), 127.05, 126.86, 126.06, 117.30, 115.77 (d, *J* = 21 Hz), 62.27, 55.93, 47.97, 34.44, 28.80.

HRMS [$C_{26}H_{24}FN_3O_2S$ + H]$^+$: 462.16460 calculated, 462.16440 found.

***N*-(((2*S*,5*R*)-5-(4-(Pyridin-3-yl)phenyl)pyrrolidin-2-yl)methyl)isoquinoline-5-sulfonamide (102).**

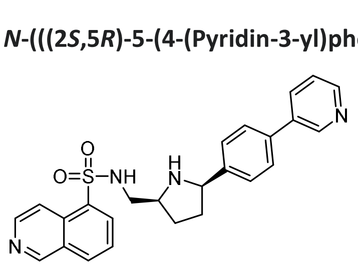

Following general procedure A, **130g** (7.5 mg, 14 µmol) was reacted with TFA to afford the title compound as a white solid (4.2 mg, 9.3 µmol, 68%).

$^{1}$H NMR (850 MHz, $CDCl_3$) δ 9.38 (s, 1H), 8.84 (d, *J* = 2.4 Hz, 1H), 8.71 (d, *J* = 6.0 Hz, 1H), 8.60 (dd, *J* = 4.8, 1.6 Hz, 1H), 8.49 – 8.47 (m, 1H), 8.47 (s, 1H), 8.22 (d, *J* = 8.2 Hz, 1H), 7.89 – 7.86 (m, 1H), 7.72 (t, *J* = 7.5 Hz, 1H), 7.47 (d, *J* = 8.2 Hz, 2H), 7.38 (ddd, *J* = 7.8, 4.8, 0.9 Hz, 1H), 7.35 – 7.32 (m, 2H), 4.23 (dd, *J* = 8.8, 6.5 Hz, 1H), 3.49 – 3.44 (m, 1H), 3.04 (dd, *J* = 12.1, 4.2 Hz, 1H), 2.83 (dd, *J* = 12.1, 6.4 Hz, 1H), 2.13 – 2.08 (m, 1H), 1.94 – 1.88 (m, 1H), 1.60 – 1.54 (m, 2H).

$^{13}$C NMR (214 MHz, $CDCl_3$) δ 153.58, 148.57, 148.38, 145.45, 144.32, 136.73, 136.44, 134.40, 134.32, 133.71, 133.54, 131.39, 129.19, 127.22, 127.13, 126.08, 123.74, 117.29, 62.24, 55.98, 47.99, 34.51, 28.78.

HRMS [$C_{25}H_{24}N_4O_2S$ + H]$^+$: 445.16927 calculated, 445.16913 found.

***N*$^1$-Tritylethane-1,2-diamine (104)**

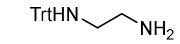

Ethylenediamine (**103**) (267 mL, 4.00 mol) and $K_2CO_3$ (66.3 g, 440 mmol) were suspended in DCM (700 mL) after which a solution of trityl chloride (112 g, 400 mmol) in DCM (700 mL) was added dropwise over 40 min. The reaction-mixture was stirred overnight at RT, filtered, concentrated under reduced pressure and co-evaporated with toluene to yield the product (123 g, quant.) which was used without further purification.

$^{1}$H NMR (400 MHz, $CDCl_3$) δ 7.48 (d, *J* = 7.6 Hz, 6H), 7.26 (t, *J* = 7.7 Hz, 6H), 7.17 (t, *J* = 7.3 Hz, 3H), 2.79 (t, *J* = 5.9 Hz, 2H), 2.21 (t, *J* = 6.0 Hz, 2H), 1.51 (bs, 3H).

$^{13}$C NMR (100 MHz, $CDCl_3$) δ 146.24, 128.76, 127.89, 126.34, 70.77, 46.60, 42.89.

***N*$^1$-(4-Bromobenzyl)-*N*$^2$-tritylethane-1,2-diamine (105)**

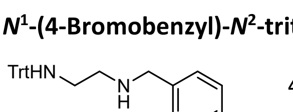

4-Bromobenzaldehyde (0.95 g, 5.1 mmol) was dissolved in methanol (10 mL), and *N*$^1$-tritylethane-1,2-diamine (1.7 g, 5.6 mmol, 1.1 equiv.) was dissolved in THF (5 mL) and added thereto. The mixture was stirred at room temperature for 1 h, after which sodium borohydride (0.29 g, 7.6 mmol, 1.5 equiv.) was slowly added, followed by stirring overnight. Water (40 mL) was added to the mixture and the mixture was extracted with DCM (3× 30 mL). The organic layers were combined, dried with $Na_2SO_4$, filtered and purified by column chromatography (2% → 5% MeOH (10% aq. $NH_3$) in DCM) to yield title compound **105 (**1.04 g, 2.04 mmol, 40%).

**_tert_-Butyl (4-bromobenzyl)(2-(tritylamino)ethyl)carbamate (106)**

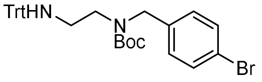


Di-*tert*-butyl-dicarbonate (0.16 g, 0.73 mmol) was dissolved in DCM (1 mL) and slowly added to a mixture of **105** (0.52 g, 1.10 mmol) and triethylamine (0.30 mL, 2.16 mmol) in DCM (5 mL). The reaction mixture was stirred at room temperature overnight, after which it was concentrated *in vacuo*. The residue was purified by column chromatography (5% → 10% EtOAc in pentane) to yield the title product **106 (**0.33 g, 0.65 mmol, 59%).

$^1$H NMR (400 MHz, $CDCl_3$) δ 7.57 – 6.94 (m, 19H), 4.34 (s, 2H), 3.42 – 3.19 (m, 2H), 2.36 – 2.14 (m, 2H), 1.64 (bs, 1H), 1.54 – 1.32 (m, 9H).

$^{13}$C NMR (101 MHz, $CDCl_3$) δ 156.21, 146.02, 137.50, 131.68, 128.63, 127.96, 127.35, 126.41, 121.28, 80.10, 70.86, 50.16, 47.43, 42.22, 28.51.

**_tert_-Butyl (4-(pyridin-3-yl)benzyl)(2-(tritylamino)ethyl)carbamate (107)**

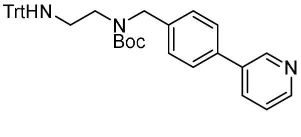


The mixture of **106** (0.30 g, 0.52 mmol), 3-pyridinylboronic acid (86 mg, 0.70 mmol), $Pd(PPh_3)_4$ (30 mg, 26 µmol), potassium carbonate (0.31 g, 2.2 mmol) in water (2 mL) and 1,4-dioxane (6 mL) was deoxygenated under nitrogen flow and sealed. The mixture was heated to 90°C and stirred overnight. The reaction mixture was then filtrated, concentrated and was purified by column chromatography (30% EtOAc in pentane) to yield **107** (0.23 g, 0.40 mmol, 76%).

$^1$H NMR (400 MHz, $CDCl_3$) δ 8.82 (s, 1H), 8.58 (dd, *J* = 4.8, 1.7 Hz, 1H), 7.90 – 7.73 (m, 1H), 7.59 – 7.09 (m, 21H), 4.47 (s, 2H), 3.47 – 3.20 (m, 2H), 2.39 – 2.17 (m, 2H), 1.59 – 1.34 (m, 9H).

$^{13}$C NMR (101 MHz, $CDCl_3$) δ 156.11, 148.56, 148.35, 146.05, 138.61, 136.79, 136.37, 134.35, 128.65, 127.94, 127.36, 126.39, 123.67, 80.04, 70.87, 50.35, 47.50, 42.25, 28.54.

**_tert_-Butyl (2-aminoethyl)(4-(pyridin-3-yl)benzyl)carbamate (108)**

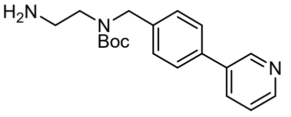


To a solution of **107** (0.20 g, 0.35 mmol) and triethylsilane (0.40 mL, 2.5 mmol) in DCM (10 mL) on ice bath, was added TFA (0.15 mL). The reaction mixture was stirred at room temperature overnight, after which it was basified by adding sat. aq. $Na_2CO_3$ (10 mL), extracted with DCM (3× 25 mL). The combined organic layers were dried over $Na_2SO_4$, filtered and purified by column chromatography (5% MeOH (10% aq. $NH_3$) in DCM) to yield **108 (**0.10 g, 0.32 mmol, 91%).

$^1$H NMR (400 MHz, $CDCl_3$) δ 8.84 (d, *J* = 2.4 Hz, 1H), 8.58 (dd, *J* = 4.8, 1.7 Hz, 1H), 7.87 (dt, *J* = 7.9, 2.0 Hz, 1H), 7.55 (d, *J* = 8.1 Hz, 2H), 7.42 – 7.31 (m, 3H), 4.53 (s, 2H), 3.30 (d, *J* = 27.2 Hz, 2H), 2.97 – 2.68 (m, 2H), 1.47 (s, 9H).

$^{13}$C NMR (101 MHz, $CDCl_3$) δ 156.20, 148.51, 148.27, 138.59, 136.80, 136.26, 134.28, 128.46, 127.33, 123.61, 80.10, 50.89, 50.06, 40.57, 28.49.

**_tert_-Butyl (2-(isoquinoline-5-sulfonamido)ethyl)(4-(pyridin-3-yl)benzyl)carbamate (109)**

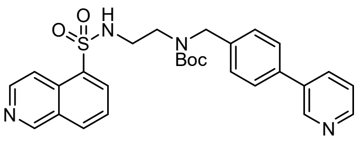


**109** (100 mg, 0.19 mmol, 83%) was synthesized from **108** (79 mg, 0.23 mmol) according to general procedure B.

$^1$H NMR (400 MHz, $CDCl_3$) δ 9.36 – 9.30 (m, 1H), 8.81 (s, 1H), 8.63 – 8.55 (m, 2H), 8.43 (d, *J* = 6.1 Hz, 1H), 8.38 (dd, *J* = 7.4, 1.2 Hz, 1H), 8.16 (d, *J* = 8.2 Hz, 1H), 7.86 (d, *J* = 8.0 Hz, 1H), 7.64 (t, *J* = 8.2 Hz, 1H), 7.46 (d, *J* = 8.6 Hz, 2H), 7.39 (dd, *J* = 8.0, 4.8 Hz, 1H), 7.22 (dd, *J* = 8.2, 3.2 Hz, 2H), 4.39 (s, 2H), 3.41 – 3.24 (m, 2H), 3.13 – 2.99 (m, 2H), 1.43 (s, 9H).

$^{13}$C NMR (101 MHz, $CDCl_3$) δ 156.57, 153.18, 148.34, 147.93, 144.90, 137.92, 136.71, 136.11, 134.55, 134.35, 133.36, 133.00, 131.19, 129.03, 127.90, 127.24, 125.88, 123.70, 117.37, 51.37, 46.66, 42.19, 28.26.

**_N_-(2-Aminoethyl)isoquinoline-5-sulfonamide (111)**

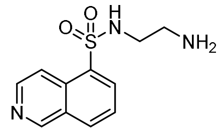

Isoquinoline-5-sulfonic acid (**110**) (10.0 g, 47.8 mmol) was dissolved in $SOCl_2$ (60 mL) and DMF (1.2 mL). The mixture was refluxed at 60°C until TLC analysis showed the complete conversion of the starting material. The $SOCl_2$ was evaporated *in vacuo*, and the reaction mixture was washed with DCM and then filtered. The crude sulfonyl chloride formed was immediately used in the following reaction.

Ethylene diamine (15.1 mL, 227 mmol) was added dropwise to a cooled (0°C) and stirred solution of the crude sulfonyl chloride (10.0 g, 37.7 mmol) in DCM (600 mL). The mixture was then stirred at room temperature for 2 h. The reaction mixture was diluted with sat. aq. $Na_2CO_3$ (10 mL), washed with brine (50 mL) and extracted with DCM (3×). The organic layers were combined, dried over $MgSO_4$, filtered and concentrated. The residue was then co-evaporated with toluene to remove the remaining ethylene diamine giving **111** (10.3 g, 35.0 mmol, 93%) as a dark yellow solid that was used without further purification.

***N*-(2-((4-Bromobenzyl)amino)ethyl)isoquinoline-5-sulfonamide (112a)**

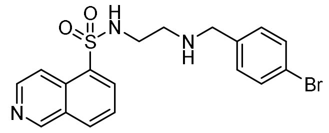

**111** (0.10 g, 3.96 mmol) and 4-bromobenzaldehyde (0.36 g, 1.93 mmol) were dissolved in THF (20 mL) in the presence of activated 3 Å molecular sieves. Then, sodium triacetoxyborohydride (0.84 g, 3.96 mmol) and glacial acetic acid (110 µL, 1.93 mmol) were added. The reaction mixture was stirred overnight, after which sat. aq. $Na_2CO_3$ (5 mL) was added to quench the reaction. The mixture was then diluted with brine (5 mL), extracted with $Et_2O$ (10 mL) and DCM (3×). The combined organic layers were dried over $MgSO_4$, filtered and concentrated *in vacuo*. The crude product purified by column chromatography (1% → 10% MeOH (10% aq. $NH_3$) in DCM) to give **112A** (0.64 g, 1.52 mmol, 79%).

$^1$H NMR (400 MHz, $CDCl_3$) δ 9.35 (d, *J* = 1.0 Hz, 1H), 8.65 (d, *J* = 6.1 Hz, 1H), 8.45 – 8.40 (m, 2H), 8.20 (dt, *J* = 8.2, 1.2 Hz, 1H), 7.69 (dd, *J* = 8.2, 7.4 Hz, 1H), 7.38 – 7.31 (m, 2H), 7.00 – 6.95 (m, 2H), 3.49 (s, 2H), 3.02 – 2.96 (m, 2H), 2.65 – 2.59 (m, 2H).

$^{13}$C NMR (101 MHz, $CDCl_3$) δ 153.46, 145.24, 138.59, 134.28, 133.70, 133.44, 131.59, 131.30, 129.67, 129.10, 126.05, 121.01, 117.26, 52.53, 47.42, 42.54.

***N*-(2-(((6-Bromopyridin-3-yl)methyl)amino)ethyl)isoquinoline-5-sulfonamide (112b)**

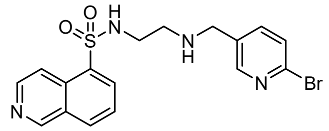

**111** (1.00 g, 3.98 mmol) and 6-bromonicotinaldehyde (0.36 g, 1.94 mmol) were dissolved in THF (20 mL) in the presence of activated 3 Å molecular sieves. Then, sodium triacetoxyborohydride (0.84 g, 3.98 mmol) and glacial acetic acid (122 µL, 1.94 mmol) were added. The reaction mixture was stirred overnight. Sat. aq. $Na_2CO_3$ (5 mL) was added to the reaction, which was then diluted with brine (10 mL) and extracted with $Et_2O$ (10 mL) and DCM (3×). The organic layers were combined, dried over $MgSO_4$, filtered and concentrated *in vacuo*. The crude product was purified by column chromatography (1% → 10% MeOH (10% aq. $NH_3$) in DCM) to afford title compound **112b** (0.73 g, 1.73 mmol, 89%).

$^1$H NMR (400 MHz, $CDCl_3$) δ 9.39 (bs, 1H), 8.73 – 7.70 (m, 1H), 8.51 – 8.41 (m, 2H), 8.26 – 8.21 (m, 1H), 8.19 – 8.16 (m, 1H), 7.78 – 7.68 (m, 1H), 7.49 – 7.36 (m, 2H), 3.59 (s, 2H), 3.13 – 3.00 (m, 2H), 2.72 – 2.65 (m, 2H).

***N*-(2-((4-Bromo-3-chlorobenzyl)amino)ethyl)isoquinoline-5-sulfonamide (112c)**

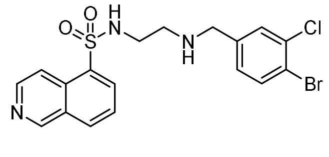

**111** (1.00 g, 3.98 mmol) and 3-chloro-4-bromoaldehyde (0.43 g, 1.94 mmol) were dissolved in THF (20 mL) in the presence of activated 3 Å molecular sieves. Then, sodium triacetoxyborohydride (0.84 g, 3.98 mmol) and glacial acetic acid (122 µL, 1.94 mmol) were added. The reaction mixture was stirred overnight. Sat. aq. $Na_2CO_3$ (5 mL) was added to quench the reaction, which was then diluted with brine (10 mL) and extracted with $Et_2O$ (10 mL) and DCM (3×). The organic layers were combined, dried over $MgSO_4$, filtered and

concentrated *in vacuo*. The crude product was purified by column chromatography (1% → 10% MeOH (10% aq. $NH_3$) in DCM) to afford title compound **112c** (0.8 g, 1.76 mmol, 91%).

$^1$H NMR (400 MHz, $CDCl_3$) δ 9.39 (bs, 1H), 8.77 – 8.67 (m, 1H), 8.51 – 8.41 (m, 2H), 8.26 – 8.22 (m, 1H), 7.78 – 7.67 (m, 1H), 7.55 – 7.50 (m, 1H), 7.29 – 7.27 (m, 1H), 6.93 – 6.89 (m, 1H), 3.53 (s, 2H), 3.13 – 2.99 (m, 2H), 2.70 – 2.62 (m, 2H).

**_N_-(2-((4-Bromo-3-fluorobenzyl)amino)ethyl)isoquinoline-5-sulfonamide (112d)**

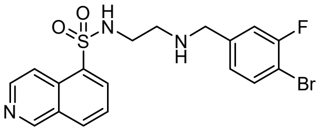


**111** (1.00 g, 3.98 mmol) and 3-fluoro-4-bromoaldehyde (0.39 g, 1.9 mmol) were dissolved in THF (20 mL) in the presence of activated 3 Å molecular sieves. Then, sodium triacetoxyborohydride (0.84 g, 4.0 mmol) and glacial acetic acid (122 µL, 1.94 mmol) were added. The reaction mixture was stirred overnight. Sat. aq. $Na_2CO_3$ (5 mL) was added to the reaction, which was then diluted with brine (10 mL) and extracted with $Et_2O$ (10 mL) and DCM (3×). The organic layers were combined, dried over $MgSO_4$, filtered and concentrated *in vacuo*. The crude product was purified by column chromatography (1% → 10% MeOH (10% aq. $NH_3$) in DCM) to afford title compound **112d** (0.62 g, 1.4 mmol, 73%).

$^1$H NMR (400 MHz, $CDCl_3$) δ 9.39 (bs, 1H), 8.74 – 8.70 (m, 1H), 8.50 – 8.39 (m, 2H), 8.26 – 8.21 (m, 1H), 7.75 – 7.70 (m, 1H), 7.48 – 7.43 (m, 1H), 6.99 – 6.94 (m, 1H), 6.86 – 6.81 (m, 1H), 3.58 (s, 2H), 3.10 – 3.00 (m, 2H), 2.71 – 2.64 (m, 2H).

***tert*-Butyl (4-bromobenzyl)(2-(isoquinoline-5-sulfonamido)ethyl)carbamate (113a)**

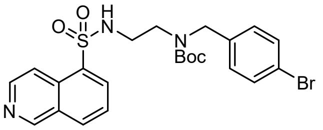


**112a** (2.26 g, 5.38 mmol) and $NaHCO_3$ (500 mg, 5.92 mmol) were suspended in THF (15 mL) and cooled to 0°C. $Boc_2O$ (1.26 g, 5.92 mmol) was then carefully added to this mixture, followed by 6 h of stirring. The reaction was diluted with sat. aq. $Na_2CO_3$ (5 mL), followed by dilution with brine (10 mL) and extraction with DCM (3×). The combined organic layers were dried over $MgSO_4$, filtered and concentrated *in vacuo*. The crude product was purified by column chromatography (20% → 40% EtOAc in pentane) to give title compound **113a** (2.42 g, 4.65 mmol, 86%).

$^1$H NMR (400 MHz, $CDCl_3$) δ 9.37 (s, 1H), 8.66 (d, *J* = 6.1 Hz, 1H), 8.45 – 8.32 (m, 2H), 8.21 (d, *J* = 8.2 Hz, 1H), 7.69 (t, *J* = 7.8 Hz, 1H), 7.39 – 7.30 (m, 2H), 6.96 (d, *J* = 8.2 Hz, 2H), 4.27 (s, 2H), 3.39 – 3.20 (m, 2H), 3.11 – 2.89 (m, 2H), 1.43 (s, 9H).

$^{13}$C NMR (101 MHz, $CDCl_3$) δ 154.93, 153.37, 145.25, 136.89, 133.64, 133.29, 131.83, 131.32, 129.17, 128.94, 126.00, 121.41, 117.41, 81.25, 51.35, 46.77, 42.63, 28.43.

***tert*-Butyl ((6-bromopyridin-3-yl)methyl)(2-(isoquinoline-5-sulfonamido)ethyl)carbamate (113b)**

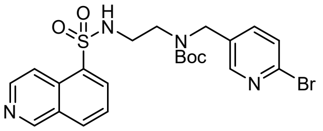


**112b** (0.73 g, 1.7 mmol) and $NaHCO_3$ (0.16 g, 1.9 mmol) were dissolved in THF (10 mL) and cooled to 0°C. $Boc_2O$ (0.4 g, 1.9 mmol) was carefully added and the mixture was stirred overnight. The reaction was quenched with sat. aq. $Na_2CO_3$ (5 mL), washed with brine (10 mL) and extracted with DCM (3×). The organic layers were combined, dried over $MgSO_4$, filtered and concentrated *in vacuo*. This yielded crude product **113b** (1.1 g, 1.73 mmol, 100%). which was directly used in the next step.

***tert*-Butyl ((6-bromopyridin-3-yl)methyl)(2-(isoquinoline-5-sulfonamido)ethyl)carbamate (113c)**

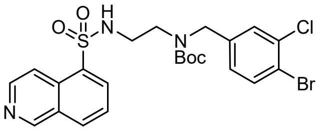


**112C** (0.8 g, 1.76 mmol) and $NaHCO_3$ (0.16 g, 1.94 mmol) were dissolved in THF (10 mL) and cooled to 0°C $Boc_2O$ (0.4 g, 1.9 mmol) was carefully added and the mixture was stirred overnight. The reaction was diluted with sat. aq. $Na_2CO_3$ (5 mL), washed with brine (10 mL) and extracted with DCM (3×). The organic layers were combined, dried over

MgSO4, filtered and concentrated *in vacuo*. This yielded crude product **113c** (0.98 g, 1.76 mmol, 100%) which was directly used in the next step.

***tert*-Butyl ((6-bromopyridin-3-yl)methyl)(2-(isoquinoline-5-sulfonamido)ethyl)carbamate (113d)**

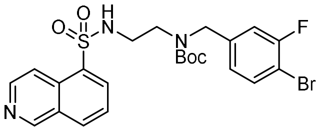


**112d** (0.60 g, 1.4 mmol) and $NaHCO_3$ (0.16 g, 1.9 mmol) were dissolved in THF (10 mL) and cooled to 0°C. $Boc_2O$ (0.4 g, 1.9 mmol) was carefully added and the mixture was stirred overnight. The reaction was diluted with sat. aq. $Na_2CO_3$ (5 mL), washed with brine (10 mL) and extracted with DCM (3×). The organic layers were combined, dried over $MgSO_4$, filtered and concentrated *in vacuo*. This yielded crude product **113d** (1.0 g, 1.4 mmol, 100%) which was directly used in the next step.

**4-Bromo-*N*-(2-(isoquinoline-5-sulfonamido)ethyl)benzamide (114)**

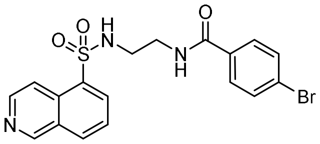


To a mixture of **111** (0.25 g, 1.0 mmol), 4-bromobenzonic acid (0.26 g, 1.3 mmol) and HATU (0.38 g, 1.0 mmol) in DCM (30 mL) was added DIPEA (0.18 mL, 1.0 mmol), followed by stirring overnight. The reaction mixture was then washed with brine, and the organic layer was dried over $Na_2SO_4$, filtered and purified by column chromatography (3% MeOH (10% aq. $NH_3$) in DCM) to yield title compound **114** (0.20 g, 0.43 mmol, 43%).

$^1$H NMR (400 MHz, $CDCl_3$) δ 9.25 (s, 1H), 8.56 (d, *J* = 6.2 Hz, 1H), 8.42 – 8.36 (m, 2H), 8.14 (d, *J* = 8.2 Hz, 1H), 7.66 (t, *J* = 7.8 Hz, 1H), 7.52 – 7.46 (m, 2H), 7.46 – 7.39 (m, 2H), 7.13 (t, *J* = 5.7 Hz, 1H), 3.76 – 3.63 (m, 2H), 3.54 – 3.48 (m, 2H), 3.23 – 3.18 (m, 2H).

$^{13}$C NMR (101 MHz, $CDCl_3$) δ 167.62, 153.25, 145.03, 134.15, 133.84, 133.37, 132.40, 131.81, 131.18, 129.10, 128.68, 126.51, 126.15, 117.42, 43.60, 40.12.

**2- (Tritylamino)ethan-1-ol (116)**

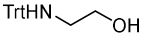


To a solution of trityl chloride (1.4 g, 5.0 mmol) and $K_2CO_3$ (0.76 g, 5.5 mmol) in DCM (17 mL) at 0 °C was added dropwise ethanolamine (**115**) (1.5 mL, 25 mmol). The reaction was allowed to warm to room temperature and stirred for 3 h before sat. aq. $NaHCO_3$ (15 mL) and $H_2O$ (15 mL) were added. The organic layer was collected and the aqueous layer extracted with DCM (3× 30 mL). The combined organic layers were dried over $Na_2SO_4$, filtered and concentrated under reduced pressure. The residue was purified by column chromatography (20% EtOAc in pentane) to yield **116** (1.5 g, 4.95 mmol, 99%).

$^1$H NMR (400 MHz, $CDCl_3$) δ 7.65 – 7.52 (m, 6H), 7.42 – 7.31 (m, 6H), 7.27 (t, *J* = 7.3 Hz, 3H), 3.71 (t, *J* = 5.3 Hz, 2H), 2.69 (bs, 1H), 2.41 (t, *J* = 5.3 Hz, 2H), 2.06 (bs, 1H).

$^{13}$C NMR (101 MHz, $CDCl_3$) δ 145.88, 128.62, 127.85, 126.34, 70.55, 62.47, 45.60.

**2-((4-Bromobenzyl)oxy)-*N*-tritylethan-1-amine (117)**

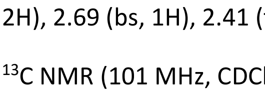


A solution of **116** (1.5 g, 4.95 mmol) in DMF (4 mL) was cooled on ice, and carefully NaH (60% in oil, 0.25 g, 6.25 mmol) was added. 4-Bromo-benzylbromide (0.92 g, 3.3 mmol) was added. The reaction mixture was stirred at room temperature overnight, after which the reaction was quenched with water (10 mL). The mixture was extracted with EtOAc (3× 10 mL). The combined organic layers were dried over $Na_2SO_4$, filtered and concentrated under reduced pressure. The residue was purified by column chromatography (20% EtOAc in pentane) to yield title compound **117** (0.78 g, 1.65 mmol, 50%).

$^1$H NMR (400 MHz, $CDCl_3$) δ 7.51 – 7.41 (m, 8H), 7.29 – 7.22 (m, 6H), 7.20 – 7.12 (m, 5H), 4.37 (s, 2H), 3.58 (t, *J* = 5.3 Hz, 2H), 2.37 (t, *J* = 5.3 Hz, 2H).

$^{13}$C NMR (101 MHz, $CDCl_3$) δ 146.17, 137.60, 131.56, 129.30, 128.78, 127.93, 126.38, 121.47, 72.10, 70.76, 70.60, 43.32.

**2-((4-(Pyridin-3-yl)benzyl)oxy)-*N*-tritylethan-1-amine (118)**

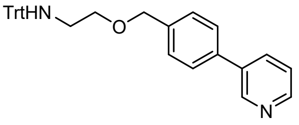

**118** (400 mg, 0.85 mmol, 51%) was synthesized from **117** (780 mg, 1.65 mmol) according to general procedure D.

$^1$H NMR (400 MHz, $CDCl_3$) δ 8.84 (dd, *J* = 2.3, 0.9 Hz, 1H), 8.58 (dd, *J* = 4.8, 1.6 Hz, 1H), 7.86 (ddd, *J* = 7.9, 2.4, 1.6 Hz, 1H), 7.60 – 7.53 (m, 2H), 7.52 – 7.46 (m, 6H), 7.44 – 7.38 (m, 2H), 7.35 (ddd, *J* = 7.9, 4.8, 0.9 Hz, 1H), 7.30 – 7.23 (m, 6H), 7.20 – 7.15 (m, 3H), 4.50 (s, 2H), 3.65 (t, *J* = 5.3 Hz, 2H), 2.41 (t, *J* = 5.3 Hz, 2H).

$^{13}$C NMR (101 MHz, $CDCl_3$) δ 148.51, 148.32, 146.17, 138.63, 137.13, 136.45, 134.38, 128.77, 128.34, 127.88, 127.21, 126.33, 123.64, 72.42, 70.75, 70.64, 43.33.

**2-((4-(Pyridin-3-yl)benzyl)oxy)ethan-1-amine (119)**

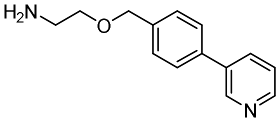

**119** (100 mg, 0.44 mmol, 54%) was synthesized from **118** (380 mg, 0.81 mmol) according to general procedure A.

$^1$H NMR (400 MHz, $CDCl_3$) δ 8.84 (dd, *J* = 2.4, 0.9 Hz, 1H), 8.59 (dd, *J* = 4.8, 1.6 Hz, 1H), 7.87 (ddd, *J* = 7.9, 2.4, 1.6 Hz, 1H), 7.61 – 7.55 (m, 2H), 7.50 – 7.44 (m, 2H), 7.37 (ddd, *J* = 7.9, 4.8, 0.9 Hz, 1H), 4.60 (s, 2H), 3.57 (t, *J* = 5.3 Hz, 2H), 2.93 (t, *J* = 5.3 Hz, 2H).

$^{13}$C NMR (101 MHz, $CDCl_3$) δ 148.56, 148.35, 138.44, 137.28, 136.42, 134.40, 128.50, 127.29, 123.66, 72.79, 72.66, 41.99.

***N*-(2-((4-Bromobenzyl)(methyl)amino)ethyl)isoquinoline-5-sulfonamide (120)**

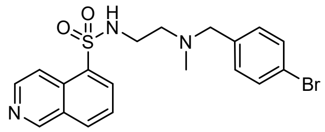

**112a** (0.58 g, 1.4 mmol), formaldehyde (46 mg, 1.5 mmol) and sodium triacetoxyborohydride (0.59 g, 2.8 mmol) were suspended in THF (15 mL) and MeOH (2.5 mL). The reaction mixture was stirred at room temperature overnight, after which it was diluted with sat. aq. $NaHCO_3$ (10 mL) and extracted with DCM (3×). The organic layers were combined, dried over $MgSO_4$, filtered and concentrated. The crude product was purified by column chromatography (1% → 10% MeOH (10% aq. $NH_3$) in DCM) to give title compound **120** (0.38 g, 0.88 mmol, 64%).

**(*S*)-2-(((Triisopropylsilyl)oxy)methyl)pyrrolidine (122a)**

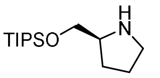

Following general procedure E, L-prolinol (**121a**) (5.00 g, 49.4 mmol) was reacted with TIPS-Cl (12.7 mL, 59.3 mmol), imidazole (6.73 g, 99.0 mmol) and DMAP, (0.302 g, 2.47 mmol) to afford the title compound as a colourless oil (2.89 g, 11.2 mmol, 23%) which was stored at -20°C to avoid degradation.

$^1$H NMR (400 MHz, $CDCl_3$) δ 3.71 – 3.61 (m, 2H), 3.23 – 3.13 (m, 1H), 3.03 – 2.98 (m, 1H), 2.87 – 2.87 (m, 1H), 2.05 (s, 1H), 1.82 – 1.70 (m, 2H), 1.54 – 1.46 (m, 1H), 1.13 – 1.04 (m, 21H).

$^{13}$C NMR (101 MHz, $CDCl_3$) δ 65.92, 60.19, 46.50, 27.35, 25.41, 17.90, 11.87.

**(*R*)-2-(((Triisopropylsilyl)oxy)methyl)pyrrolidine (122b)**

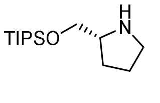

Following general procedure E, R-prolinol (**121b**) (3.00 g, 29.7 mmol) was reacted with TIPS-Cl (7.62 mL, 35.6 mmol), imidazole (4.04 g, 59.3 mmol) and DMAP (0.181 g, 1.48 mmol) to afford the title compound as a colourless oil (2.40 g, 9.30 mmol, 31%) which was stored at -20°C to avoid degradation.

$^1$H NMR (300 MHz, $CDCl_3$) δ 3.78 – 3.56 (m, 2H), 3.26 – 3.09 (m, 1H), 3.09 – 2.93 (m, 1H), 2.93 – 2.76 (m, 1H), 2.02 (s, 1H), 1.88 – 1.61 (m, 3H), 1.60 – 1.41 (m, 1H), 1.21 – 0.91 (m, 21H).

$^{13}$C NMR (75 MHz, $CDCl_3$) δ 66.00, 60.27, 46.58, 27.43, 25.49, 17.99, 11.94.

**(2*R*,5*S*)-2-(4-Bromophenyl)-5-(((triisopropylsilyl)oxy)methyl)pyrrolidine (123a)**

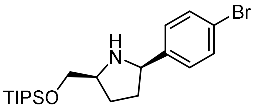


Following general procedure F, **122a** (1.57 g, 6.09 mmol) was reacted with *n*-BuLi (1.6 M in hexanes, 3.8 mL, 6.1 mmol), benzophenone (1.33 g, 7.31 mmol) and bromobenzenelithium (9.13 mmol, prepared according to general procedure G) to afford the title compound as a crude mixture with benzhydrol (1.5 g crude, 35% purity, 1.6 mmol product, 26%).

$^1$H NMR (400 MHz, $CDCl_3$) δ 7.39 – 7.05 (m, 4H + benzhydrol-Ar), 3.89 – 3.83 (m, 2H), 3.70 – 3.61 (m, 2H), 3.12 (tt, *J* = 7.6, 4.2 Hz, 1H), 2.07 – 1.97 (m, 1H), 1.79 – 1.68 (m, 2H), 1.60 – 1.50 (m, 1H), 1.07 – 1.00 (m, 21H).

$^{13}$C NMR (101 MHz, $CDCl_3$) δ 142.78, 131.38, 128.29 – 61 (+ benzhydrol-Ar), 75.78, 65.26, 62.11, 60.26, 34.26, 27.79, 18.02, 11.90.

**(2*S*,5*S*)-2-(4-Bromophenyl)-5-(((triisopropylsilyl)oxy)methyl)pyrrolidine (123b)**

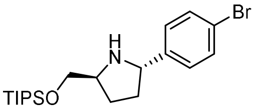


Following general procedure F, **122a** (1.57 g, 6.09 mmol) was reacted with *n*-BuLi (1.6 M in hexanes, 3.8 mL, 6.1 mmol), benzophenone (1.33 g, 7.31 mmol) and bromobenzenelithium (9.13 mmol, prepared according to general procedure G) to afford the title compound as a crude mixture with benzophenone (0.35 g crude, 60% purity, 0.49 mmol product, 8%).

$^1$H NMR (400 MHz, $CDCl_3$) δ 7.41 – 7.13 (m, 4H + benzophenone-Ar), 4.23 – 4.15 (m, 1H), 3.63 (dd, *J* = 5.7, 1.7 Hz, 2H), 3.53 – 3.45 (m, 1H), 2.21 – 2.12 (m, 1H), 2.03 – 1.92 (m, 1H), 1.66 – 1.53 (m, 2H), 1.09 – 1.04 (m, 21H).

$^{13}$C NMR (101 MHz, $CDCl_3$) δ 144.45, 131.38-127.46 (+ benzophenone-Ar), 120.37, 66.05, 60.42, 59.86, 35.09, 27.70, 18.13, 12.04.

**(2*S*,5*R*)-2-(4-Bromophenyl)-5-(((triisopropylsilyl)oxy)methyl)pyrrolidine (123c)**

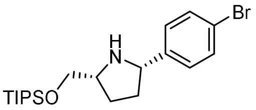


Following general procedure F, **122b** (2.39 g, 9.28 mmol) was reacted with *n*-BuLi (1.6 M in hexanes, 5.8 mL, 9.3 mmol), benzophenone (2.03 g, 11.1 mmol) and bromobenzenelithium (13.9 mmol, prepared according to general procedure G) to afford the title compound as a crude mixture with benzhydrol (2.15 g crude, 50% purity, 2.61 mmol product, 28%).

$^1$H NMR (300 MHz, $CDCl_3$) δ 7.41– 7.13 (m, 4H + benzhydrol-Ar), 4.08 – 3.87 (m, 1H), 3.79 – 3.57 (m, 2H), 3.32 – 3.11 (m, 2H), 2.16 – 1.98 (m, 1H), 1.90 – 1.67 (m, 2H), 1.67 – 1.47 (m, 1H), 1.18 – 0.93 (m, 21H).

$^{13}$C NMR (75 MHz, $CDCl_3$) δ 143.13, 130.44, 127.50-125.63 (+ benzhydrol-Ar), 126.49, 119.59, 65.14, 61.23, 59.48, 33.43, 26.85, 17.14, 11.05.

**(2*R*,5*R*)-2-(4-Bromophenyl)-5-(((triisopropylsilyl)oxy)methyl)pyrrolidine (123d)**

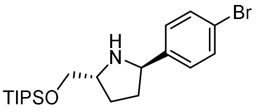

Following general procedure F, **122b** (2.39 g, 9.28 mmol) was reacted with *n*-BuLi (1.6 M in hexanes, 5.8 mL, 9.3 mmol), benzophenone (2.03 g, 11.1 mmol) and bromobenzenelithium (13.9 mmol, prepared according to general procedure G) to afford the title compound as a crude mixture with benzophenone (0.430 g crude, 60% purity, 0.625 mmol product, 7%). $^{1}$H NMR (300 MHz, $CDCl_3$) δ 7.43 – 7.21 (m, 4H + benzophenone-Ar), 4.26 – 4.15 (m, 1H), 3.68 – 3.58 (m, 2H), 3.56 – 3.46 (m, 1H), 2.21 – 2.13 (m, 1H), 2.03 – 1.93 (m, 1H), 1.74 – 1.53 (m, 2H), 1.08 – 1.04 (m, 21H).

$^{13}$C NMR (75 MHz, $CDCl_3$) δ 143.60, 130.61-125.14 (+ benzophenone-Ar), 119.36, 65.12, 59.43, 58.92, 34.16, 26.73, 17.15, 11.07.

***tert*-Butyl (2*R*,5*S*)-2-(4-bromophenyl)-5-(((triisopropylsilyl)oxy)methyl)pyrrolidine-1-carboxylate (124a)**

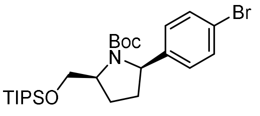

Following general procedure H, **123a** (0.85 g crude, 35% purity, 0.91 mmol product) was reacted with $Boc_2O$ (494 mg, 2.26 mmol) and triethylamine (0.252 mL, 1.81 mmol) for 1.5 h to afford the title compound as a colourless oil (0.315 g, 0.615 mmol, 68%).

$^{1}$H NMR (500 MHz, 333 K, $CDCl_3$) δ 7.38 (d, *J* = 8.4 Hz, 2H), 7.16 (d, *J* = 8.4 Hz, 2H), 4.69 (bs, 1H), 4.07 – 3.99 (m, 1H), 3.98 – 3.76 (m, 2H), 2.25 – 2.15 (m, 1H), 2.13 – 2.04 (m, 1H), 1.99 – 1.91 (m, 1H), 1.91 – 1.83 (m, 1H), 1.32 – 1.23 (m, 9H), 1.10 – 1.05 (m, 21H).

$^{13}$C NMR (126 MHz, 333 K, $CDCl_3$) δ 155.22, 144.18, 131.36, 127.76, 120.25, 79.79, 64.84, 62.84, 60.89, 34.60, 28.48, 27.21, 18.22, 12.35.

***tert*-Butyl (2*S*,5*S*)-2-(4-bromophenyl)-5-(((triisopropylsilyl)oxy)methyl)pyrrolidine-1-carboxylate (124b)**

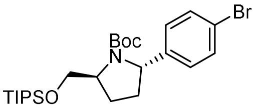

Following general procedure H, **123b** (0.16 g crude, 60% purity, 0.23 mmol product) was reacted with $Boc_2O$ (36 mg, 0.16 mmol) and triethylamine (18 µL, 0.13 mmol) for 4.5 h to afford the title compound as a colourless oil (45 mg, 65 µmol, 28%).

Rotamer equilibrium (3:2).

Major rotamer $^{1}$H NMR (300 MHz, $CDCl_3$) δ 7.41 (d, *J* = 8.5 Hz, 2H), 7.00 (d, *J* = 8.4 Hz, 2H), 4.79 (d, *J* = 8.5 Hz, 1H), 4.18 (ddt, *J* = 7.7, 5.2, 2.6 Hz, 1H), 3.98 (dd, *J* = 9.8, 5.2 Hz, 1H), 3.80 (dd, *J* = 9.8, 2.8 Hz, 1H), 2.66 – 2.50 (m, 1H), 2.10 – 1.90 (m, 2H), 1.60 (dd, *J* = 12.5, 6.1 Hz, 1H), 1.15 (s, 9H), 1.11 – 1.03 (m, 21H).

Major rotamer $^{13}$C NMR (75 MHz, $CDCl_3$) δ 153.98, 145.26, 131.28, 127.12, 120.02, 79.33, 63.61, 61.95, 59.77, 33.46, 28.24, 25.18, 18.14, 12.10.

Minor rotamer $^{1}$H NMR (300 MHz, $CDCl_3$) δ 7.41 (d, *J* = 8.5 Hz, 2H), 7.00 (d, *J* = 8.4 Hz, 2H), 4.91 (d, *J* = 8.5 Hz, 1H), 4.12 – 4.01 (m, 1H), 3.91 (dd, *J* = 9.3, 3.4 Hz, 1H), 3.61 (dd, *J* = 9.4, 7.8 Hz, 1H), 2.50 – 2.31 (m, 1H), 2.00 (m, 2H), 1.60 (dd, *J* = 12.5, 6.1 Hz, 1H), 1.44 (s, 9H), 1.13 – 1.03 (m, 21H).

Minor rotamer $^{13}$C NMR (75 MHz, $CDCl_3$) δ 153.98, 143.67, 131.55, 126.98, 120.02, 79.93, 63.61, 61.32, 59.74, 31.97, 28.64, 25.18, 18.14, 12.10.

***tert*-Butyl (2*S*,5*R*)-2-(4-bromophenyl)-5-(((triisopropylsilyl)oxy)methyl)pyrrolidine-1-carboxylate (124c)**

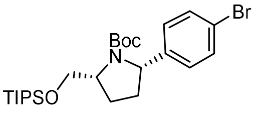

Following general procedure H, **123c** (2.15 g crude, 50% purity, 2.61 mmol product) was reacted with $Boc_2O$ (1.42 g, 6.52 mmol) and triethylamine (0.727 mL, 5.22 mmol) for 1.5 h to afford the title compound as a colourless oil (0.904 g, 1.76 mmol, 67%).

$^{1}H$ NMR (500 MHz, 333 K, $CDCl_3$) δ 7.38 (d, *J* = 8.4 Hz, 2H), 7.16 (d, *J* = 8.4 Hz, 2H), 4.69 (bs, 1H), 4.06 – 3.99 (m, 1H), 3.97 (dd, *J* = 9.4, 3.7 Hz, 1H), 3.86 (s, 1H), 2.28 – 2.14 (m, 1H), 2.14 – 2.03 (m, 1H), 2.03 – 1.91 (m, 1H), 1.91 – 1.82 (m, 1H), 1.27 (s, 9H), 1.13 – 1.03 (m, 21H).

$^{13}C$ NMR (126 MHz, 333 K, $CDCl_3$) δ 155.24, 144.32, 131.37, 127.77, 120.26, 79.80, 64.85, 62.85, 60.91, 34.55, 28.49, 27.20, 18.23, 12.36.

***tert*-Butyl (2*R*,5*R*)-2-(4-bromophenyl)-5-(((triisopropylsilyl)oxy)methyl)pyrrolidine-1-carboxylate (124d)**

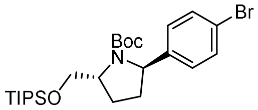


Following general procedure H, **123d** (0.430 g crude, 60% purity, 0.625 mmol product) was reacted with $Boc_2O$ (0.341 g, 1.56 mmol) and triethylamine (0.174 mL, 1.25 mmol) for 4.5 h to afford the title compound as a colourless oil (0.133 g, 0.259 mmol, 44%).

Rotamer equilibrium (3:2).

Major rotamer $^{1}H$ NMR (300 MHz, $CDCl_3$) δ 7.41 (d, *J* = 8.5 Hz, 2H), 7.00 (d, *J* = 8.4 Hz, 2H), 4.79 (d, *J* = 8.5 Hz, 1H), 4.18 (ddt, *J* = 7.7, 5.2, 2.6 Hz, 1H), 3.98 (dd, *J* = 9.8, 5.2 Hz, 1H), 3.81 (dd, *J* = 9.8, 2.8 Hz, 1H), 2.66 – 2.48 (m, 1H), 2.13 – 1.87 (m, 2H), 1.60 (dd, *J* = 11.0, 6.2 Hz, 1H), 1.15 (s, 9H), 1.11 – 1.01 (m, 21H).

Major rotamer $^{13}C$ NMR (75 MHz, $CDCl_3$) δ 153.94, 145.22, 131.25, 127.09, 120.00, 79.28, 63.59, 61.92, 59.74, 33.44, 28.21, 25.16, 18.12, 12.08.

Minor rotamer $^{1}H$ NMR (300 MHz, $CDCl_3$) δ 7.41 (d, *J* = 8.5 Hz, 2H), 7.00 (d, *J* = 8.4 Hz, 2H), 4.91 (d, *J* = 8.4 Hz, 1H), 4.12 – 4.02 (m, 1H), 3.91 (dd, *J* = 9.3, 3.4 Hz, 1H), 3.61 (dd, *J* = 9.4, 7.8 Hz, 1H), 2.48 – 2.35 (m, 1H), 2.13 – 1.89 (m, 2H), 1.60 (dd, *J* = 11.4, 5.8 Hz, 1H), 1.44 (s, 9H), 1.12 – 1.04 (m, 21H).

Minor rotamer $^{13}C$ NMR (75 MHz, $CDCl_3$) δ 153.80, 143.64, 131.52, 126.95, 120.27, 79.88, 63.59, 61.29, 59.71, 31.94, 28.61, 25.16, 18.12, 12.08.

***tert*-Butyl (2*R*,5*S*)-2-(4-bromophenyl)-5-(hydroxymethyl)pyrrolidine-1-carboxylate (125a)**

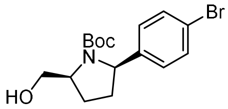


Following general procedure I, **124a** (0.494 g, 0.963 mmol) was reacted with TBAF (1 M in THF, 4.82 mL, 4.82 mmol) for 2.5 h to afford the title compound as a colourless oil (0.313 g, 0.877 mmol, 91%).

$^{1}H$ NMR (400 MHz, $CDCl_3$) δ 7.44 (d, *J* = 8.4 Hz, 2H), 7.13 (d, *J* = 8.5 Hz, 2H), 4.79 (t, *J* = 7.0 Hz, 1H), 4.16 (p, *J* = 6.3 Hz, 1H), 3.78 (d, *J* = 6.9 Hz, 2H), 2.34 – 2.15 (m, 1H), 2.11 – 1.91 (m, 1H), 1.90 – 1.73 (m, 1H), 1.63 (bs, 1H), 1.21 (s, 9H).

$^{13}C$ NMR (101 MHz, $CDCl_3$) δ 143.41, 131.49, 127.42, 120.43, 80.97, 67.55, 62.96, 61.68, 34.36, 28.17, 27.19.

***tert*-Butyl (2*S*,5*S*)-2-(4-bromophenyl)-5-(hydroxymethyl)pyrrolidine-1-carboxylate (125b)**

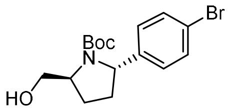


Following general procedure I, **124b** (48 mg, 94 µmol) was reacted with TBAF (1 M in THF, 0.468 mL, 0.468 mmol) for 1.25 h to afford the title compound as a colourless oil (19 mg, 53 µmol, 57%).

$^{1}H$ NMR (300 MHz, $CDCl_3$) δ 7.43 (d, *J* = 8.4 Hz, 2H), 6.99 (d, *J* = 8.1 Hz, 2H), 4.83 (dd, *J* = 8.1, 2.4 Hz, 1H), 4.31 (tt, *J* = 7.3, 3.4 Hz, 1H), 3.85 – 3.64 (m, 2H), 2.45 – 2.26 (m, 1H), 2.17 – 1.98 (m, 1H), 1.75 – 1.57 (m, 2H), 1.16 (s, 9H).

$^{13}C$ NMR (75 MHz, $CDCl_3$) δ 156.33, 144.10, 131.45, 127.04, 120.35, 80.68, 67.35, 62.46, 61.27, 33.51, 28.16, 26.18.

***tert*-Butyl (2*S*,5*R*)-2-(4-bromophenyl)-5-(hydroxymethyl)pyrrolidine-1-carboxylate (125c)**

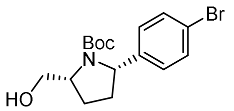

Following general procedure I, **124c** (0.910 g, 1.78 mmol) was reacted with TBAF (1 M in THF, 8.87 mL, 8.87 mmol) for 2.5 h to afford the title compound as a colourless oil (0.527 g, 1.478 mmol, 83%).

$^{1}$H NMR (400 MHz, $CDCl_3$) δ 7.44 (d, *J* = 8.4 Hz, 2H), 7.13 (d, *J* = 8.2 Hz, 2H), 4.80 (t, *J* = 7.0 Hz, 2H), 4.22 – 4.12 (m, 1H), 3.78 (t, *J* = 5.4 Hz, 2H), 2.33 – 2.19 (m, 1H), 2.08 – 1.96 (m, 2H), 1.88 – 1.75 (m, 1H), 1.62 (bs, 1H), 1.21 (s, 9H).

$^{13}$C NMR (101 MHz, $CDCl_3$) δ 131.53, 127.45, 120.48, 81.05, 67.73, 63.01, 61.75, 34.38, 28.21, 27.20.

***tert*-Butyl (2*R*,5*R*)-2-(4-bromophenyl)-5-(hydroxymethyl)pyrrolidine-1-carboxylate (125d)**

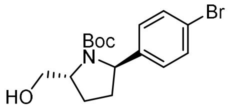

Following general procedure I, **124d** (0.133 g, 0.259 mmol) was reacted with TBAF (1 M in THF, 1.30 mL, 1.30 mmol) for 1.25 h to afford the title compound as a colourless oil (98 mg, quant.).

$^{1}$H NMR (300 MHz, $CDCl_3$) δ 7.43 (d, *J* = 8.4 Hz, 2H), 6.99 (d, *J* = 8.4 Hz, 2H), 4.83 (dd, *J* = 8.2, 2.4 Hz, 1H), 4.30 (tt, *J* = 7.2, 3.2 Hz, 1H), 3.87 – 3.55 (m, 2H), 2.51 – 2.23 (m, 2H), 2.19 – 1.98 (m, 1H), 1.77 – 1.57 (m, 2H), 1.16 (s, 9H).

$^{13}$C NMR (75 MHz, $CDCl_3$) δ 156.26, 144.09, 131.43, 127.02, 120.32, 80.63, 67.16, 62.42, 61.22, 33.46, 28.14, 26.11.

***tert*-Butyl (2*R*,5*S*)-2-(4-bromophenyl)-5-(((methylsulfonyl)oxy)methyl)pyrrolidine-1-carboxylate (126a)**

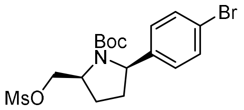

Following general procedure J, **125a** (0.313 g, 0.877 mmol) was reacted with MsCl (0.102 mL, 1.32 mmol) and triethylamine (0.367 mL, 2.63 mmol) to afford the crude title compound (0.393 g, quant.).

$^{1}$H NMR (300 MHz, $CDCl_3$) δ 7.44 (d, *J* = 8.4 Hz, 2H), 7.11 (d, *J* = 8.4 Hz, 2H), 4.68 (bs, 1H), 4.49 (dd, *J* = 9.5, 3.5 Hz, 1H), 4.39 (bs, 1H), 4.23 (bs, 1H), 3.04 (s, 3H), 2.39 – 2.18 (m, 1H), 2.17 – 1.95 (m, 2H), 1.94 – 1.78 (m, 1H), 1.40 – 1.06 (m, 9H).

***tert*-Butyl (2*S*,5*S*)-2-(4-bromophenyl)-5-(((methylsulfonyl)oxy)methyl)pyrrolidine-1-carboxylate (126b)**

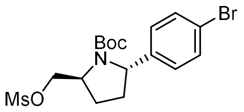

Following general procedure J, **125b** (19 mg, 53 µmol) was reacted with MsCl (6.2 µL, 80 µmol) and triethylamine (22 µL, 0.16 mmol) to afford the crude title compound (24 mg, quant.).

Rotamer equilibrium (3:1).

Major rotamer $^{1}$H NMR (400 MHz, $CDCl_3$) δ 7.43 (d, *J* = 8.4 Hz, 2H), 6.97 (d, *J* = 8.4 Hz, 2H), 4.85 (d, *J* = 8.3 Hz, 1H), 4.49 – 4.40 (m, 1H), 4.40 – 4.32 (m, 2H), 3.05 (s, 3H), 2.55 – 2.34 (m, 1H), 2.21 – 2.05 (m, 1H), 1.96 (dd, *J* = 13.3, 7.2 Hz, 1H), 1.72 (dd, *J* = 12.6, 7.1 Hz, 1H), 1.17 (s, 9H).

Major rotamer $^{13}$C NMR (101 MHz, $CDCl_3$) δ 143.89, 131.48, 126.92, 120.46, 80.40, 69.28, 61.62, 56.86, 37.19, 32.98, 28.15, 25.15;

Minor rotamer $^{1}$H NMR (400 MHz, $CDCl_3$) δ 7.43 (d, *J* = 8.4 Hz, 2H), 6.97 (d, *J* = 8.4 Hz, 2H), 4.97 (d, *J* = 8.3 Hz, 1H), 4.49 – 4.41 (m, 1H), 4.30 – 4.24 (m, 1H), 4.19 (dd, *J* = 9.6, 7.3 Hz, 1H), 3.05 (s, 3H), 2.59 – 2.29 (m, 1H), 2.23 – 2.04 (m, 1H), 1.96 (dd, *J* = 13.3, 7.2 Hz, 1H), 1.72 (dd, *J* = 12.6, 7.1 Hz, 1H), 1.47 (s, 9H).

Minor rotamer $^{13}$C NMR (101 MHz, $CDCl_3$) δ 143.89, 131.71, 126.80, 120.46, 80.40, 68.74, 61.13, 56.78, 37.70, 31.92, 28.53, 25.72.

**_tert_-Butyl (2_S_,5_R_)-2-(4-bromophenyl)-5-(((methylsulfonyl)oxy)methyl)pyrrolidine-1-carboxylate (126c)**

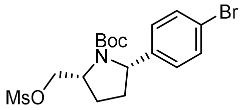


Following general procedure J, **125c** (0.526 g, 1.48 mmol) was reacted with MsCl (0.171 mL, 2.22 mmol) and triethylamine (0.618 mL, 4.43 mmol) to afford the crude title compound (0.691 g, quant.).

$^{1}$H NMR (300 MHz, $CDCl_3$) δ 7.43 (d, _J_ = 8.5 Hz, 2H), 7.12 (d, _J_ = 8.5 Hz, 2H), 4.68 (bs, 1H), 4.49 (dd, _J_ = 9.5, 3.5 Hz, 1H), 4.39 (bs, 1H), 4.28 – 4.15 (m, 1H), 3.04 (s, 3H), 2.38 – 2.20 (m, 1H), 2.19 – 1.95 (m, 2H), 1.95 – 1.80 (m, 1H), 1.19 (s, 9H).

$^{13}$C NMR (75 MHz, $CDCl_3$) δ 154.85, 146.74, 131.45, 127.37, 120.45, 80.58, 69.62, 62.46, 57.62, 45.95, 37.39, 28.17, 27.17.

**_tert_-Butyl (2_R_,5_R_)-2-(4-bromophenyl)-5-(((methylsulfonyl)oxy)methyl)pyrrolidine-1-carboxylate (126d)**

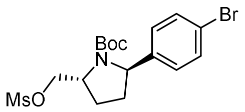


Following general procedure J, **125d** (98 mg, 0.28 mmol) was reacted with MsCl (32 µL, 0.41 mmol) and triethylamine (0.115 mL, 0.824 mmol) to afford the crude title compound (0.121 g, quant.).

Rotamer equilibrium (3:1).

Major rotamer $^{1}$H NMR (300 MHz, $CDCl_3$) δ 7.43 (d, _J_ = 8.4 Hz, 2H), 6.98 (d, _J_ = 8.3 Hz, 2H), 4.85 (d, _J_ = 8.3 Hz, 1H), 4.54 – 4.31 (m, 3H), 3.05 (s, 3H), 2.56 – 2.31 (m, 1H), 2.25 – 2.04 (m, 1H), 1.96 (dd, _J_ = 13.3, 7.3 Hz, 1H), 1.72 (dd, _J_ = 12.5, 7.0 Hz, 1H), 1.17 (s, 9H).

Major rotamer $^{13}$C NMR (75 MHz, $CDCl_3$) δ 153.94, 143.84, 131.38, 126.86, 120.36, 80.27, 69.18, 61.53, 56.78, 37.10, 32.89, 28.06, 25.06.

Minor rotamer $^{1}$H NMR (300 MHz, $CDCl_3$) δ 7.43 (d, _J_ = 8.4 Hz, 2H), 6.98 (d, _J_ = 8.3 Hz, 2H), 4.96 (d, _J_ = 8.3 Hz, 1H), 4.64 – 4.30 (m, 1H), 4.27 (dd, _J_ = 7.6, 2.6 Hz, 1H), 4.20 (dd, _J_ = 9.4, 7.3 Hz, 1H), 3.06 (s, 3H), 2.61 – 2.31 (m, 1H), 2.25 – 2.04 (m, 1H), 1.96 (dd, _J_ = 13.3, 7.3 Hz, 1H), 1.72 (dd, _J_ = 12.5, 7.0 Hz, 1H), 1.47 (s, 9H).

Minor rotamer $^{13}$C NMR (75 MHz, $CDCl_3$) δ 153.94, 143.84, 131.61, 126.75, 120.36, 80.90, 68.73, 61.04, 56.70, 37.60, 31.85, 28.43, 25.64.

**_tert_-Butyl (2_S_,5_R_)-2-(azidomethyl)-5-(4-bromophenyl)pyrrolidine-1-carboxylate (127a)**

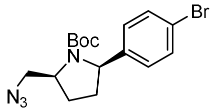


Following general procedure K, **126a** (0.391 g, 0.905 mmol) was reacted with $NaN_3$ (0.353 g, 5.43 mmol) for 65 h to afford the title compound as a colourless oil (0.239 g, 0.627 mmol, 69%).

$^{1}$H NMR (500 MHz, 333 K, $CDCl_3$) δ 7.42 (d, _J_ = 8.4 Hz, 2H), 7.12 (d, _J_ = 8.4 Hz, 2H), 4.73 (t, _J_ = 7.2 Hz, 1H), 4.08 (h, _J_ = 3.7 Hz, 1H), 3.70 (dd, _J_ = 12.0, 3.9 Hz, 1H), 3.49 (dd, _J_ = 12.0, 7.5 Hz, 1H), 2.31 – 2.20 (m, 1H), 2.10 – 1.97 (m, 1H), 1.93 – 1.80 (m, 2H), 1.29 (s, 9H).

$^{13}$C NMR (126 MHz, 333 K, $CDCl_3$) δ 154.10, 142.40, 130.60, 126.58, 119.60, 79.54, 61.74, 57.63, 53.52, 33.15, 27.42, 27.23.

**_tert_-Butyl (2_S_,5_S_)-2-(azidomethyl)-5-(4-bromophenyl)pyrrolidine-1-carboxylate (127b)**

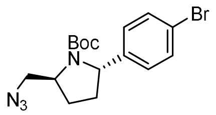


Following general procedure K, **126b** (24 mg, 55 µmol) was reacted with $NaN_3$ (21 mg, 55 µmol) for 17 h to afford the title compound as a colourless oil (11 mg, 29 µmol, 53%).

Rotamer equilibrium (7:3).

Major rotamer $^{1}H$ NMR (400 MHz, $CDCl_3$) δ 7.43 (d, *J* = 8.4 Hz, 2H), 6.98 (d, *J* = 8.4 Hz, 2H), 4.86 (d, *J* = 7.7 Hz, 1H), 4.30 – 4.19 (m, 1H), 3.66 (dd, *J* = 12.1, 6.5 Hz, 1H), 3.50 (dd, *J* = 12.1, 2.9 Hz, 1H), 2.57 – 2.35 (m, 1H), 2.15 – 2.00 (m, 1H), 1.89 – 1.77 (m, 1H), 1.76 – 1.63 (m, 1H), 1.17 (s, 9H).

Major rotamer $^{13}C$ NMR (101 MHz, $CDCl_3$) δ 153.02, 143.21, 130.46, 125.99, 119.39, 79.20, 60.78, 56.50, 51.49, 32.06, 27.20, 24.93.

Minor rotamer $^{1}H$ NMR (400 MHz, $CDCl_3$) δ 7.43 (d, *J* = 8.4 Hz, 2H), 6.98 (d, *J* = 8.4 Hz, 2H), 4.96 (d, *J* = 8.4 Hz, 1H), 4.14 (td, *J* = 8.1, 2.9 Hz, 1H), 3.57 (dd, *J* = 11.9, 2.7 Hz, 1H), 3.33 (dd, *J* = 11.9, 8.0 Hz, 1H), 2.64 – 2.29 (m, 1H), 2.15 – 1.96 (m, 1H), 1.93 – 1.79 (m, 1H), 1.78 – 1.64 (m, 1H), 1.47 (s, 9H).

Minor rotamer $^{13}C$ NMR (101 MHz, $CDCl_3$) δ 153.02, 141.83, 130.69, 125.87, 119.59, 79.71, 60.18, 56.45, 52.49, 30.94, 27.59, 25.55.

***tert*-Butyl (2*R*,5*S*)-2-(azidomethyl)-5-(4-bromophenyl)pyrrolidine-1-carboxylate (127c)**

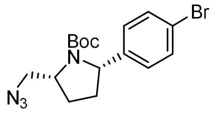


Following general procedure K, **126c** (0.691 g, 1.60 mmol) was reacted with $NaN_3$ (0.621 g, 9.55 mmol) for 65 h to afford the title compound as a colourless oil (0.370 g, 0.970 mmol, 61%).

$^{1}H$ NMR (126 MHz, 333 K, $CDCl_3$) δ 7.42 (d, *J* = 8.5 Hz, 2H), 7.12 (d, *J* = 8.4 Hz, 2H), 4.72 (t, *J* = 7.1 Hz, 1H), 4.08 (h, *J* = 3.7 Hz, 1H), 3.70 (dd, *J* = 11.9, 3.8 Hz, 1H), 3.49 (dd, *J* = 11.7, 7.4 Hz, 1H), 2.31 – 2.18 (m, 1H), 2.11 – 1.96 (m, 1H), 1.93 – 1.78 (m, 2H), 1.29 (s, 9H).

$^{13}C$ NMR (126 MHz, 333 K, $CDCl_3$) δ 154.09, 142.41, 130.60, 126.58, 119.59, 79.53, 61.74, 57.62, 53.52, 33.16, 27.42, 27.22.

***tert*-Butyl (2*R*,5*R*)-2-(azidomethyl)-5-(4-bromophenyl)pyrrolidine-1-carboxylate (127d)**

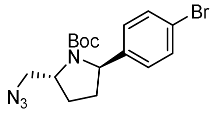


Following general procedure K, **126d** (0.121 g, 0.278 mmol) was reacted with $NaN_3$ (0.109 g, 1.67 mmol) for 17 h to afford the title compound as a colourless oil (56 mg, 0.147 mmol, 53%).

Rotamer equilibrium (7:3).

Major rotamer $^{1}H$ NMR (300 MHz, $CDCl_3$) δ 7.43 (d, *J* = 8.5 Hz, 2H), 6.98 (d, *J* = 8.4 Hz, 2H), 4.86 (d, *J* = 8.2 Hz, 1H), 4.33 – 4.21 (m, 1H), 3.66 (dd, *J* = 12.1, 6.5 Hz, 1H), 3.50 (dd, *J* = 12.1, 2.9 Hz, 1H), 2.56 – 2.32 (m, 1H), 2.17 – 2.00 (m, 1H), 1.89 – 1.76 (m, 1H), 1.76 – 1.62 (m, 1H), 1.17 (s, 9H).

Major rotamer $^{13}C$ NMR (75 MHz, $CDCl_3$) δ 152.97, 143.19, 130.42, 125.96, 119.35, 79.14, 60.75, 56.47, 51.45, 32.02, 27.16, 24.90.

Minor rotamer $^{1}H$ NMR (300 MHz, $CDCl_3$) δ 7.43 (d, *J* = 8.5 Hz, 2H), 6.98 (d, *J* = 8.4 Hz, 2H), 4.96 (d, *J* = 8.3 Hz, 1H), 4.20 – 4.04 (m, 1H), 3.57 (dd, *J* = 12.0, 2.9 Hz, 1H), 3.33 (dd, *J* = 11.9, 7.9 Hz, 1H), 2.60 – 2.31 (m, 1H), 2.20 – 2.00 (m, 1H), 1.90 – 1.76 (m, 1H), 1.76 – 1.60 (m, 1H), 1.47 (s, 9H).

Minor rotamer $^{13}C$ NMR (75 MHz, $CDCl_3$) δ 152.97, 141.80, 130.64, 125.84, 119.55, 79.65, 60.14, 56.42, 52.46, 30.91, 27.55, 25.52.

***tert*-Butyl (2*S*,5*R*)-2-(aminomethyl)-5-(4-bromophenyl)pyrrolidine-1-carboxylate (128a)**

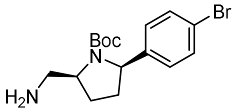


Following general procedure L, **127a** (0.221 g, 0.597 mmol) was reacted with $PPh_3$ (0.304 g, 1.16 mmol) and water (21 µL, 1.16 mmol) to afford the tile compound as a colourless oil that crystalized upon storage (0.130 g, 0.366 mmol, 63%).

$^{1}$H NMR (500 MHz, 333K, $CDCl_3$) δ 7.41 (d, *J* = 8.4 Hz, 2H), 7.11 (d, *J* = 8.4 Hz, 2H), 4.73 (t, *J* = 7.3 Hz, 1H), 3.98 – 3.93 (m, 1H), 3.09 (dd, *J* = 12.6, 5.6 Hz, 1H), 2.77 (dd, *J* = 12.6, 7.3 Hz, 1H), 2.30 – 2.19 (m, 1H), 2.08 – 1.96 (m, 1H), 1.88 – 1.73 (m, 2H), 1.49 (s, 2H), 1.28 (s, 9H).

$^{13}$C NMR (126 MHz, 333K, $CDCl_3$) δ 154.64, 142.98, 130.56, 126.58, 119.41, 79.03, 61.73, 61.07, 45.74, 33.35, 27.44, 27.29.

***tert*-Butyl (2*S*,5*S*)-2-(aminomethyl)-5-(4-bromophenyl)pyrrolidine-1-carboxylate (128b)**

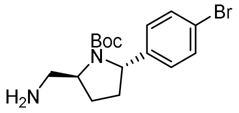


Following general procedure L, **127b** (11 mg, 29 µmol) was reacted with $PPh_3$ (17 mg, 65 µmol) and water (2 µL, 0.1 mmol) to afford the tile compound as a colourless oil that crystalized upon storage (8.7 mg, 24 µmol, 83%).

Rotamer equilibrium (8:2).

Major rotamer $^{1}$H NMR (400 MHz, $CDCl_3$) δ 7.43 (d, *J* = 8.4 Hz, 2H), 6.98 (d, *J* = 8.5 Hz, 2H), 4.82 (d, *J* = 6.3 Hz, 1H), 4.34 – 4.13 (m, 1H), 3.95 (bs, 2H), 3.14 (dd, *J* = 12.9, 5.9 Hz, 1H), 2.94 (dd, *J* = 12.9, 5.6 Hz, 1H), 2.42 – 2.27 (m, 1H), 2.20 – 2.03 (m, 1H), 1.87 – 1.74 (m, 1H), 1.74 – 1.62 (m, 1H), 1.15 (s, 9H).

Major rotamer $^{13}$C NMR (101 MHz, $CDCl_3$) δ 154.33, 142.97, 130.46, 126.04, 119.41, 79.59, 76.48, 61.06, 58.44, 44.14, 31.99, 27.16, 25.47.

Minor rotamer $^{1}$H NMR (400 MHz, $CDCl_3$) δ 7.43 (d, *J* = 8.4 Hz, 2H), 6.98 (d, *J* = 8.5 Hz, 2H), 4.94 (d, *J* = 8.4 Hz, 1H), 3.95 (bs, 3H), 3.05 (dd, *J* = 12.5, 3.3 Hz, 1H), 2.69 (dd, *J* = 12.6, 8.3 Hz, 1H), 2.42 – 2.27 (m, 1H), 2.20 – 2.03 (m, 1H), 1.87 – 1.74 (m, 1H), 1.74 – 1.62 (m, 1H), 1.45 (s, 9H).

Minor rotamer $^{13}$C NMR (101 MHz, $CDCl_3$) δ 154.33, 142.16, 130.61, 125.95, 119.41, 79.16, 76.48, 60.07, 59.71, 43.79, 31.01, 27.61, 24.87.

***tert*-Butyl (2*R*,5*S*)-2-(aminomethyl)-5-(4-bromophenyl)pyrrolidine-1-carboxylate (128c)**

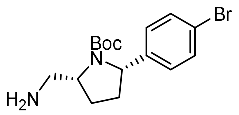


Following general procedure L, **127c** (0.359 g, 0.942 mmol) was reacted with $PPh_3$ (0.494 g, 1.83 mmol) and water (34 µL, 1.9 mmol) to afford the title compound as a colourless oil that crystalized upon storage (0.236 g, 0.066 mmol, 70%).

$^{1}$H NMR (500 MHz, $CDCl_3$) δ 7.41 (d, *J* = 8.4 Hz, 2H), 7.11 (d, *J* = 8.4 Hz, 2H), 4.73 (t, *J* = 7.3 Hz, 1H), 4.00 – 3.88 (m, 1H), 3.09 (dd, *J* = 12.6, 5.7 Hz, 1H), 2.78 (dd, *J* = 12.6, 7.3 Hz, 1H), 2.30 – 2.20 (m, 1H), 2.06 – 1.96 (m, 1H), 1.87 – 1.75 (m, 2H), 1.28 (s, 9H).

$^{13}$C NMR (126 MHz, $CDCl_3$) δ 154.65, 142.97, 130.55, 126.57, 119.41, 79.04, 61.74, 61.04, 45.73, 33.34, 27.44, 27.29.

***tert*-Butyl (2*R*,5*R*)-2-(aminomethyl)-5-(4-bromophenyl)pyrrolidine-1-carboxylate (128d)**

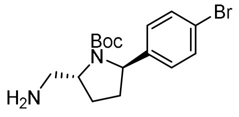


Following general procedure L, **127d** (56 mg, 0.147 mmol) was reacted with $PPh_3$ (77 mg, 0.30 mmol) and water (5.3 µL, 0.23 mmol) to afford the title compound as a colourless oil that crystalized upon storage (34 mg, 95 µmol, 65%).

Rotamer equilibrium (8:2).

Major rotamer $^{1}$H NMR (400 MHz, $CDCl_3$) δ 7.43 (d, *J* = 8.4 Hz, 2H), 6.98 (d, *J* = 8.5 Hz, 2H), 4.82 (d, *J* = 6.3 Hz, 1H), 4.25 (bs, 3H), 3.14 (dd, *J* = 12.8, 5.5 Hz, 1H), 2.91 (dd, *J* = 12.8, 6.1 Hz, 1H), 2.45 – 2.23 (m, 1H), 2.19 – 2.03 (m, 1H), 1.92 – 1.75 (m, 1H), 1.75 – 1.59 (m, 1H), 1.15 (s, 9H).

Major rotamer $^{13}$C NMR (101 MHz, $CDCl_3$) δ 155.11, 144.02, 131.41, 127.01, 120.35, 80.41, 61.95, 59.46, 44.82, 32.91, 28.14, 26.21.

Minor rotamer $^{1}$H NMR (400 MHz, $CDCl_3$) δ 7.43 (d, *J* = 8.4 Hz, 2H), 6.98 (d, *J* = 8.5 Hz, 2H), 4.94 (d, *J* = 8.4 Hz, 1H), 4.25 (bs, 3H), 3.98 (td, *J* = 8.2, 3.3 Hz, 1H), 3.06 (dd, *J* = 12.7, 3.4 Hz, 1H), 2.70 (dd, *J* = 12.6, 8.5 Hz, 1H), 2.45 – 2.23 (m, 1H), 2.19 – 2.03 (m, 1H), 1.92 – 1.75 (m, 1H), 1.75 – 1.59 (m, 1H), 1.45 (s, 9H).

Minor rotamer $^{13}$C NMR (101 MHz, $CDCl_3$) δ 153.86, 143.13, 131.58, 126.92, 120.40, 80.14, 61.03, 60.58, 44.65, 31.98, 28.58, 25.82.

***tert*-Butyl (2*R*,5*S*)-2-(4-bromophenyl)-5-((isoquinoline-5-sulfonamido)methyl)pyrrolidine-1-carboxylate (129a)**

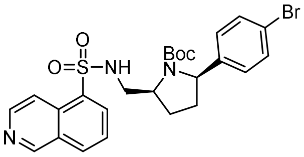


Following general procedure B, **128a** (0.113 g, 0.318 mmol) was reacted with quinoline-5-sulfonylchloride·HCl (87 mg, 0.38 mmol) and triethylamine (0.100 mL, 0.716 mmol) to afford the title compound as an off-white solid (0.166 g, 0.304 mmol, 95%).

$^{1}$H NMR (400 MHz, $CDCl_3$) δ 9.40 (s, 1H), 8.66 (d, *J* = 6.2 Hz, 1H), 8.49 (d, *J* = 6.2 Hz, 1H), 8.45 (dd, *J* = 7.4, 1.2 Hz, 1H), 8.25 (d, *J* = 8.2 Hz, 1H), 7.72 (t, *J* = 7.3 Hz, 1H), 7.56 (bs, 1H), 7.20 (d, *J* = 8.0 Hz, 2H), 6.75 (d, *J* = 7.9 Hz, 2H), 4.62 (t, *J* = 7.5 Hz, 1H), 4.10 (t, *J* = 8.8 Hz, 1H), 3.29 – 2.95 (m, 2H), 2.23 – 2.12 (m, 1H), 2.07 – 1.90 (m, 1H), 1.73 – 1.56 (m, 2H), 1.15 (s, 9H).

$^{13}$C NMR (101 MHz, $CDCl_3$) δ 156.78, 153.18, 145.19, 142.60, 134.41, 133.33, 133.14, 131.30, 131.22, 129.05, 126.92, 125.92, 120.29, 117.47, 81.15, 62.97, 58.07, 48.92, 34.35, 29.66, 27.95.

***tert*-Butyl (2*S*,5*S*)-2-(4-bromophenyl)-5-((isoquinoline-5-sulfonamido)methyl)pyrrolidine-1-carboxylate (129b)**

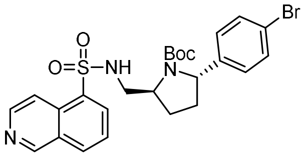


Following general procedure B, **128b** (8.7 mg, 24 μmol) was reacted with quinoline-5-sulfonylchloride·HCl (8 mg, 0.04 mmol) and triethylamine (10 μL, 73 μmol) to afford the title compound as an off-white solid (11 mg, 20 μmol, 84%).

$^{1}$H NMR (400 MHz, $CDCl_3$) δ 9.37 (s, 1H), 8.70 (d, *J* = 6.2 Hz, 1H), 8.45 (d, *J* = 6.1 Hz, 1H), 8.43 (dd, *J* = 7.4, 1.2 Hz, 1H), 8.21 (d, *J* = 8.2 Hz, 1H), 7.79 – 7.64 (m, 1H), 7.40 (d, *J* = 8.5 Hz, 2H), 6.88 (d, *J* = 8.4 Hz, 2H), 4.72 (d, *J* = 6.5 Hz, 1H), 4.25 – 4.17 (m, 1H), 3.16 (dd, *J* = 12.4, 3.9 Hz, 1H), 3.06 (dd, *J* = 12.4, 7.5 Hz, 1H), 2.37 – 2.17 (m, 1H), 2.12 – 2.00 (m, 1H), 1.67 – 1.58 (m, 2H), 1.11 (s, 9H).

$^{13}$C NMR (101 MHz, $CDCl_3$) δ 154.82, 152.35, 144.41, 142.58, 133.80, 132.49, 132.07, 130.49, 130.44, 128.22, 125.85, 124.96, 119.49, 116.54, 79.97, 61.23, 57.05, 47.66, 32.03, 27.08, 26.01.

***tert*-Butyl (2*S*,5*R*)-2-(4-bromophenyl)-5-((isoquinoline-5-sulfonamido)methyl)pyrrolidine-1-carboxylate (129c)**

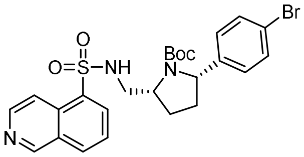


Following general procedure B, **128c** (0.144 g, 0.630 mmol) was reacted with quinoline-5-sulfonylchloride·HCl (0.172 g, 0.757 mmol) and triethylamine (0.207 mL, 1.48 mmol) to afford the title compound as an off-white solid (0.324 g, 0.593 mmol, 94%).

$^{1}$H NMR (400 MHz, $CDCl_3$) δ 9.40 (s, 1H), 8.65 (d, *J* = 6.1 Hz, 1H), 8.49 (d, *J* = 6.2 Hz, 1H), 8.45 (d, *J* = 7.4 Hz, 1H), 8.25 (d, *J* = 8.2 Hz, 1H), 7.72 (t, *J* = 7.8 Hz, 1H), 7.61 (bs, 1H), 7.20 (d, *J* = 8.0 Hz, 2H), 6.75 (d, *J* = 8.0 Hz, 2H), 4.62 (t, *J* = 7.2 Hz, 1H), 4.19 – 3.98 (m, 1H), 3.38 – 2.91 (m, 2H), 2.29 – 2.10 (m, 1H), 2.10 – 1.89 (m, 1H), 1.76 – 1.53 (m, 2H), 1.14 (s, 9H).

$^{13}$C NMR (101 MHz, $CDCl_3$) δ 156.69, 153.10, 145.03, 142.56, 134.30, 133.26, 133.05, 131.17, 131.09, 128.94, 126.85, 125.88, 120.15, 117.37, 80.96, 62.87, 57.99, 48.85, 34.25, 28.40, 27.85.

***tert*-Butyl (2*R*,5*R*)-2-(4-bromophenyl)-5-((isoquinoline-5-sulfonamido)methyl)pyrrolidine-1-carboxylate (129d)**

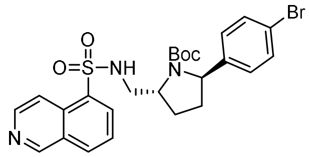

Following general procedure B, **128d** (38 mg, 0.11 mmol) was reacted with quinoline-5-sulfonylchloride·HCl (37 mg, 0.16 mmol) and triethylamine (34 µL, 0.24 mmol) to afford the title compound as an off-white solid (57 mg, 0.10 mmol, 98%).

$^1$H NMR (400 MHz, $CDCl_3$) δ 9.36 (s, 1H), 8.67 (d, *J* = 6.1 Hz, 1H), 8.45 (d, *J* = 5.8 Hz, 1H), 8.43 (dd, *J* = 7.4, 1.2 Hz, 1H), 8.20 (d, *J* = 8.2 Hz, 1H), 7.70 (t, *J* = 7.6 Hz, 1H), 7.40 (d, *J* = 8.4 Hz, 2H), 6.89 (d, *J* = 8.4 Hz, 2H), 4.72 (d, *J* = 6.4 Hz, 1H), 4.27 – 4.16 (m, 1H), 3.16 (dd, *J* = 12.4, 4.4 Hz, 1H), 3.08 (dd, *J* = 12.4, 7.1 Hz, 1H), 2.36 – 2.19 (m, 1H), 2.09 – 2.00 (m, 1H), 1.78 – 1.52 (m, 2H), 1.10 (s, 9H).

$^{13}$C NMR (101 MHz, $CDCl_3$) δ 154.65, 152.30, 144.27, 142.60, 133.79, 132.46, 132.03, 130.44, 130.38, 128.17, 125.83, 124.96, 119.43, 116.53, 79.84, 61.13, 57.00, 47.24, 31.92, 27.04, 25.75.

***tert*-Butyl (2*R*,5*S*)-2-(4-(6-fluoropyridin-3-yl)phenyl)-5-((isoquinoline-5-sulfonamido)methyl)pyrrolidine-1-carboxylate (130a)**

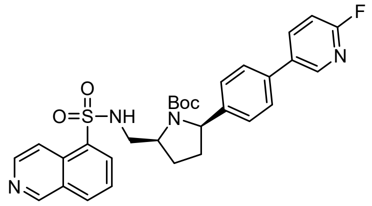

Following general procedure D, **129a** (84 mg, 0.15 mmol) was reacted with (6-fluoropyridin-3-yl)boronic acid (26 mg, 0.18 mmol), $K_2CO_3$ (85 mg, 0.62 mmol) and $Pd(PPh_3)_4$ (2.7 mg, 2.3 µmol) to afford the title compound as an off-white solid (78 mg, 0.14 mmol, 90%).

$^1$H NMR (300 MHz, $CDCl_3$) δ 9.41 (s, 1H), 8.68 (d, *J* = 6.1 Hz, 1H), 8.52 (s, 1H), 8.48 (dd, *J* = 7.3, 1.3 Hz, 1H), 8.38 (d, *J* = 2.8 Hz, 1H), 8.25 (d, *J* = 8.2 Hz, 1H), 7.94 (dd, *J* = 7.9, 2.7 Hz, 1H), 7.72 (t, *J* = 7.4 Hz, 1H), 7.24 (d, *J* = 7.9 Hz, 2H), 7.03 (dd, *J* = 8.4, 2.9 Hz, 1H), 6.98 (d, *J* = 9.4 Hz, 2H), 4.73 (t, *J* = 6.2 Hz, 1H), 4.20 – 4.11 (m, 1H), 3.28 – 3.05 (m, 2H), 2.30 – 2.18 (m, 1H), 2.09 – 1.96 (m, 3H), 1.79 – 1.71 (m, 1H), 1.70 – 1.61 (m, 1H), 1.17 (s, 9H).

$^{13}$C NMR (75 MHz, $CDCl_3$) δ 162.19 (d, *J* = 239 Hz), 152.26, 144.72 (d, *J* = 15 Hz), 144.45, 142.76, 138.64 (d, *J* = 7.9 Hz), 134.28, 133.66, 133.42 (d, *J* = 4.5 Hz), 132.36, 130.45, 128.25, 125.96, 125.13, 125.02, 116.68, 108.61 (d, *J* = 37.5 Hz), 80.33, 62.25, 57.16, 48.29, 33.54, 27.85, 27.12.

***tert*-Butyl (2*S*,5*S*)-2-(4-(6-fluoropyridin-3-yl)phenyl)-5-((isoquinoline-5-sulfonamido)methyl)pyrrolidine-1-carboxylate (130b)**

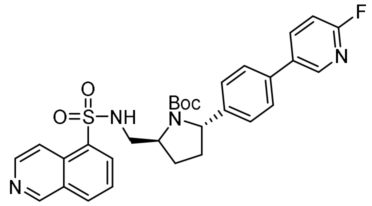

Following general procedure D, **129b** (11 mg, 20 µmol) was reacted with (6-fluoropyridin-3-yl)boronic acid (3.5 mg, 25 µmol), $K_2CO_3$ (11 mg, 82 µmol) and $Pd(PPh_3)_4$ (0.4 mg, 0.3 µmol) to afford the title compound as an off-white solid (5.9 mg, 10 µmol, 51%).

$^1$H NMR (400 MHz, $CDCl_3$) δ 9.37 (s, 1H), 8.71 (d, *J* = 6.0 Hz, 1H), 8.47 (d, *J* = 6.2 Hz, 1H), 8.44 (dd, *J* = 7.3, 1.2 Hz, 1H), 8.40 (d, *J* = 2.6 Hz, 1H), 8.22 (d, *J* = 8.2 Hz, 1H), 7.95 (td, *J* = 8.1, 2.8 Hz, 1H), 7.75 – 7.67 (m, 1H), 7.46 (d, *J* = 8.1 Hz, 2H), 7.11 (d, *J* = 8.3 Hz, 2H), 7.00 (dd, *J* = 8.5, 2.9 Hz, 1H), 4.82 (d, *J* = 7.8 Hz, 1H), 4.31 – 4.22 (m, 1H), 3.26 – 3.05 (m, 2H), 2.39 – 2.25 (m, 1H), 2.18 – 2.06 (m, 1H), 1.79 – 1.66 (m, 2H), 1.11 (s, 9H).

$^{13}$C NMR (101 MHz, $CDCl_3$) δ 161.37 (d, *J* = 412 Hz), 155.8, 152.36, 144.82 (d, *J* = 15 Hz), 144.43, 143.67, 138.69 (d, *J* = 8.3 Hz), 134.33, 133.84, 133.43 (d, *J* = 4.6 Hz), 132.51, 132.10, 130.48, 128.25, 126.00, 125.00, 124.98, 116.58, 108.62 (d, *J* = 37 Hz), 79.88, 61.47, 57.12, 47.79, 32.15, 27.09, 26.16.

***tert*-Butyl (2*S*,5*R*)-2-(4-(6-fluoropyridin-3-yl)phenyl)-5-((isoquinoline-5-sulfonamido)methyl)pyrrolidine-1-carboxylate (130c)**

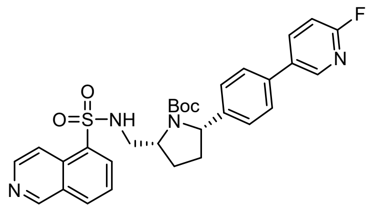

Following general procedure D, **129c** (0.10 g, 0..18 mmol) was reacted with (6-fluoropyridin-3-yl)boronic acid (31 mg, 0.22 mmol), $K_2CO_3$ (0.101 g, 0.732 mmol) and $Pd(PPh_3)_4$ (3.2 mg, 2.7 µmol) to afford the title compound as an off-white solid (97 mg, 0.17 mmol, 94%).

$^1$H NMR (300 MHz, CDCl$_3$) δ 9.41 (s, 1H), 8.67 (d, *J* = 6.2 Hz, 1H), 8.50 (s, 2H), 8.48 (s, 1H), 8.38 (s, 1H), 8.25 (d, *J* = 8.2 Hz, 1H), 7.95 (t, *J* = 6.4 Hz, 1H), 7.73 (t, *J* = 7.7 Hz, 1H), 7.25 (d, *J* = 7.8 Hz, 2H), 7.04 (s, 1H), 7.01 (s, 2H), 4.74 (s, 1H), 4.16 (s, 1H), 3.48 – 2.81 (m, 2H), 2.34 – 2.14 (m, 1H), 2.10 – 1.92 (m, 1H), 1.82 – 1.57 (m, 2H), 1.18 (s, 9H).

$^{13}$C NMR (75 MHz, CDCl$_3$) δ 163.09 (d, *J* = 239 Hz), 153.21, 145.61 (d, *J* = 15 Hz), 145.31, 143.72, 139.60 (d, *J* = 7.8 Hz), 135.16, 134.58, 134.37 (d, *J* = 5.1 Hz), 133.30, 131.36, 129.17, 126.87, 126.07, 126.00, 117.60, 109.54 (d, *J* = 38 Hz), 81.19, 63.17, 58.12, 49.15, 34.46, 28.73, 28.04.

***tert*-Butyl (2*R*,5*R*)-2-(4-(6-fluoropyridin-3-yl)phenyl)-5-((isoquinoline-5-sulfonamido)methyl)pyrrolidine-1-carboxylate (130d)**

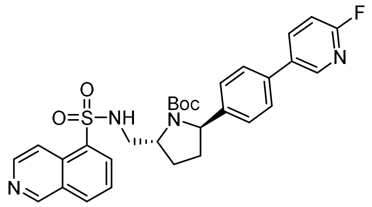

Following general procedure D, **129d** (57 mg, 0.10 mmol) was reacted with (6-fluoropyridin-3-yl)boronic acid (18 mg, 0.13 mmol), $K_2CO_3$ (58 mg, 0.42 mmol) and $Pd(PPh_3)_4$ (1.8 mg, 1.6 µmol) to afford the title compound as an off-white solid (36 mg, 64 µmol, 61%).

$^1$H NMR (300 MHz, CDCl$_3$) δ 9.37 (s, 1H), 8.70 (d, *J* = 6.1 Hz, 1H), 8.47 (d, *J* = 5.9 Hz, 1H), 8.44 (dd, *J* = 7.4, 1.3 Hz, 1H), 8.40 (d, *J* = 2.6 Hz, 1H), 8.21 (d, *J* = 8.3 Hz, 1H), 7.95 (td, *J* = 7.9, 2.6 Hz, 1H), 7.71 (t, *J* = 8.0 Hz, 1H), 7.46 (d, *J* = 8.2 Hz, 2H), 7.11 (d, *J* = 8.2 Hz, 2H), 7.00 (dd, *J* = 8.6, 3.1 Hz, 1H), 4.82 (d, *J* = 7.1 Hz, 1H), 4.35 – 4.14 (m, 1H), 3.33 – 3.05 (m, 2H), 2.43 – 2.21 (m, 1H), 2.21 – 2.01 (m, 1H), 1.76 – 1.62 (m, 2H), 1.11 (s, 9H).

$^{13}$C NMR (75 MHz, CDCl$_3$) δ 163.22 (d, *J* = 240 Hz), 155.87, 153.34, 145.80 (d, *J* = 15 Hz), 145.37, 144.70, 139.67 (d, *J* = 8.0 Hz), 135.31, 133.46, 133.05, 131.46, 129.23, 126.98, 125.99, 117.58, 109.59 (d, *J* = 37 Hz), 80.81, 62.43, 58.13, 48.56, 33.10, 28.08, 27.04.

***tert*-Butyl (2*R*,5*S*)-2-([1,1'-biphenyl]-4-yl)-5-((isoquinoline-5-sulfonamido)methyl)pyrrolidine-1-carboxylate (130e)**

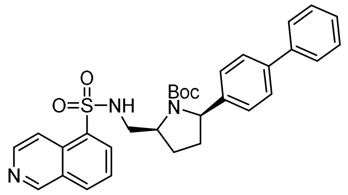

Following general procedure D, **129a** (15 mg, 27 µmol) was reacted with phenylboronic acid (4.0 mg, 33 µmol), $K_2CO_3$ (15 mg, 0.11 mmol) and $Pd(PPh_3)_4$ (0.5 mg, 0.4 µmol) to afford the title compound as an off-white solid (12 mg, 22 µmol, 80%).

$^1$H NMR (500 MHz, CDCl$_3$) δ 9.40 (d, *J* = 1.0 Hz, 1H), 8.69 (d, *J* = 6.1 Hz, 1H), 8.52 (d, *J* = 6.2 Hz, 1H), 8.47 (dd, *J* = 7.3, 1.2 Hz, 1H), 8.23 (dt, *J* = 8.3, 1.1 Hz, 1H), 7.71 (dd, *J* = 8.2, 7.3 Hz, 1H), 7.55 (dd, *J* = 8.3, 1.2 Hz, 2H), 7.46 (t, *J* = 7.7 Hz, 2H), 7.40 – 7.32 (m, 1H), 7.27 (d, *J* = 8.0 Hz, 2H), 6.88 (d, *J* = 7.7 Hz, 2H), 4.70 (s, 1H), 4.19 – 4.09 (m, 1H), 3.24 (s, 1H), 3.13 – 3.04 (m, 1H), 2.27 – 2.17 (m, 1H), 2.08 – 1.98 (m, 2H), 1.81 – 1.69 (m, 1H), 1.66 – 1.59 (m, 2H), 1.15 (s, 9H).

$^{13}$C NMR (126 MHz, CDCl$_3$) δ 152.31, 144.57, 141.60, 139.78, 138.85, 133.70, 132.37, 130.53, 128.30, 127.98, 126.44, 126.07, 125.00, 124.76, 116.76, 80.34, 62.44, 57.17, 48.60, 33.58, 28.03, 27.13.

***tert*-Butyl (2*R*,5*S*)-2-(4'-fluoro-[1,1'-biphenyl]-4-yl)-5-((isoquinoline-5-sulfonamido)methyl)pyrrolidine-1-carboxylate (130f)**

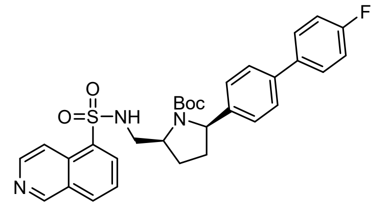

Following general procedure D, **129a** (15 mg, 27 µmol) was reacted with pyridin-3-ylboronic acid (4.6 mg, 33 µmol), $K_2CO_3$ (15 mg, 0.11 mmol) and $Pd(PPh_3)_4$ (0.5 mg, 0.4 µmol) to afford the title compound as an off-white solid (12 mg, 21 µmol, 75%).

$^1$H NMR (500 MHz, $CDCl_3$) δ 9.40 (d, *J* = 1.0 Hz, 1H), 8.69 (d, *J* = 6.1 Hz, 1H), 8.51 (d, *J* = 6.0 Hz, 1H), 8.48 (dd, *J* = 7.3, 1.3 Hz, 1H), 8.23 (d, *J* = 8.2 Hz, 1H), 7.71 (t, *J* = 7.3 Hz, 1H), 7.52 – 7.44 (m, 2H), 7.22 (d, *J* = 7.9 Hz, 2H), 7.14 (t, *J* = 8.7 Hz, 2H), 6.90 (d, *J* = 7.6 Hz, 2H), 4.71 (s, 1H), 4.18 – 4.10 (m, 1H), 3.22 (s, 1H), 3.13 – 3.05 (m, 1H), 2.27 – 2.17 (m, 1H), 2.07 – 1.98 (m, 1H), 1.79 – 1.70 (m, 1H), 1.62 (s, 1H), 1.16 (s, 9H).

$^{13}$C NMR (126 MHz, $CDCl_3$) δ 161.61 (d, *J* = 247 Hz), 156.30, 152.30, 144.55, 141.65, 137.91, 135.91, 133.70, 132.39, 130.54, 128.30, 127.62 (d, *J* = 7.9 Hz), 125.94, 125.01, 124.84, 116.76, 114.83 (d, *J* = 21 Hz), 80.36, 62.39, 57.17, 48.52, 33.58, 27.99, 27.14.

***tert*-Butyl (2*S*,5*R*)-2-((isoquinoline-5-sulfonamido)methyl)-5-(4-(pyridin-3-yl)phenyl)pyrrolidine-1-carboxylate (130g)**

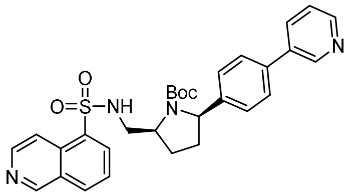

Following general procedure D, **129a** (15 mg, 27 µmol) was reacted with pyridin-3-ylboronic acid (4.0 mg, 33 µmol), $K_2CO_3$ (15 mg, 0.11 mmol) and $Pd(PPh_3)_4$ (0.5 mg, 0.4 µmol) to afford the title compound as an off-white solid (11 mg, 21 µmol, 76%).

$^1$H NMR (400 MHz, $CDCl_3$) δ 9.41 (d, *J* = 0.8 Hz, 1H), 8.80 (d, *J* = 1.5 Hz, 1H), 8.69 (d, *J* = 6.1 Hz, 1H), 8.60 (dd, *J* = 4.8, 1.6 Hz, 1H), 8.51 (d, *J* = 6.1 Hz, 1H), 8.48 (dd, *J* = 7.3, 1.2 Hz, 1H), 8.25 (d, *J* = 8.2 Hz, 1H), 7.84 (dt, *J* = 7.9, 1.8 Hz, 1H), 7.72 (t, *J* = 7.6 Hz, 1H), 7.39 (dd, *J* = 7.0, 4.8 Hz, 1H), 7.29 (s, 2H), 6.96 (d, *J* = 7.8 Hz, 2H), 4.73 (s, 1H), 4.23 – 4.10 (m, 1H), 3.31 – 3.18 (m, 1H), 3.10 (t, *J* = 10.9 Hz, 1H), 2.30 – 2.19 (m, 1H), 2.10 – 2.00 (m, 2H), 1.79 – 1.69 (m, 1H), 1.66 – 1.58 (m, 1H), 1.16 (s, 9H).

NMR spectra of key compounds

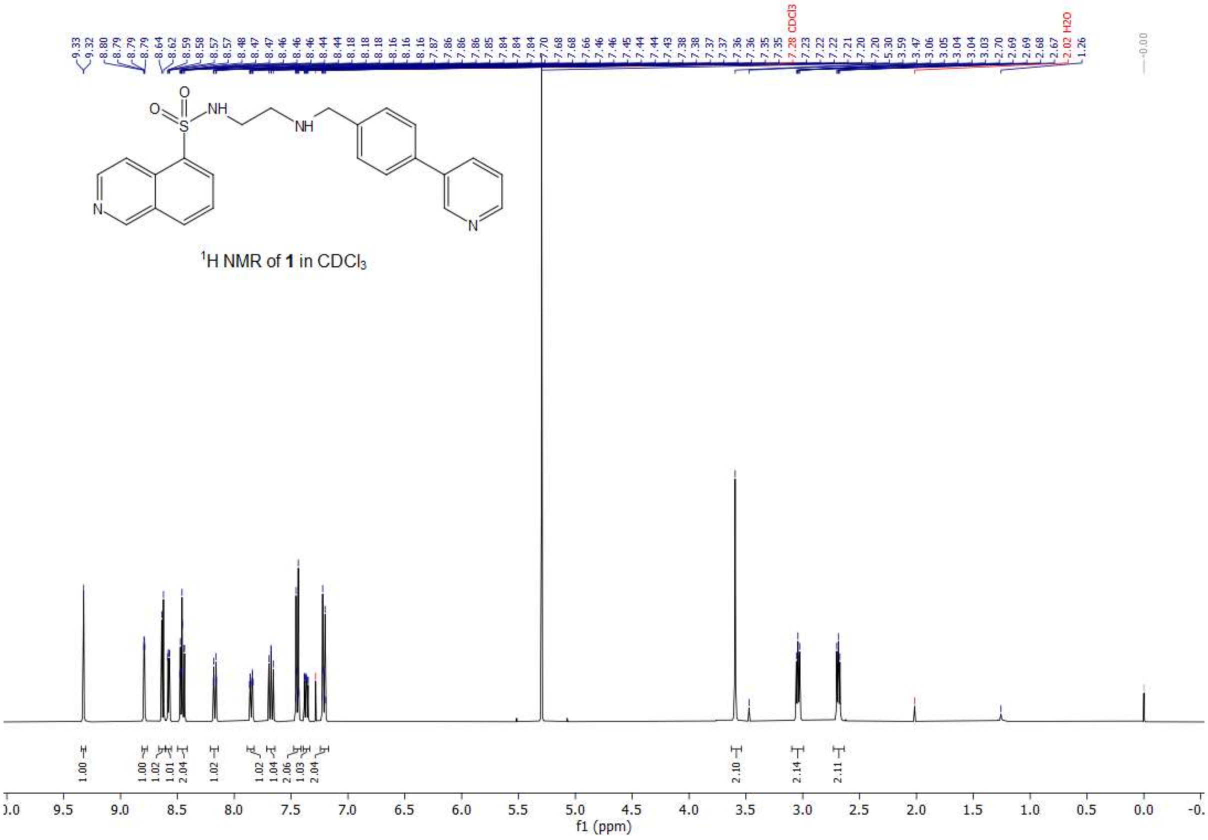


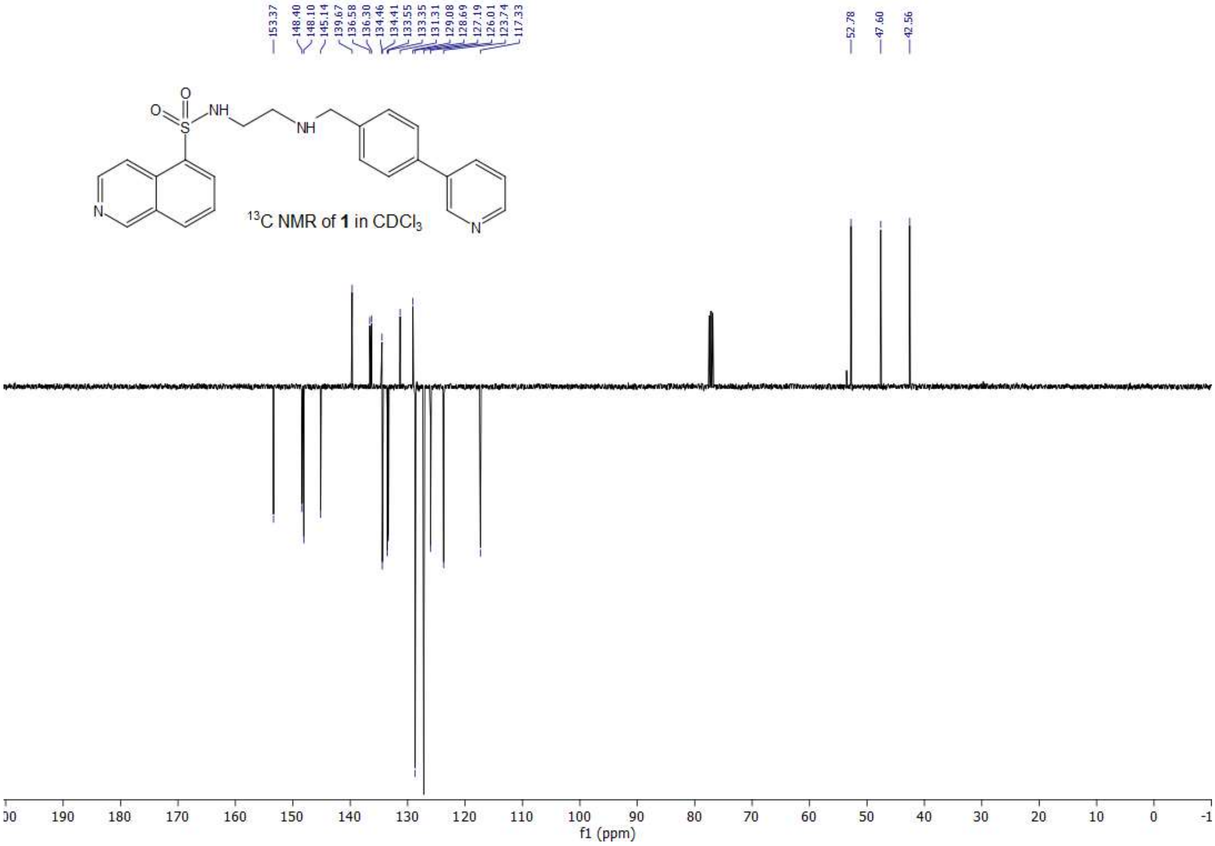

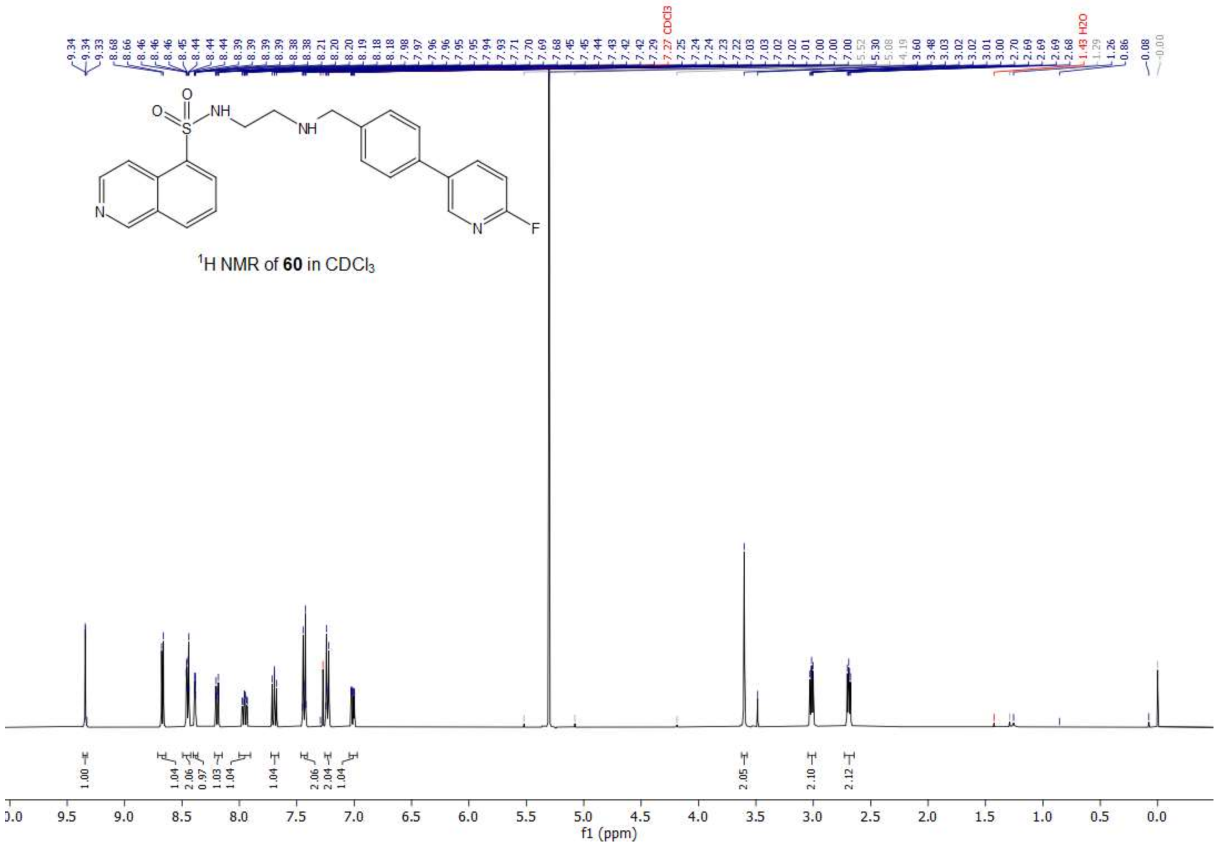

$^{1}$H NMR of 60 in CDCl3
f1 (ppm)


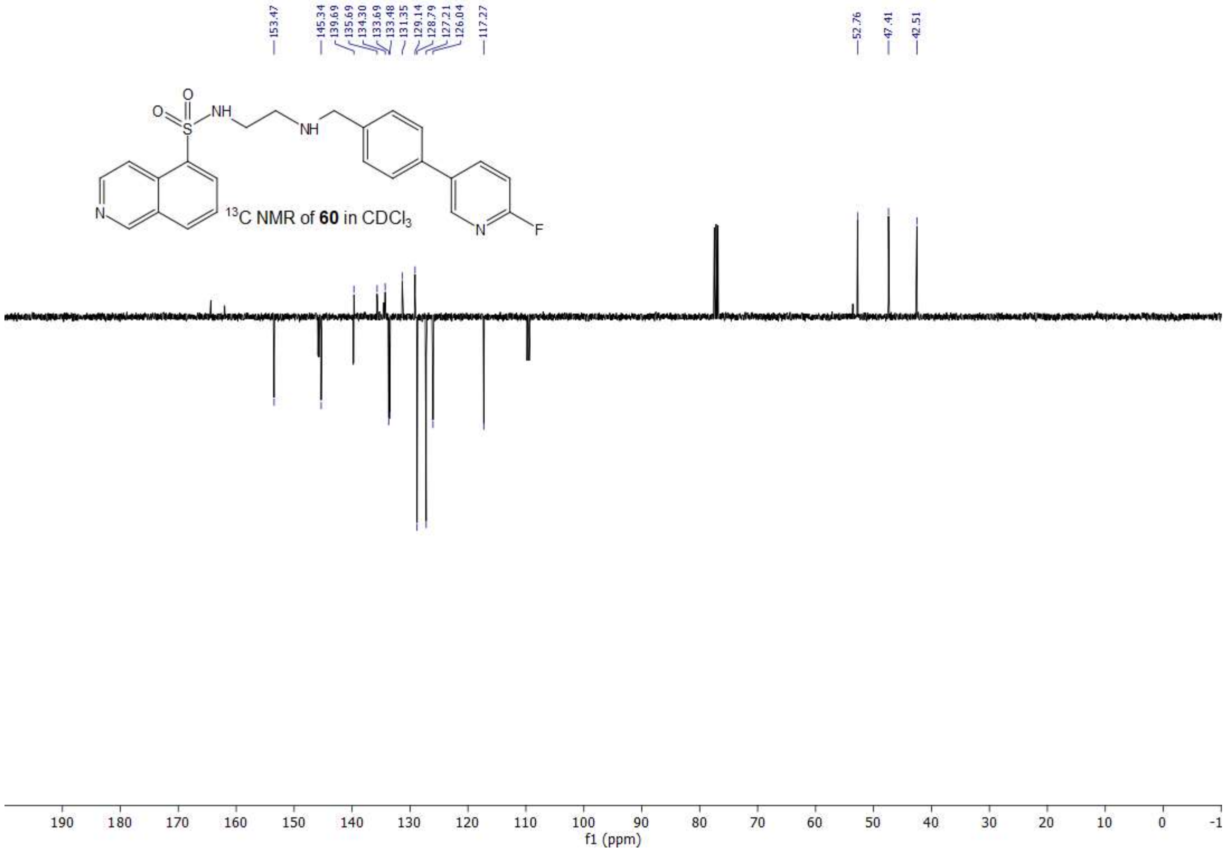

153.47
145.34
139.69
135.69
134.30
133.69
133.48
131.35
129.14
128.79
127.21
126.04
117.27
52.76
47.41
42.51
$^{13}$C NMR of 60 in CDCl3
f1 (ppm)

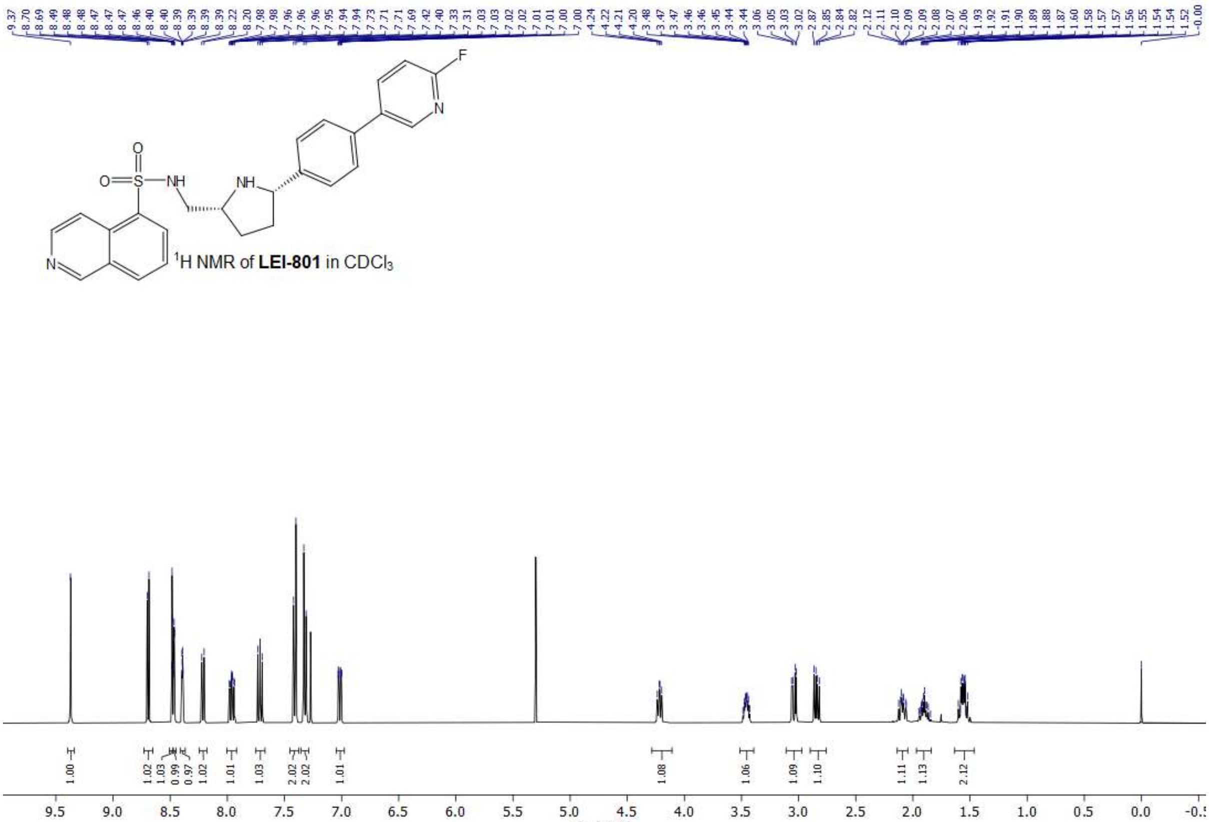


$^{1}$H NMR of **LEI-801** in $CDCl_3$

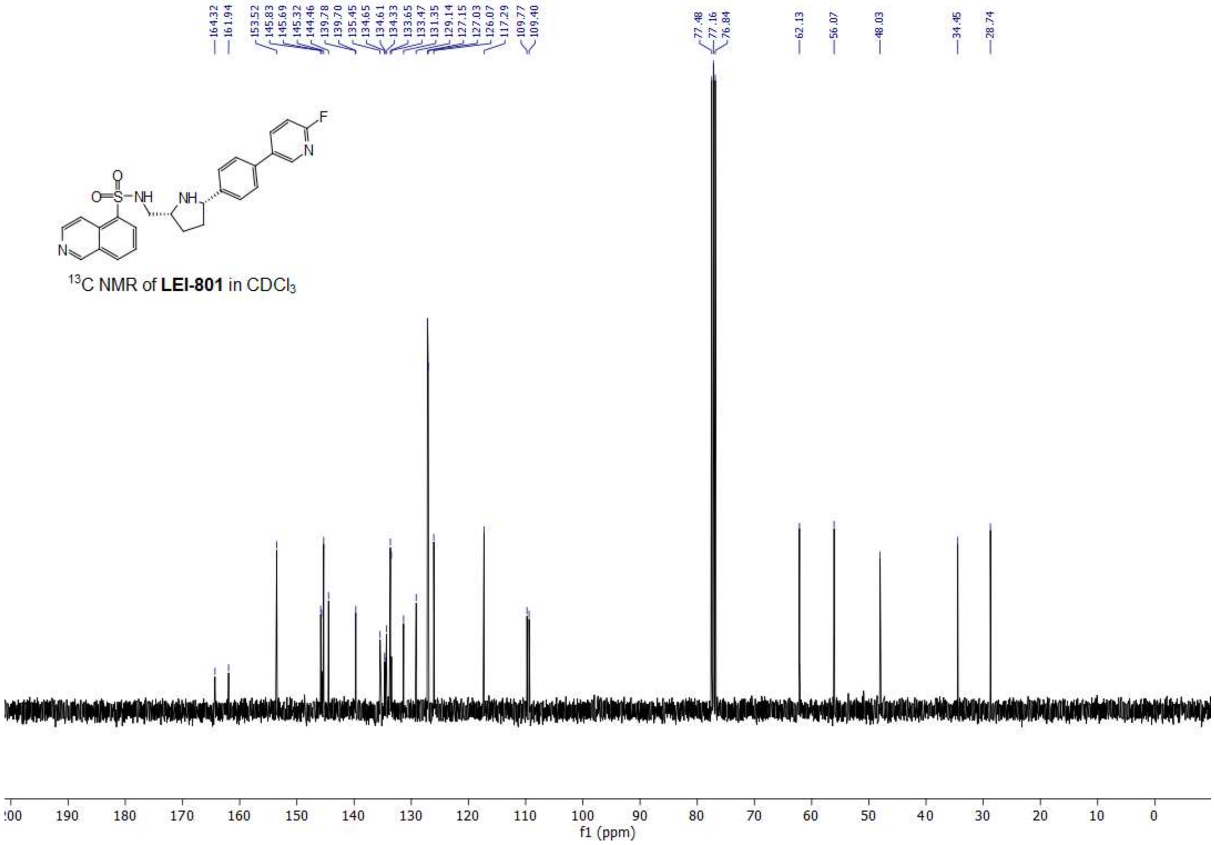


$^{13}$C NMR of **LEI-801** in $CDCl_3$

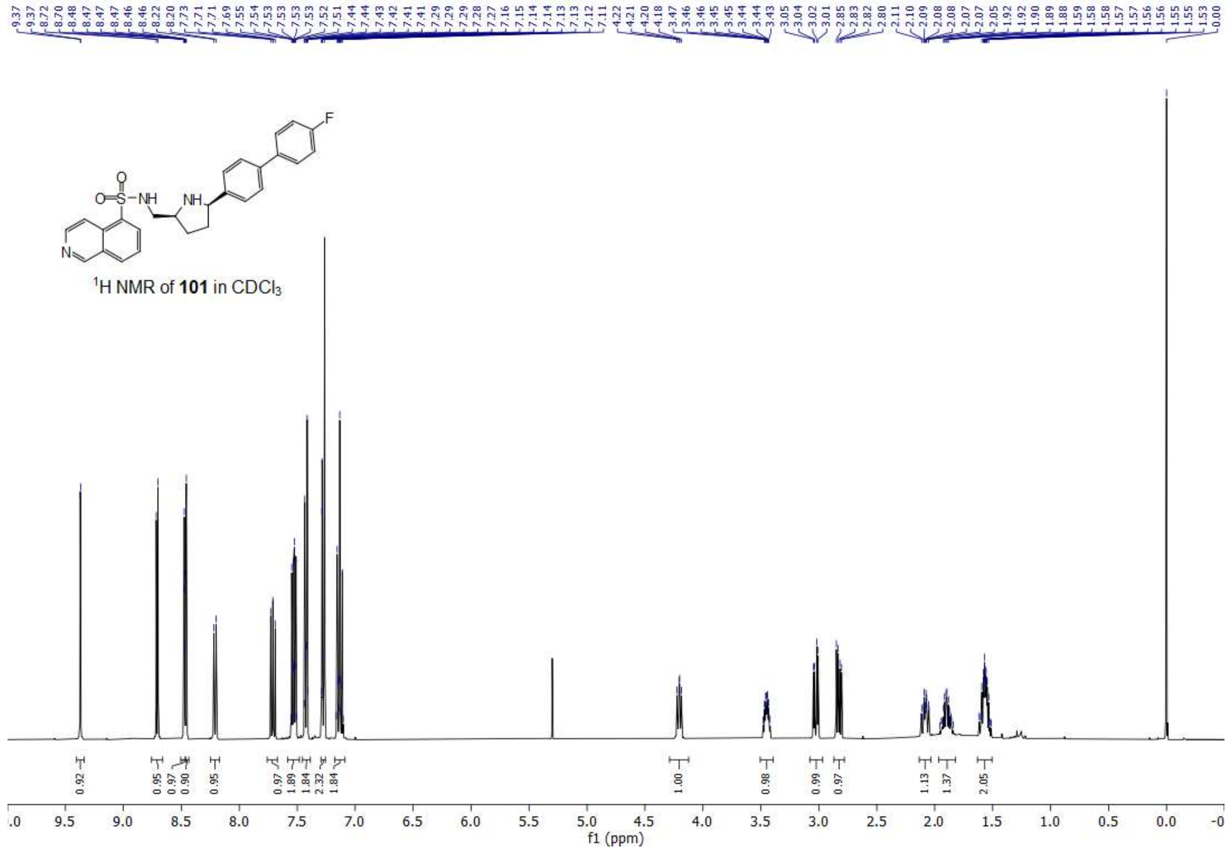

$^{1}$H NMR of 101 in CDCl3
f1 (ppm)


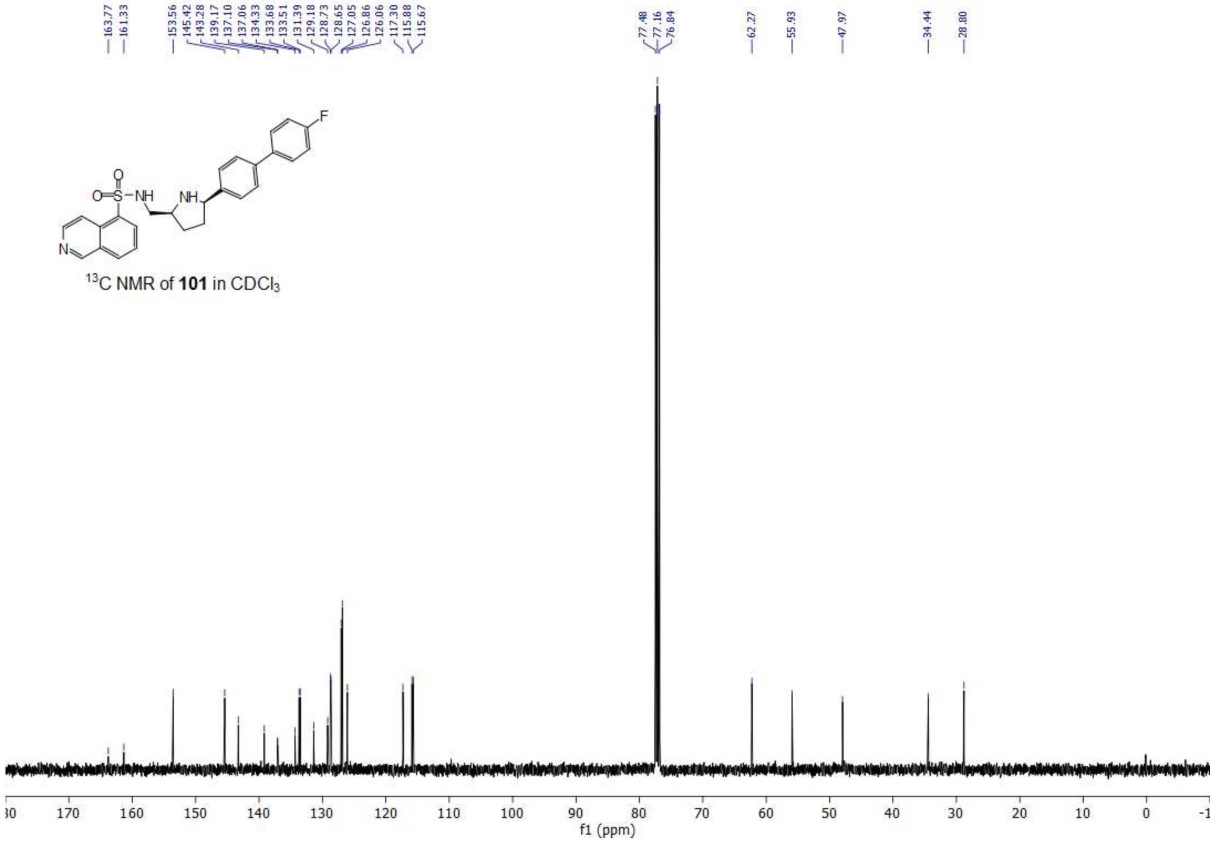

$^{13}$C NMR of 101 in CDCl3
f1 (ppm)

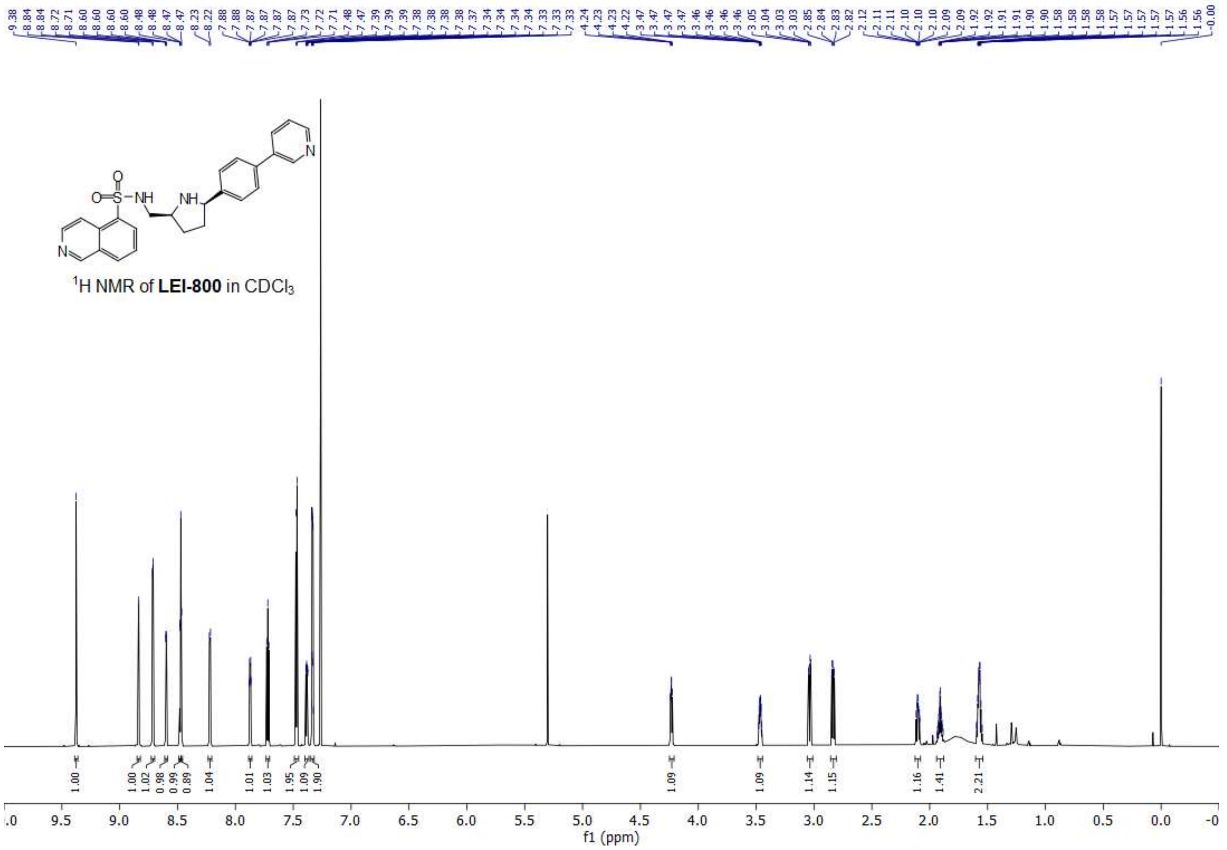
$^1$H NMR of **LEI-800** in CDCl$_3$
f1 (ppm)

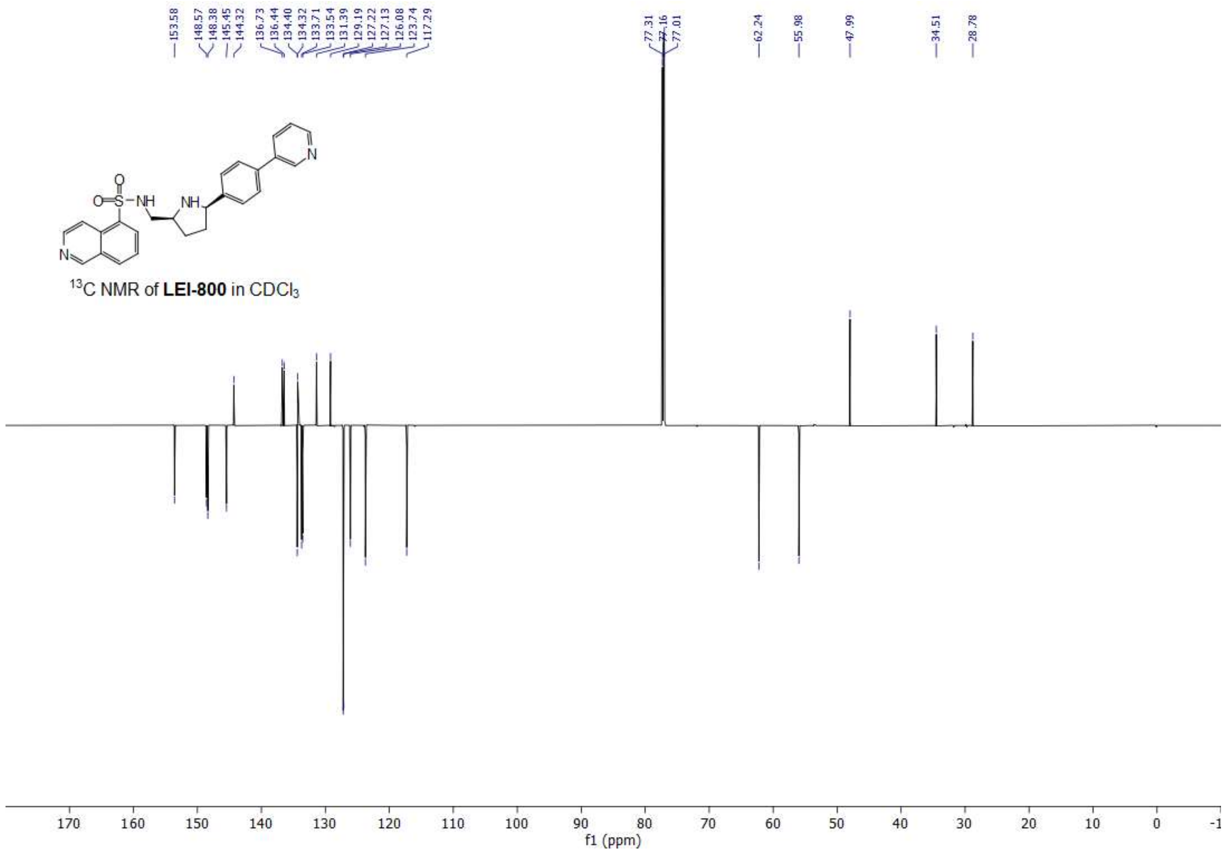
$^{13}$C NMR of **LEI-800** in CDCl$_3$
f1 (ppm)

## References


1. Schindelin, J. *et al.* Fiji: An open-source platform for biological-image analysis. *Nature Methods* **9**, 676–682 (2012).
2. Berg, S. *et al.* Ilastik: Interactive Machine Learning for (Bio)Image Analysis. *Nature Methods* **16**, 1226–1232 (2019).
3. Ducret, A., Quardokus, E. M. & Brun, Y. V. MicrobeJ, a tool for high throughput bacterial cell detection and quantitative analysis. *Nature Microbiology* **1**, 1–7 (2016).
4. Jensen, K. F. The Escherichia coli K-12 'wild types' W3110 and MG1655 have an rph frameshift mutation that leads to pyrimidine starvation due to low pyrE expression levels. *Journal of Bacteriology* **175**, 3401–3407 (1993).
5. Yuan, R. & Meselson, M. DNA Restriction Enzyme from Escherichia coli. *Methods in Enzymology* **21**, 269–273 (1971).
6. Jiang, Y. *et al.* Multigene editing in the Escherichia coli genome via the CRISPR-Cas9 system. *Applied and Environmental Microbiology* **81**, 2506–2514 (2015).
7. Tan, Y. Z. *et al.* Addressing preferred specimen orientation in single-particle cryo-EM through tilting. *Nat Methods* **14**, 793–796 (2017).

## Source data uncropped gels

**Source data for the Supplementary Figure 5**

Uncropped gels (top to bottom: LEI-800, **60**)

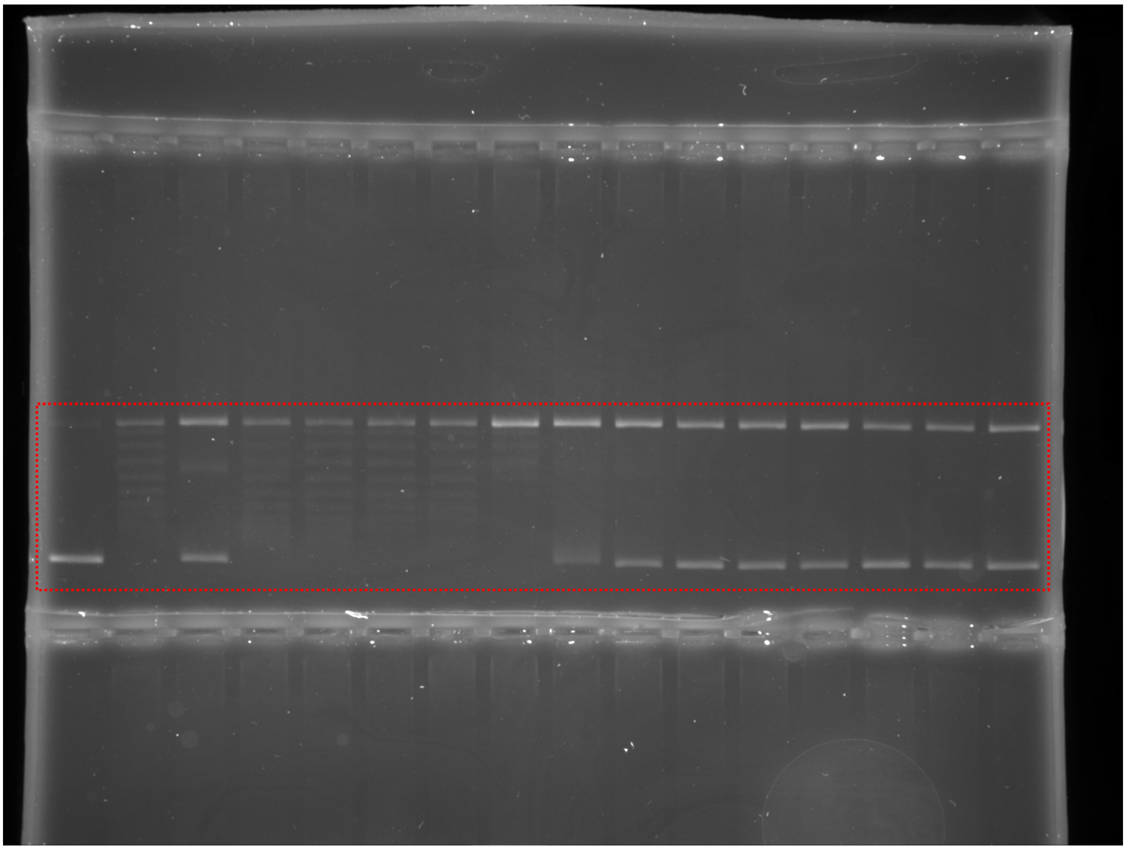

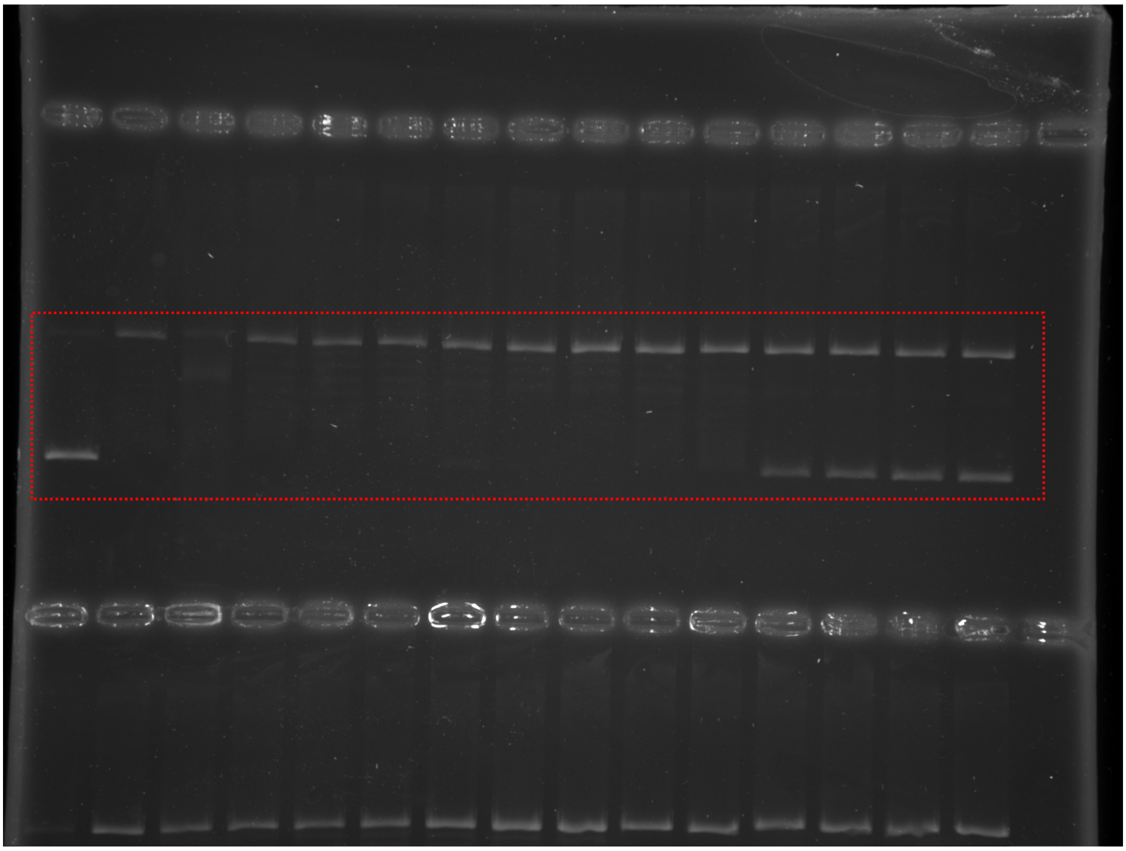

**Source data for the Supplementary Figure 8 (a)**

Uncropped gels (top to bottom: S97L, WT, S172A)

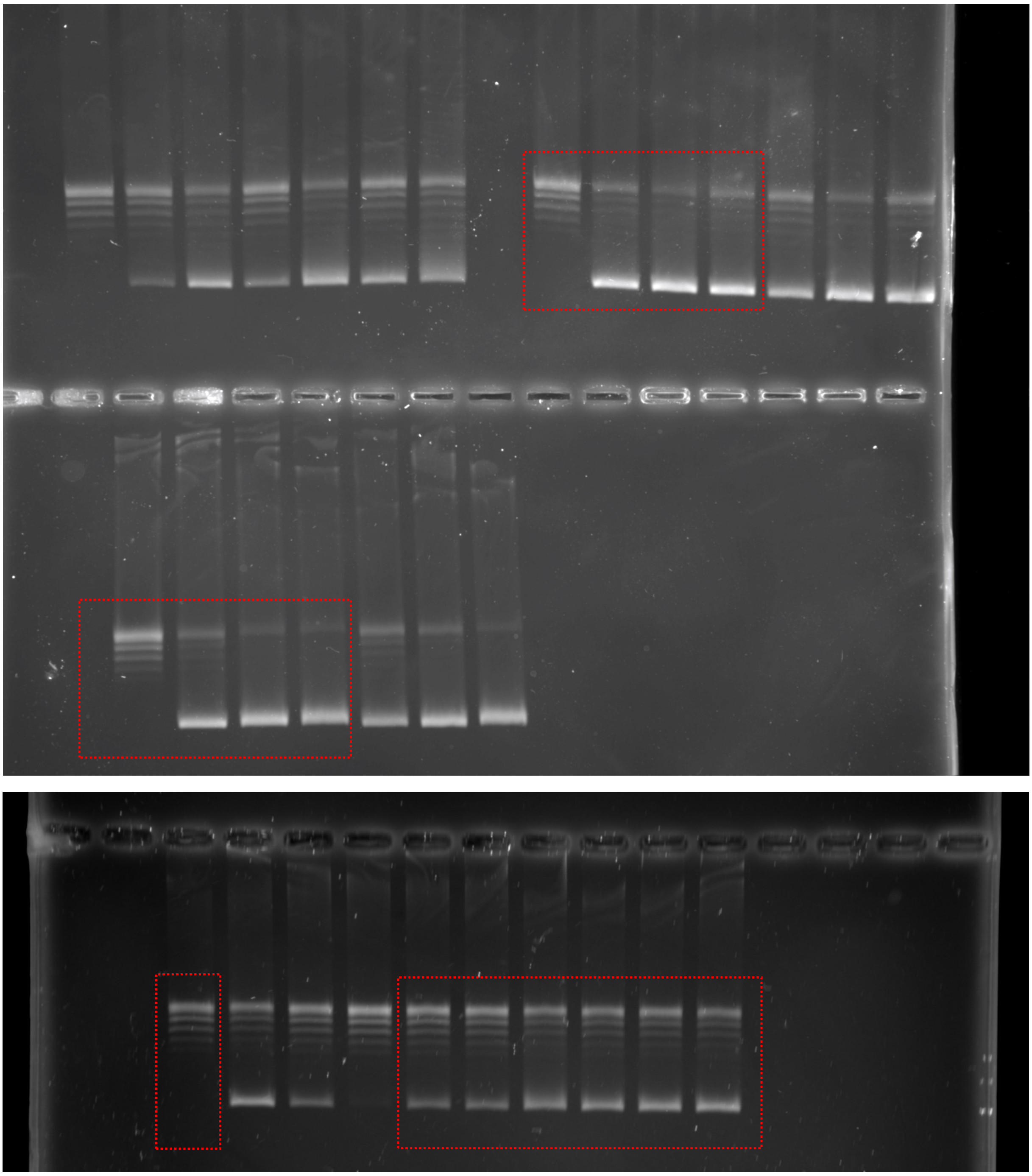

**Source data for the Supplementary Figure 8 (b,c)**

Uncropped gels for Ec GyrA S97L+LEI-800 (expts 1,2,3)

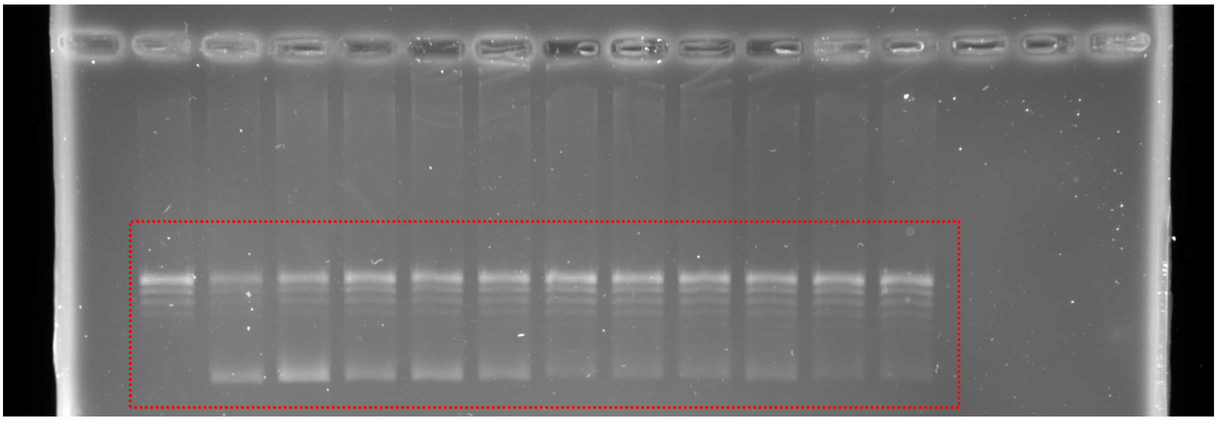

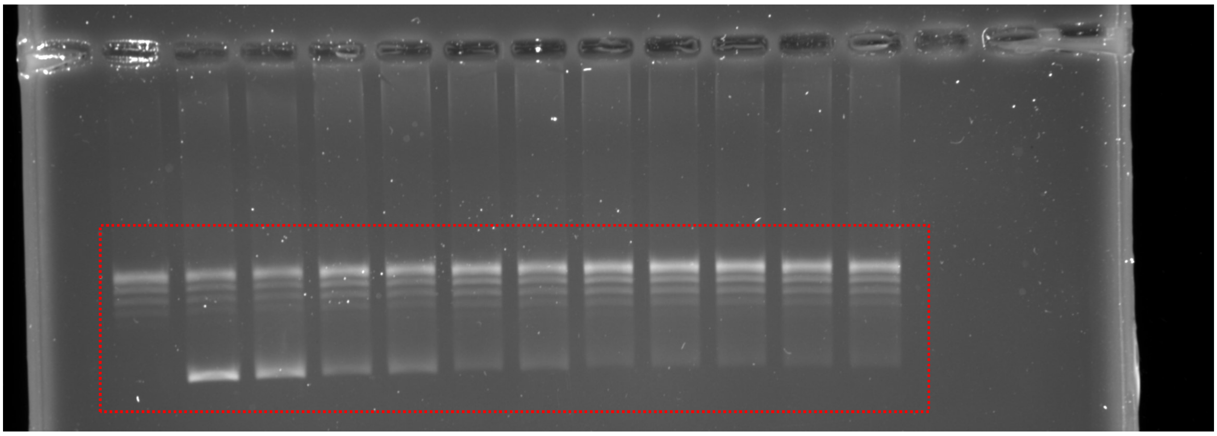

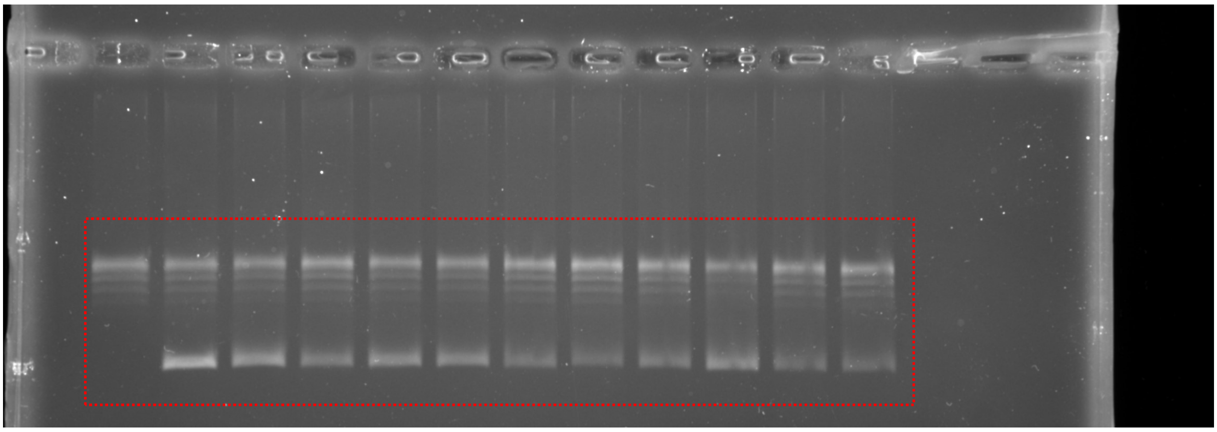

**Source data for the Supplementary Figure 8 (b,c)**

Uncropped gels for Ec GyrA S97L+LEI-801 (expts 1,2,3)

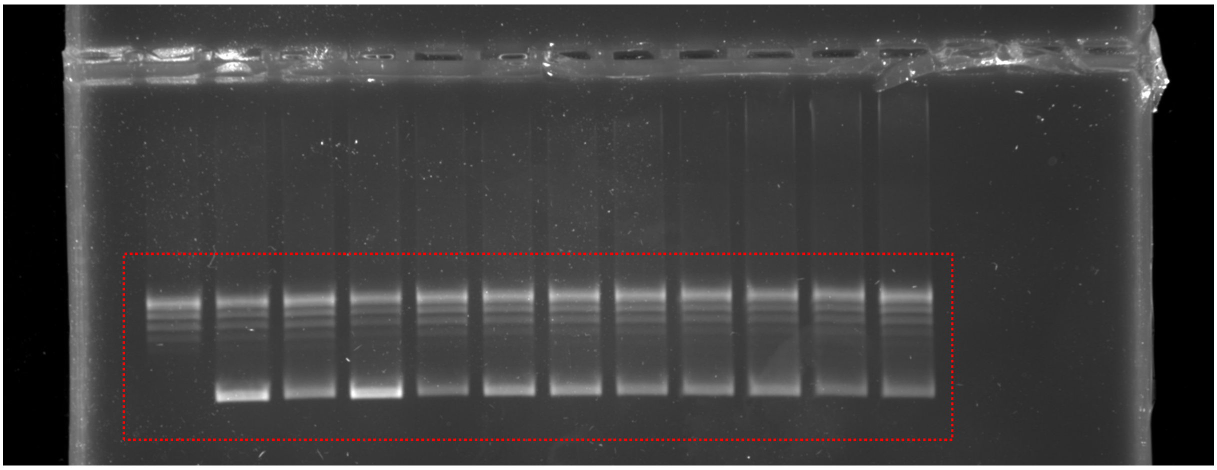

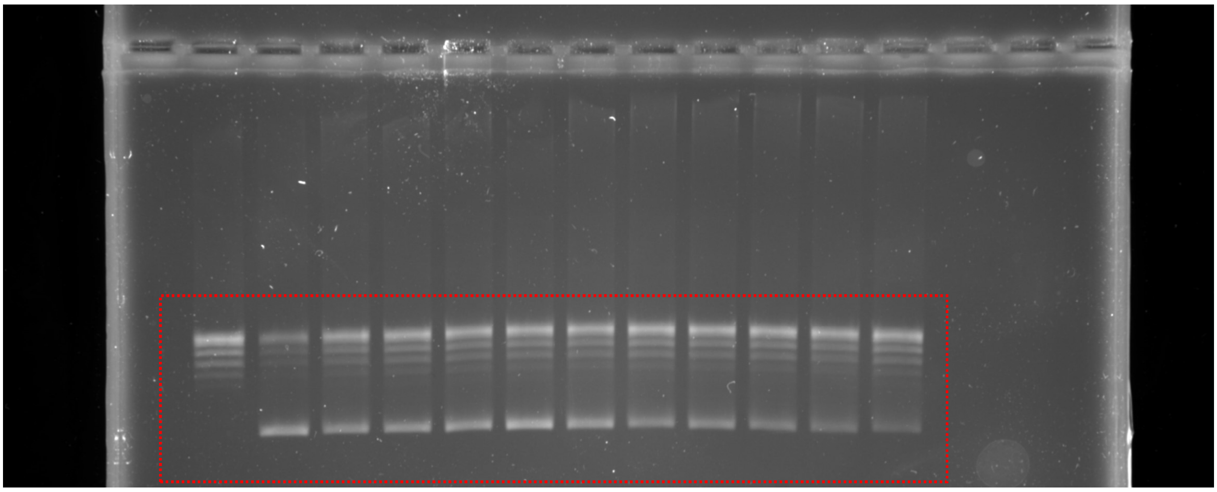

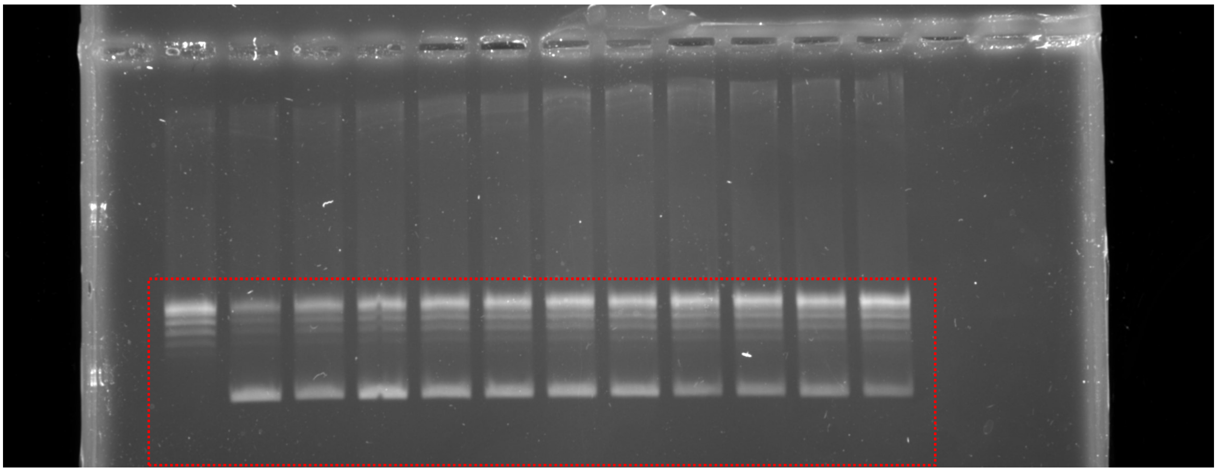

**Source Data for the Supplementary Figure 9 (a,b)**

Uncropped gels used for Ec Gyr S172A+LEI-800 – expts 1,2,3

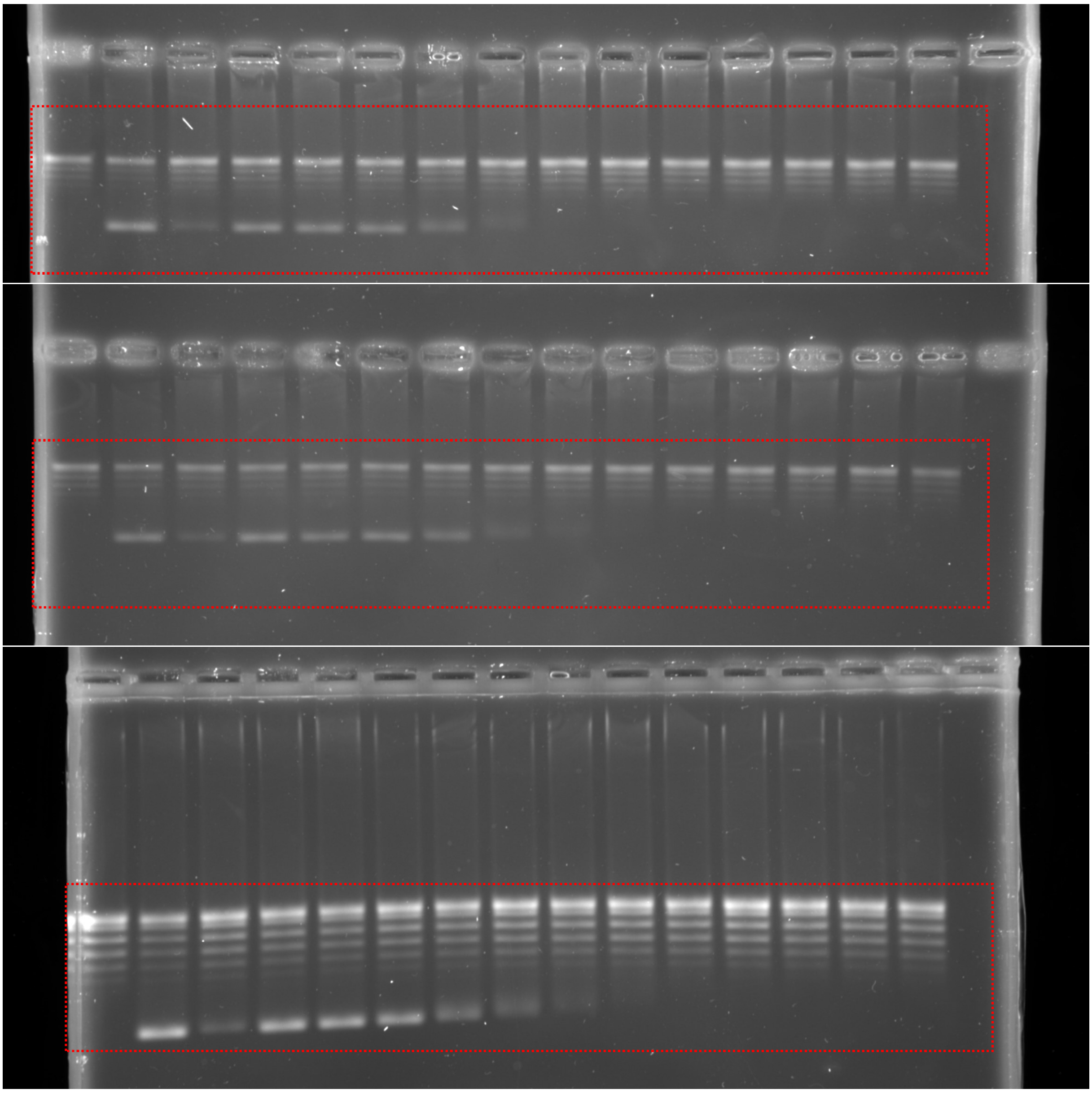

**Source Data for the Supplementary Figure 9 (a,b)**

Uncropped gels used for Ec Gyr S172A+LEI-801 – expts 1,2,3

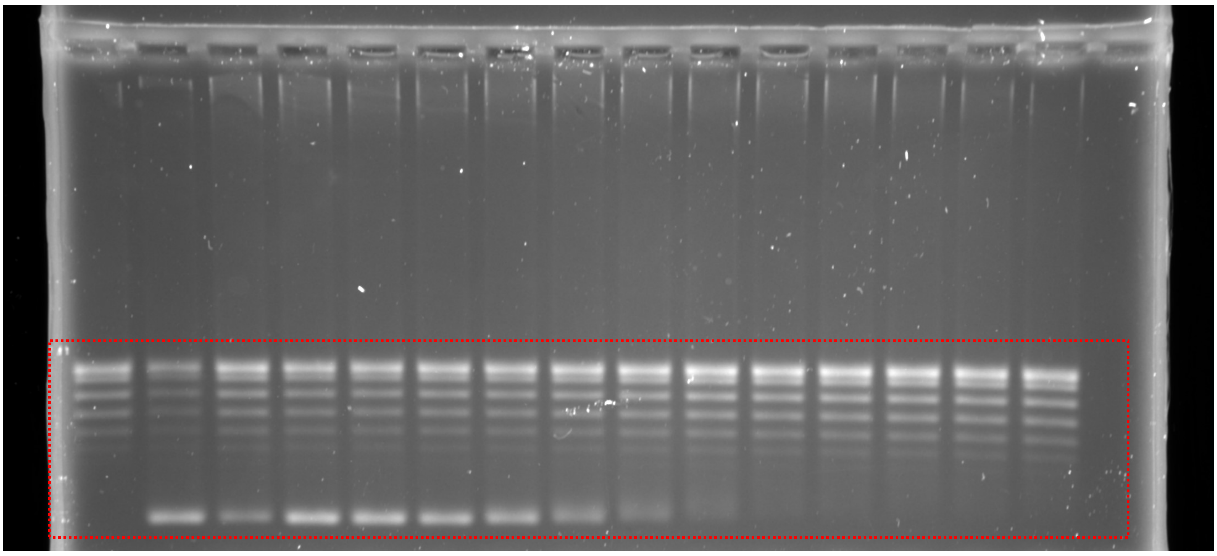

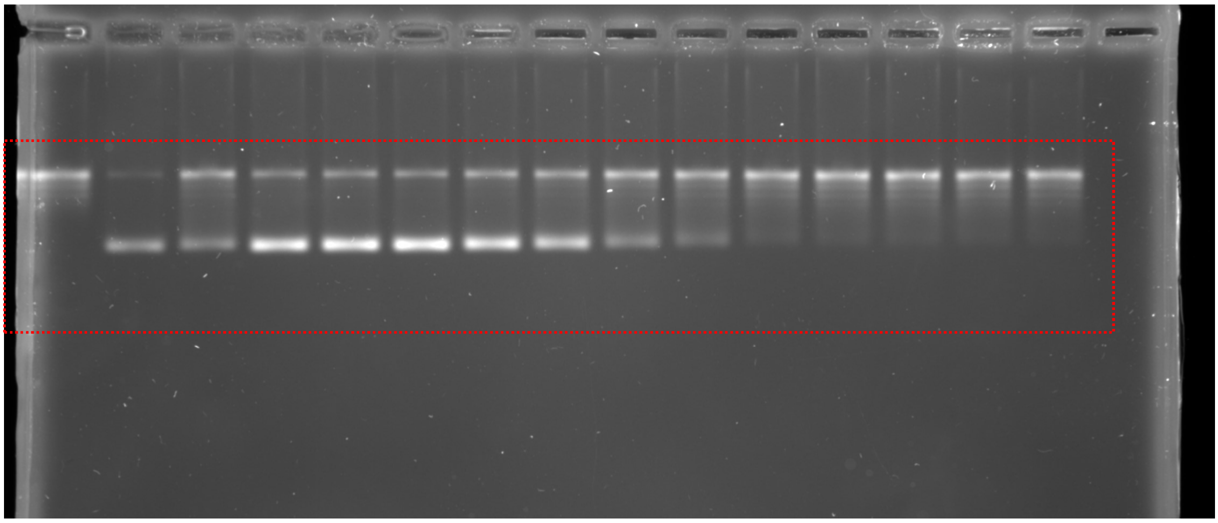

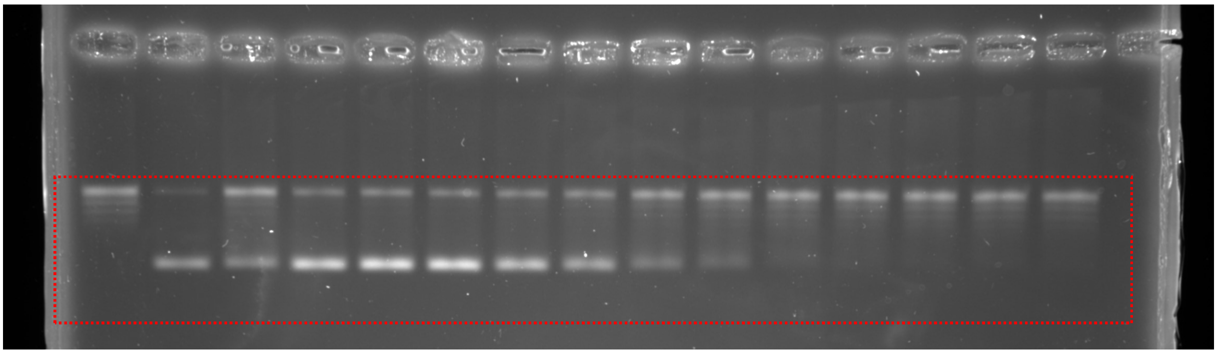

**Source data for Supplementary Figure 11 (c)**

Uncropped gels used for DNA quantification Ab gyrase+LEI-800 (expt 1,2,3)

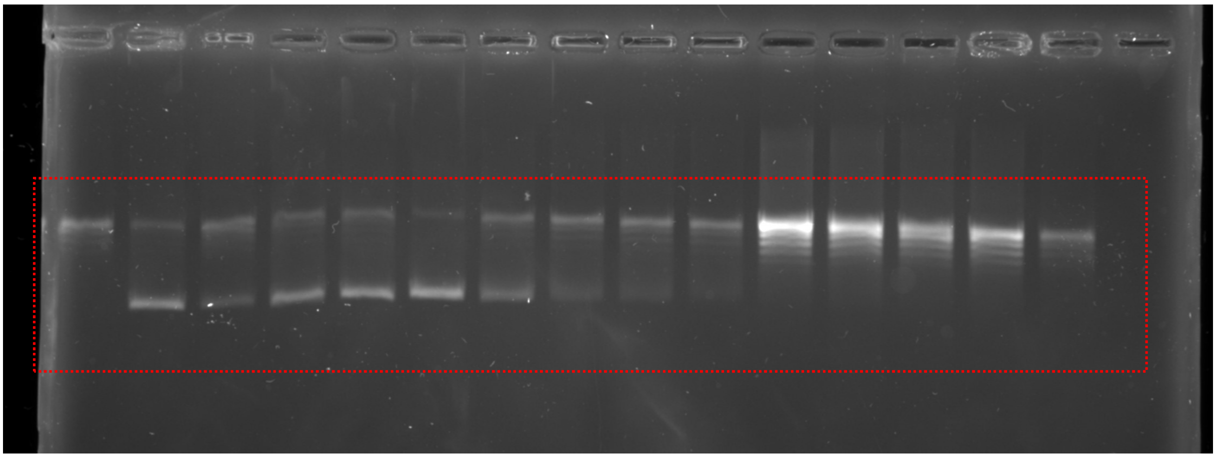

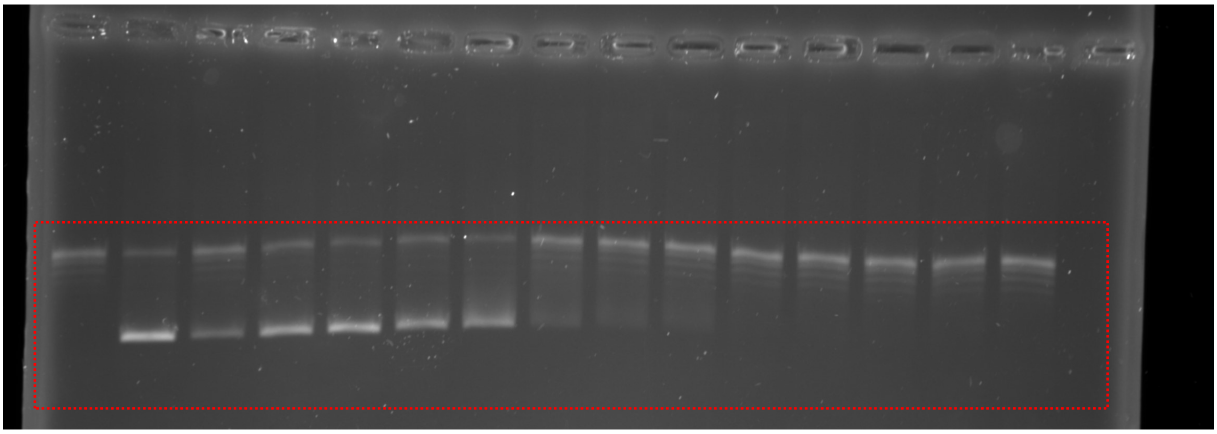

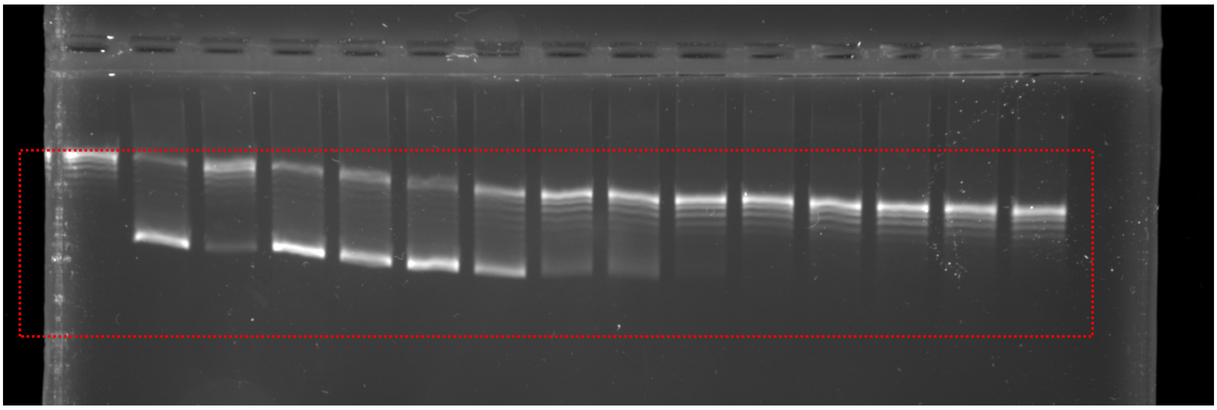

**Source data for Supplementary Figure 11 (c)**

Uncropped gels used for DNA quantification Pa gyrase + LEI-800 (expt 1,2,3)

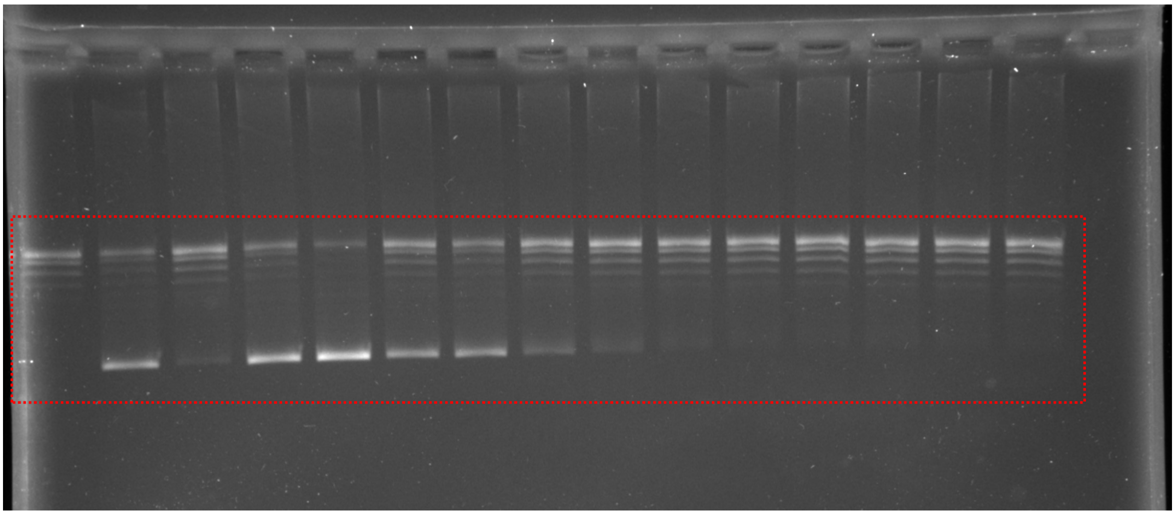

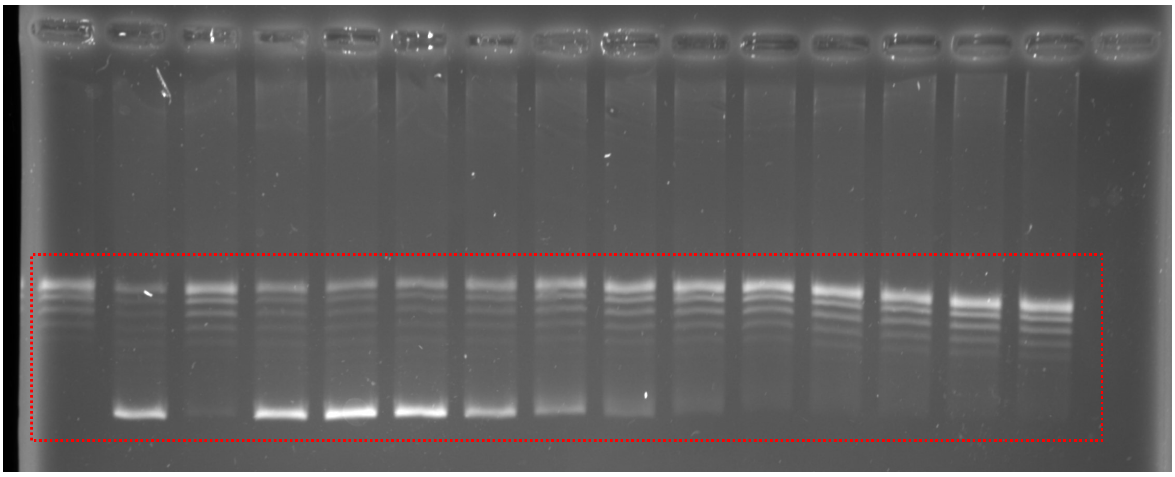

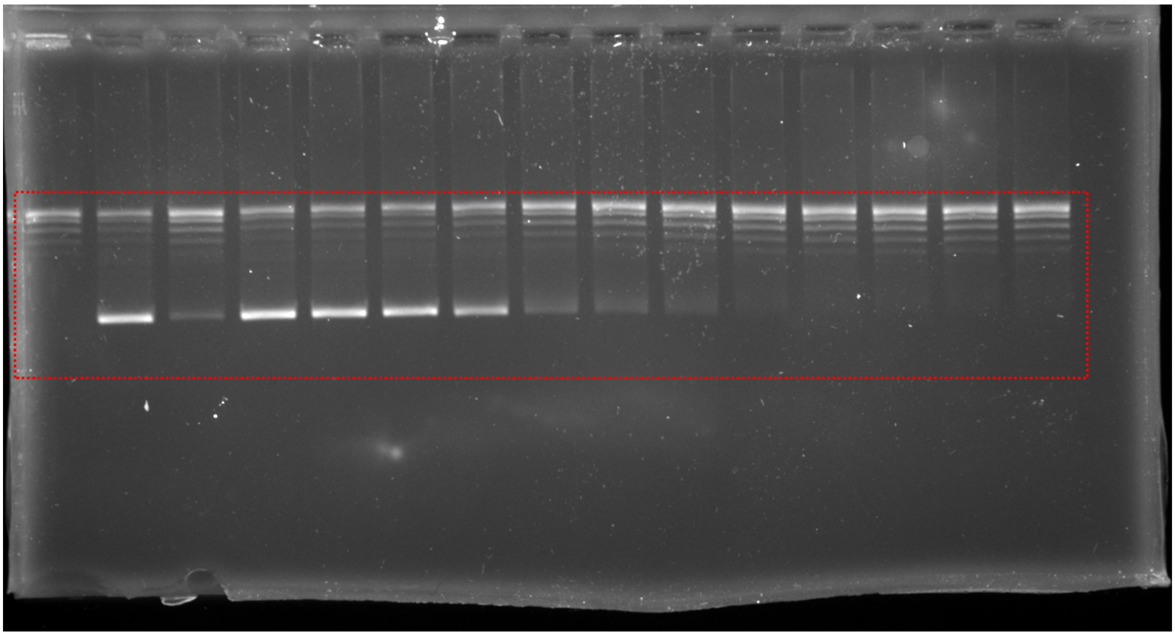

**Source Data for the Supplementary Figure 12 (e)**

Uncropped gels (expts. 1,2,3) – EcGyr+CIP (expts 1,2,3)

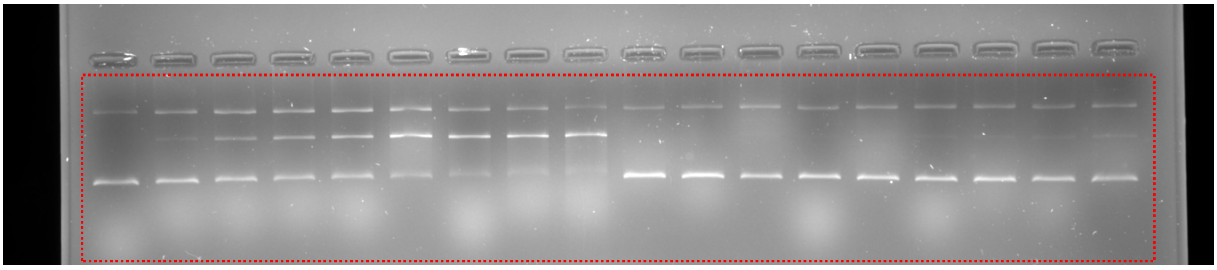

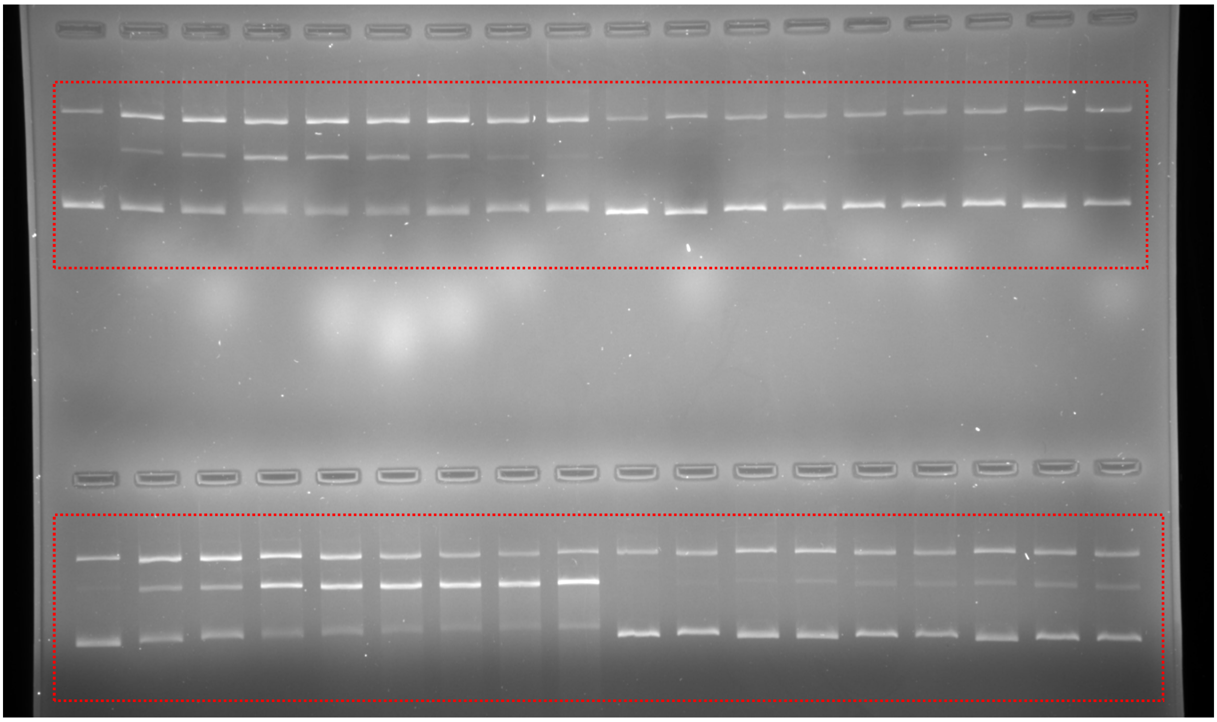

**Source Data for the Supplementary Figure 12 (e)**

Uncropped gels (expts. 1,2,3) – EcGyr+$Ca^{2+}$

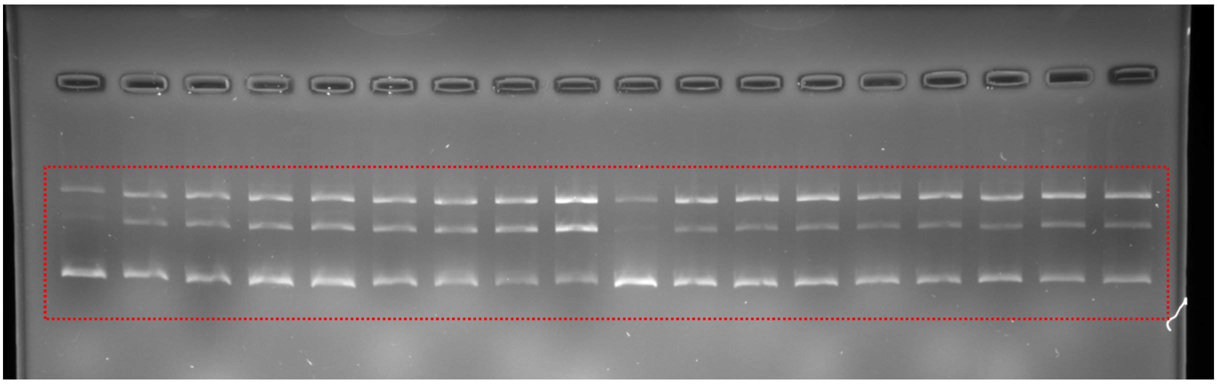

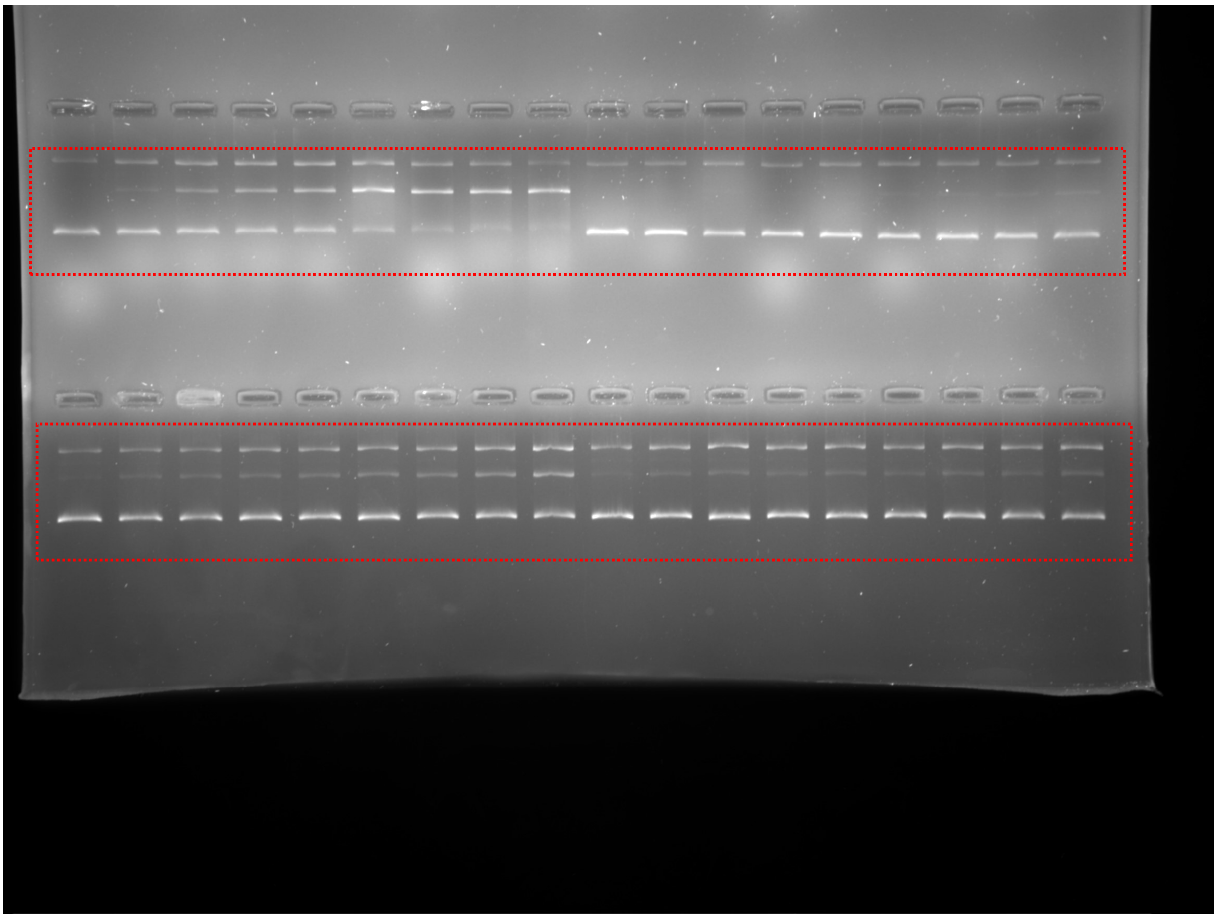

Supplement: Supplementary file 1 — Supplementary Tables 1–18, Figs. 1–12, synthetic procedures and NMR spectra key compounds, and source data uncropped gels. [file 41557_2024_1516_MOESM1_ESM.pdf]
