# Supplementary material for: Discovery of isoquinoline sulfonamides as allosteric gyrase inhibitors with activity against fluoroquinolone-resistant bacteria
Source: Nat Chem. 2024 Jun 19;16(9):1462–72. doi: 10.1038/s41557-024-01516-x (PMC11374673; doi:10.1038/s41557-024-01516-x)
Supplement: Supplementary file 2 — Reporting Summary [file 41557_2024_1516_MOESM2_ESM.pdf]

nature portfolio

Corresponding author(s): Dmitry Ghilarov; Nathaniel I. Martin; Mario van der Stelt

Last updated by author(s): Mar 6, 2024

Reporting Summary

Nature Portfolio wishes to improve the reproducibility of the work that we publish. This form provides structure for consistency and transparency in reporting. For further information on Nature Portfolio policies, see our Editorial Policies and the Editorial Policy Checklist.

Statistics

For all statistical analyses, confirm that the following items are present in the figure legend, table legend, main text, or Methods section.

n/a	Confirmed	
☐	☒	The exact sample size (n) for each experimental group/condition, given as a discrete number and unit of measurement
☐	☒	A statement on whether measurements were taken from distinct samples or whether the same sample was measured repeatedly
☒	☐	The statistical test(s) used AND whether they are one- or two-sided<br>*Only common tests should be described solely by name; describe more complex techniques in the Methods section.*
☒	☐	A description of all covariates tested
☒	☐	A description of any assumptions or corrections, such as tests of normality and adjustment for multiple comparisons
☐	☒	A full description of the statistical parameters including central tendency (e.g. means) or other basic estimates (e.g. regression coefficient) AND variation (e.g. standard deviation) or associated estimates of uncertainty (e.g. confidence intervals)
☒	☐	For null hypothesis testing, the test statistic (e.g. F, t, r) with confidence intervals, effect sizes, degrees of freedom and P value noted<br>*Give P values as exact values whenever suitable.*
☒	☐	For Bayesian analysis, information on the choice of priors and Markov chain Monte Carlo settings
☒	☐	For hierarchical and complex designs, identification of the appropriate level for tests and full reporting of outcomes
☒	☐	Estimates of effect sizes (e.g. Cohen's d, Pearson's r), indicating how they were calculated

Our web collection on statistics for biologists contains articles on many of the points above.

Software and code

Policy information about availability of computer code

Data collection	Novaseq control software was used for Genome sequencing. MARS (V4.20) was used for absorbance data collection (MTT). Data acquisition of agarose gels was performed with Image Lab Touch Software (v2.0.0.27).
Data analysis	The acquired images from the DNA supercoiling assay were analyzed using ImageLab 6.1 software (Bio-Rad Laboratories, Inc).<br><br>For the bacterial cytological profiling (BCP) the following software and code were used: Illastik (v 1.3.3), ImageJ (v 1.53m), the ImageJ plugin MicrobeJ (v 5.131) and a custom macro available from GitHub.<br><br>Genome sequencing data were analysed using SAMtools42 (v 0.1.19) and ANNOVAR43 (v 2015MAR2 2), BreakDancer (v 1.4.4) and Burrow-Wheeler Alignment tool (BWA v0.7.841).<br><br>Cryo-EM data processing was done in cryoSPARC 3.3.1.45, DeepEMhancer, ChimeraX, Coot, ISOLDE, phenix.refine, Grade server, MolProbity, MTriage. Graphpad Prism (v 9.0) was used to perform statistical tests and generate graphs and plots.

For manuscripts utilizing custom algorithms or software that are central to the research but not yet described in published literature, software must be made available to editors and reviewers. We strongly encourage code deposition in a community repository (e.g. GitHub). See the Nature Portfolio guidelines for submitting code & software for further information.

Data

Policy information about availability of data

All manuscripts must include a data availability statement. This statement should provide the following information, where applicable:

- Accession codes, unique identifiers, or web links for publicly available datasets
- A description of any restrictions on data availability
- For clinical datasets or third party data, please ensure that the statement adheres to our policy

Source Data for Figures are provided as separate Source Data files. All Source data for Supplementary Figures are listed in the Supplementary Data file, except for uncropped gels which are listed at the end of the Supplementary Information. The raw microscopy images used for the BCP morphological analysis and quantification are available from BioImage Archive (S-BIAD1079). Whole-genome sequencing data is available at NCBI through the BioProject accession number PRJNA855320. The Gyr-LEI-800 coordinates have been submitted to the Protein Data Bank (https://www.rcsb.org/) with ID 8QQI. The corresponding EM maps have been submitted to the Electron Microscopy Data Bank (https://www.ebi.ac.uk/pdbe/emdb/) with ID EMD-18592. The raw data were submitted the Electron Microscopy Public Image Archive (https://www.ebi.ac.uk/pdbe/emdb/empiar/) with ID EMPIAR-11884.

Research involving human participants, their data, or biological material

Policy information about studies with human participants or human data. See also policy information about sex, gender (identity/presentation), and sexual orientation and race, ethnicity and racism.

Reporting on sex and gender	Not applicable.
Reporting on race, ethnicity, or other socially relevant groupings	Not applicable.
Population characteristics	Not applicable.
Recruitment	Not applicable.
Ethics oversight	Not applicable.

Note that full information on the approval of the study protocol must also be provided in the manuscript.

Field-specific reporting

Please select the one below that is the best fit for your research. If you are not sure, read the appropriate sections before making your selection.

☒ Life sciences ☐ Behavioural & social sciences ☐ Ecological, evolutionary & environmental sciences

For a reference copy of the document with all sections, see nature.com/documents/nr-reporting-summary-flat.pdf

Life sciences study design

All studies must disclose on these points even when the disclosure is negative.

Sample size	The sample size for each experiment was chosen based on our earlier published work (1,2) and in-house SOPs.<br>1) Bakker, A. T. et al. Chemical Proteomics Reveals Antibiotic Targets of Oxadiazolones in MRSA. J. Am. Chem. Soc. 145, 1136–1143 (2023).<br>2) Jiang, M. et al. A monoacylglycerol lipase inhibitor showing therapeutic efficacy in mice without central side effects or dependence. Nat Commun 14, 8039 (2023).
Data exclusions	No data were excluded.
Replication	All in vitro and cellular (mammalian and bacterial) experiments were performed in at least 2 independent expeirments (N = 2) with biological replicates (n=2). All attempts at replication were succesful.
Randomization	Not applicable.
Blinding	Not applicable.

Reporting for specific materials, systems and methods

We require information from authors about some types of materials, experimental systems and methods used in many studies. Here, indicate whether each material, system or method listed is relevant to your study. If you are not sure if a list item applies to your research, read the appropriate section before selecting a response.

Materials & experimental systems

n/a	Involved in the study
☒	☐ Antibodies
☐	☒ Eukaryotic cell lines
☒	☐ Palaeontology and archaeology
☒	☐ Animals and other organisms
☒	☐ Clinical data
☒	☐ Dual use research of concern
☒	☐ Plants

Methods

n/a	Involved in the study
☒	☐ ChIP-seq
☒	☐ Flow cytometry
☒	☐ MRI-based neuroimaging

Eukaryotic cell lines

Policy information about cell lines and Sex and Gender in Research

Cell line source(s)	HepG2 (ATCC HB-8065) and HEK293T (ATCC CRL-3216)
Authentication	The cell lines used have been authenticated by the ATCC through STR profiling.
Mycoplasma contamination	Cell lines were regularly tested for mycoplasma contamination and were tested negative
Commonly misidentified lines (See ICLAC register)	No misidentified cell lines were used.

Plants

Seed stocks	The study did not involve plants.
Novel plant genotypes	The study did not involve plants.
Authentication	This information is not applicable.
