# Supplementary material for: Discovery of isoquinoline sulfonamides as allosteric gyrase inhibitors with activity against fluoroquinolone-resistant bacteria
Source: Nat Chem. 2024 Jun 19;16(9):1462–72. doi: 10.1038/s41557-024-01516-x (PMC11374673; doi:10.1038/s41557-024-01516-x)

Source data Figure 4c,d

Ciprofloxacin: all three gels used for quantification figure 4c, top gel displayed in figure 4d

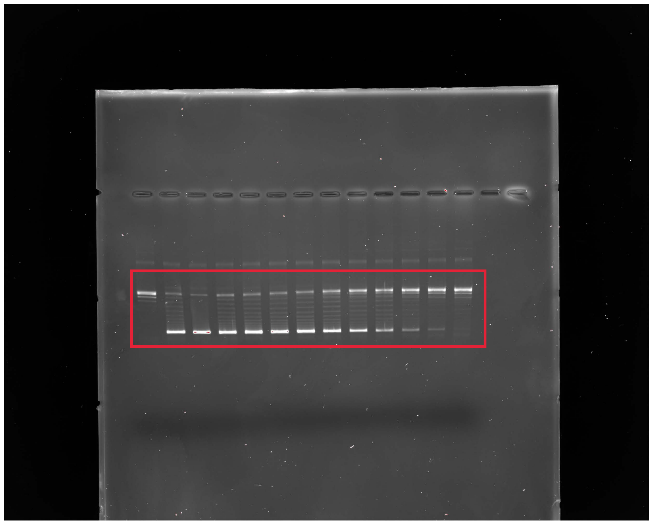

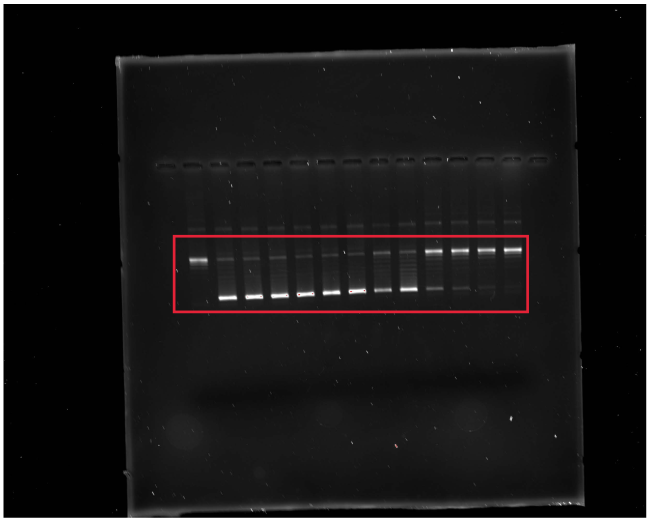

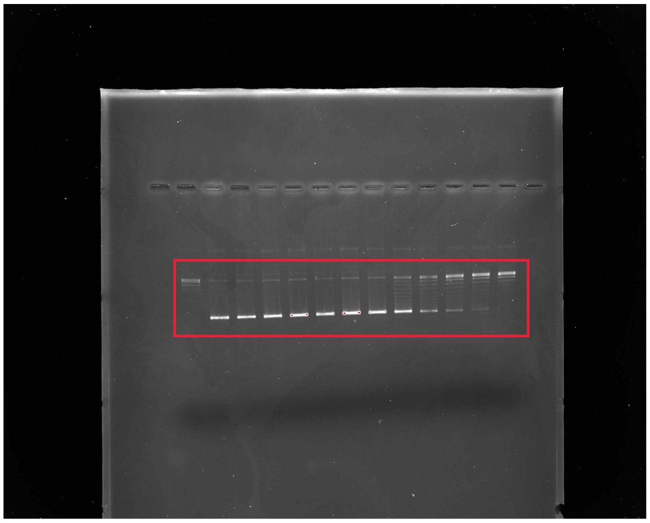

LEI-801: all three gels used for quantification figure 4c, top gel displayed in figure 4d

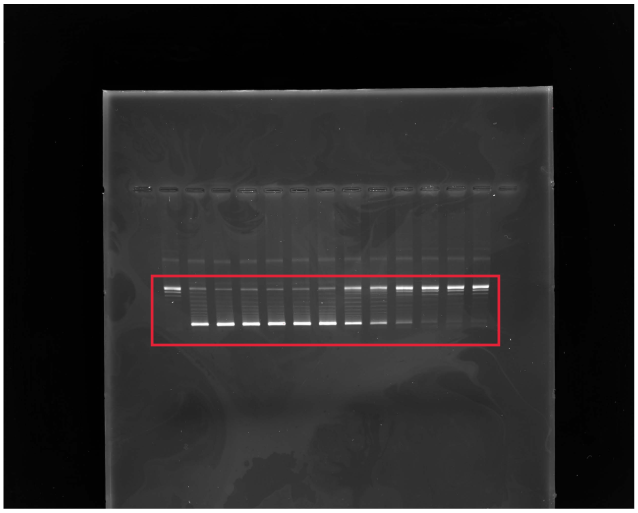

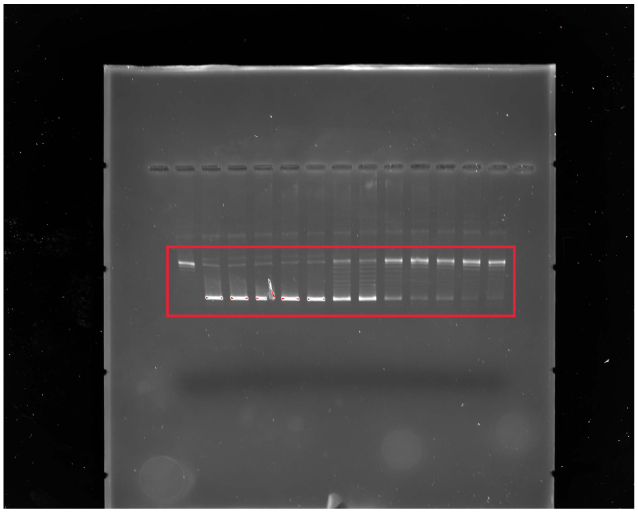

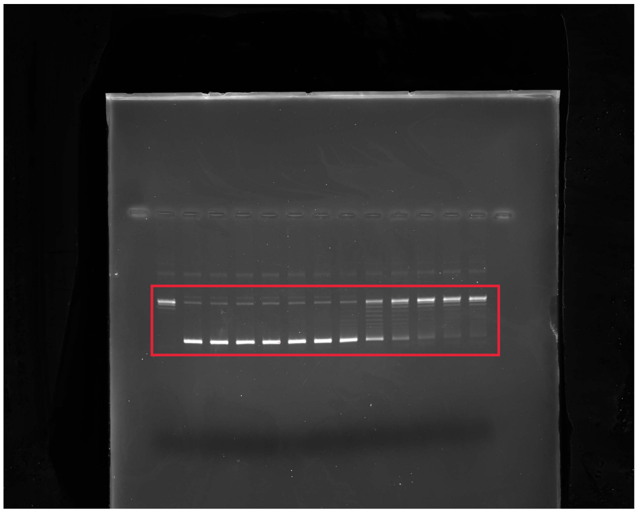

LEI-800: all three gels used for quantification figure 4c, top gel displayed in figure 4d

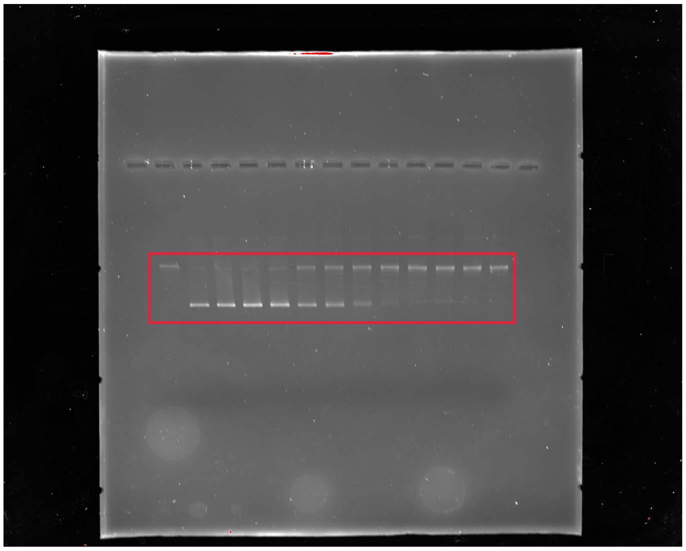

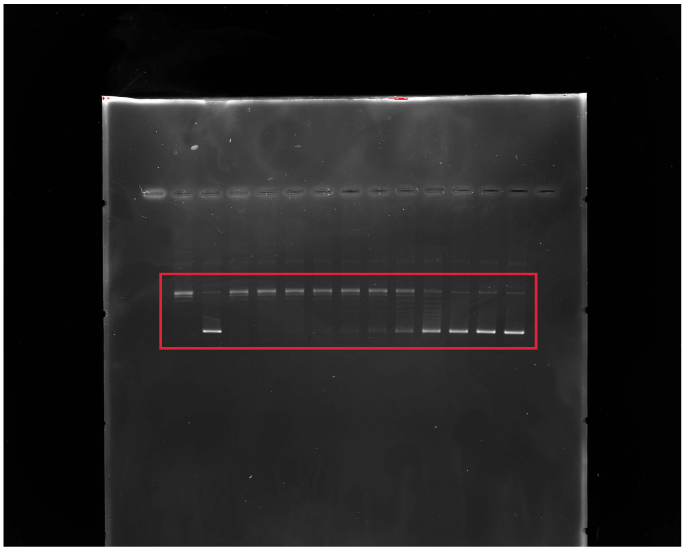

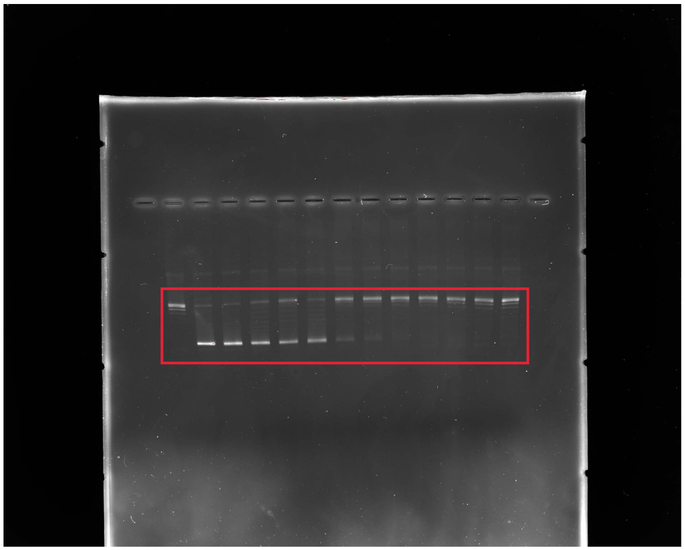

Supplement: Supplementary file 4 — Uncropped gels used for quantification in Fig. 4c and example gels in Fig. 4d. [file 41557_2024_1516_MOESM4_ESM.pdf]
